# Identification of a novel ligand for the ATAD2 bromodomain with selectivity over BRD4 through a fragment growing approach†
†Electronic supplementary information (ESI) available. See DOI: 10.1039/c8ob00099a


**DOI:** 10.1039/c8ob00099a

**Published:** 2018-02-15

**Authors:** Duncan C. Miller, Mathew P. Martin, Santosh Adhikari, Alfie Brennan, Jane A. Endicott, Bernard T. Golding, Ian R. Hardcastle, Amy Heptinstall, Stephen Hobson, Claire Jennings, Lauren Molyneux, Yvonne Ng, Stephen R. Wedge, Martin E. M. Noble, Celine Cano

**Affiliations:** a Newcastle Drug Discovery , Northern Institute for Cancer Research , School of Chemistry , Newcastle University , Newcastle upon Tyne , NE1 7RU , UK . Email: celine.cano@ncl.ac.uk ; Tel: +44 (0)191 208 7060; b Newcastle Drug Discovery , Northern Institute for Cancer Research , Paul O'Gorman Building , Medical School , Framlington Place , Newcastle upon Tyne NE2 4HH , UK . Email: martin.noble@ncl.ac.uk ; Tel: +44 (0)191 208 4466

## Abstract

ATAD2 is an ATPase that is overexpressed in a variety of cancers and associated with a poor patient prognosis. This protein has been suggested to function as a cofactor for a range of transcription factors, including the proto-oncogene MYC and the androgen receptor. ATAD2 comprises an ATPase domain, implicated in chromatin remodelling, and a bromodomain which allows it to interact with acetylated histone tails. Dissection of the functional roles of these two domains would benefit from the availability of selective, cell-permeable pharmacological probes. An *in silico* evaluation of the 3D structures of various bromodomains suggested that developing small molecule ligands for the bromodomain of ATAD2 is likely to be challenging, although recent reports have shown that ATAD2 bromodomain ligands can be identified. We report a structure-guided fragment-based approach to identify lead compounds for ATAD2 bromodomain inhibitor development. Our findings indicate that the ATAD2 bromodomain can accommodate fragment hits (*M*_r_ < 200) that yield productive structure–activity relationships, and structure-guided design enabled the introduction of selectivity over BRD4.

## Abstract 2

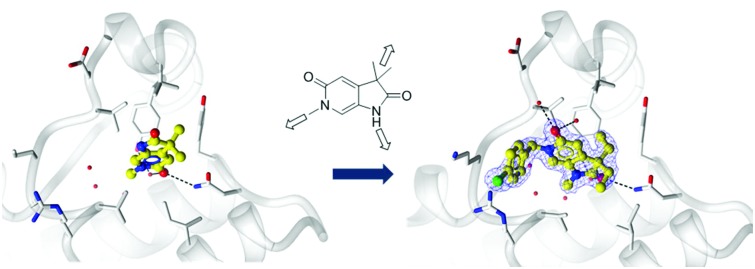
Structure-guided expansion of a fragment hit for the ATAD2 bromodomain enabled improvement in ATAD2 inhibition and selectivity over BRD4.

## Introduction

Histone proteins undergo several post translational modifications (PTMs) that affect DNA transcription, replication and repair, and genomic architecture.^1^ Histone PTMs are regulated by three groups of proteins known as writers, erasers and readers. Bromodomains are a class of protein interaction modules, conserved across evolution, which recognise ε-*N*-acetyl lysine PTMs.^2^ A total of 61 bromodomains in 46 diverse proteins have been identified in the human proteome, and these are divided into eight structural classes. Within subfamily IV in the structure-based classification of bromodomain-containing proteins is ATAD2 (ATPase Family, AAA Domain Containing 2). ATAD2 is overexpressed in a wide range of human cancers, including breast, lung, and prostate carcinomas, and it is present in low levels in normal non-tumour cells.^3^–^5^ The overexpression of ATAD2 has been associated with a poor patient outcome to treatment in breast,^6^ lung,^7^ ovarian,^8^ hepatocellular,^9^ endometrial^10^ and gastric^11^ cancers. Functionally, ATAD2 has been shown to act as a coactivator of various transcription factors including the androgen receptor (AR),^4^ and the proto-oncogene MYC,^5^ and studies examining gene silencing of ATAD2, using siRNA or shRNA, report it to have a role in tumour cell proliferation and survival.^3^,^12^ These studies suggest that ATAD2 is a potential target for cancer drug discovery, and small-molecule inhibitors would provide insight into the phenotypic response to the inhibition of ATAD2.

ATAD2 comprises a four helical bundle (α_Z_, α_A_, α_B_, α_C_) and two loops (ZA and BC). The acetyllysine binding pocket, created by helices α_B_, α_C_ and the ZA loop, is polar and shallow compared to several other bromodomains.^13^ The ZA loop of ATAD2, which forms a major part of the binding site is polar, whereas, the binding site in the bromodomain BRD4 is mostly hydrophobic.^14^ The flexibility of the ZA loop coupled to the shallow and polar nature of the binding site resulted in the druggability of the ATAD2 bromodomain being classified as ‘difficult’.^13^,^14^

Compounds **1** and **2a–b** were recently disclosed as relatively potent inhibitors of the bromodomain of ATAD2.^15^–^17^ Compound **1** was not selective for ATAD2 over BRD4. Enhanced ATAD2 potency and selectivity over BRD4 was achieved in this series through introduction of a cyclic sulfone (**2a**) or a difluorocyclohexane moiety (**2b**). These groups form favourable interactions with the sidechains of Arg1007 and Arg1077 in the ATAD2 binding site, and achieve selectivity due to unfavourable interactions with lipophilic Trp81 and Met149 sidechains in BRD4. Compounds **3–5** were reported as ATAD2 inhibitors arising from a fragment screen,^18^ but the low potency of these fragments makes them unsuitable as chemical probes of ATAD2 function. More recently **BAY-850** (**6**) has been reported as a potent ATAD2 inhibitor arising from screening of a DNA-encoded library with an unusual dimer-inducing mode of action, although not BRD4 selectivity data was reported.^19^

This work describes the development of ATAD2 inhibitors employing structure-guided optimization of a fragment hit, with potential for development into selective chemical tools to investigate ATAD2 bromodomain function in biological systems.
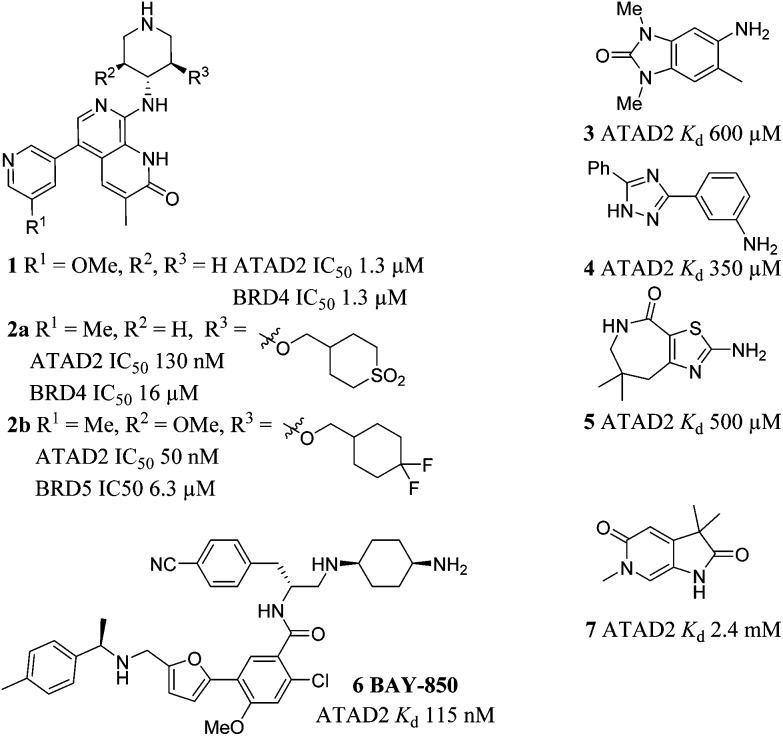


## Synthesis

**Scheme 1:**
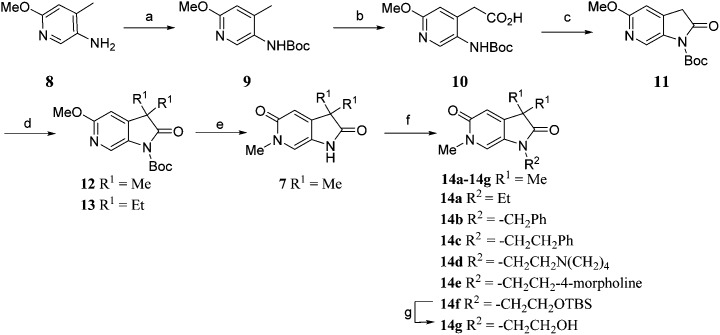
Reagents and conditions: (a) Boc_2_O, THF, Na_2_CO_3_, THF, r.t., 42 h, 95%; (b) (i) *s*-BuLi, THF, –78 °C, 15 min; (ii) CO_2_ (dry ice), –78 °C to r.t., 45 min, 74%; (c) Ac_2_O, tetrabutylammonium acetate, 65 °C, 1 h, 81%; (d) R^1^ = Me: MeI, Cs_2_CO_3_, MeCN, 60 °C, 3 h, 78%; R^1^ = Et: EtI, Cs_2_CO_3_, MeCN, 60 °C, 3 h, 51% (e) MeI, MeCN, 170 °C, μW, 1 h, quant; (f) for compounds **14a–c** (i) NaH, DMF, r.t., 15 min; (ii) ethyl iodide or benzyl bromide or (2-bromoethyl)benzene, r.t., 3 h, 61% (**14a**), 61%; (**14b**), 30% (**14c**); For compounds **14d–f**: Cs_2_CO_3_, DMF, 100 °C, μW, 30 min: 1-(2-chloroethyl)pyrrolidine hydrochloride 40% (**14d**), or 4-(2-chloroethyl)morpholine hydrochloride, 82% (**14e**), or (2-bromoethoxy)(*tert*-butyl)dimethylsilane, 55%; (**14f**); (g) TBAF, THF, r.t., 18 h, 90%.

Screening of a small targeted fragment library identified 1,6-dihydro-2*H*-pyrrolo[2,3-*c*]pyridine-2,5(3*H*)-dione **7** as a promising scaffold for the design of inhibitors of the ATAD2 bromodomain. Synthesis of the bicyclic template started with protection of commercially available 6-methoxy-4-methylpyridin-3-amine **8** as its *tert*-butyl carbamate to give **9** (Scheme 1). Deprotonation followed by quenching with carbon dioxide gave **10**. Acetic anhydride mediated ring closure in the presence of catalytic tetra-*N*-butylammonium acetate followed by bis-alkylation with iodomethane or iodoethane gave **12** and **13**, respectively. Deprotection and *N*-alkylpyridone formation was achieved in a single step by heating **12** with iodomethane to give compound **7**. Targets varying the *N*^1^ substituent (**14a–f**) were synthesised by alkylation of **7**. **14f** was deprotected using TBAF to provide **14g**.

**Scheme 2:**
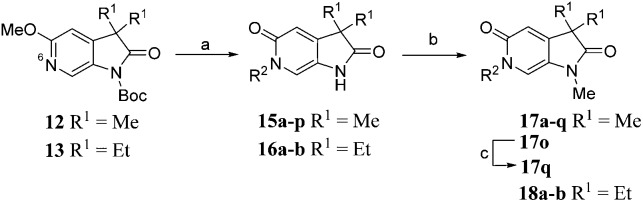
Reagents and conditions: (a) RX, MeCN, 170 °C, μW, 45 min, 22–93%; (b) MeI, Cs_2_CO_3_, DMF, 100 °C, μW, 30 min, 74–94%; (c) NaOH, EtOH, H_2_O, 100 °C, 23 h, 32%; Structures of **17a–q** and **18a–b** are given in Tables 2 and 3.

*N* ^6^-Alkylated target compounds were synthesised from **12** or **13** under microwave irradiation using an array of alkyl halides to give **15a–p** and **16a–b**, respectively. Subsequent *N*^1^-methylation gave compounds **17a–q** and **18a–b** (Scheme 2).

**Scheme 3:**
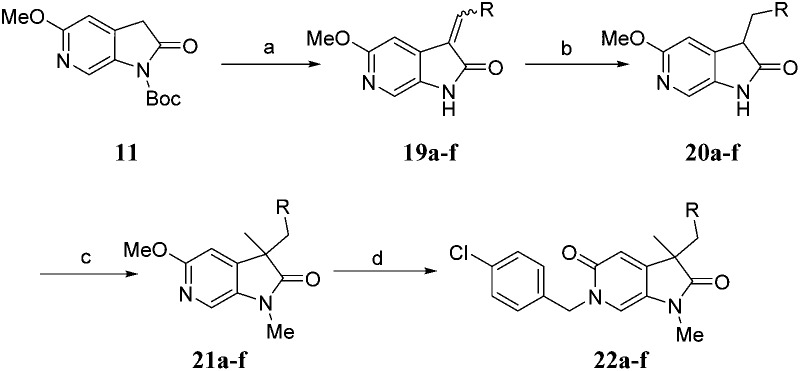
Reagents and conditions: (a) RCHO, piperidine, THF, 100 °C, 30 min, 50–88%; (b) H_2_, 10% Pd/C, THF, MeOH, r.t., 2 h; (c) MeI, Cs_2_CO_3_, DMF, 50 °C, 1.5 h, 55–56% over 2 steps; (d) 4-chlorobenzyl bromide, MeCN, 170 °C μW, 45 min, 50–64%. Structures of **22a–f** are given in Table 4.

Diversification of the *C*^3^-position could be achieved using Knoevenagel condensation of aldehydes with **11** to provide **19a–j** in high yield (Scheme 3). Subsequent alkene reduction, followed by methylation provided **21a–f**. Treatment of methoxypyridines **21a–f** with benzyl bromides at high temperature provided the target pyridones (**22a–f**) in moderate yields.

## Results and discussion

**Fig. 1:**
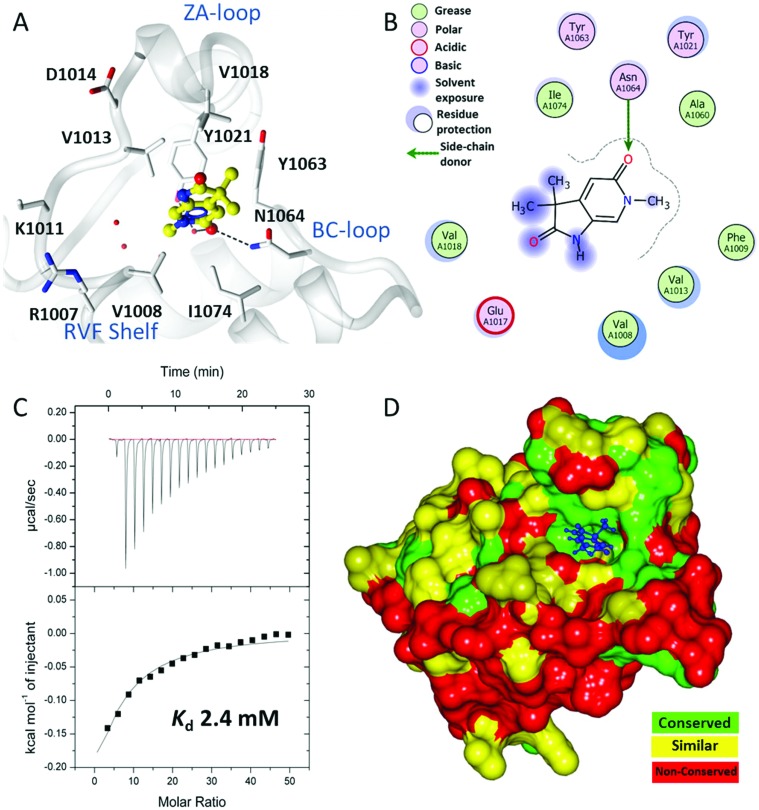
Binding of initial fragment hit **7**. (A) Binding mode of fragment hit **7** (yellow ball and stick) bound to ATAD2 (white). ATAD2 active site residues shown in cylinder and conserved water molecules shown as red spheres. Potential hydrogen bonding interactions shown in black dash. (B) 2D Interaction map of **7** with the active site residues of ATAD2 represented by Lidia within Coot. (C) Isothermal titration calorimetry of **7** binding with saturation to ATAD2. (D) ATAD2 represented in solid surface and coloured through conservation of sequence identity between ATAD2 and the first bromodomain of BRD4.

A crystallographic screen of a small targeted library identified pyrrolidinopyridone **7** as a fragment which bound in the *N*-acetyllysine histone binding site of the bromodomain of ATAD2 (Fig. 1A and B). Isothermal titration calorimetry (ITC) was used to infer the binding affinity of **7** for the bromodomain of ATAD2, with an apparent *K*_d_ of 2.4 mM (Fig. 1C). The pyridone carbonyl forms a hydrogen bond with the carboxamide sidechain of Asn_1064_, and another through a water-mediated hydrogen bond to Tyr_1021_, with the *N*^6^-methyl group projecting towards a pocket containing four conserved water molecules. This interaction network mimics the acetylated lysine of the histone, and is consistent with small hydrophobic groups that occupy the equivalent pocket in other bromodomain family ligands such as the BRD4 inhibitors JQ1^20^ and I-BET762.^21^ Selectivity over other bromodomain family members is important in the development of a tool compound to investigate the role of the ATAD2 bromodomain in biological systems.^22^ Minimizing the BET activity of an ATAD2 chemical probe is particularly necessary due to the known effects of BET inhibitors. Thus BRD4 was selected as representative BET family member to assess compound selectivity. The binding orientation of **7** in ATAD2 was designated as binding mode 1 for this template. The residues that line the acetylated lysine binding pocket of BRD4 and ATAD2 are highly conserved (Fig. 1D), with greater divergence in the more solvent exposed residues including the shelf region of the protein. Achieving selectivity for ATAD2 over BRD4 would thus require identification of fragments that extend away from the conserved core and towards the less conserved areas.

**Fig. 2:**
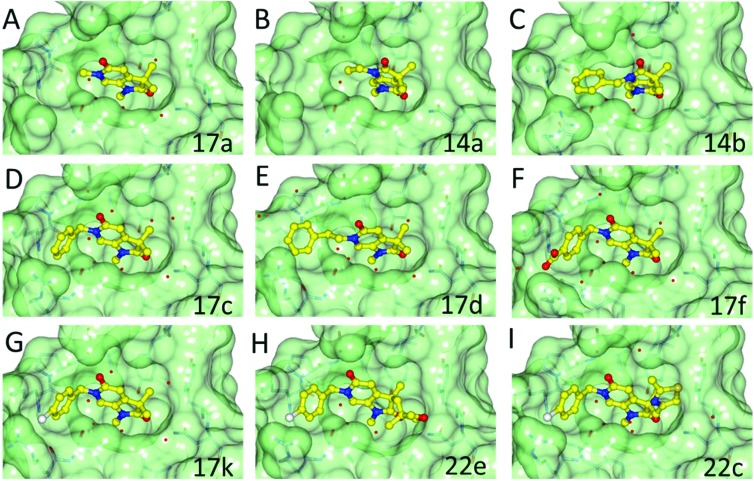
Overlay of the crystal structures of fragment **17a**, **14a**, **14b**, **17c**, **17d**, **17f**, **17k**, **22e** and **22c** bound to ATAD2. Compounds represented in yellow as ball and stick. Binding pocket of ATAD2 shown in transparent surface, with neighbouring residues of compound shown in stick representation. Conserved waters are represented by red spheres.

Methylation of the *N*^1^ position (**17a**) resulted in a change in binding mode (Fig. 2A), whereby the fragment core rotates and the pyrrolidinone carbonyl now forms a hydrogen bond with the carboxamide sidechain of Asn_1064_, and a second through a water-mediated hydrogen bond to Tyr_1021_. In this binding mode the *N*^1^-methyl group projects towards the conserved water molecules. Rotation of the pyrrolidinone core allows the pyridone carbonyl to interact through a water molecule with the backbone NH of Asp_1014_ on the ZA loop. One of the *C*^3^-geminal methyl groups occupies a small hydrophobic pocket formed between the sidechains of ZA-loop residues Tyr_1021_, Val_1018_ and the Tyr_1063_ residue that reside next to the conserved asparagine. Additional hydrophobic interactions are formed with the base of the binding pocket, where Val_1008_ and Ile_1074_ interact with the second *C*^3^-geminal methyl group and pyrrolidinone core, respectively. This binding orientation was designated as binding mode 2. Intriguingly, when the *N*^1^ substituent was further elaborated to an ethyl group (**14a**: Fig. 2B) the compound reverted to binding mode 1, suggesting the conserved water-rich pocket was unable to accommodate the larger ethyl group.

Fragments **7**, **17a**, and **14a** bound very weakly and were unable to displace the acetylated histone ligand sufficiently in a homogeneous time-resolved fluorescence (HTRF) assay to enable a *K*_d_ to be determined, although some inhibition was observed at the highest concentration assayed (1000 μM; Table 1). Surface plasmon resonance (SPR) was used to assess the direct binding of the fragments and provide *K*_d_ values for binding to both ATAD2 and BRD4. SPR can yield artefactual data where the concentration of a small-molecular analyte exceeds ∼250 μM, and accordingly, this was the highest concentration used in the assays. Where the *K*_d_ exceeded this limit, projected *K*_d_s were determined by extrapolation, using the observed response units for compound binding (RU) relative to the molecular weight of a known control compound.^23^ Compounds **7** and **17a** bound too weakly to ATAD2 to allow *K*_d_ values to be calculated. However, **17a** did show detectable binding to BRD4. Compound **14a** showed a remarkable increase in affinity towards BRD4, reporting a *K*_d_ of 12.7 μM and a binding constant 100-fold weaker towards ATAD2 (Table 1).

**Table 1:** ATAD2 inhibition data for compounds **7**, **14a–g** and **17a**

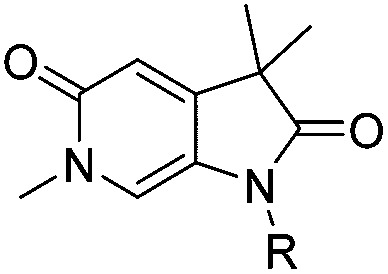					
ID	R	ATAD2 IC_50_ ^*a*^ (μM)	ATAD2 LE	ATAD2 *K*_d_ ^*b*^ (μM)	BRD4 *K*_d_ ^*b*^ (μM)
**7**	H	>1000	N/A	>2000	>2000
				2400^*c*^	
**17a**	Me	>1000	N/A	>2000	1500
**14a**	Et	>1000	N/A	1300	12.7
**14b**	Bn	810 ± 57	0.21	>2000	1050
**14c**	–CH_2_CH_2_Ph	868 ± 33	0.19	1400	800
**14d**	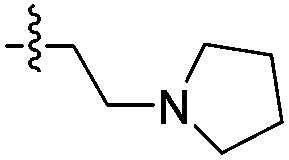	>1000	N/A	>2000	1700
**14e**	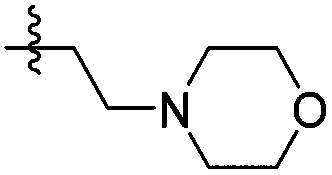	>1000	N/A	>2000	>2000
**14g**	–CH_2_CH_2_OH	>1000	N/A	>2000	>2000

^*a*^HTRF format assay.

^*b*^SPR format assay.

^*c*^Isothermal titration calorimetry. Ligand efficiency (LE) = 1.4(–log IC_50_)/*N*, where *N* is number of non-hydrogen atoms.

The two binding modes offered different vectors to explore structure activity relationships (SARs) of substituents directed towards the RVF shelf and the ZA loop. Thus a set of *N*^1^-alkylated pyrrolidinopyridones was prepared to attempt to access this region while maintaining binding mode 1 (Table 1). Lipophilic benzyl (**14b**) and phenethyl (**14c**) substituents exhibited sub-millimolar HTRF IC_50_ values, and showed the first detectable signs of direct binding to both ATAD2 and BRD4 when analysed by SPR. Co-crystallisation confirmed that **14b** retained binding mode 1 (Fig. 2C). Hydrophilic substituents (**14d–g**) gave no measurable ATAD2 inhibition or binding in HTRF or SPR format experiments.

To attempt to exploit binding mode 2, a set of *N*^6^-alkylated pyrrolidinopyridones were also prepared (Table 2) with *N*^6^-benzyl (**17c**) and *N*^6^-phenethyl (**17d**) analogues giving sub-millimolar ATAD2 IC_50_ values. As expected, **17c** and **17d** retained binding mode 2 (Fig. 2D and E), with the benzyl group sitting above the RVF shelf, forming a hydrophobic interaction with the sidechain of Lys_1011_. Pairwise comparison with *N*^1^-*des*-methyl analogue **15c** confirmed the importance of a methyl group in the conserved water pocket. **17c** and **17d** bound more tightly to BRD4 than to ATAD2 in the SPR experiments.

**Table 2:** ATAD2 inhibition, SPR and BRD4 binding data for compounds **15c** and **17b–d**

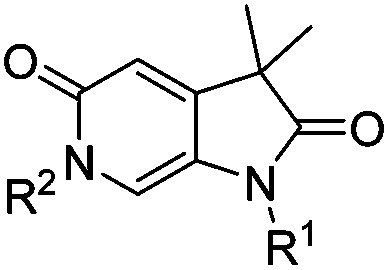						
ID	R^1^	R^2^	ATAD2 IC_50_ ^*a*^ (μM)	ATAD2 LE	ATAD2 *K*_d_ ^*b*^ (μM)	BRD4 *K*_d_ ^*b*^ (μM)
**17b**	H	Pr	>1000	N/A	>2000	1000
**15c**	H	Bn	>1000	N/A	2000	1400
**17c**	Me	Bn	681 ± 120	0.21	700	101
**17d**	Me	–CH_2_CH_2_Ph	741 ± 74	0.20	1000	161

^*a*^HTRF format assay.

^*b*^SPR format assay. Ligand efficiency (LE) = 1.4(–log IC_50_)/*N*, where *N* is number of non-hydrogen atoms.

**Table 3:** ATAD2 inhibition, SPR and BRD4 binding data for compounds **17e–q**

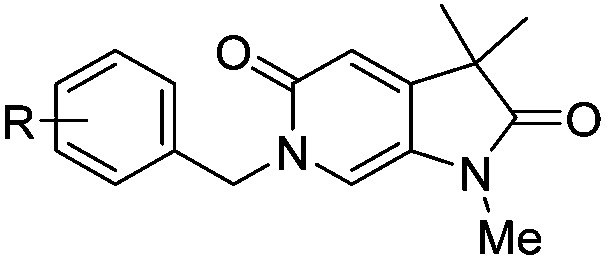					
ID	R	ATAD2 IC_50_^*a*^ (μM)	ATAD2 LE	ATAD2 *K*_d_^*b*^ (μM)	BRD4 *K*_d_^*b*^ (μM)
**17e**	4-CO_2_Me	651 ± 1	0.18	900	141
**17f**	4-CO_2_H	533 ± 40	0.19	600	1500
**17g**	4-CN	562 ± 67	0.20	900	142
**17h**	4-CONH_2_	680 ± 66	0.18	700	300
**17i**	4-SO_2_Me	359 ± 37	0.19	600	200
**17j**	4-Cl	190 ± 63	0.24	200	40
**17k**	4-Br	236 ± 36	0.23	138	48
**17l**	4-Me	274 ± 75	0.23	400	58
**17m**	4-CF_3_	179 ± 43	0.21	400	124
**17n**	3,4-DiCl	299 ± 28	0.21	159	65
**17o**	2-CN	>1000	N/A	>2000	300
**17p**	3-CN	668 ± 28	0.19	800	184
**17q**	2-CO_2_H	>1000	N/A	>2000	300

^*a*^HTRF format assay.

^*b*^SPR format assay. Ligand efficiency (LE) = 1.4(–log IC_50_)/*N*, where *N* is number of non-hydrogen atoms.

With the aim of improving binding affinity towards ATAD2 the sidechain of Arg_1007_ was targeted through substitution of the 4-position of the *N*^6^-benzyl group. Compound **17f** showed similar ATAD2 affinity to **17c**, and the co-crystal structure with ATAD2 (Fig. 2F) confirmed that the carboxylate did not form the projected charge–charge interaction with Arg_1007_. Other analogues bearing functionality with the potential to form hydrogen bonds with Arg_1007_ gave no further improvement in inhibition (**17g–i**). Lipophilic substituents at the 4-position of the benzyl group (**17j–m**, Fig. 2G) provided the most potent benzylic analogues, but continued to be selective for BRD4. Addition of a second chloro substituent at the 3-position (**17n**) resulted in a modest drop in potency. *N*^6^-Benzyl analogues with substituents at the 2- and 3-positions of the benzylic ring (**17o–q**) were prepared to attempt to introduce further interactions with the ZA loop, or to favourably influence the conformation of the ligand, but no further improvement in ATAD2 affinity was observed.

Attention turned to exploring substitution at the *C*^3^-geminal dimethyl group. The *pro-S* methyl group occupies a small lipophilic pocket in the ligand binding site of ATAD2, whereas the *pro-R* methyl provides opportunities for fragment growth. It was envisioned that larger substituents at the *pro-R* position may occupy the cleft between the ZA and BC loops where the substrate acetylated lysine sidechain enters the active site, and may also provide improved selectivity over the more sterically demanding BRD4. Synthetic chemistry to access chiral *C*^3^ analogues that retained one methyl but differed in the second *C*^3^ substituent proved challenging, with mono-alkylation strategies found to be low yielding. Knoevenagel condensation was successful when the aldehyde coupling partner did not contain an enolisable proton, allowing the synthesis of **22a–f** (Table 4) as racemic mixtures. Symmetrical *C*^3^-diethyl analogues **18a** and **18b** were also prepared.

**Table 4:** ATAD2 inhibition, SPR and BRD4 binding data for compounds **22a–f** and **18a–b**

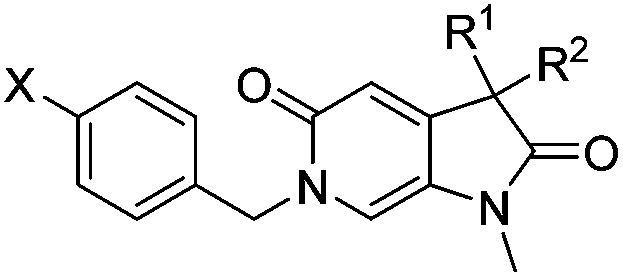							
ID	R^1^	R^2^	X	ATAD2 IC_50_^*a*^ (μM)	ATAD2 LE	ATAD2 *K*_d_^*b*^ (μM)	BRD4 *K*_d_^*b*^ (μM)
**22a**	Me	Bn	Cl	>1000	N/A	500	>2000
**22b**	Me	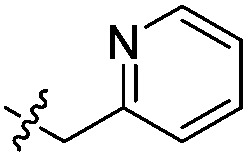	Cl	>1000	N/A	300	1700
**22c**	Me	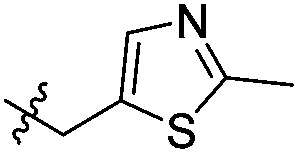	Cl	352	0.17	110	1500
**22d**	Me	CH_2_C(CH_3_)_3_	Cl	489 ± 18	0.18	125	>2000
**22e**	Me	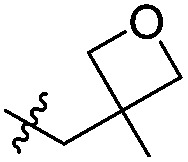	Cl	214	0.19	500	>2000
**22f**	Me	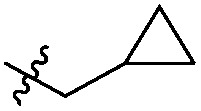	Cl	304 ± 83	0.20	113	800
**18a**	Et	Et	Cl	163 ± 23	0.22	107	1000
**18b**	Et	Et	CF_3_	163 ± 37	0.20	300	>2000

^*a*^HTRF format assay.

^*b*^SPR format assay. Ligand efficiency (LE) = 1.4(–log IC_50_)/*N*, where *N* is number of non-hydrogen atoms.

**Fig. 3:**
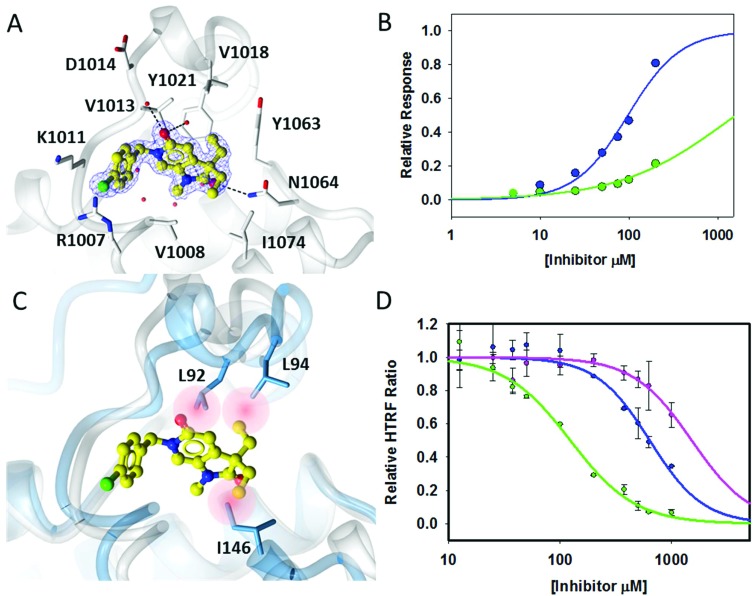
Binding interactions of **18a**. (A) Crystal structure of **18a** (yellow ball and stick) within the histone binding site of ATAD2 shown in white ribbon, active site residues shown in cylinder and conserved water molecules shown in red sphere. Key potential hydrogen bonding interactions shown in black dash. (B) Direct binding data using SPR of **18a** with ATAD2 (blue) and BRD4 (green). (C) Superimposition of **18a** bound to ATAD2 with BRD4 showing potential steric clashes of **18a** within the binding site of BRD4 (red). (D) HTRF data of improved potency within the series **17a** pink, **17c** blue, **18a** green.

The crystal structure of **18a** bound to ATAD2 (Fig. 3A) showed that the diethyl analogues delve further into the hydrophobic pockets formed within the ZA-loop residues Tyr_1021_, Val_1018_ and with Ile_1074_ in the base of the binding site. Gratifyingly, these analogues resulted in a significant improvement in selectivity over BRD4 while retaining ATAD2 binding and inhibition (Table 4) (Fig. 3B).

Superimposition of the crystal structure of **18a** with the first bromodomain of BRD4 (PDB entry ; 2OSS) indicates that the selectivity for ATAD2 over BRD4 may be due to a steric clash of one of the ethyl groups with Leu_94_ and the other with Ile_146_ in the BRD4 structure (Fig. 3C).

## Conclusions

Pyrrolidinopyridone **7** was identified through crystallographic fragment screening as a novel ligand for the ATAD2 bromodomain. Assessment in HTRF, ITC and SPR assays confirmed that this was a low affinity fragment. Structure-guided fragment growing at the *N*^1^, *N*^6^ and *C*^3^ substituents led to the identification of two binding modes for this template in ATAD2. For binding mode 1, methylation of *N*^1^, and incorporation of substituted benzyl groups at *N*^6^ led to compounds with ATAD2 binding affinities below 200 μM (Fig. 3D), but these compounds bound more tightly to BRD4 than to ATAD2. Increasing steric demand at the *C*^3^ position led to compounds **18a** and **18b**, retaining ATAD2 binding affinity and activity in the HTRF biochemical assay. These *C*^3^-substituents conferred selectivity over BRD4 through the introduction of unfavorable interactions with lipophilic amino acid sidechains in the more sterically demanding BRD4 *N*-acetyllysine binding site.

Overall, we have demonstrated that structure-based optimisation of ATAD2 fragments was able to bring millimolar hits into a micromolar potency range. Although inhibition of ATAD2 in the pyrrolidinopyridone series reached a plateau at 100–200 μM, we have succeeded in identifying interactions that can enhance potency while introducing selectivity *versus* BRD4. Application of these lessons might contribute to the development of improved chemical probes of ATAD2's role and structure–function relationship, if applied to fragments with superior initial ligand efficiency.

## Author contributions

Duncan C. Miller – Experimental design and interpretation, data analysis, synthesis and characterization of compounds and manuscript preparation.

Mathew Martin – Protein expression, crystallisation and structure determination, assay development and manuscript preparation. ^†^Santosh Adhikari – Synthesis and characterization of compounds.

Alfie Brennan – Synthesis and characterization of compounds.

Jane A. Endicott – Project conception, experimental design and interpretation.

Bernard T. Golding – Experimental design and interpretation, data analysis.

Ian R. Hardcastle – Experimental design and interpretation, data analysis.

Amy Heptinstall – Synthesis and characterization of compounds.

Stephen Hobson – Synthesis and characterization of compounds.

Claire Jennings – Protein expression, crystallisation and structure determination.

Lauren Molyneux – Synthesis and characterization of compounds.

Yvonne Ng – Assay development.

Stephen R. Wedge – Project conception, experimental design and interpretation.

Martin E. M. Noble – Project conception, experimental design and interpretation, data analysis and manuscript preparation.

Céline Cano – Project conception, experimental design and interpretation, data analysis and manuscript preparation (submitting author).

## Conflicts of interest

There are no conflicts of interest to declare.

## Supplementary Material

Supplementary information

Supplementary information
