# Supplementary material for: Identification of a novel ligand for the ATAD2 bromodomain with selectivity over BRD4 through a fragment growing approach
Source: Org Biomol Chem. 2018 Feb 15;16(11):1843–50. doi: 10.1039/c8ob00099a (PMC6102691; doi:10.1039/c8ob00099a)
Supplement: Supplementary file 1 [file OB-016-C8OB00099A-s001.pdf]

Electronic Supplementary Material (ESI) for Organic & Biomolecular Chemistry.
This journal is © The Royal Society of Chemistry 2018

Supplementary Information

For

Identification of a Novel Ligand for the ATAD2 Bromodomain with Selectivity Over BRD4 Through a Fragment Growing Approach.

Duncan C. Miller, Mathew P. Martin, Santosh Adhikari, Alfie Brennan, Jane a. Endicott, Bernard T. Golding, Ian R. Hardcastle, Amy Heptinstall, Stephen Hobson, Claire Jennings, Lauren Molyneux, Yvonne Ng, Stephen R. Wedge, Martin E. M. Noble, Celine Cano

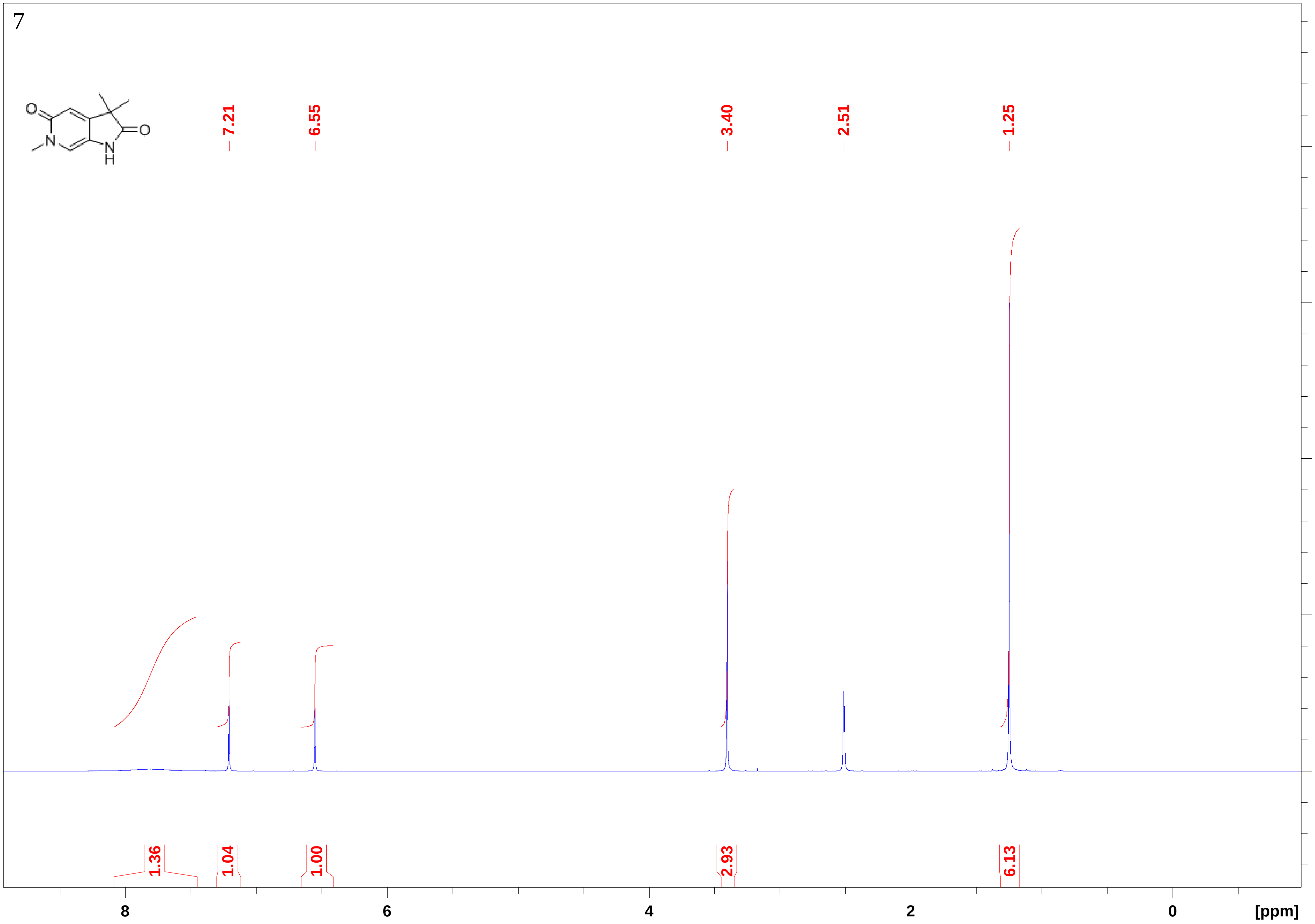
7
7.21
6.55
3.40
2.51
1.25
1.36
1.04
1.00
2.93
6.13
8
6
4
2
0
[ppm]
[rel]
20
15
10
5
0

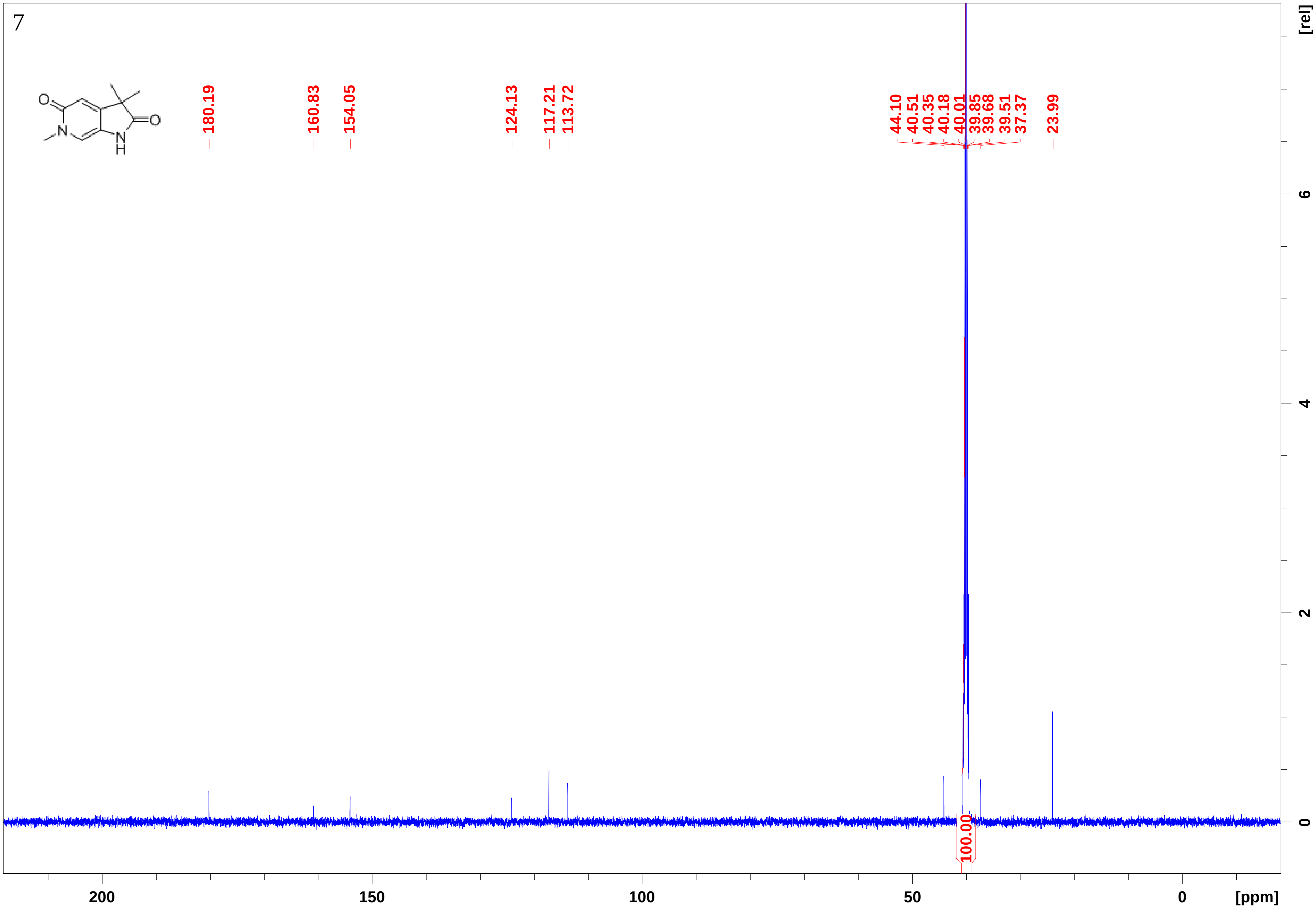
7
180.19
160.83
154.05
124.13
117.21
113.72
44.10
40.51
40.35
40.18
40.01
39.85
39.68
39.51
37.37
23.99
100.00
[rel]
6
4
2
0
200
150
100
50
0
[ppm]

13

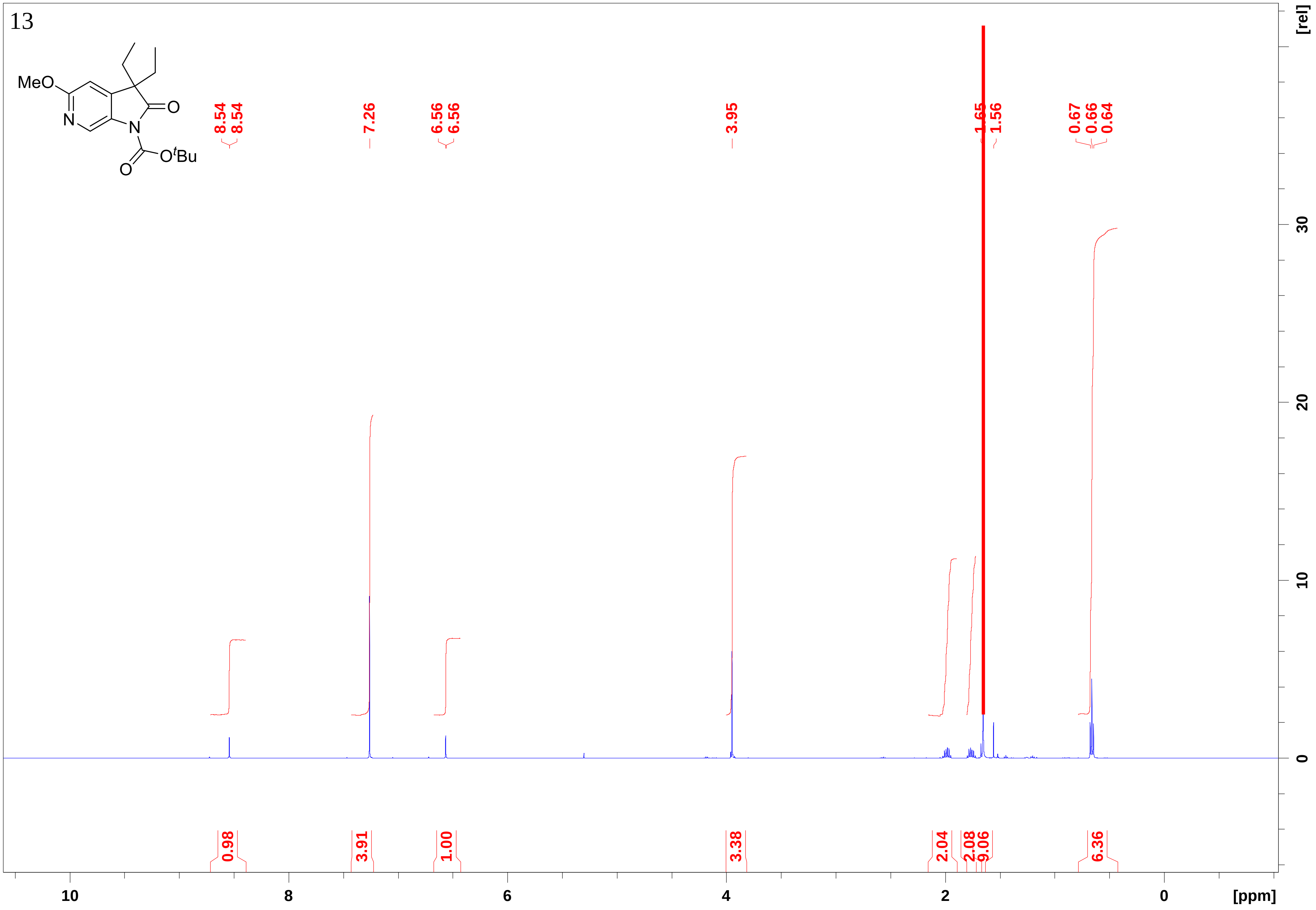

MeO
N
O
N
O
O^{t}Bu
8.54
8.54
7.26
6.56
6.56
3.95
1.65
1.56
0.67
0.66
0.64
0.98
3.91
1.00
3.38
2.04
2.08
9.06
6.36
10
8
6
4
2
0
[ppm]
[rel]
30
20
10
0

14a

CD3OD

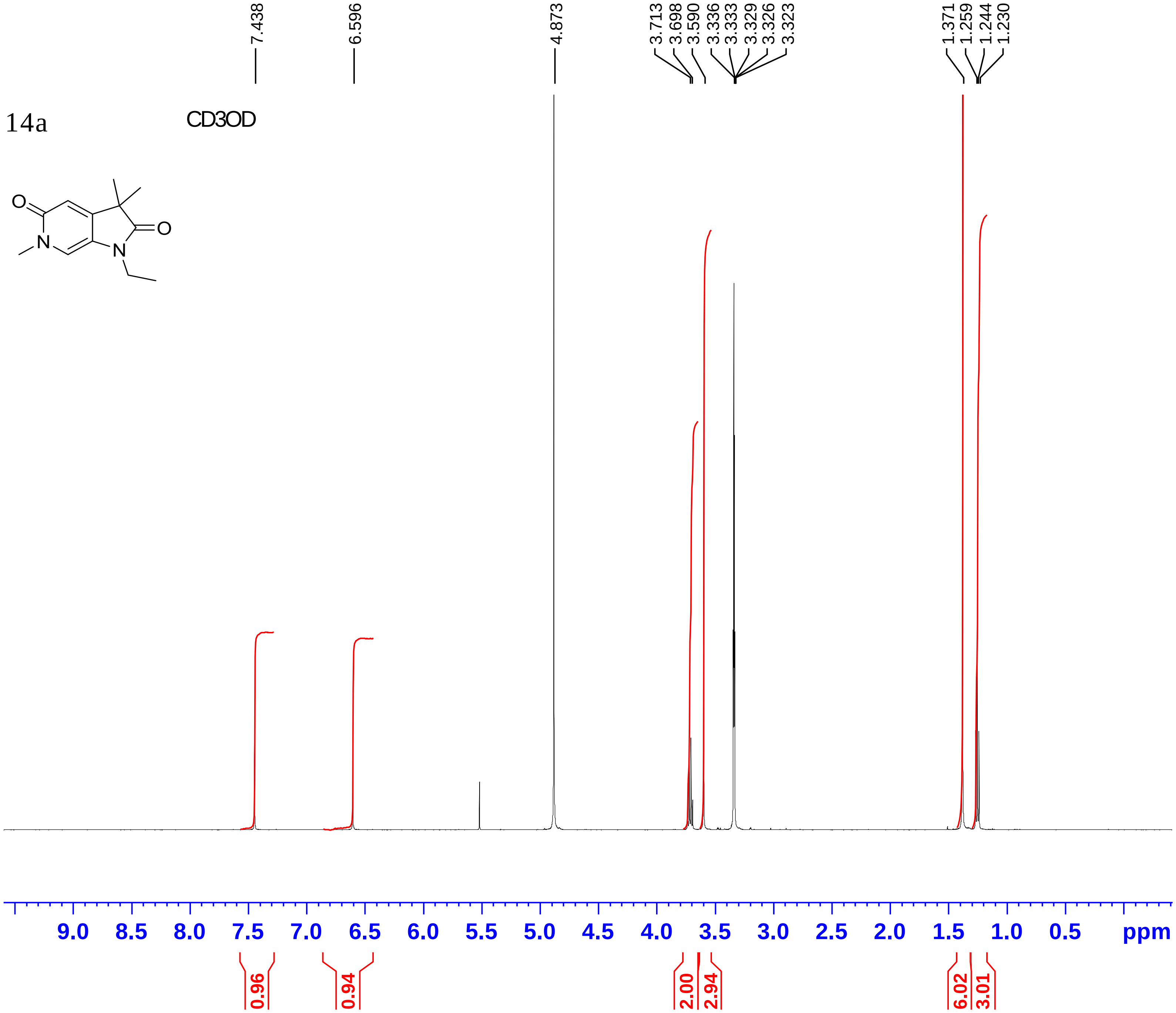

Current Data Parameters

NAME	DCM-494-29-A CD3OD
EXPNO	10
PROCNO	1

F2 - Acquisition Parameters

Date_	20140910
Time	13.24
INSTRUM	spect
PROBHD	5 mm PABBO BB-
PULPROG	zg30
TD	65536
SOLVENT	MeOD
NS	16
DS	0
SWH	10330.578 Hz
FIDRES	0.157632 Hz
AQ	3.1719425 sec
RG	575
DW	48.400 usec
DE	11.99 usec
TE	297.9 K
D1	1.00000000 sec
TD0	1

======== CHANNEL f1 ========

SFO1	500.3030896 MHz
NUC1	1H
P1	16.25 usec
PLW1	18.33600044 W

F2 - Processing parameters

SI	65536
SF	500.2999951 MHz
WDW	EM
SSB	0
LB	0.30 Hz
GB	0
PC	1.00

14a

CD3OD

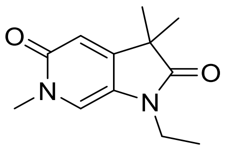

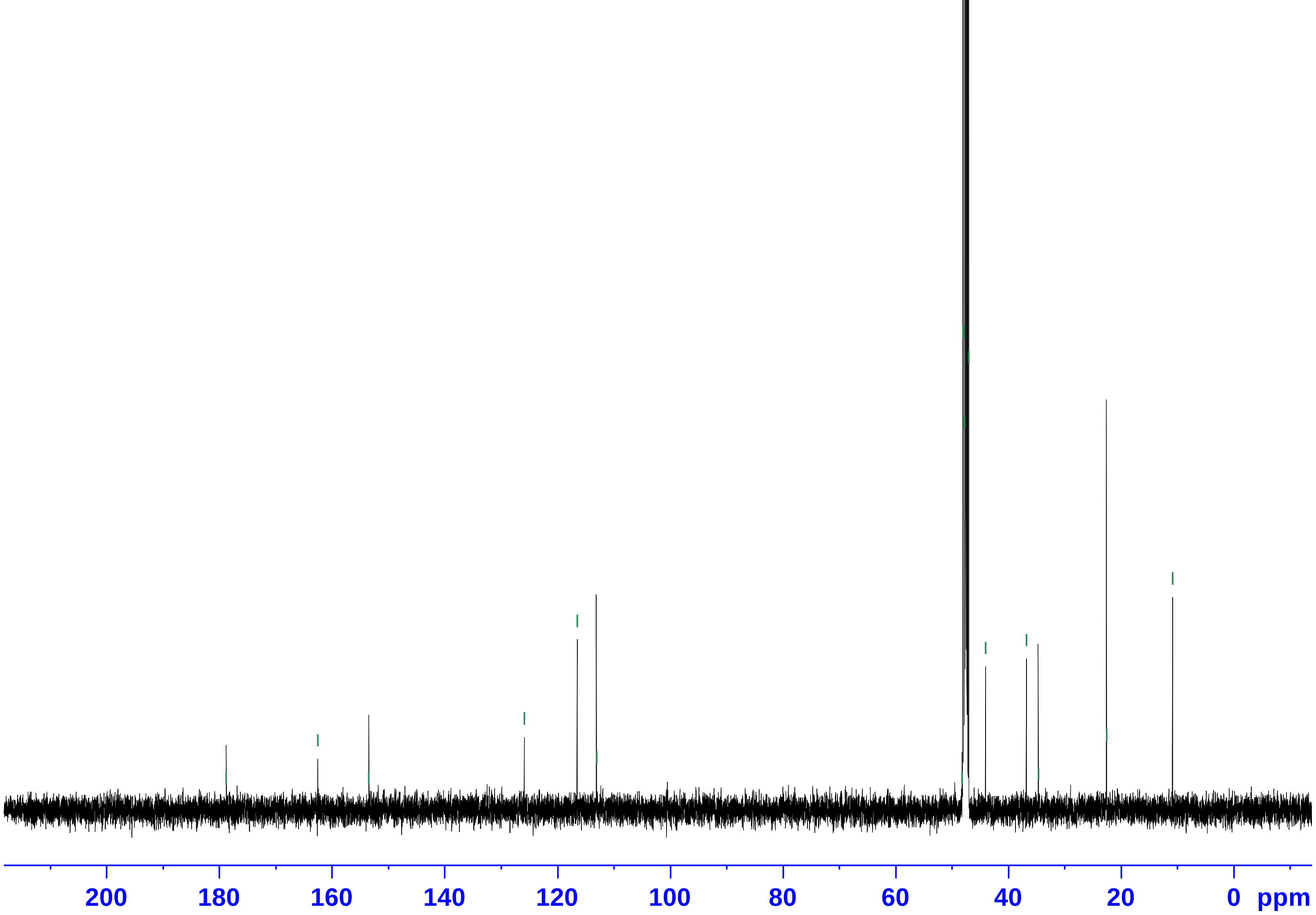

Current Data Parameters
NAME DCM-494-29-A CD3OD
EXPNO 11
PROCNO 1

F2 - Acquisition Parameters
Date_ 20140911
Time 3.13
INSTRUM spect
PROBHD 5 mm PABBO BB-
PULPROG zgpg30
TD 65536
SOLVENT MeOD
NS 2048
DS 2
SWH 29761.904 Hz
FIDRES 0.454131 Hz
AQ 1.1010048 sec
RG 456
DW 16.800 usec
DE 7.68 usec
TE 298.6 K
D1 2.00000000 sec
D11 0.03000000 sec
TD0 1

======== CHANNEL f1 ========

SFO1 125.8131151 MHz
NUC1 13C
P1 9.75 usec
PLW1 82.38999939 W

======== CHANNEL f2 ========

SFO2 500.3020012 MHz
NUC2 1H
CPDPRG[2 waltz16
PCPD2 80.00 usec
PLW2 18.33600044 W
PLW12 0.75654000 W
PLW13 0.48418999 W

F2 - Processing parameters
SI 65536
SF 125.8005350 MHz
WDW EM
SSB 0
LB 1.00 Hz
GB 0
PC 1.40

14b

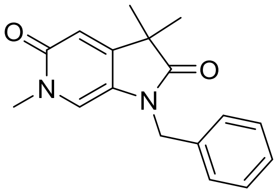

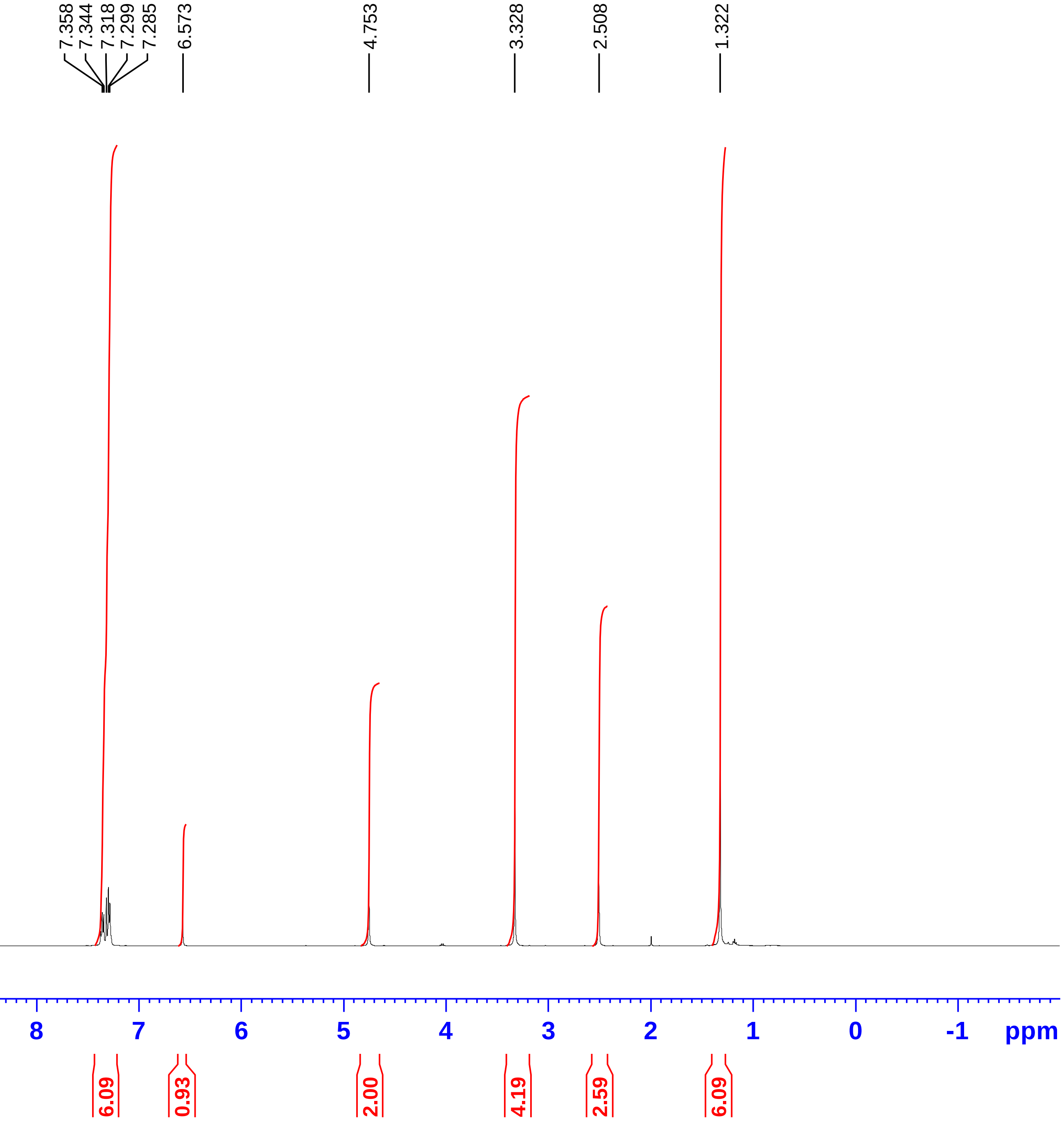

Current Data Parameters

NAME	SA-473-192
EXPNO	20
PROCNO	1

F2 - Acquisition Parameters

Date_	20141128
Time	10.03
INSTRUM	spect
PROBHD	5 mm PABBO BB-
PULPROG	zg30
TD	65536
SOLVENT	DMSO
NS	16
DS	0
SWH	10330.578 Hz
FIDRES	0.157632 Hz
AQ	3.1719425 sec
RG	406
DW	48.400 usec
DE	11.99 usec
TE	298.0 K
D1	1.00000000 sec
TD0	1

======== CHANNEL f1 ========

SFO1	500.3030896 MHz
NUC1	1H
P1	16.25 usec
PLW1	18.33600044 W

F2 - Processing parameters

SI	65536
SF	500.3000000 MHz
WDW	EM
SSB	0
LB	0.30 Hz
GB	0
PC	1.00

14b

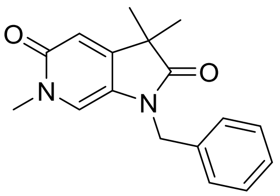

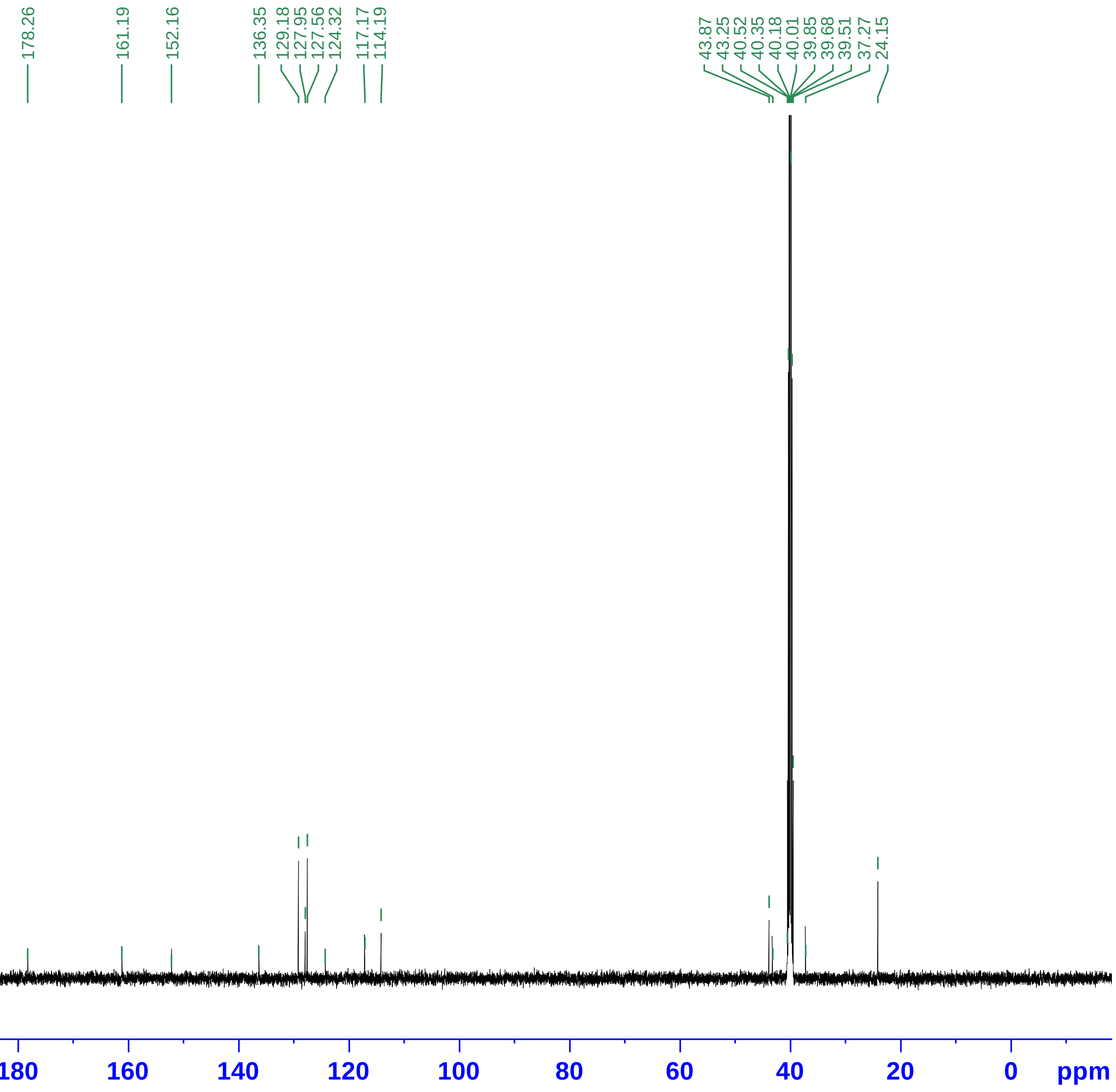

Current Data Parameters
NAME SA-473-192
EXPNO 21
PROCNO 1

F2 - Acquisition Parameters
Date_ 20141128
Time 10.25
INSTRUM spect
PROBHD 5 mm PABBO BB-
PULPROG zgpg30
TD 65536
SOLVENT DMSO
NS 256
DS 2
SWH 29761.904 Hz
FIDRES 0.454131 Hz
AQ 1.1010048 sec
RG 456
DW 16.800 usec
DE 7.68 usec
TE 298.0 K
D1 2.00000000 sec
D11 0.03000000 sec
TD0 1

======== CHANNEL f1 ========

SFO1 125.8131151 MHz
NUC1 13C
P1 9.75 usec
PLW1 82.38999939 W

======== CHANNEL f2 ========
SFO2 500.3020012 MHz
NUC2 1H
CPDPRG[2 waltz16
PCPD2 80.00 usec
PLW2 18.33600044 W
PLW12 0.75654000 W
PLW13 0.48418999 W

F2 - Processing Parameters
SI 65536
SF 125.8005350 MHz
WDW EM
SSB 0
LB 1.00 Hz
GB 0
PC 1.40

14c

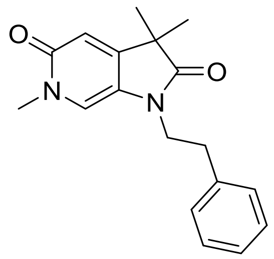

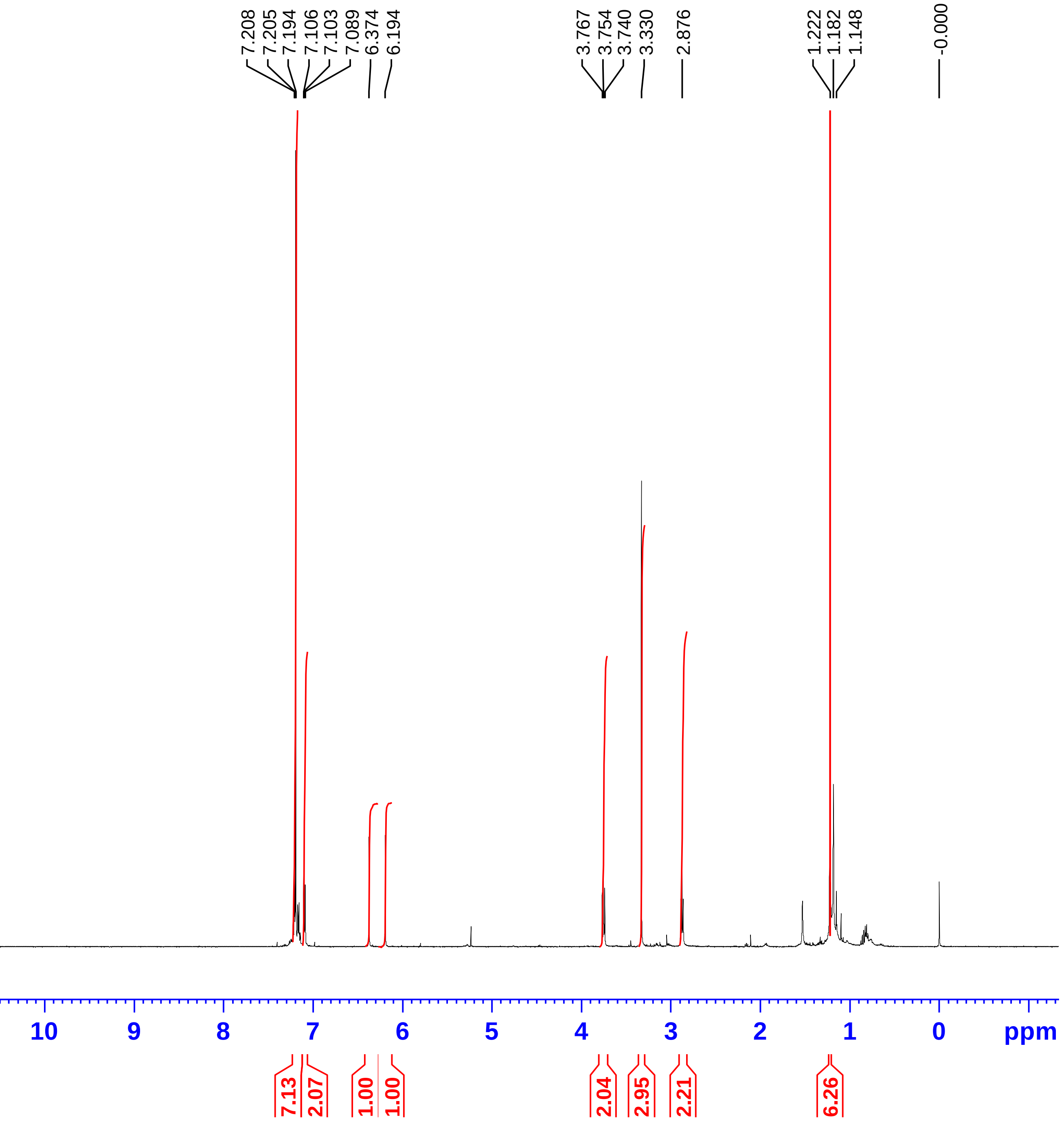

Current Data Parameters

NAME	SA-473-196
EXPNO	30
PROCNO	1

F2 - Acquisition Parameters

Date_	20141215
Time	14.15
INSTRUM	spect
PROBHD	5 mm PABBO BB-
PULPROG	zg30
TD	65536
SOLVENT	CDCl3
NS	16
DS	0
SWH	10330.578 Hz
FIDRES	0.157632 Hz
AQ	3.1719425 sec
RG	645
DW	48.400 usec
DE	11.99 usec
TE	290.9 K
D1	1.00000000 sec
TD0	1

======== CHANNEL f1 ========

SFO1	500.3030896 MHz
NUC1	1H
P1	16.25 usec
PLW1	18.33600044 W

F2 - Processing parameters SI 65536

SF	500.3000446 MHz
WDW	EM
SSB	0
LB	0.30 Hz
GB	0
PC	1.00

14c

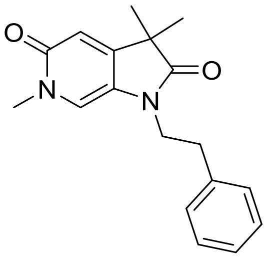

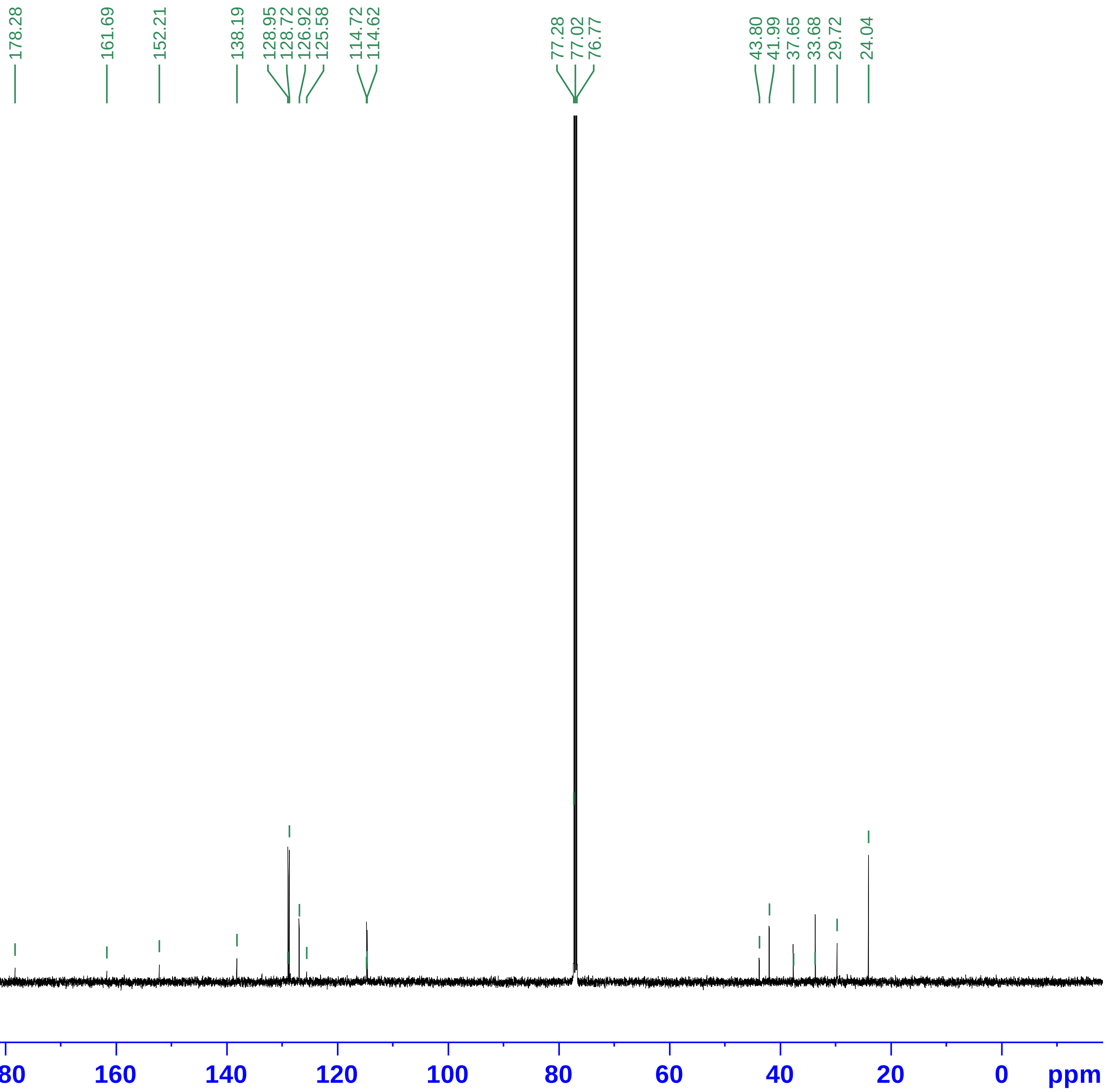

BRUKER

Newcastle University

CANCER RESEARCH UK

Current Data Parameters
NAME SA-473-196
EXPNO 31
PROCNO 1

F2 - Acquisition Parameters
Date_ 20141217
Time 21.14
INSTRUM spect
PROBHD 5 mm PABBO BB-
PULPROG zgpg
TD 26782
SOLVENT CDCl3
NS 2048
DS 2
SWH 29761.904 Hz
FIDRES 1.111265 Hz
AQ 0.4499376 sec
RG 575
DW 16.800 usec
DE 7.72 usec
TE 296.1 K
D1 5.00000000 sec
D11 0.03000000 sec
TD0 1

======== CHANNEL f1 ========
SFO1 125.8131151 MHz
NUC1 13C
P1 9.75 usec
PLW1 82.38999939 W

======== CHANNEL f2 ========
SFO2 500.3020012 MHz
NUC2 1H
CPDPRG[2 waltz16
PCPD2 80.00 usec
PLW2 18.33600044 W
PLW12 0.75654000 W
PLW13 0.48418999 W

F2 - Processing parameters
SI 65536
SF 125.8005350 MHz
WDW EM
SSB 0
LB 1.00 Hz
GB 0
PC 1.40

14d

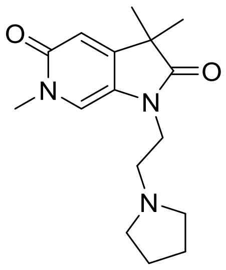

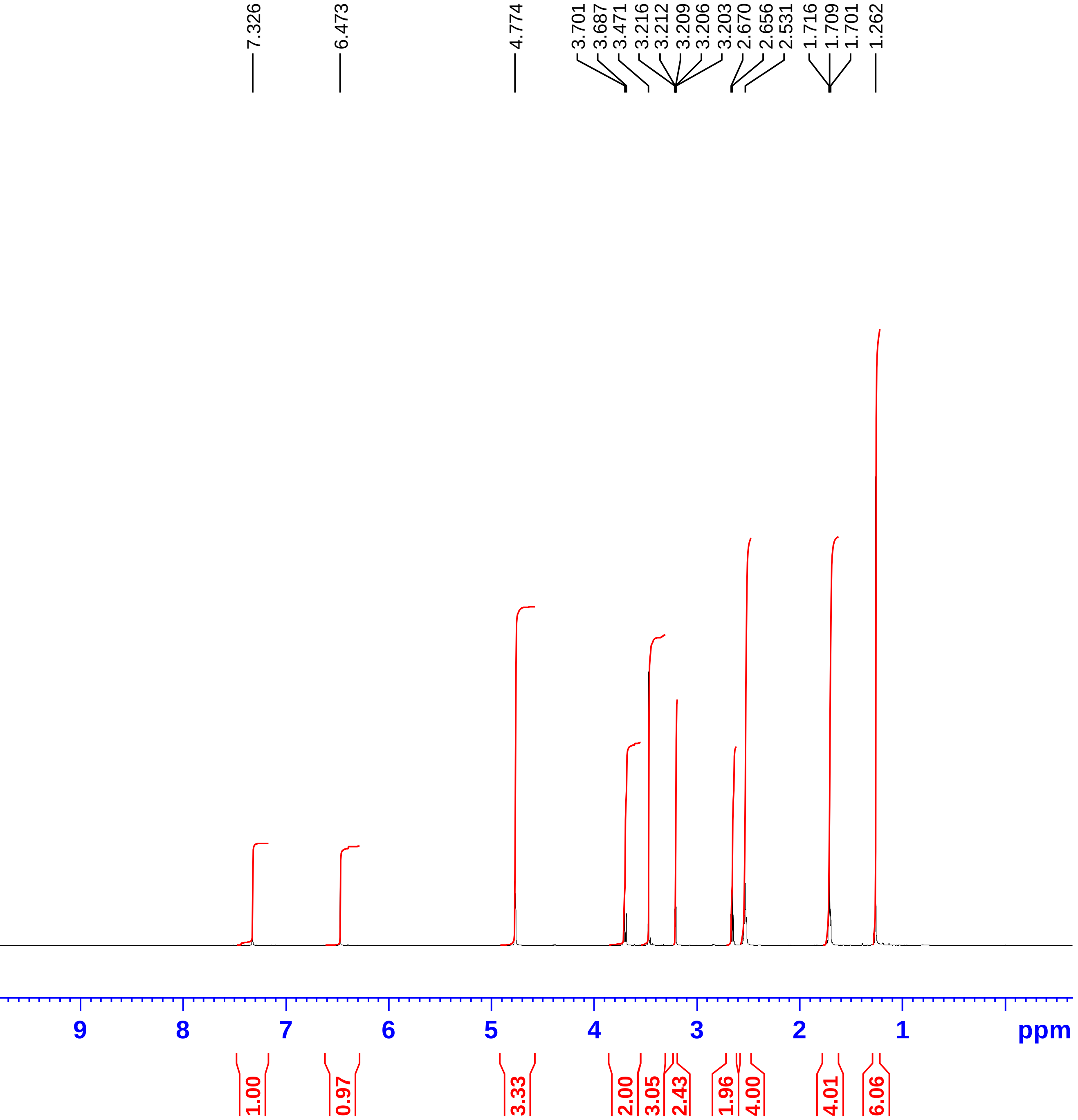

Current Data Parameters
NAME SA-500-16-2nd purification 11
EXPNO
PROCNO 1

F2 - Acquisition Parameters
Date_ 20150116
Time 1.57
INSTRUM spect
PROBHD 5 mm PABBO BB-
PULPROG zg30
TD 65536
SOLVENT MeOD
NS 16
DS 0
SWH 10330.578 Hz
FIDRES 0.157632 Hz
AQ 3.1719425 sec
RG 362
DW 48.400 usec
DE 11.99 usec
TE 295.0 K
D1 1.00000000 sec
TD0 1

======== CHANNEL f1 ========
SFO1 500.3030896 MHz
NUC1 1H
P1 16.25 usec
PLW1 18.33600044 W

F2 - Processing parameters
SI 65536
SF 500.3000602 MHz
WDW EM
SSB 0
LB 0.30 Hz
GB 0
PC 1.00

14d

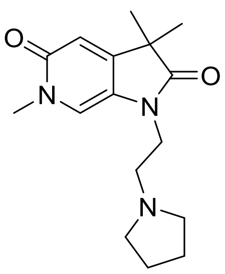

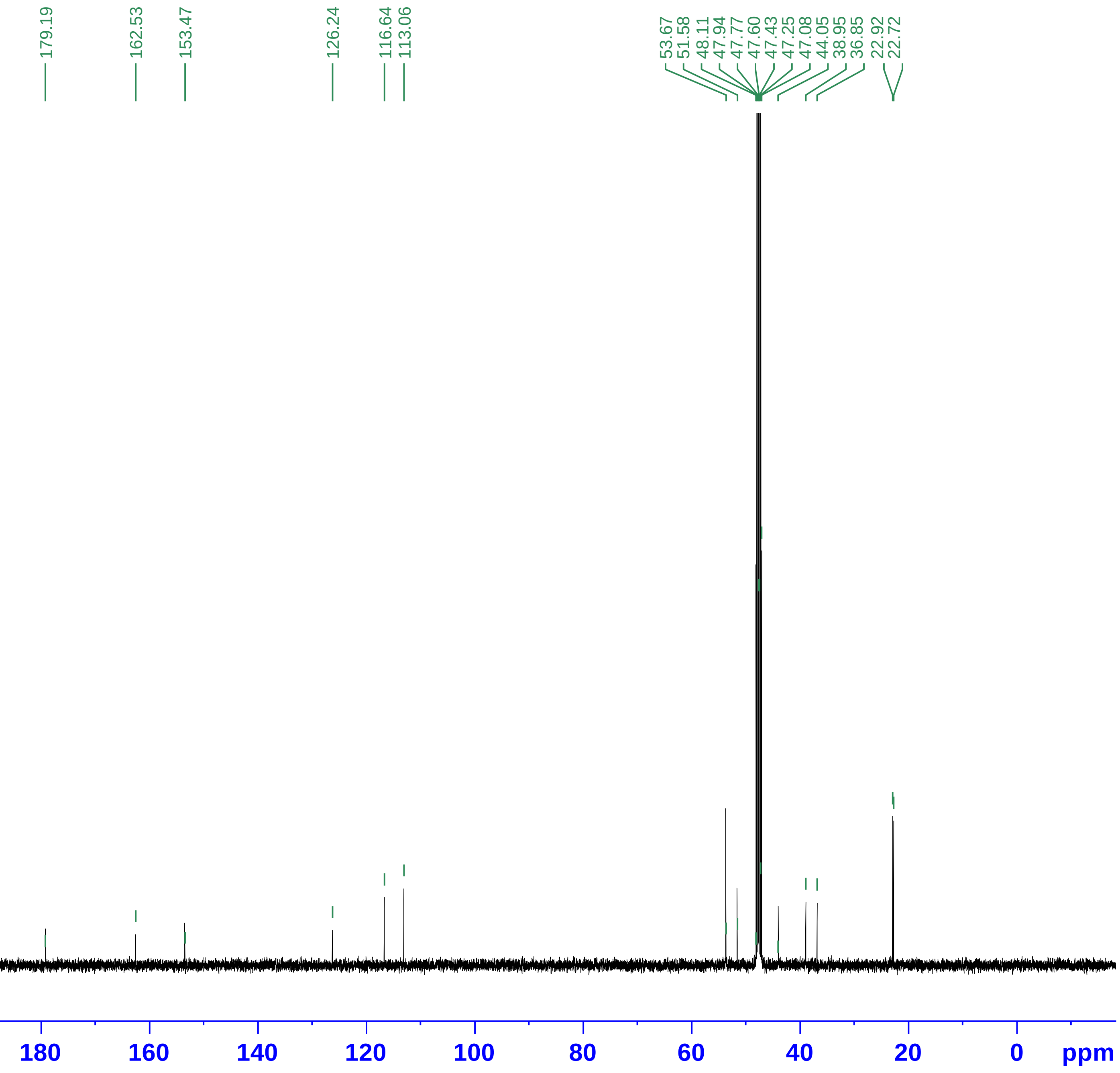

Current Data Parameters
NAME SA-500-16-2nd purification
EXPNO 10
PROCNO 1

F2 - Acquisition Parameters
Date_ 20150116
Time 1.54
INSTRUM spect
PROBHD 5 mm PABBO BB-
PULPROG zgpg30
TD 65536
SOLVENT MeOD
NS 256
DS 2
SWH 29761.904 Hz
FIDRES 0.454131 Hz
AQ 1.1010048 sec
RG 456
DW 16.800 usec
DE 7.68 usec
TE 296.9 K
D1 2.00000000 sec
D11 0.03000000 sec
TD0 1

======== CHANNEL f1 ========
SFO1 125.8131151 MHz
NUC1 13C
P1 9.75 usec
PLW1 82.38999939 W

======== CHANNEL f2 ========
SFO2 500.3020012 MHz
NUC2 1H
CPDPRG[2 waltz16
PCPD2 80.00 usec
PLW2 18.33600044 W
PLW12 0.75654000 W
PLW13 0.48418999 W

F2 - Processing Parameters
SI 65536
SF 125.8005350 MHz
WDW EM
SSB 0
LB 1.00 Hz
GB 0
PC 1.40

14e

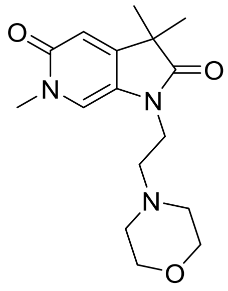

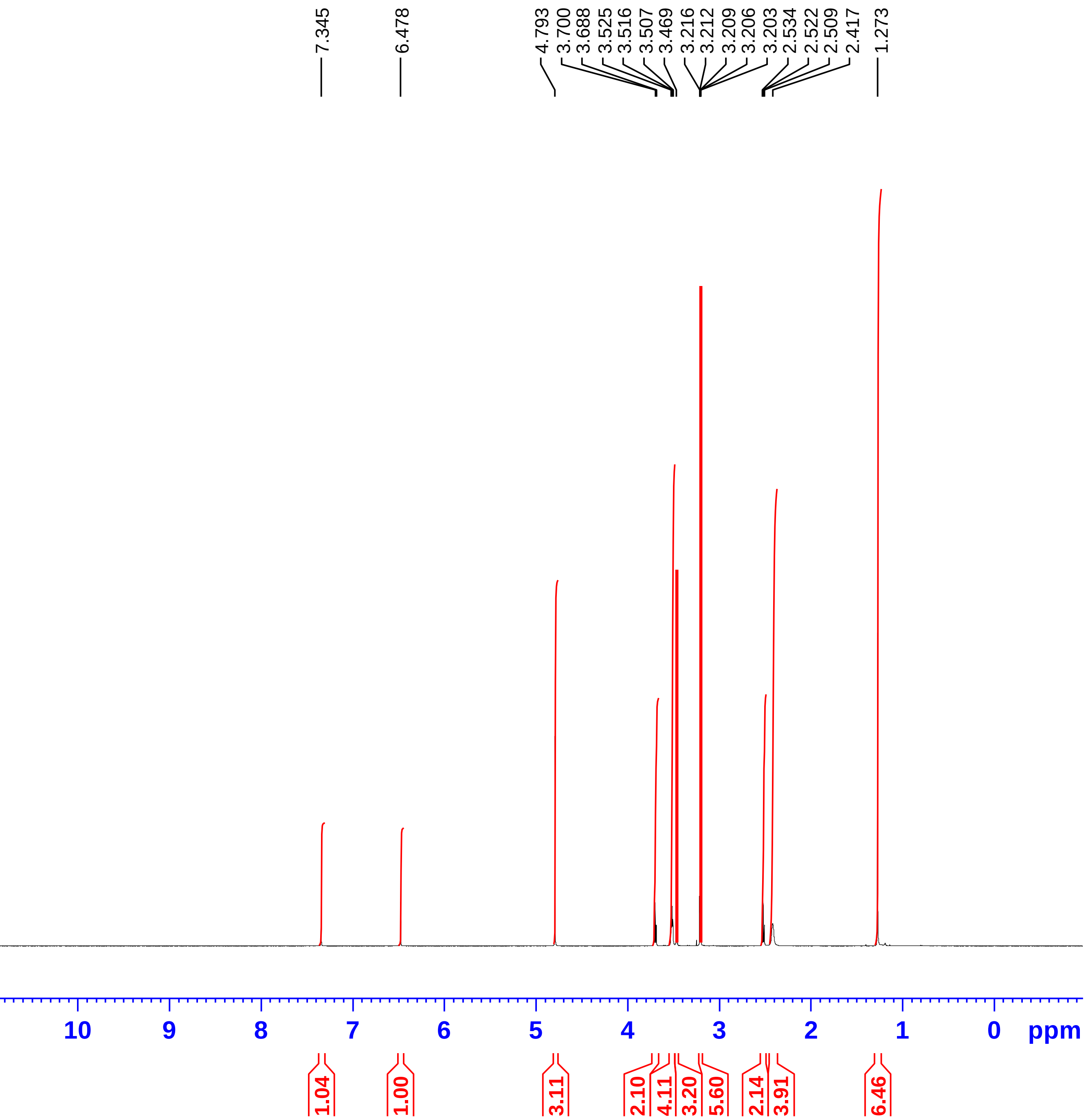

Current Data Parameters

NAME	SA-500-14
EXPNO	30
PROCNO	1

F2 - Acquisition Parameters

Date_	20150115
Time	15.20
INSTRUM	spect
PROBHD	5 mm PABBO BB-
PULPROG	zg30
TD	65536
SOLVENT	MeOD
NS	16
DS	0
SWH	10330.578 Hz
FIDRES	0.157632 Hz
AQ	3.1719425 sec
RG	512
DW	48.400 usec
DE	11.99 usec
TE	293.7 K
D1	1.00000000 sec
TD0	1

======== CHANNEL f1 ========

SFO1	500.3030896 MHz
NUC1	1H
P1	16.25 usec
PLW1	18.33600044 W

F2 - Processing parameters

SI	65536
SF	500.3000602 MHz
WDW	EM
SSB	0
LB	0.30 Hz
GB	0
PC	1.00

14e

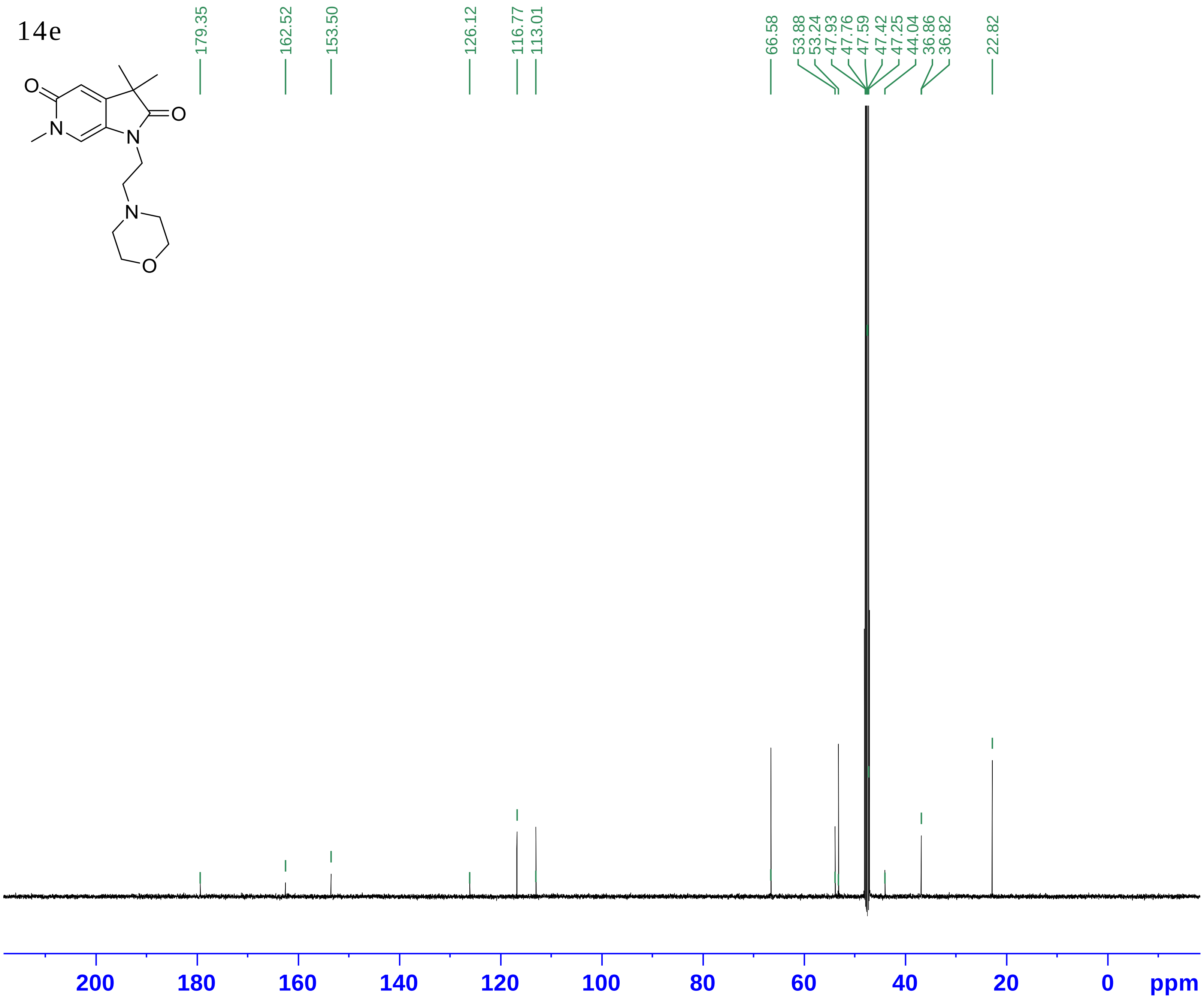

Current Data Parameters
NAME SA-500-14
EXPNO 35
PROCNO 1

F2 - Acquisition Parameters
Date_ 20150116
Time 1.36
INSTRUM spect
PROBHD 5 mm PABBO BB-
PULPROG zgpg
TD 26782
SOLVENT MeOD
NS 2048
DS 2
SWH 29761.904 Hz
FIDRES 1.111265 Hz
AQ 0.4499376 sec
RG 456
DW 16.800 usec
DE 7.72 usec
TE 296.6 K
D1 5.00000000 sec
D11 0.03000000 sec
TD0 1

======== CHANNEL f1 ========
SFO1 125.8131151 MHz
NUC1 13C
P1 9.75 usec
PLW1 82.38999939 W

======== CHANNEL f2 ========
SFO2 500.3020012 MHz
NUC2 1H
CPDPRG[2 waltz16
PCPD2 80.00 usec
PLW2 18.33600044 W
PLW12 0.75654000 W
PLW13 0.48418999 W

F2 - Processing Parameters
SI 65536
SF 125.8005350 MHz
WDW EM
SSB 0
LB 1.00 Hz
GB 0
PC 1.40

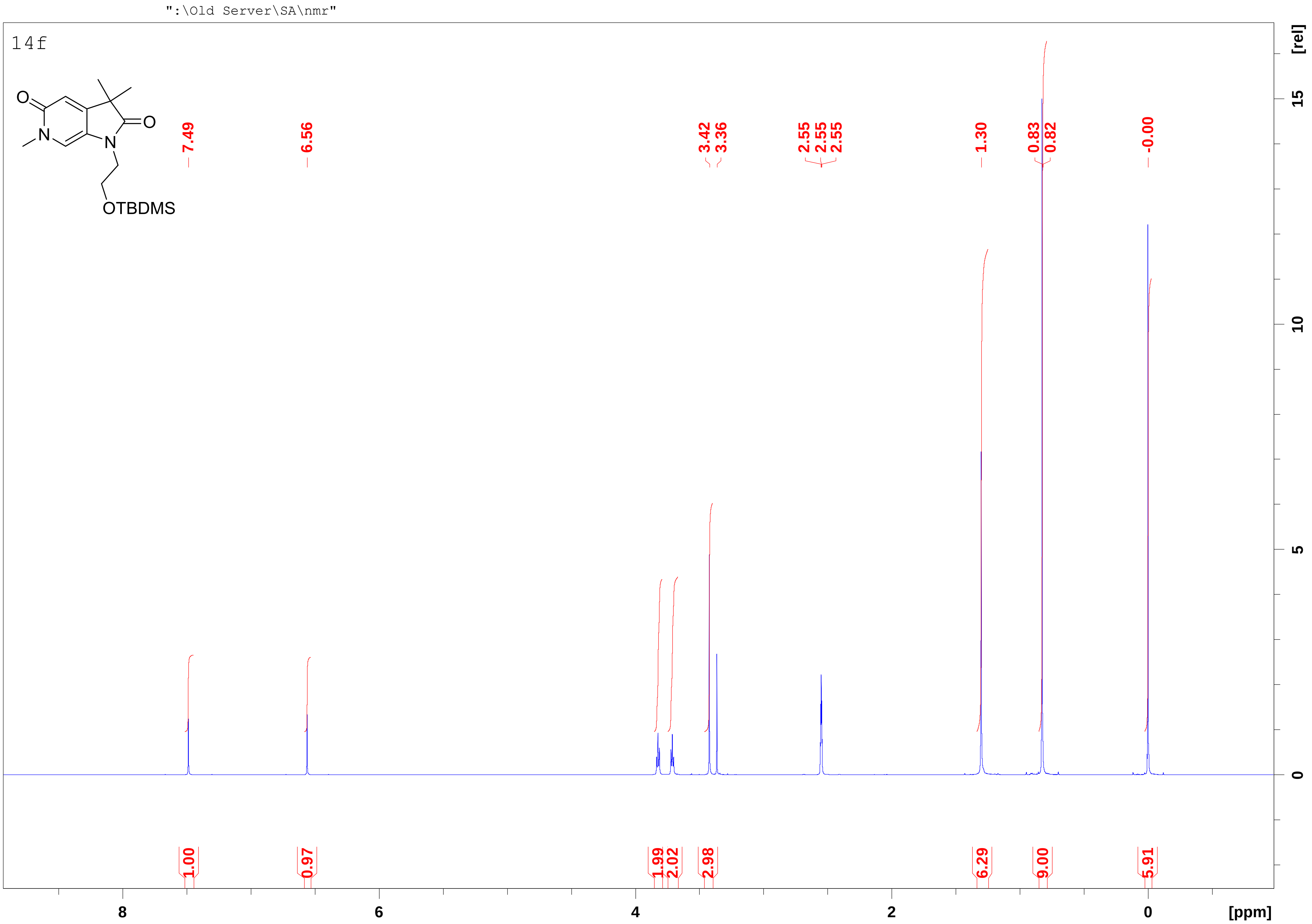

":\Old Server\SA\nmr"
14f
O
N
N
O
OTBDMS
7.49
6.56
3.42
3.36
2.55
2.55
2.55
1.30
0.83
0.82
-0.00
1.00
0.97
1.99
2.02
2.98
6.29
9.00
5.91
8
6
4
2
0
[ppm]
[rel]
15
10
5
0

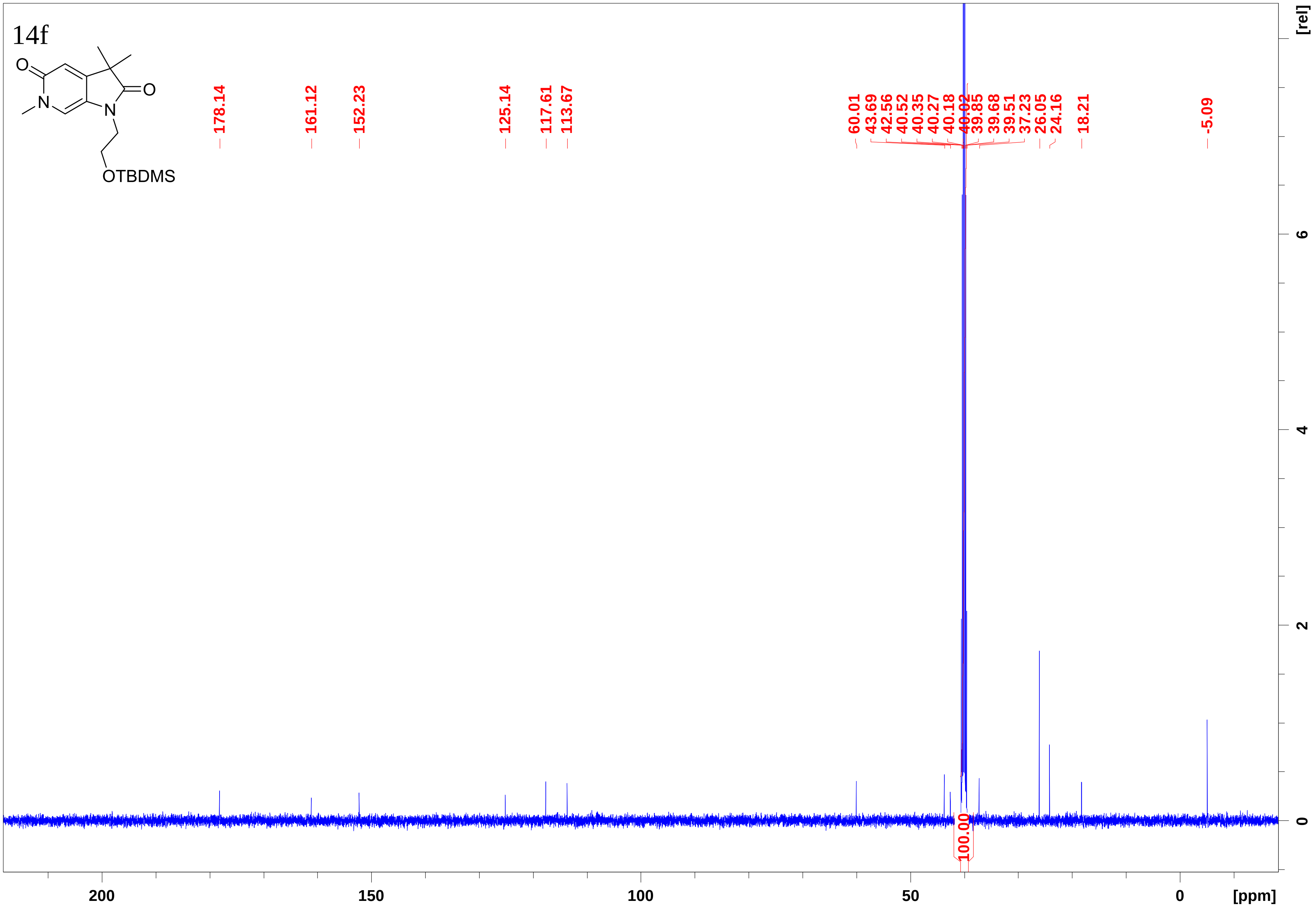

14f
O
N
N
O
OTBDMS
178.14
161.12
152.23
125.14
117.61
113.67
60.01
43.69
42.56
40.52
40.35
40.27
40.18
40.02
39.85
39.68
39.51
37.23
26.05
24.16
18.21
-5.09
100.00
[rel]
6
4
2
0
200
150
100
50
0
[ppm]

14g

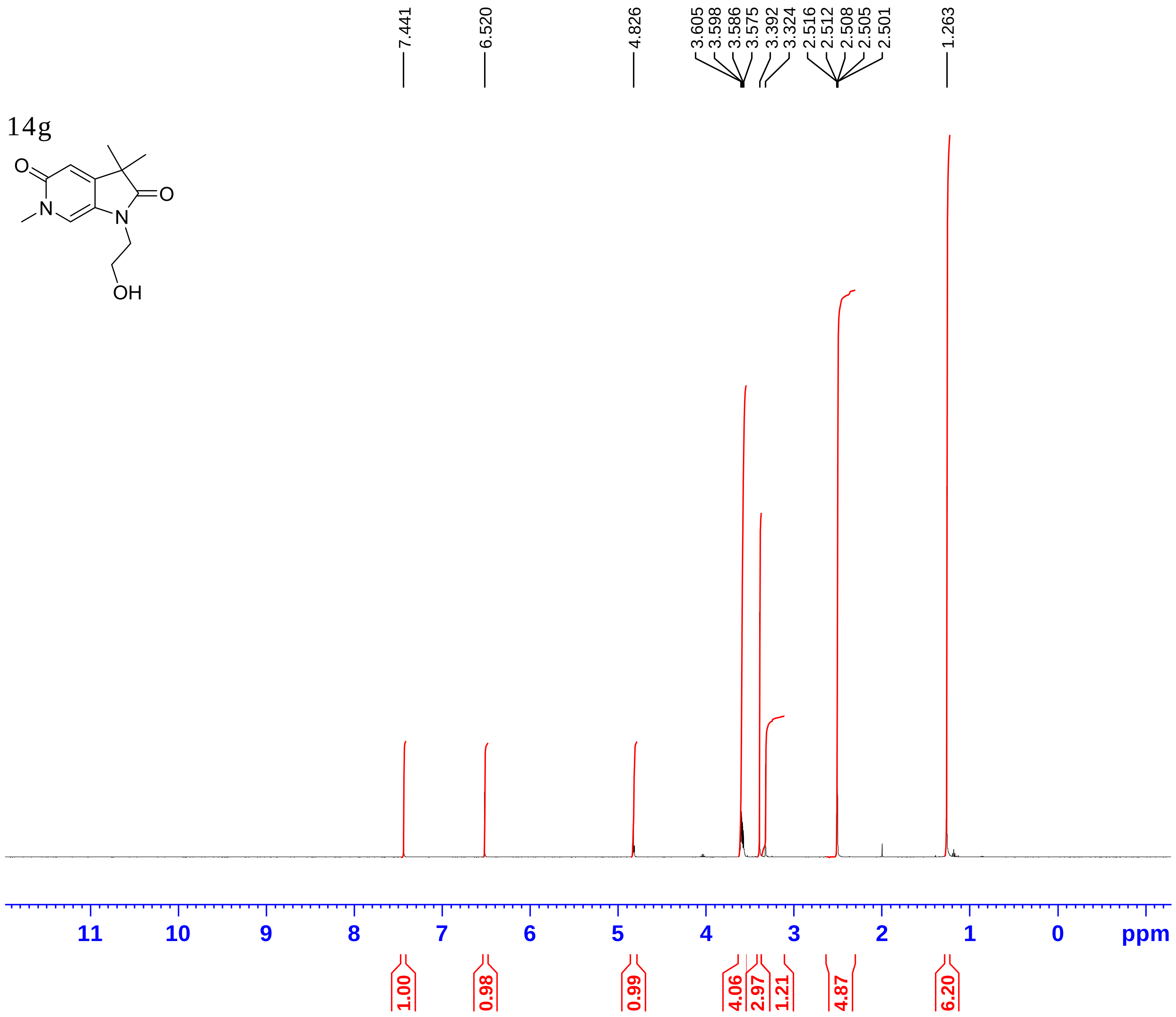

Current Data Parameters

NAME	SA-473-216
EXPNO	20
PROCNO	1

F2 - Acquisition Parameters

Date_	20141211
Time	14.32
INSTRUM	spect
PROBHD	5 mm PABBO BB-
PULPROG	zg30
TD	65536
SOLVENT	DMSO
NS	16
DS	0
SWH	10330.578 Hz
FIDRES	0.157632 Hz
AQ	3.1719425 sec
RG	575
DW	48.400 usec
DE	11.99 usec
TE	297.7 K
D1	1.00000000 sec
TD0	1

======== CHANNEL f1 ========

SFO1	500.3030896 MHz
NUC1	1H
P1	16.25 usec
PLW1	18.33600044 W

F2 - Processing parameters SI 65536

SF	500.3000000 MHz
WDW	EM
SSB	0
LB	0.30 Hz
GB	0
PC	1.00

14g

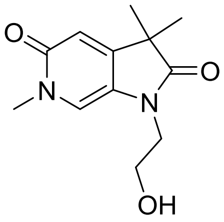

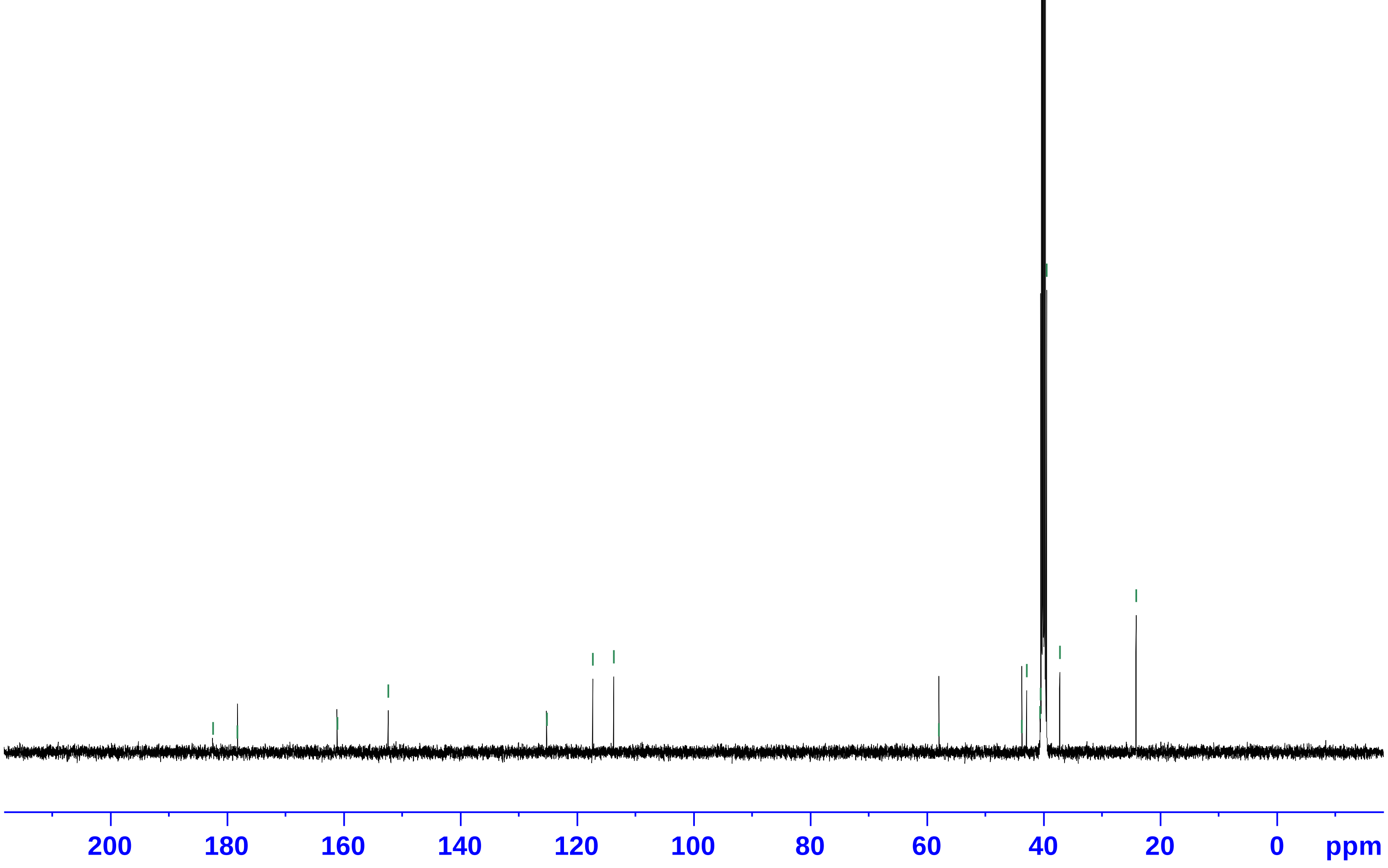

Current Data Parameters
NAME SA-473-216
EXPNO 25
PROCNO 1

F2 - Acquisition Parameters
Date_ 20141212
Time 0.55
INSTRUM spect
PROBHD 5 mm PABBO BB-
PULPROG zgpg30
TD 65536
SOLVENT DMSO
NS 1024
DS 2
SWH 29761.904 Hz
FIDRES 0.454131 Hz
AQ 1.1010048 sec
RG 456
DW 16.800 usec
DE 7.68 usec
TE 298.0 K
D1 2.00000000 sec
D11 0.03000000 sec
TD0 1

======== CHANNEL f1 ========

SFO1 125.8131151 MHz
NUC1 13C
P1 9.75 usec
PLW1 82.38999939 W

======== CHANNEL f2 ========
SFO2 500.3020012 MHz
NUC2 1H
CPDPRG[2 waltz16
PCPD2 80.00 usec
PLW2 18.33600044 W
PLW12 0.75654000 W
PLW13 0.48418999 W

F2 - Processing Parameters
SI 65536
SF 125.8005350 MHz
WDW EM
SSB 0
LB 1.00 Hz
GB 0
PC 1.40

15c

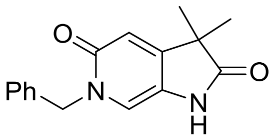

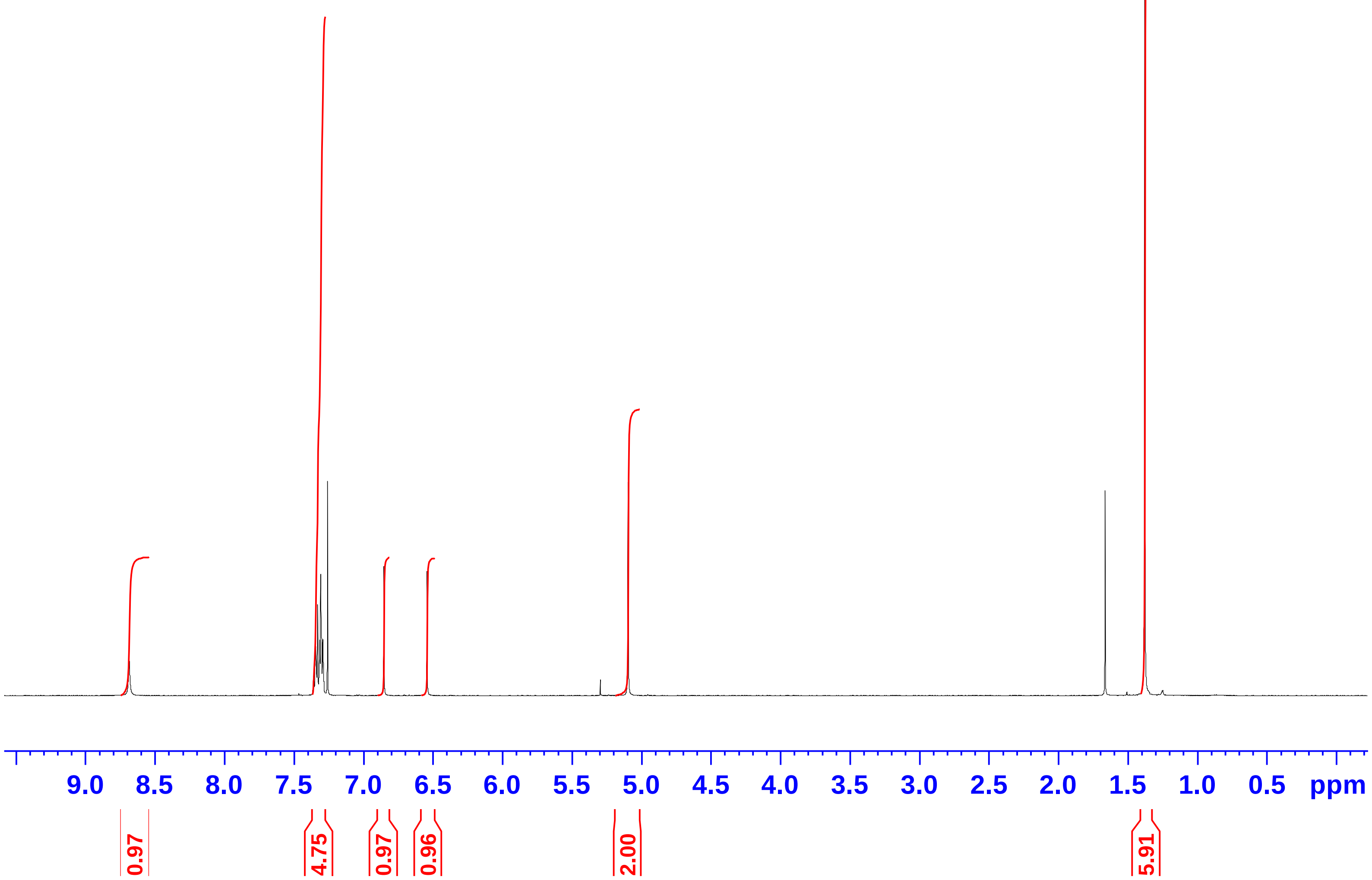

Current Data Parameters
NAME DCM-494-73-B CDCL3 CONC
EXPNO 11
PROCNO 1

F2 - Acquisition Parameters
Date_ 20141029
Time 8.32
INSTRUM spect
PROBHD 5 mm PABBO BB-
PULPROG zg30
TD 65536
SOLVENT CDCl3
NS 16
DS 0
SWH 10330.578 Hz
FIDRES 0.157632 Hz
AQ 3.1719425 sec
RG 456
DW 48.400 usec
DE 11.99 usec
TE 298.0 K
D1 1.00000000 sec
TD0 1

======== CHANNEL f1 ========
SFO1 500.3030896 MHz
NUC1 1H
P1 16.25 usec
PLW1 18.33600044 W

F2 - Processing Parameters
SI 65536
SF 500.3000125 MHz
WDW EM
SSB 0
LB 0.30 Hz
GB 0
PC 1.00

15c

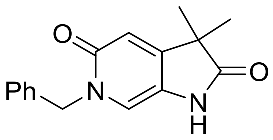

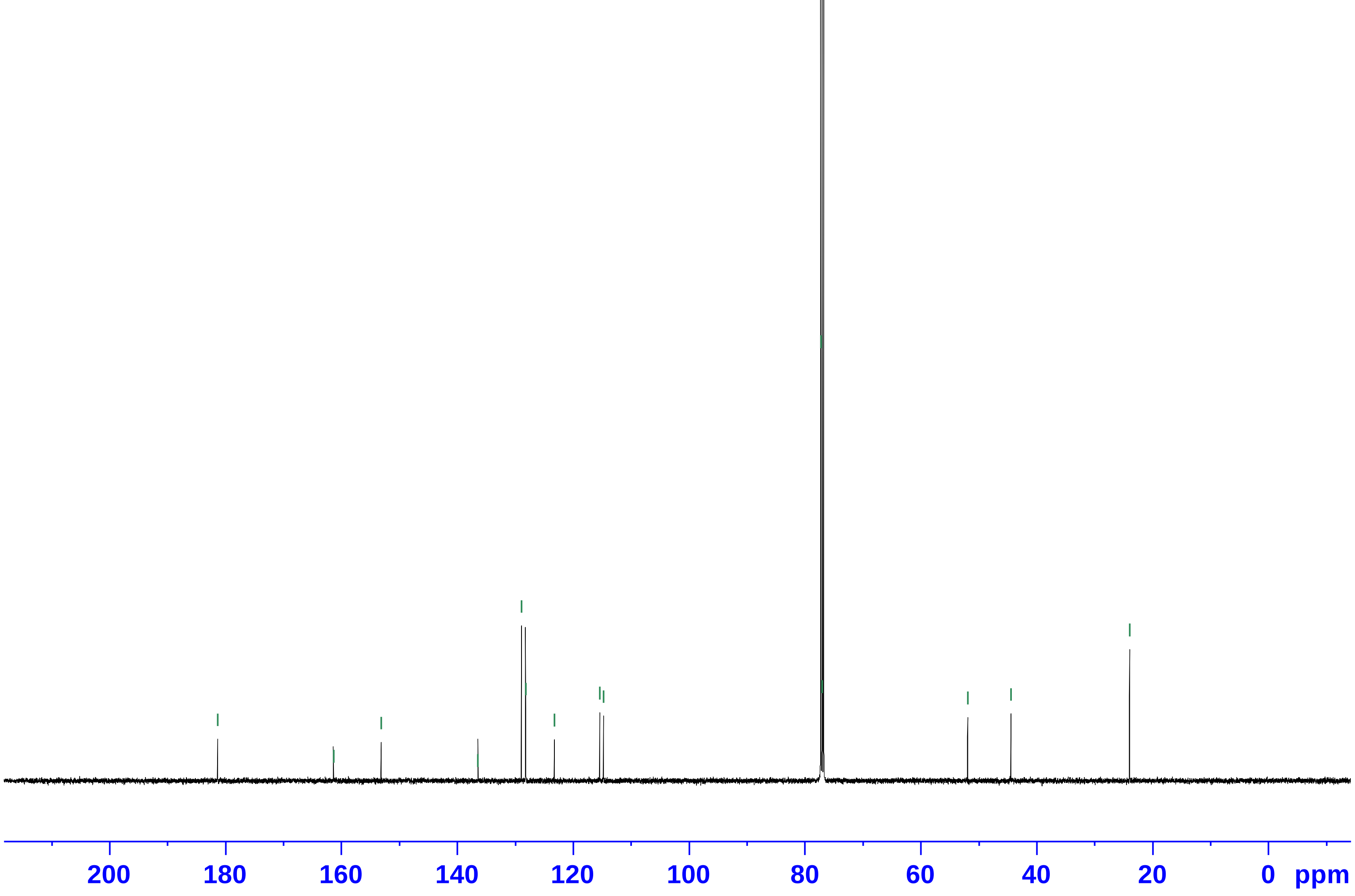

Current Data Parameters
NAME DCM-494-73-B CDCL3 CONC
EXPNO 10
PROCNO 1

F2 - Acquisition Parameters
Date_ 20141029
Time 4.42
INSTRUM spect
PROBHD 5 mm PABBO BB-
PULPROG zgpg30
TD 65536
SOLVEN CDCl3
T NS 2048
DS 2
SWH 29761.904 Hz
FIDRES 0.454131 Hz
AQ 1.1010048 sec
RG 575
DW 16.800 usec
DE 7.68 usec
TE 298.1 K
D1 2.00000000 sec
D11 0.03000000 sec
TD0 1

======== CHANNEL f1 ========
SFO1 125.8131151 MHz
NUC1 13C
P1 9.75 usec
PLW1 82.38999939 W

======== CHANNEL f2 ========
SFO2 500.3020012 MHz
NUC2 1H
CPDPRG[2 waltz16
PCPD2 80.00 usec
PLW2 18.33600044 W
PLW12 0.75654000 W
PLW13 0.48418999 W

F2 - Processing parameters
SI 65536
SF 125.8005350 MHz
WDW EM
SSB 0
LB 1.00 Hz
GB 0
PC 1.40

15d

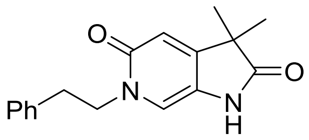

CDCL3

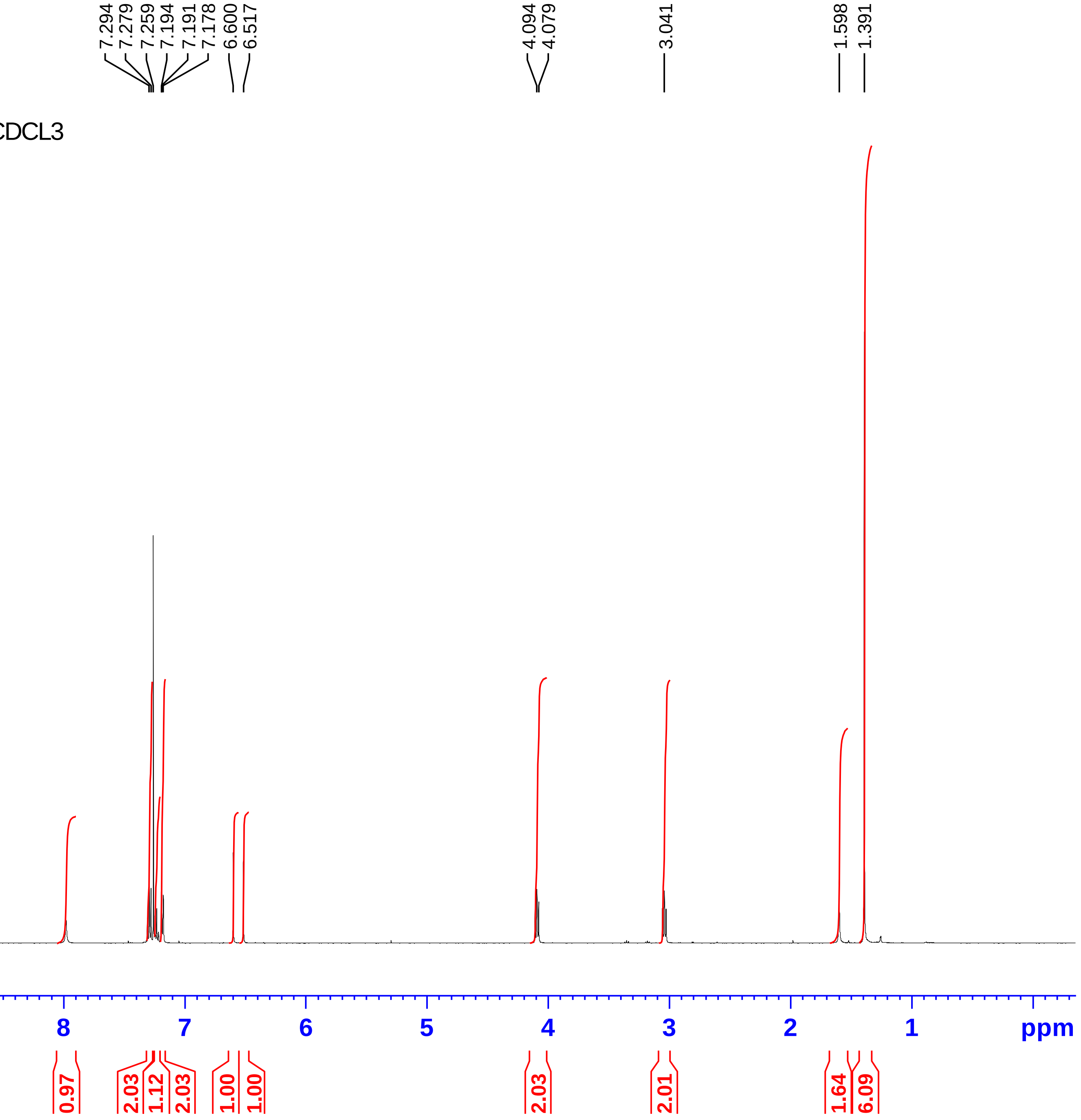

Current Data Parameters
NAME DCM-494-80-A CDCL3
EXPNO 10
PROCNO 1

F2 - Acquisition Parameters
Date_ 20141030
Time 14.22
INSTRUM spect
PROBHD 5 mm PABBO BB-
PULPROG zg30
TD 65536
SOLVENT CDCl3
NS 16
DS 0
SWH 10330.578 Hz
FIDRES 0.157632 Hz
AQ 3.1719425 sec
RG 645
DW 48.400 usec
DE 11.99 usec
TE 298.1 K
D1 1.00000000 sec
TD0 1

======== CHANNEL f1 ========
SFO1 500.3030896 MHz
NUC1 1H
P1 16.25 usec
PLW1 18.33600044 W

F2 - Processing parameters
SI 65536
SF 500.3000127 MHz
WDW EM
SSB 0
LB 0.30 Hz
GB 0
PC 1.00

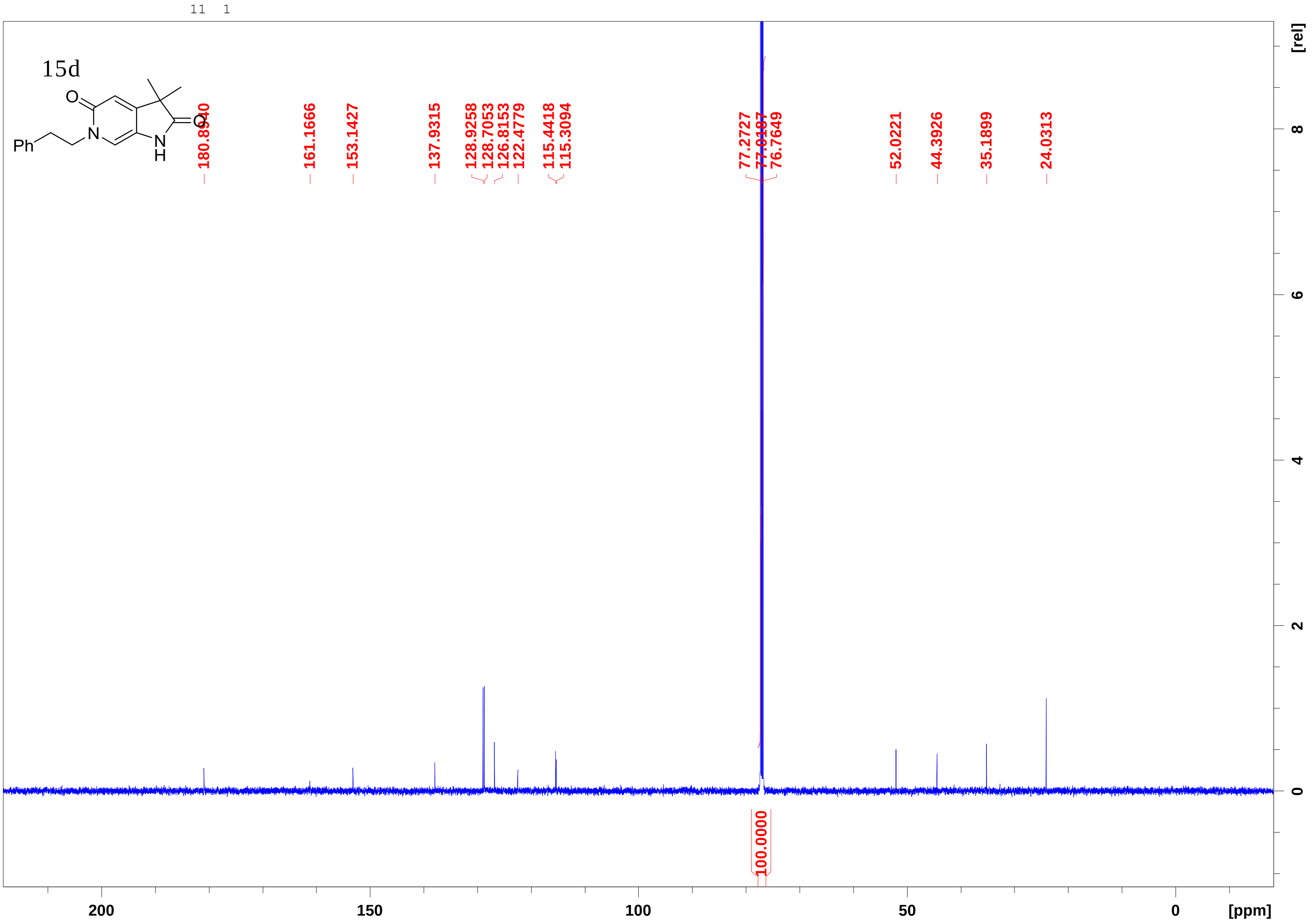

15d
Ph
N
O
N
H
180.8940
161.1666
153.1427
137.9315
128.9258
128.7053
126.8153
122.4779
115.4418
115.3094
77.2727
77.0187
76.7649
52.0221
44.3926
35.1899
24.0313
100.0000
200
150
100
50
0
[ppm]
[rel]
8
6
4
2
0

15e

CDCL3

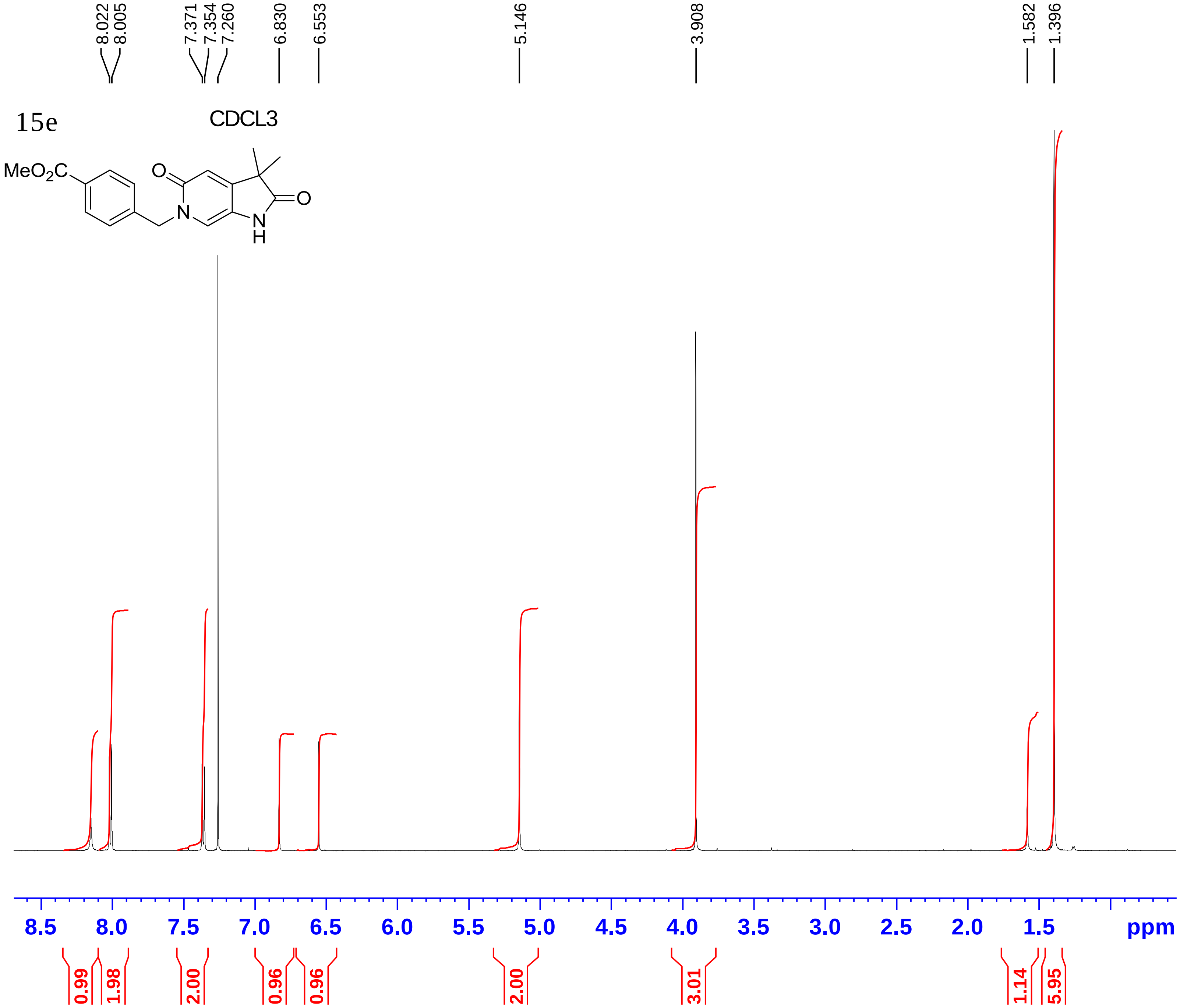

Current Data Parameters
NAME DCM-494-115-A CDCL3 CONC
EXPNO 10
PROCNO 1

F2 - Acquisition Parameters
Date_ 20141209
Time 16.21
INSTRUM spect
PROBHD 5 mm PABBO BB-
PULPROG zg30
TD 65536
SOLVENT CDCl3
NS 16
DS 0
SWH 10330.578 Hz
FIDRES 0.157632 Hz
AQ 3.1719425 sec
RG 645
DW 48.400 usec
DE 11.99 usec
TE 298.1 K
D1 1.00000000 sec
TD0 1

======== CHANNEL f1 ========
SFO1 500.3030896 MHz
NUC1 1H
P1 16.25 usec
PLW1 18.33600044 W

F2 - Processing parameters
SI 65536
SF 500.3000124 MHz
WDW EM
SSB 0
LB 0.30 Hz
GB 0
PC 1.00

CDCL3

15e

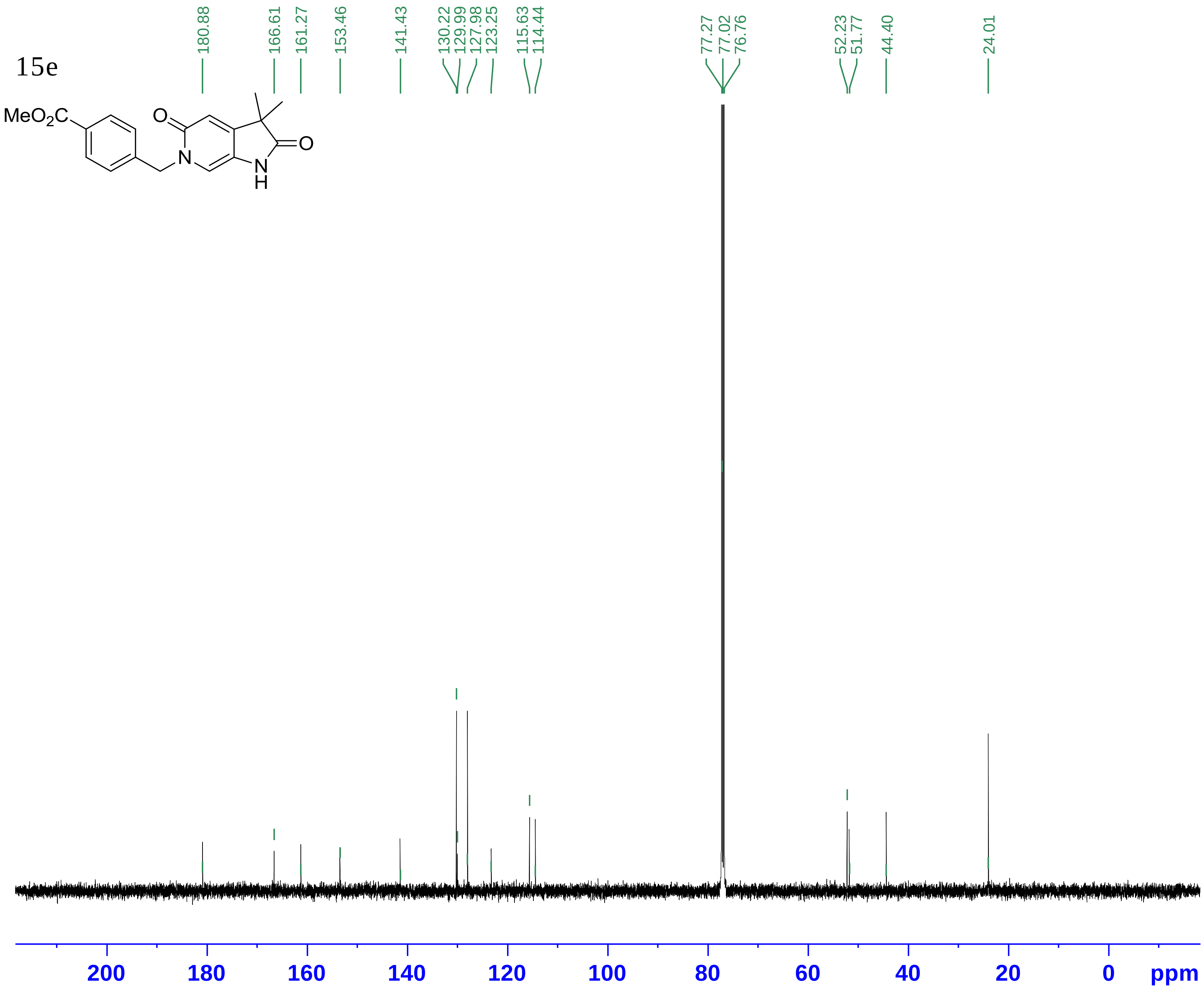

Current Data Parameters
NAME DCM-494-115-A CDCL3 CONC
EXPNO 12
PROCNO 1

F2 - Acquisition Parameters
Date 20141209
Time 23.02
INSTRUM spect
PROBHD 5 mm PABBO BB-
PULPROG zgpg3
TD 0
SOLVENT 6553
NS 6
DS CDCl3
SWH 2048
FIDRES 2
AQ 29761.904 Hz
RG 0.454131 Hz
DW 1.1010048 sec
DE 575
TE 16.800 usec
D1 7.68 usec
D11 298.0 K
TD0 2.00000000 sec
0.03000000 sec
1

======== CHANNEL f1 ========
SFO1 125.8131151 MHz
NUC1 13C
P1 9.75 usec
PLW1 82.38999939 W

======== CHANNEL f2 ========
SFO2 500.3020012 MHz
NUC2 1H
CPDPRG[2 waltz16
PCPD2 80.00 usec
PLW2 18.33600044 W
PLW12 0.75654000 W
PLW13 0.48418999 W

F2 - Processing Parameters
SI 65536
SF 125.8005350 MHz
WDW EM
SSB 0
LB 1.00 Hz
GB 0
PC 1.40

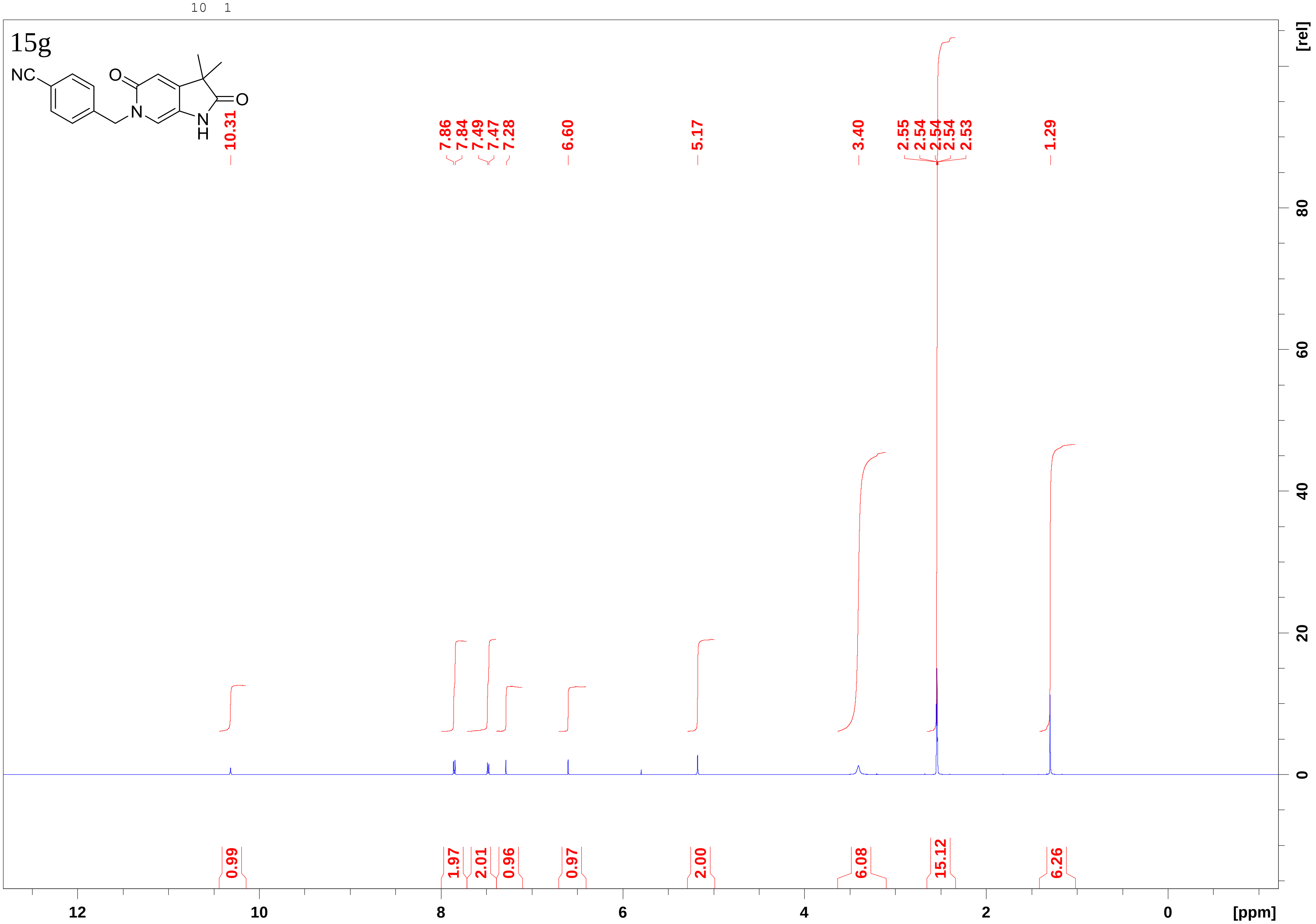

10 1
15g
NC
O
O
N
N
H
10.31
7.86
7.84
7.49
7.47
7.28
6.60
5.17
3.40
2.55
2.54
2.54
2.54
2.53
1.29
0.99
1.97
2.01
0.96
0.97
2.00
6.08
15.12
6.26
[rel]
80
60
40
20
0
12
10
8
6
4
2
0
[ppm]

15g

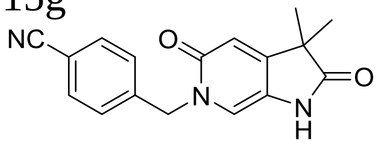

12 1 "V:\Old Server\DCM\nmr"

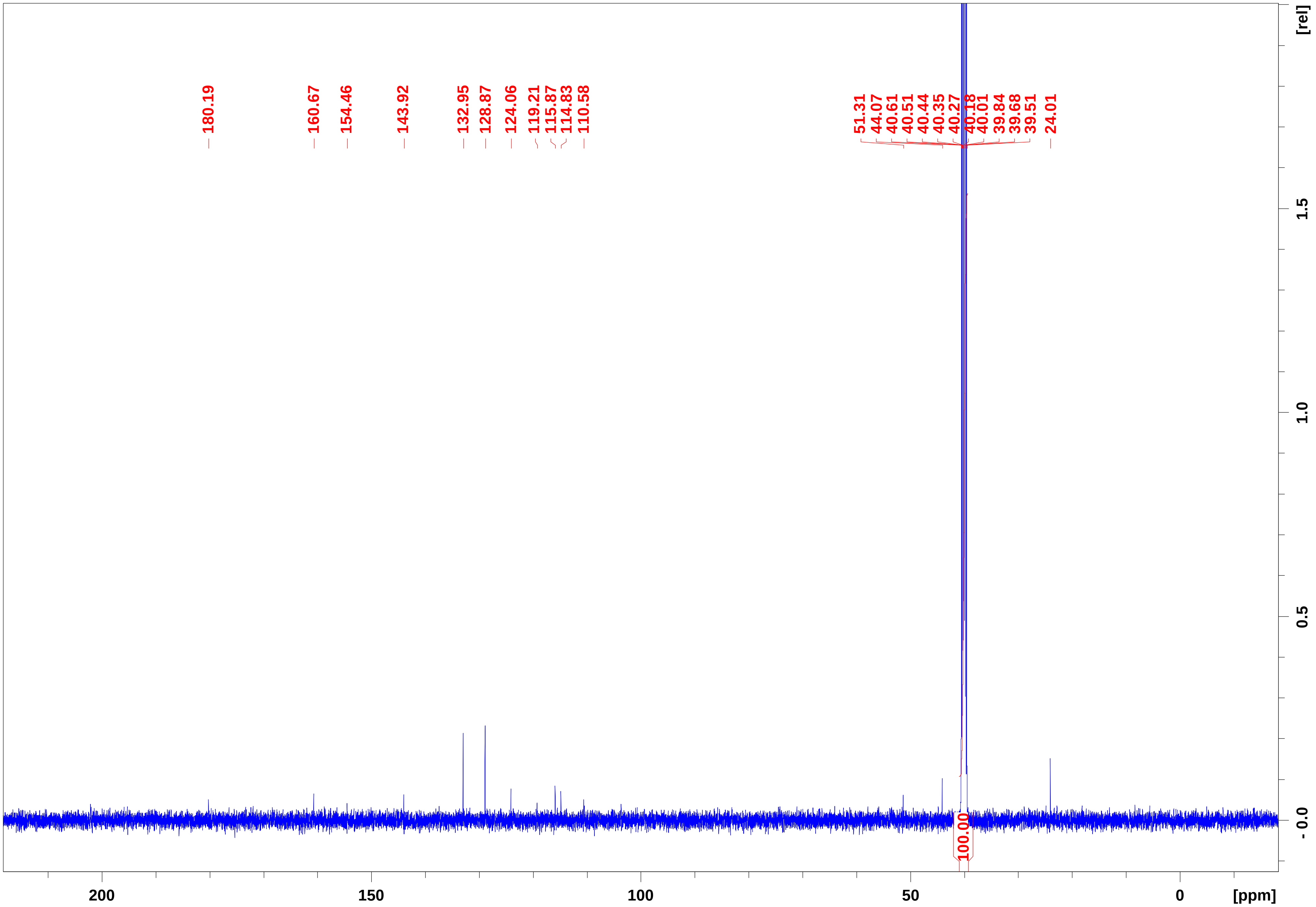

15i

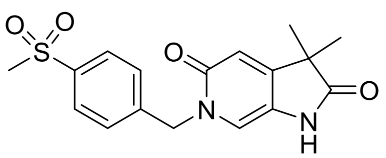

20 1 "V:\Old Server\SA\nmr"

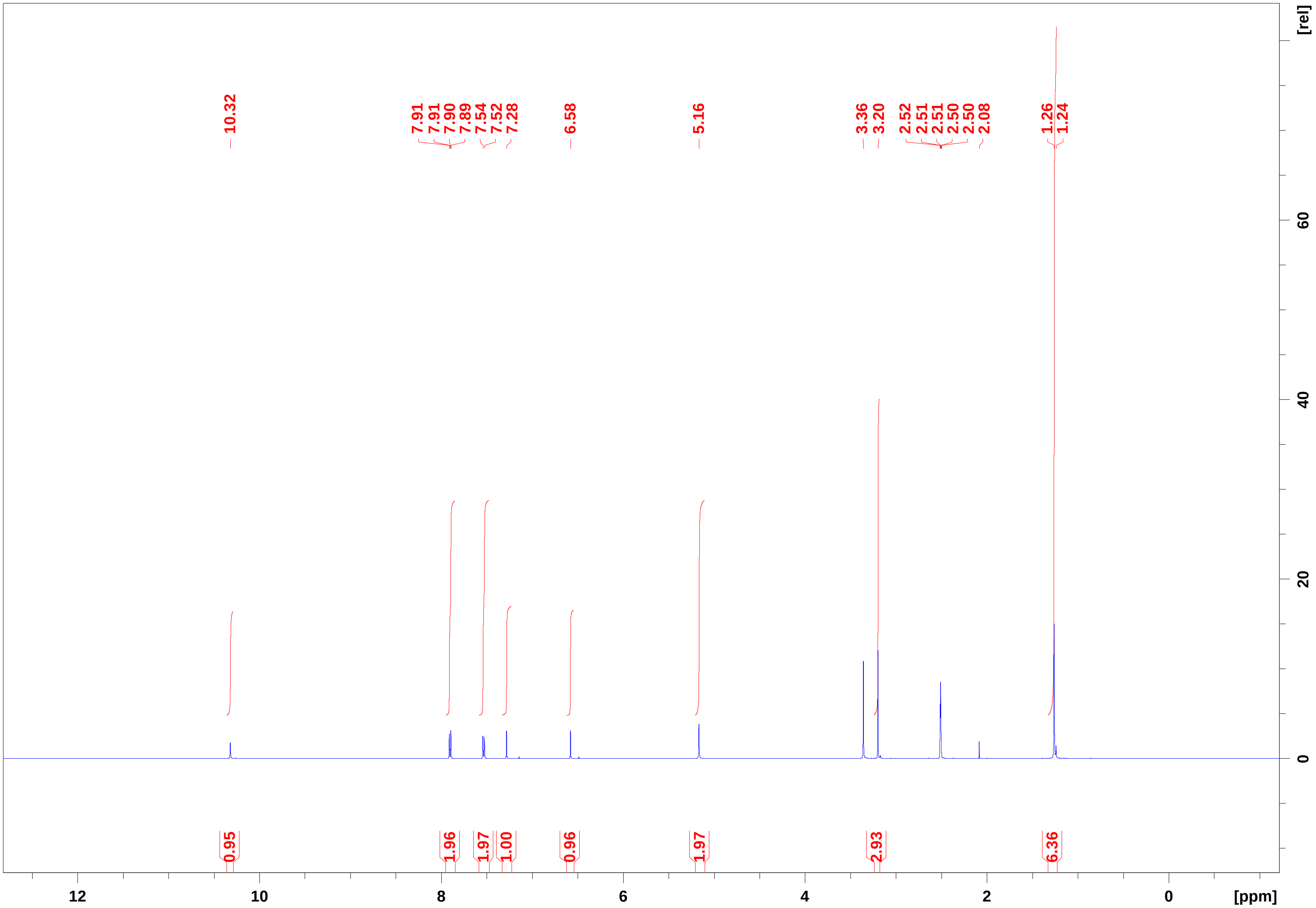

15i

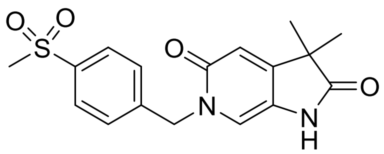

"V:\Old Server\SA\nmr"

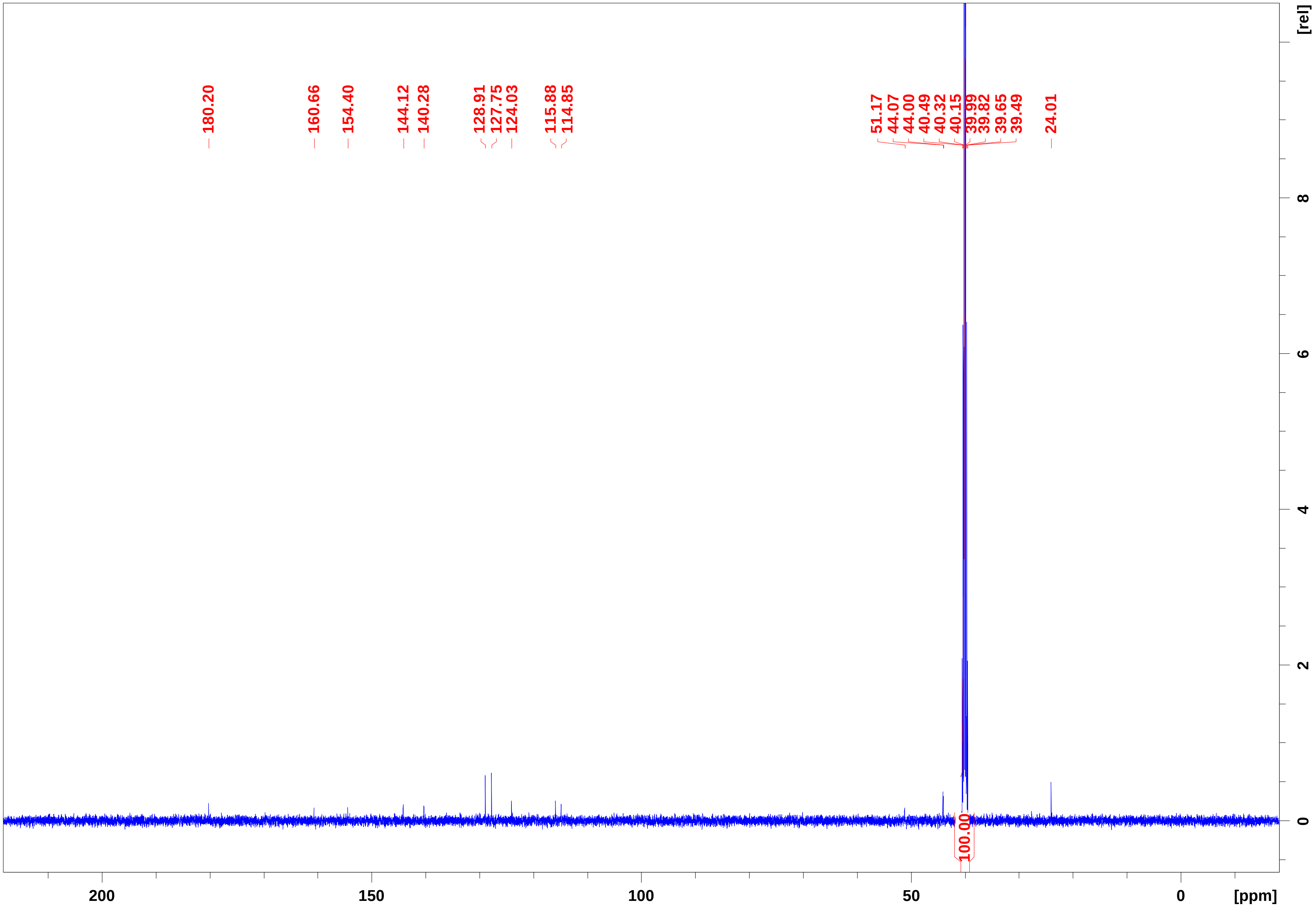

15j

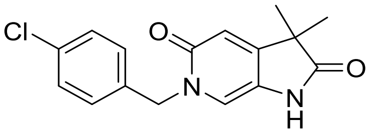

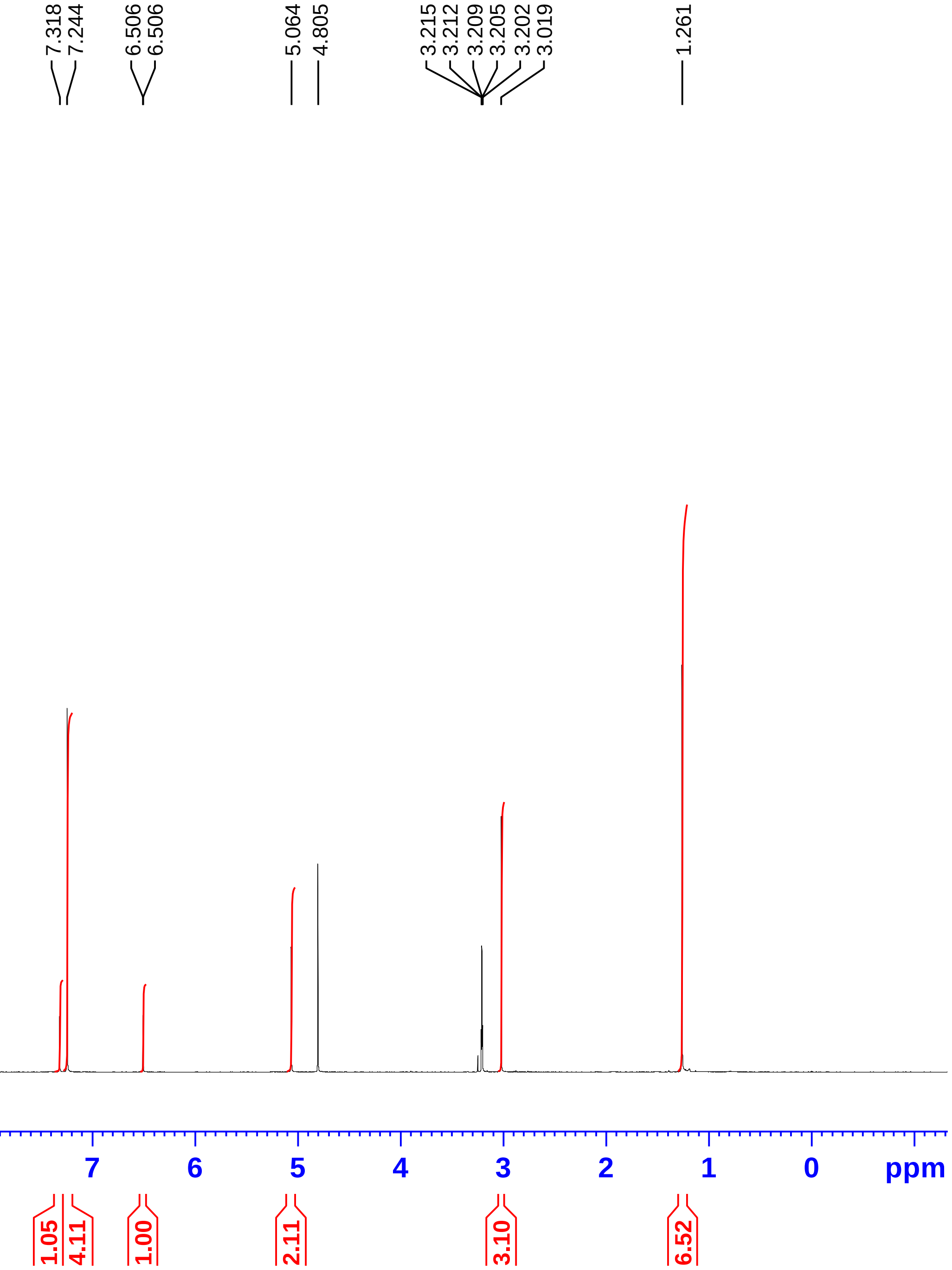

Current Data Parameters
NAME SA-500-42
EXPNO 10
PROCNO 1

F2 - Acquisition Parameters
Date_ 20150122
Time 17.08
INSTRUM spect
PROBHD 5 mm PABBO BB-
PULPROG zg30
TD 65536
SOLVENT MeOD
NS 16
DS 0
SWH 10330.578 Hz
FIDRES 0.157632 Hz
AQ 3.1719425 sec
RG 456
DW 48.400 usec
DE 11.99 usec
TE 292.6 K
D1 1.00000000 sec
TD0 1

======== CHANNEL f1 ========
SFO1 500.3030896 MHz
NUC1 1H
P1 16.25 usec
PLW1 18.33600044 W

F2 - Processing parameters SI 65536
SF 500.3000605 MHz
WDW EM
SSB 0
LB 0.30 Hz
GB 0
PC 1.00

15j

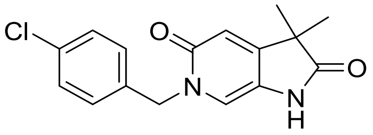

1 "V:\Old Server\SA\nmr"

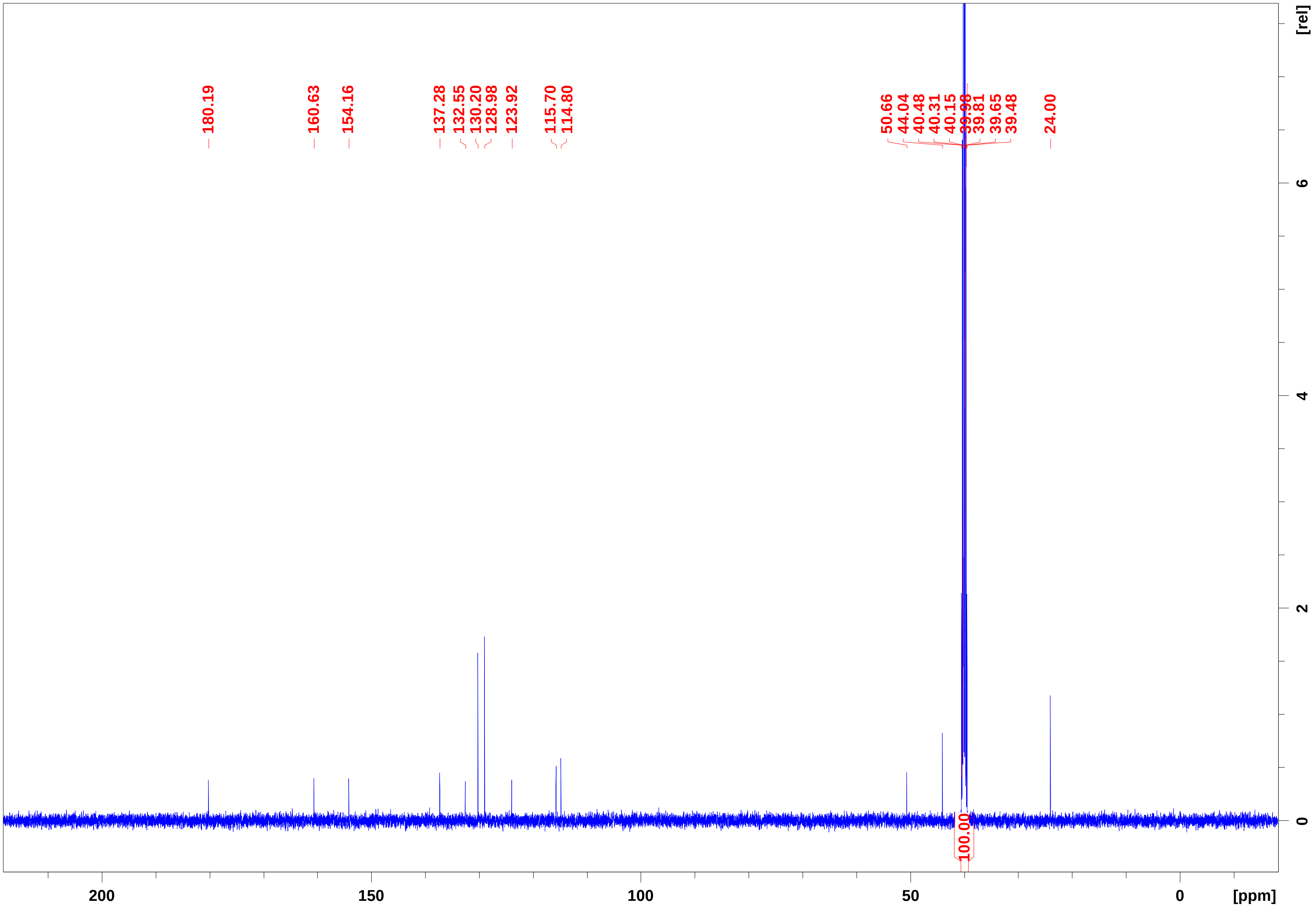

15k

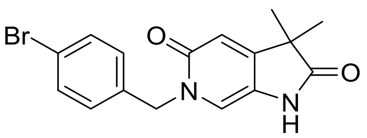

Br
O
N
N
H
O

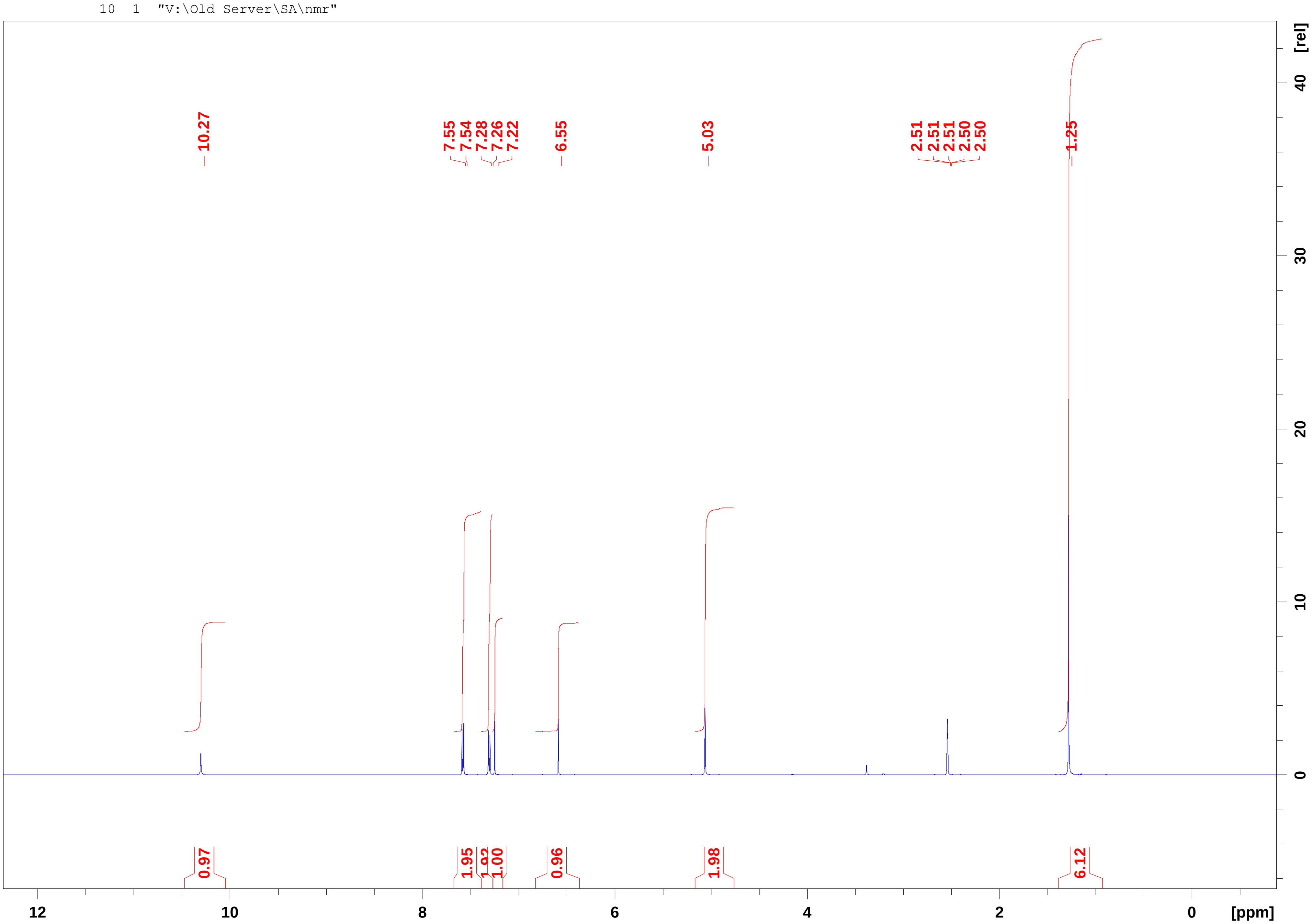

10 1 "V:\Old Server\SA\nmr"
10.27
7.55
7.54
7.28
7.26
7.22
6.55
5.03
2.51
2.51
2.51
2.50
2.50
1.25
0.97
1.95
1.00
0.96
1.98
6.12
12
10
8
6
4
2
0
[ppm]
[rel]
0
10
20
30
40

15k

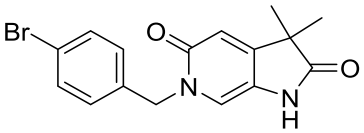

"V:\Old Server\SA\nmr"

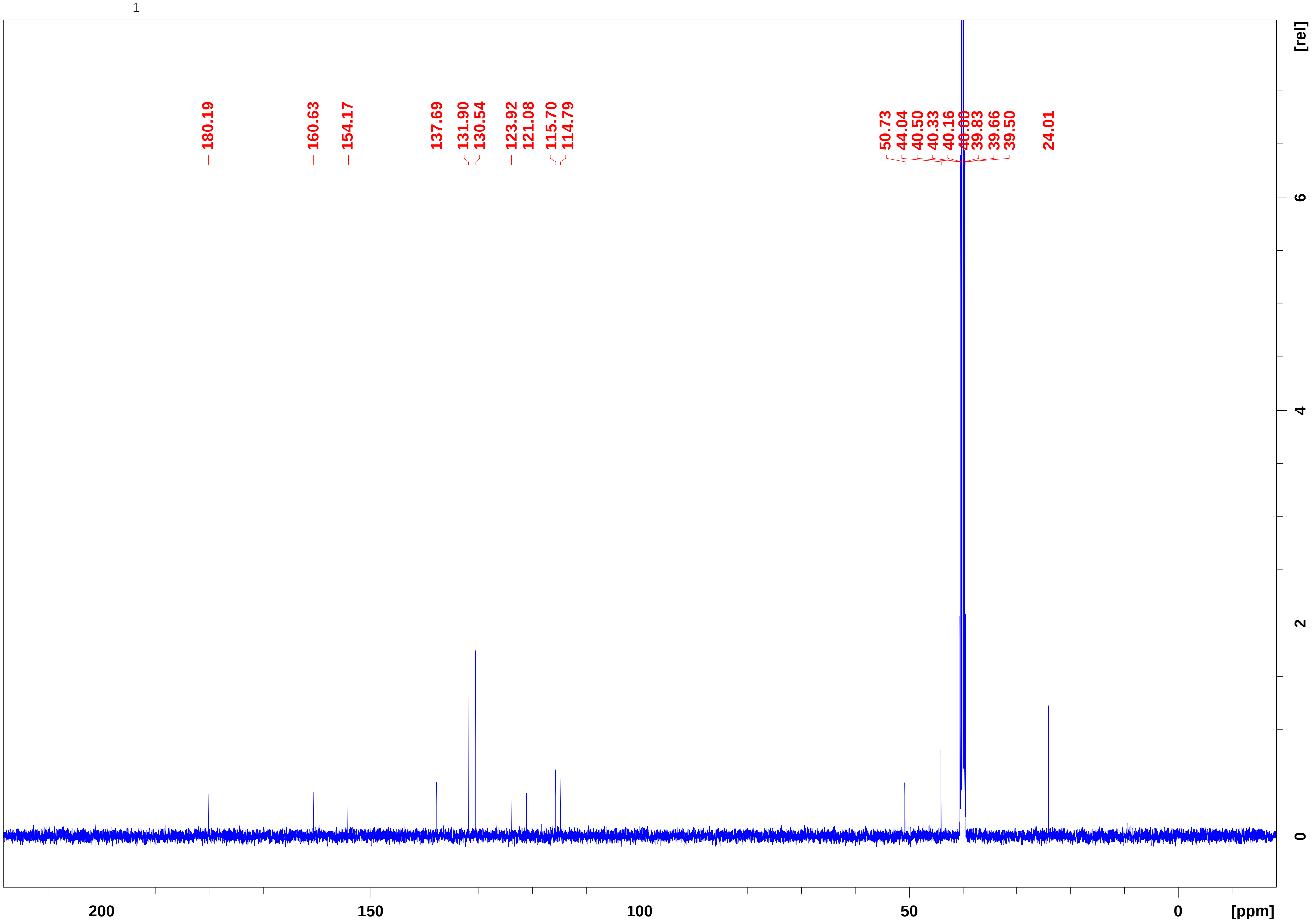

15l

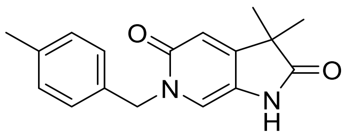

SA-500-72 10 1 "V:\Old Server\SA\nmr"

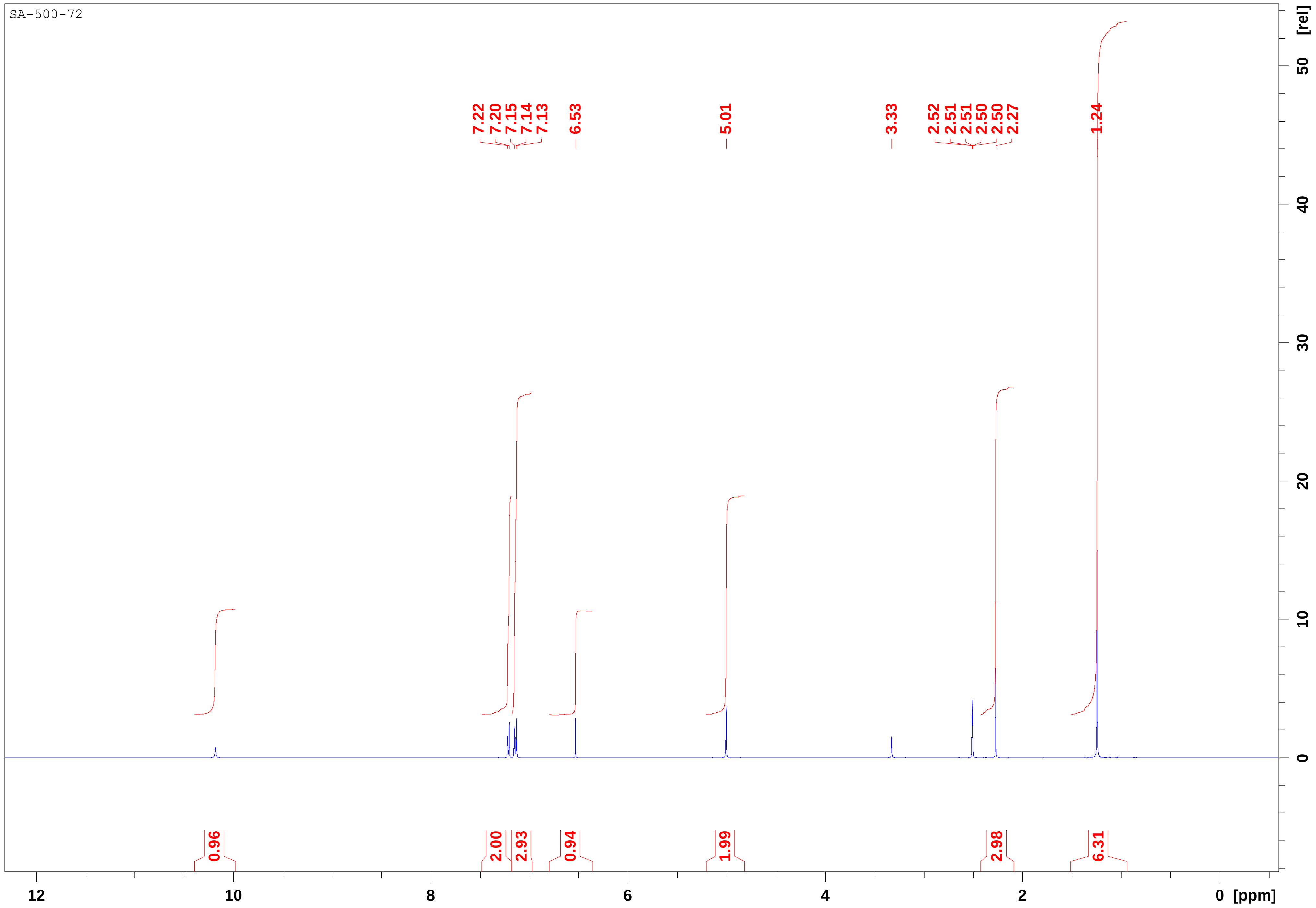

15l

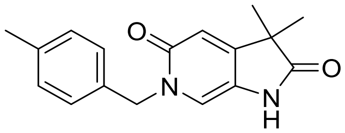

SA-500-72 11 1 "V:\Old Server\SA\nmr"

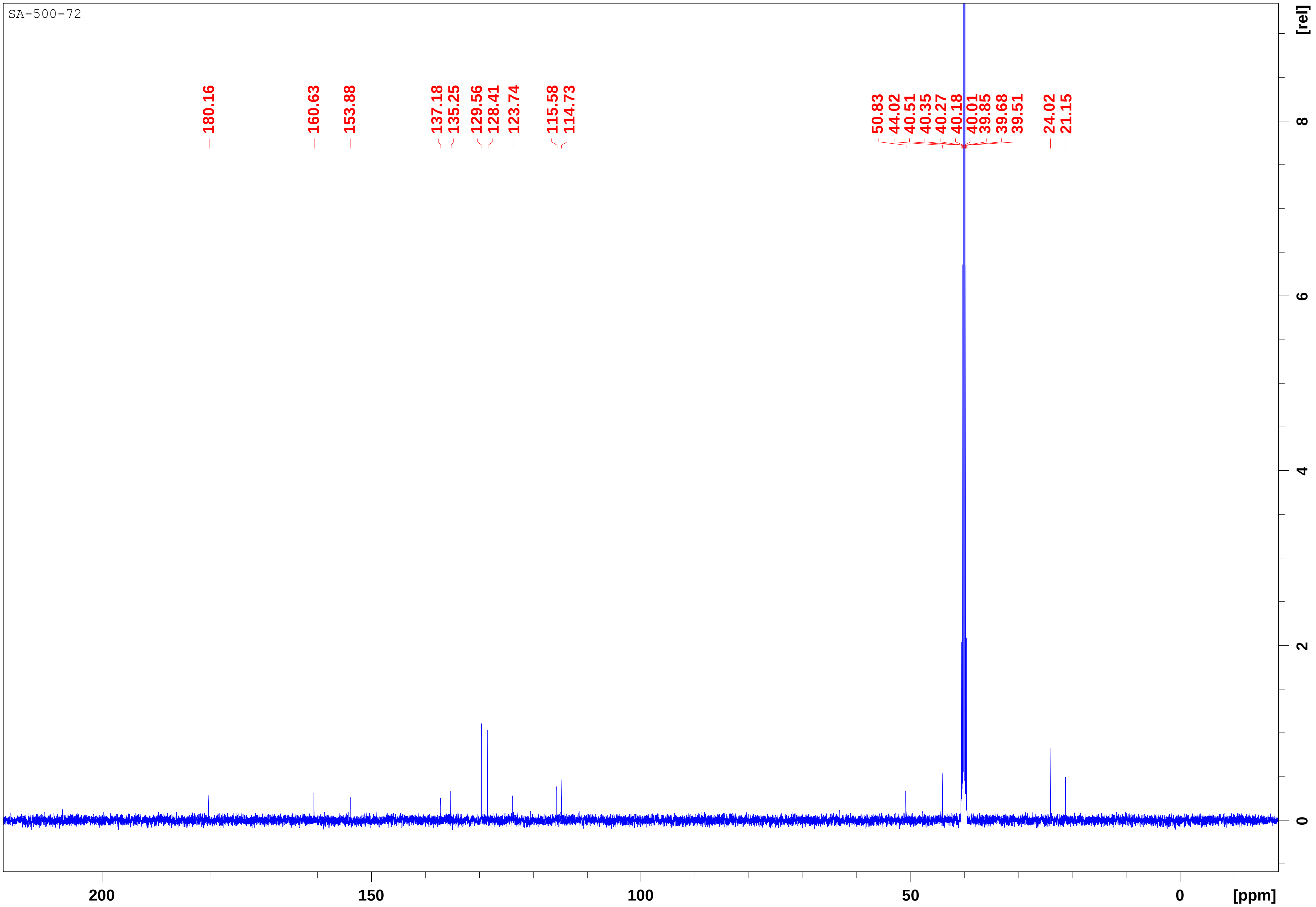

15m

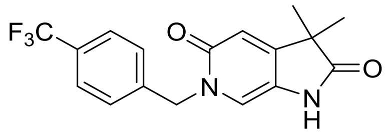

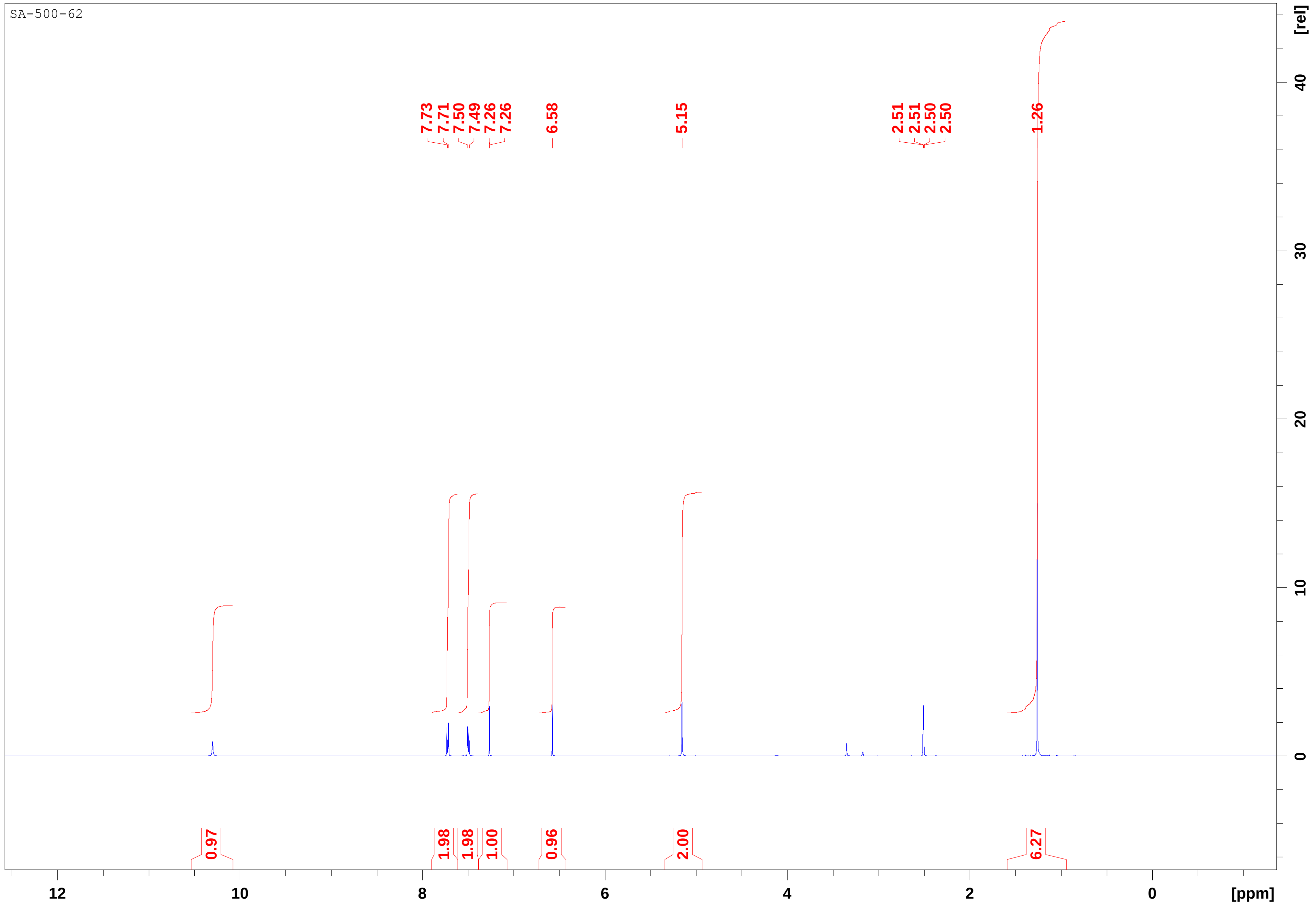

SA-500-62 10 1 "V:\Old Server\SA\nmr"
SA-500-62
7.73
7.71
7.50
7.49
7.26
7.26
6.58
5.15
2.51
2.51
2.50
2.50
1.26
0.97
1.98
1.98
1.00
0.96
2.00
6.27
[rel]
[ppm]

15m

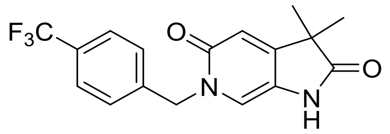
F3C
O
N
N
H
O

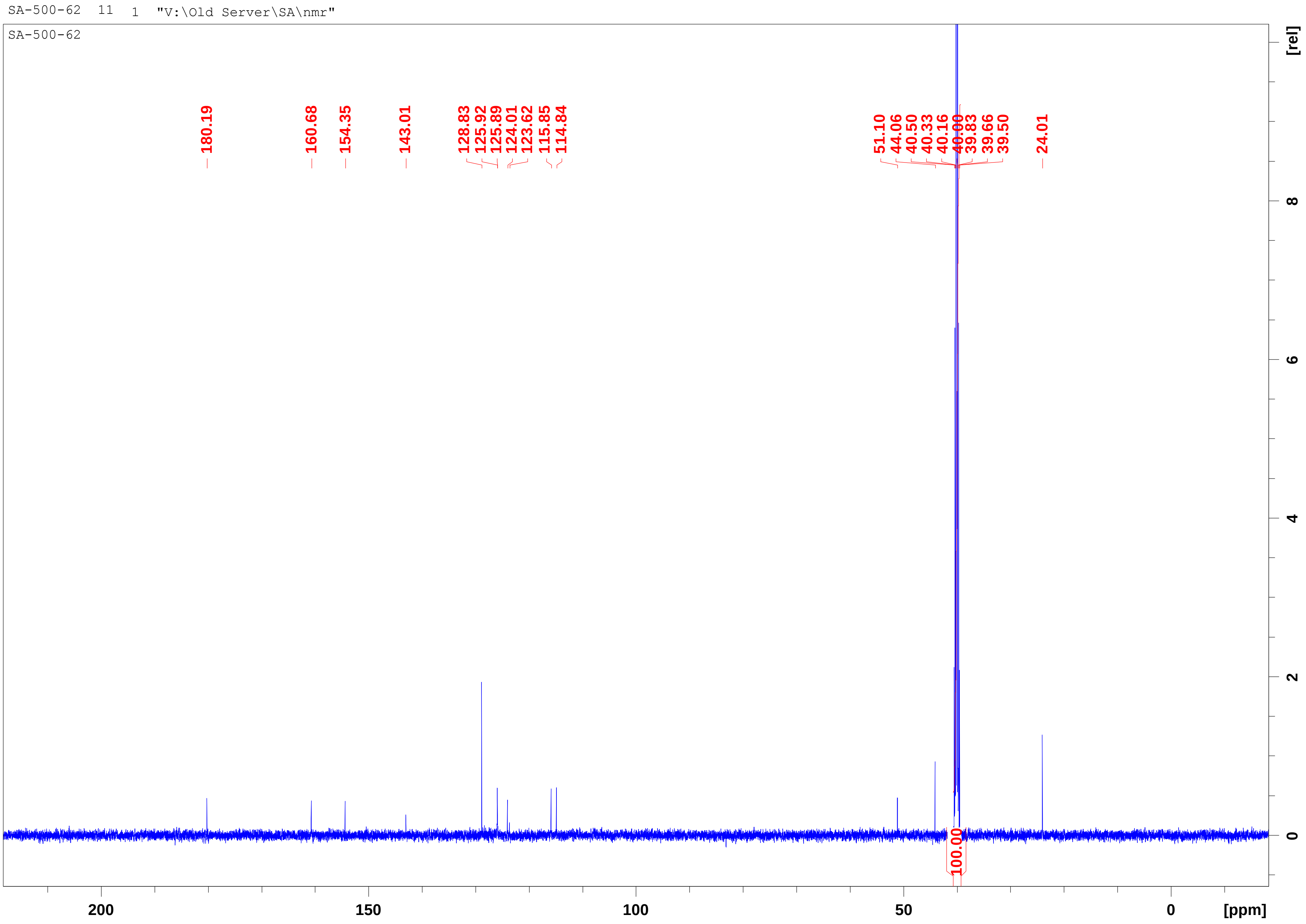
SA-500-62 11 1 "V:\Old Server\SA\nmr"
SA-500-62
180.19
160.68
154.35
143.01
128.83
125.92
125.89
124.01
123.62
115.85
114.84
51.10
44.06
40.50
40.33
40.16
40.00
39.83
39.66
39.50
24.01
100.00
[rel]
8
6
4
2
0
200
150
100
50
0
[ppm]

15n

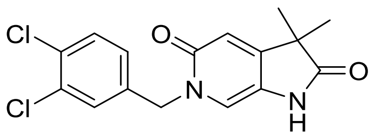

SA-500-78 10 1 "V:\Old Server\SA\nmr"

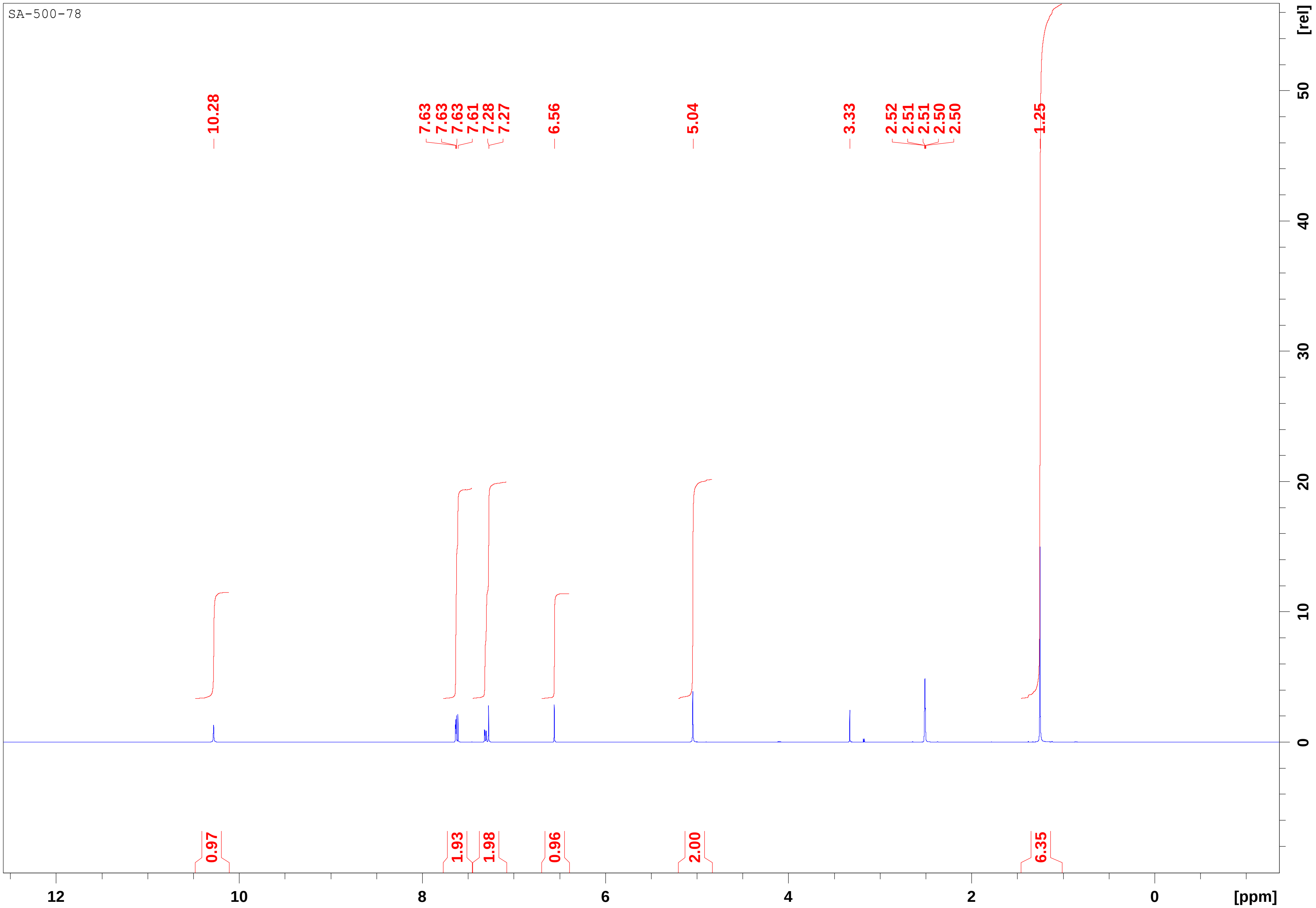

15n

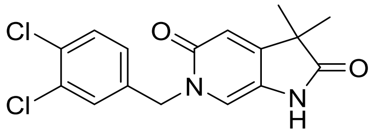

SA-500-78 11 1 "V:\Old Server\SA\nmr"

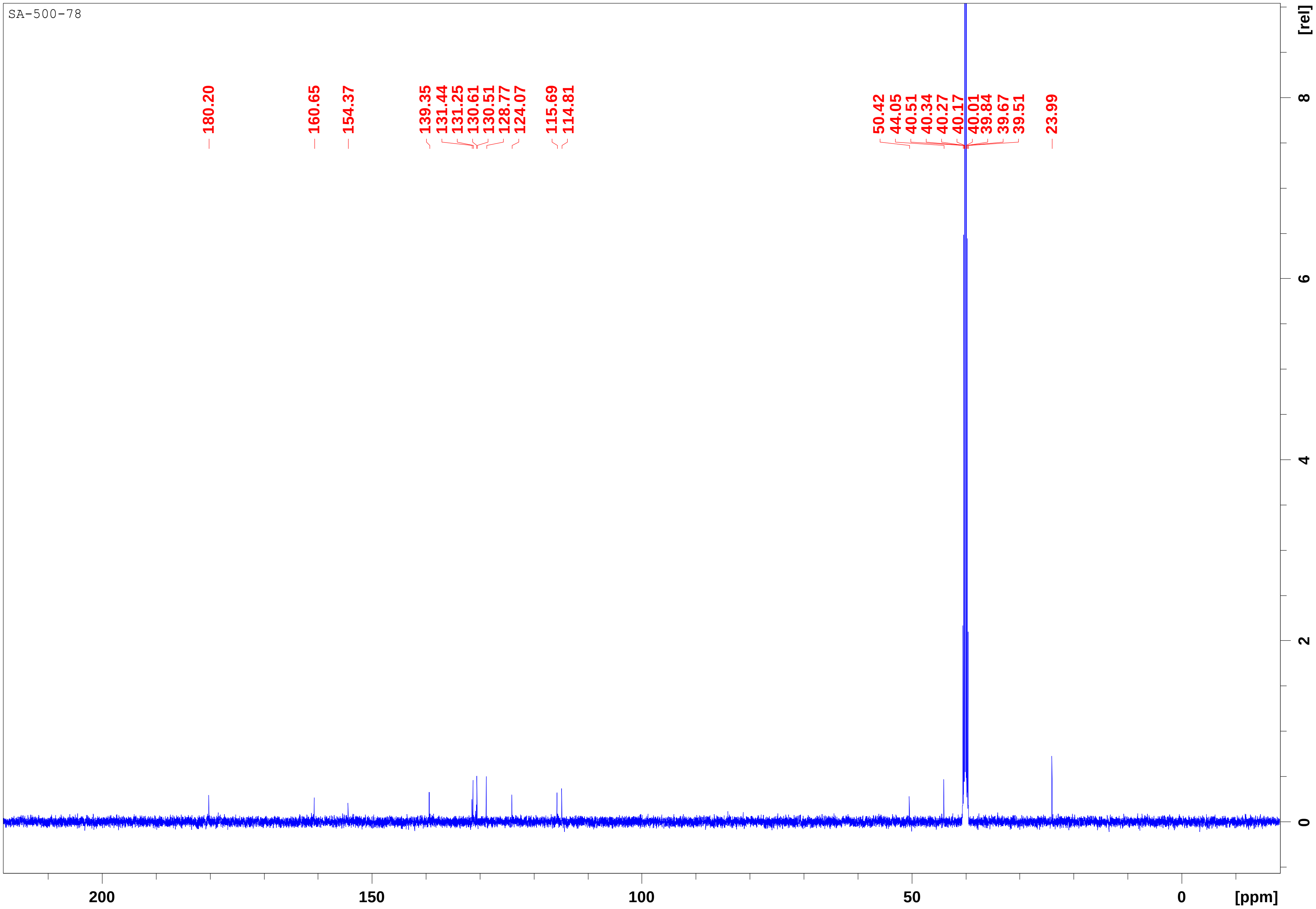

15o

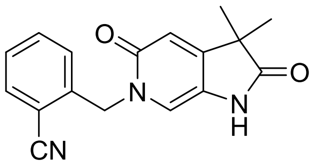

SA-500-22 10 1 "V:\Old Server\SA\nmr"

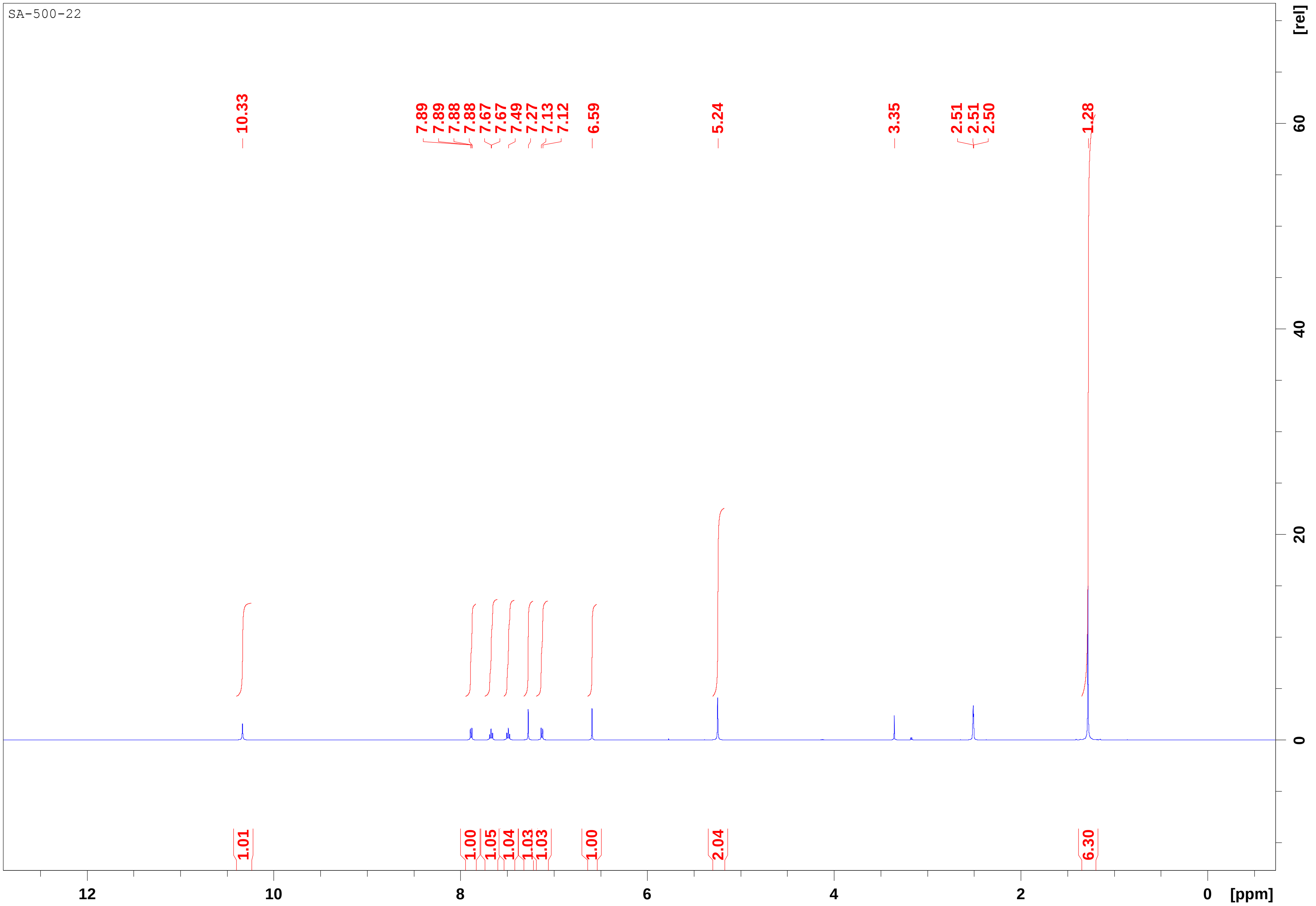

15o

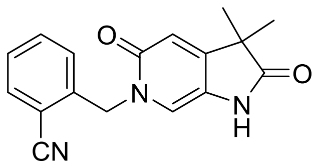

SA-500-22 11 1 "V:\Old Server\SA\nmr"

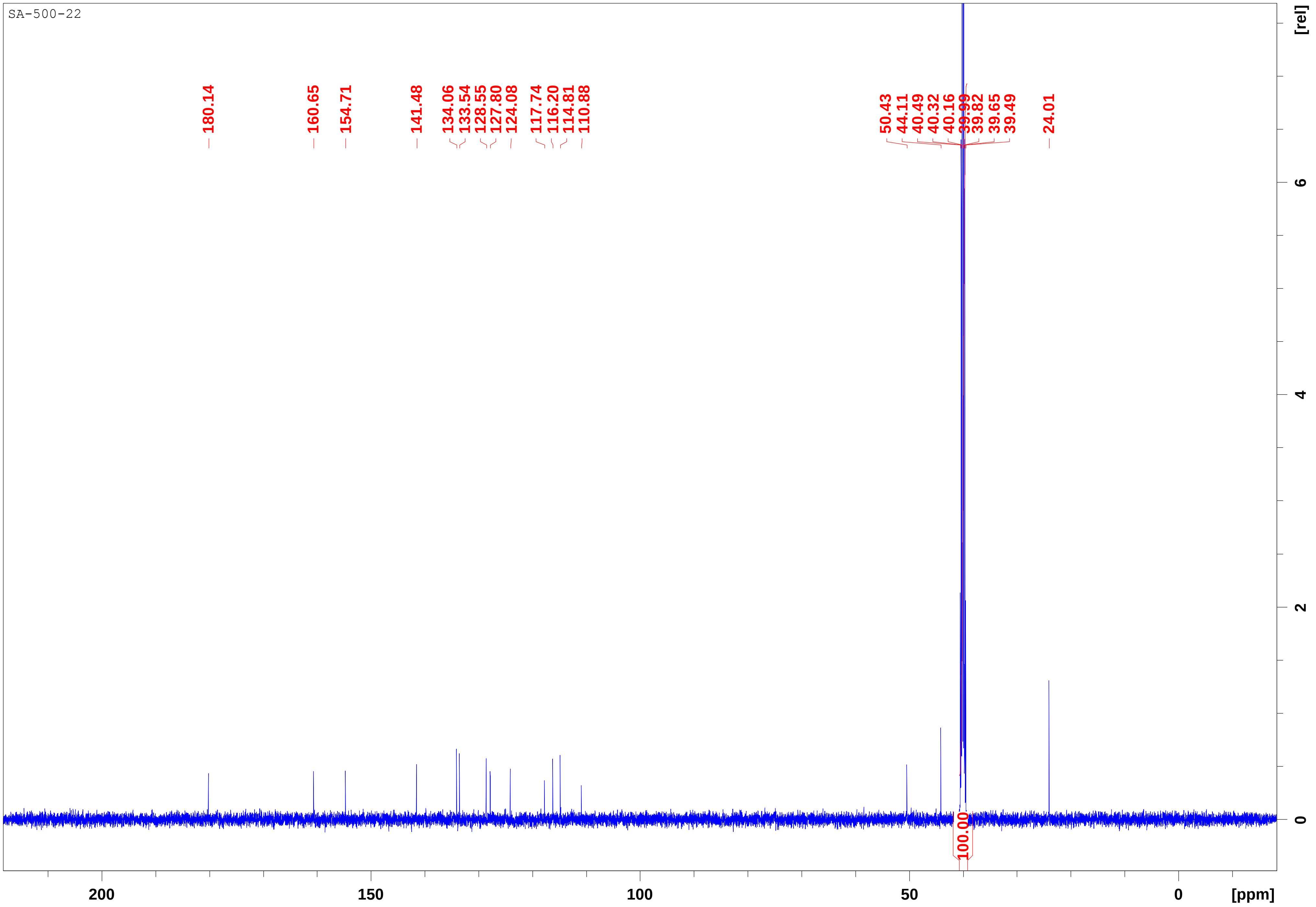

15p

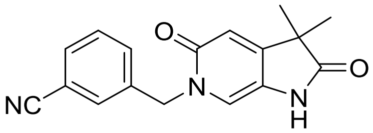

SA-500-28 10 1 "V:\Old Server\SA\nmr"

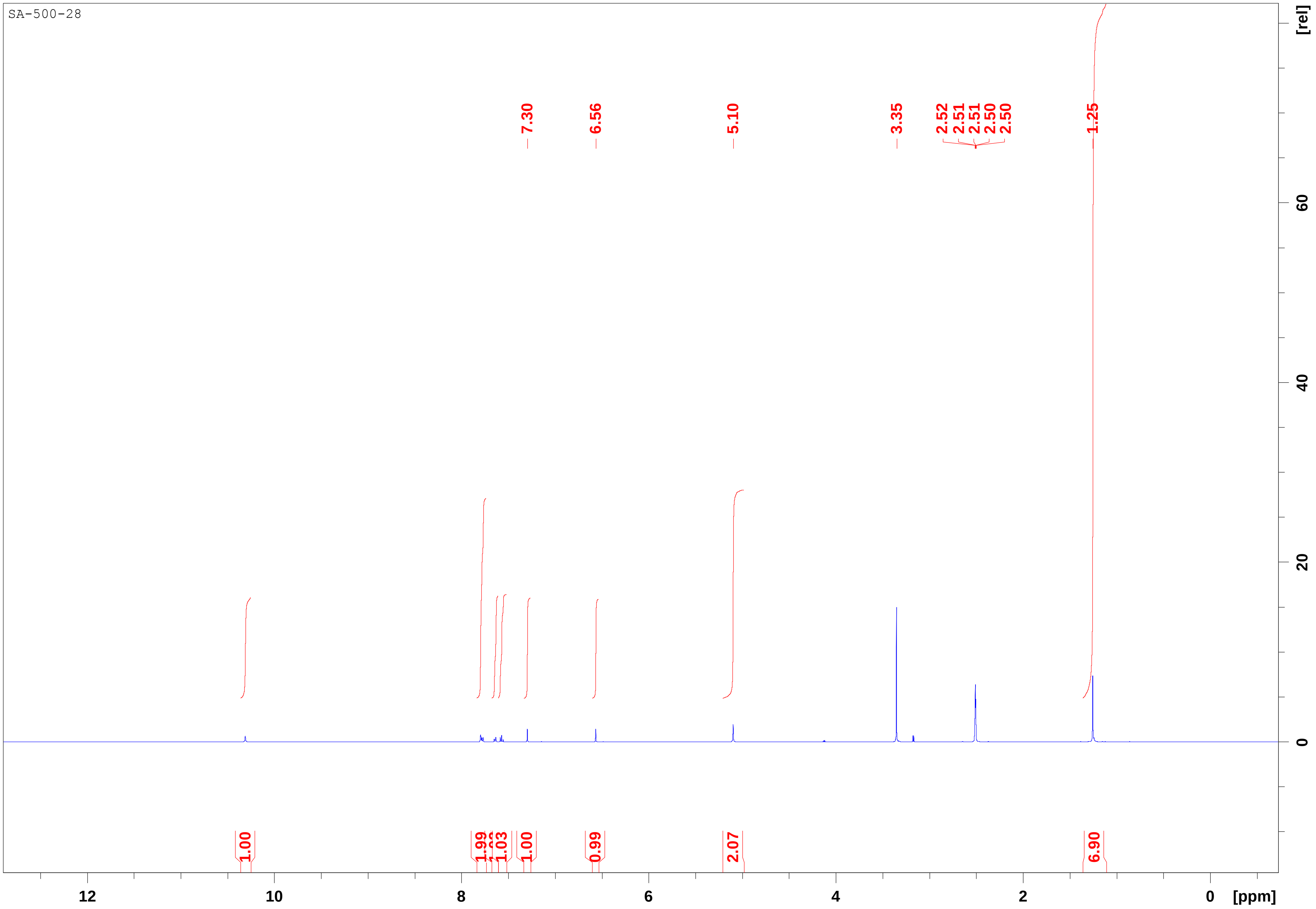

15p

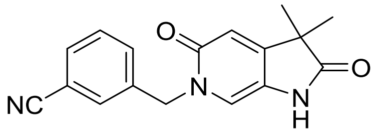

SA-500-28 15 1 "V:\Old Server\SA\nmr"

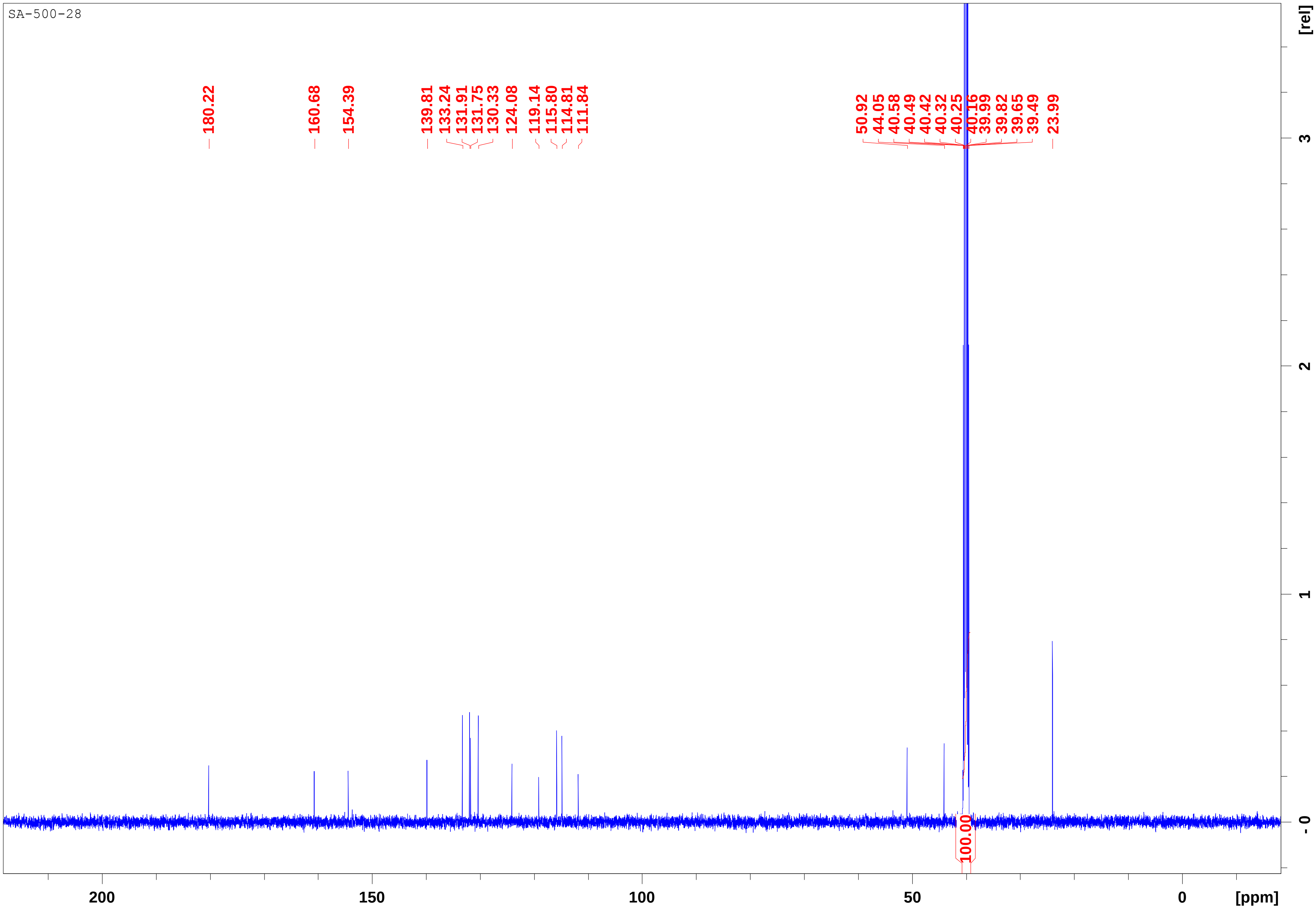

16a

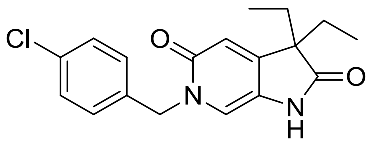
Cl
O
N
O
N
H

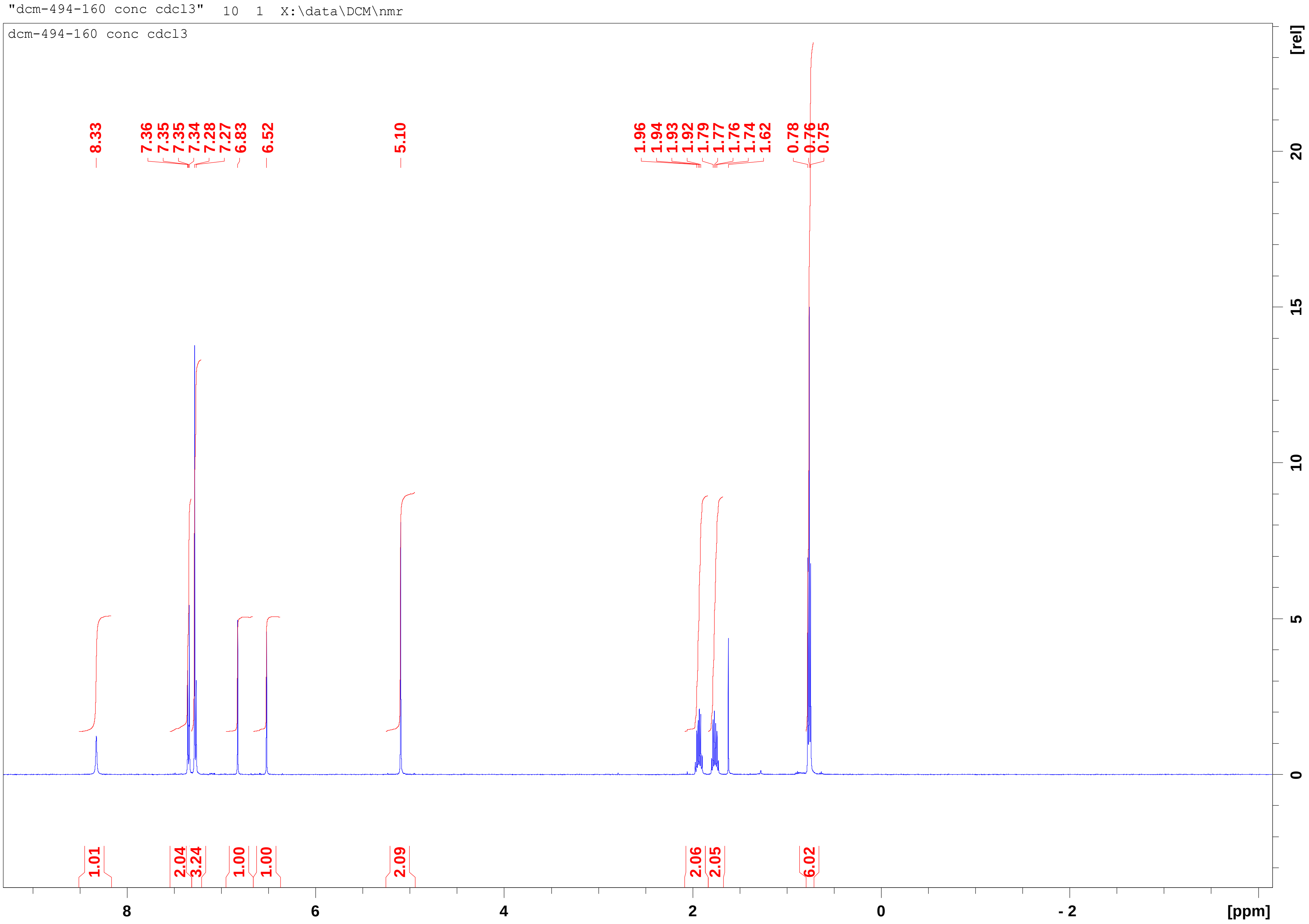
"dcm-494-160 conc cdcl3" 10 1 X:\data\DCM\nmr
dcm-494-160 conc cdcl3
8.33
7.36
7.35
7.35
7.34
7.28
7.27
6.83
6.52
5.10
1.96
1.94
1.93
1.92
1.79
1.77
1.76
1.74
1.62
0.78
0.76
0.75
1.01
2.04
3.24
1.00
1.00
2.09
2.06
2.05
6.02
8
6
4
2
0
- 2
[ppm]
[rel]
20
15
10
5
0

16a

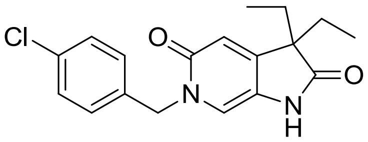
Cl
O
N
N
H
O

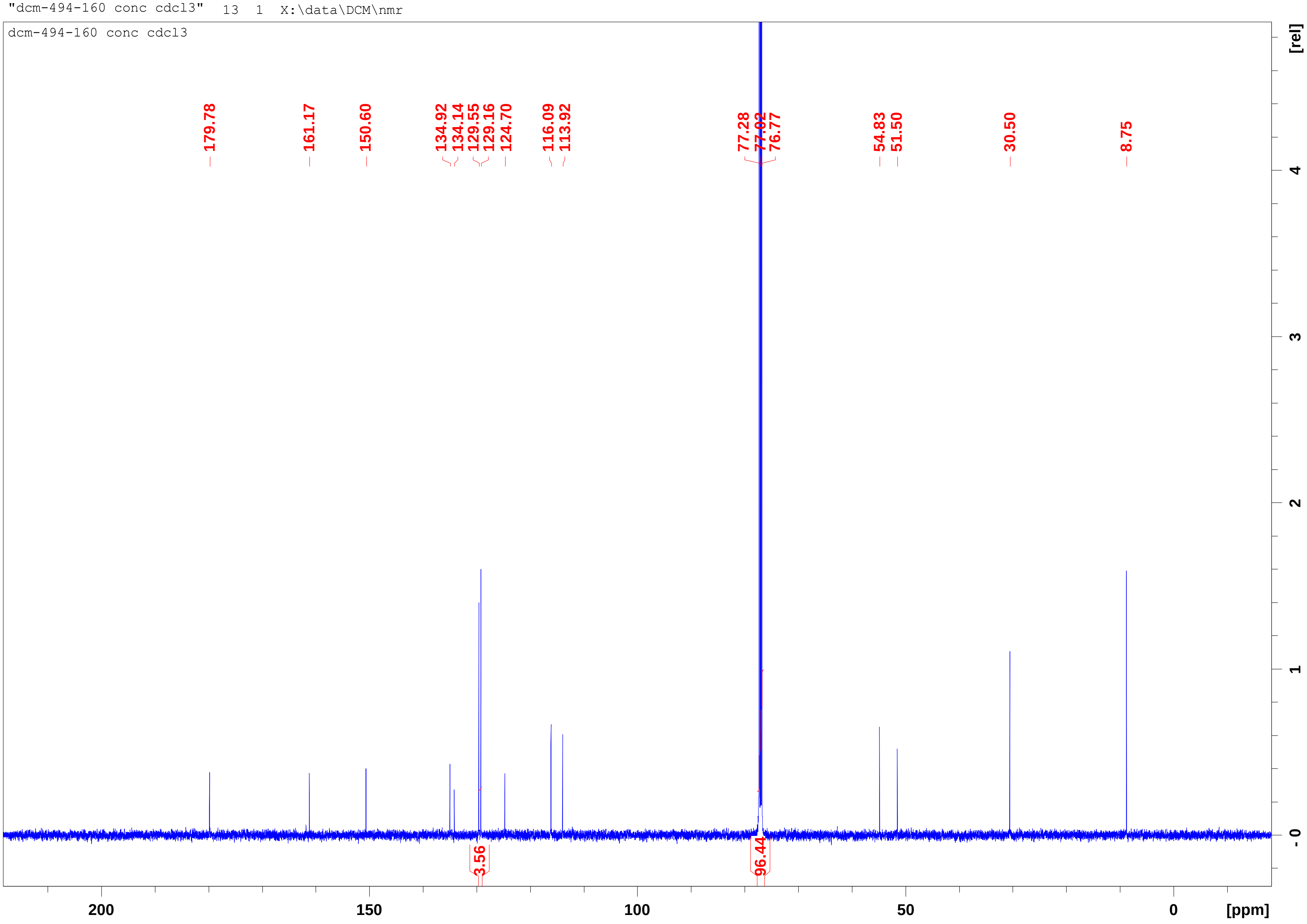
"dcm-494-160 conc cdcl3" 13 1 X:\data\DCM\nmr
dcm-494-160 conc cdcl3
179.78
161.17
150.60
134.92
134.14
129.55
129.16
124.70
116.09
113.92
77.28
77.02
76.77
54.83
51.50
30.50
8.75
3.56
96.44
[rel]
[ppm]

16b

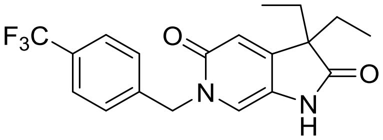

"DCM-494-180-A CDCL3 CONC" 10 1 X:\data\DCM\nmr

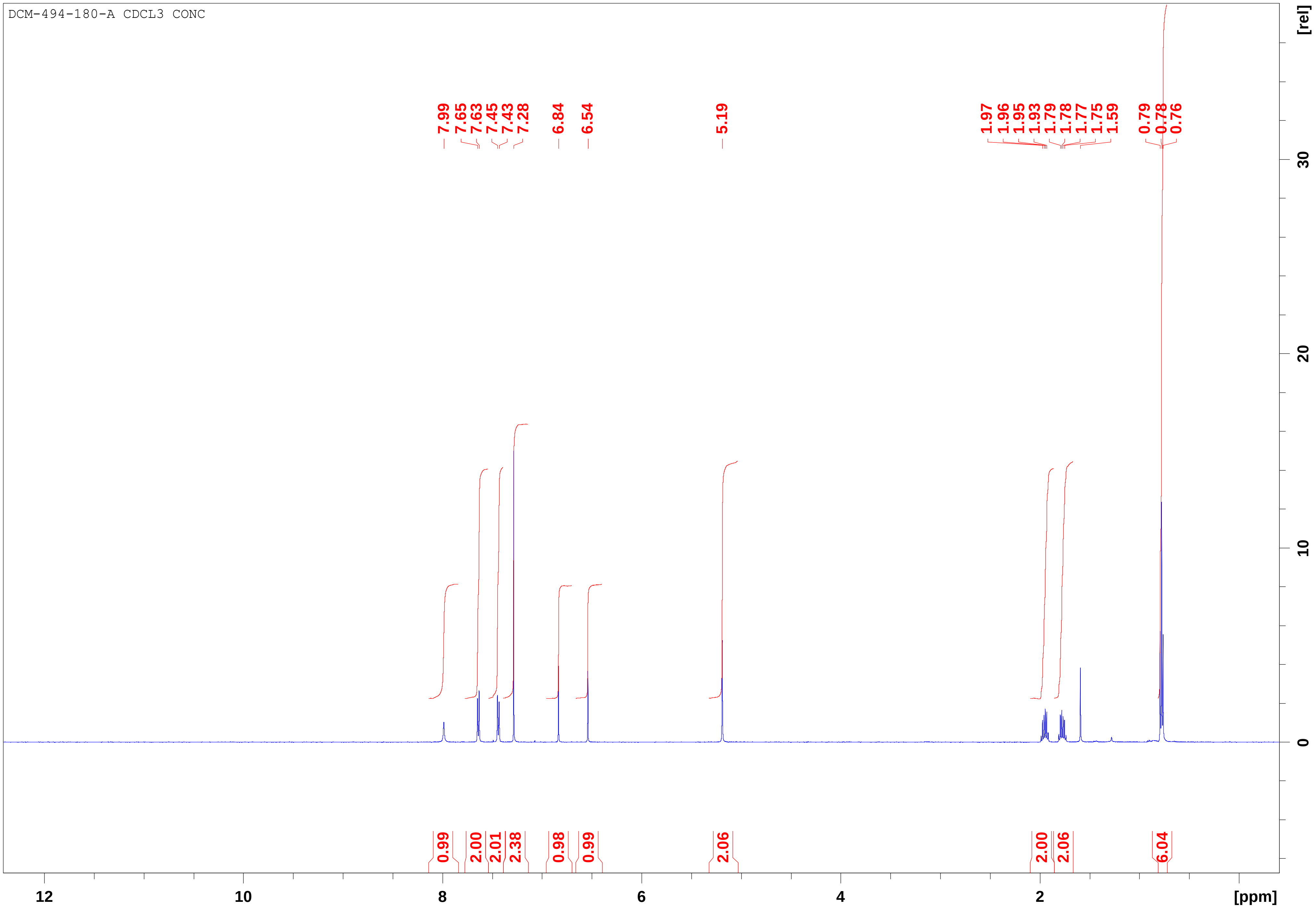

16b

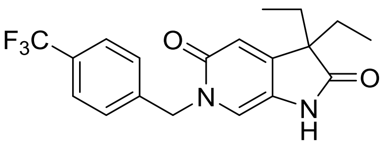

"DCM-494-180-A CDCL3 CONC" 16 1 X:\data\DCM\nmr

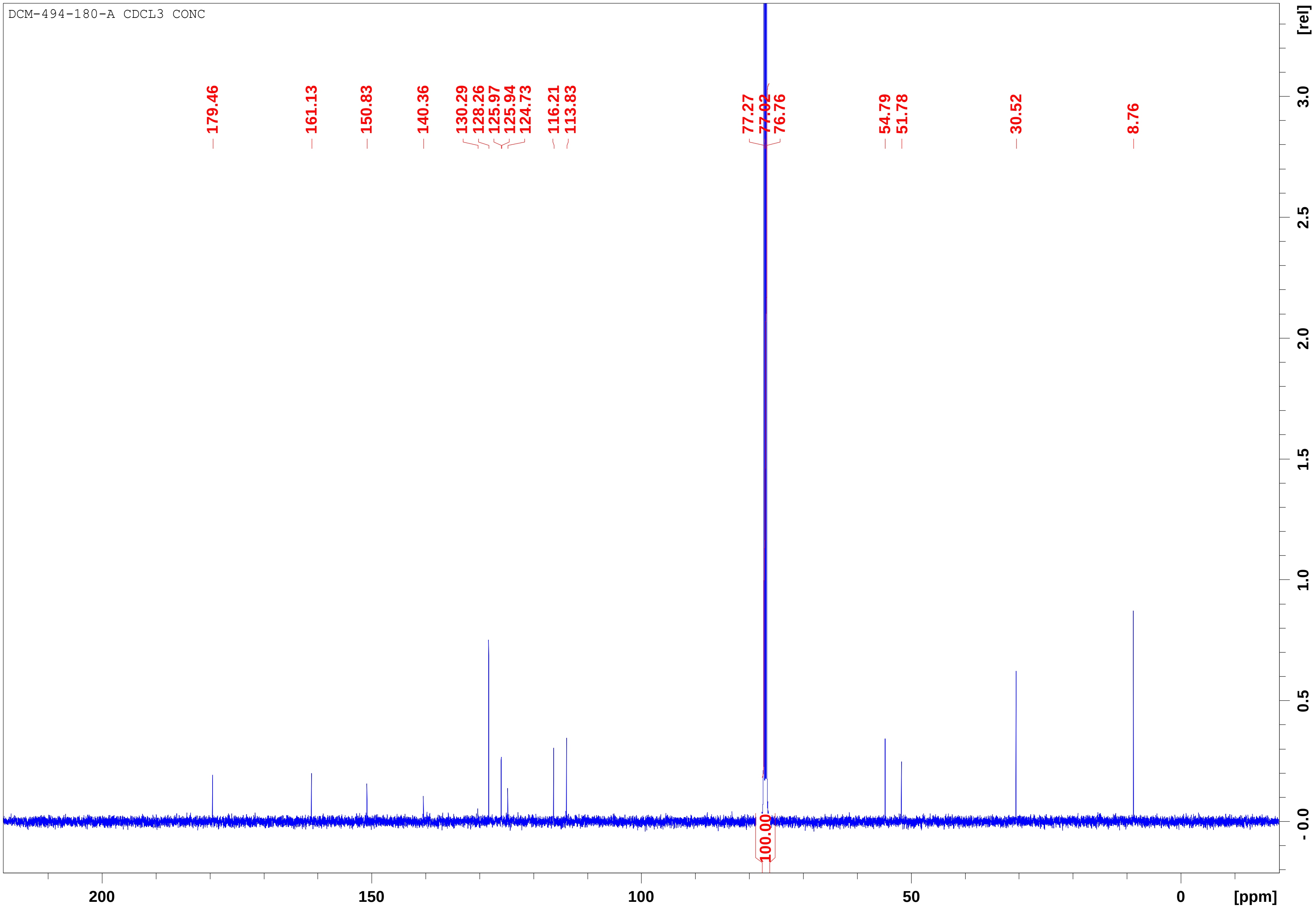

CD3OD

17a

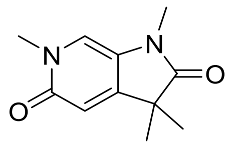

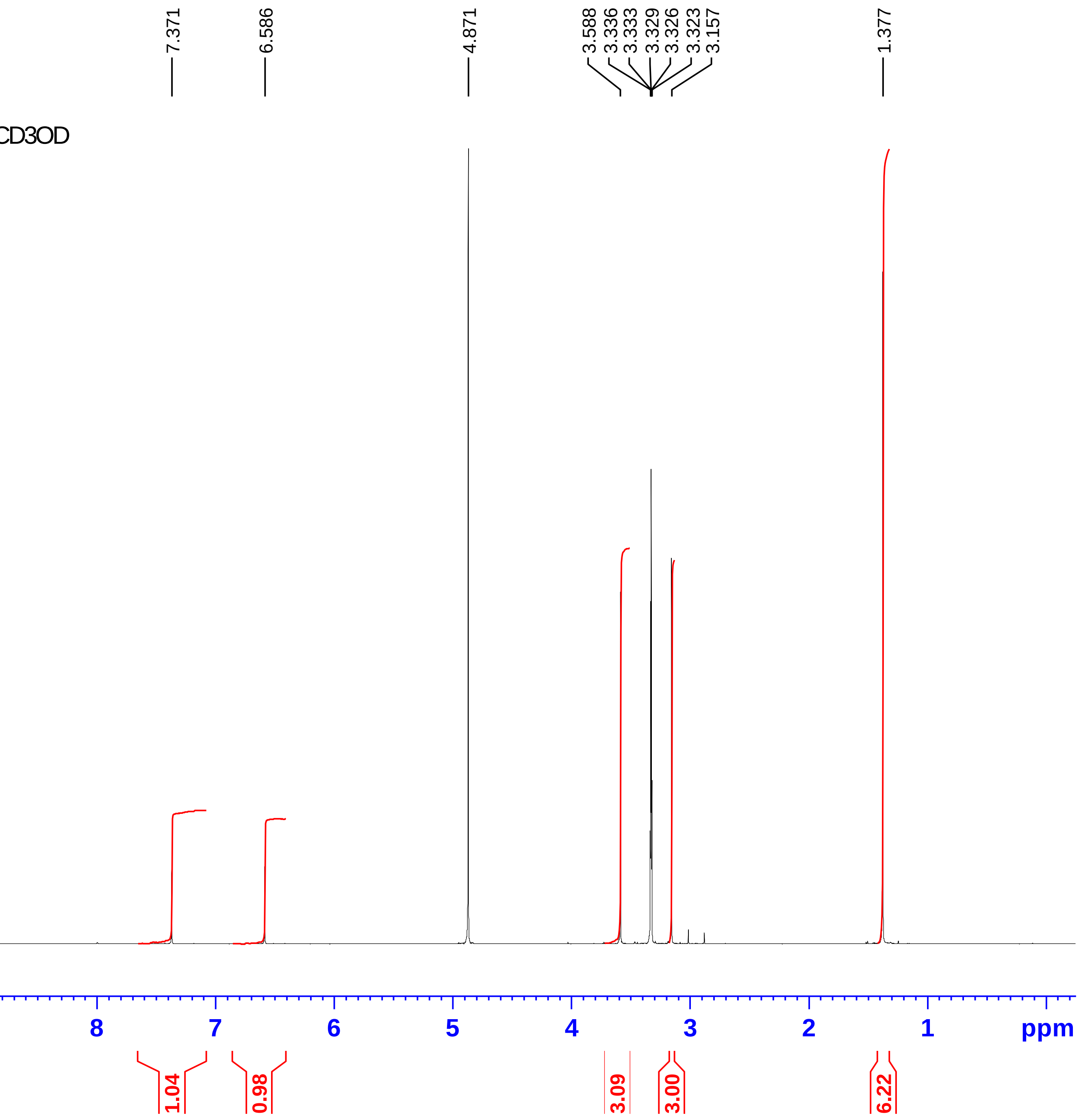

Current Data Parameters
NAME DCM-494-23-A CD3OD
EXPNO 10
PROCNO 1

F2 - Acquisition Parameters
Date_ 20140908
Time 13.35
INSTRUM spect
PROBHD 5 mm PABBO BB-
PULPROG zg30
TD 65536
SOLVENT MeOD
NS 16
DS 0
SWH 10330.578 Hz
FIDRES 0.157632 Hz
AQ 3.1719425 sec
RG 575
DW 48.400 usec
DE 11.99 usec
TE 297.9 K
D1 1.00000000 sec
TD0 1

======== CHANNEL f1 ========
SFO1 500.3030896 MHz
NUC1 1H
P1 16.25 usec
PLW1 18.33600044 W

F2 - Processing parameters SI 65536
SF 500.3000000 MHz
WDW EM
SSB 0
LB 0.30 Hz
GB 0
PC 1.00

CD3OD

17a

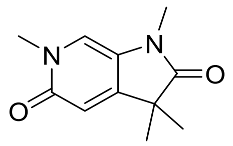

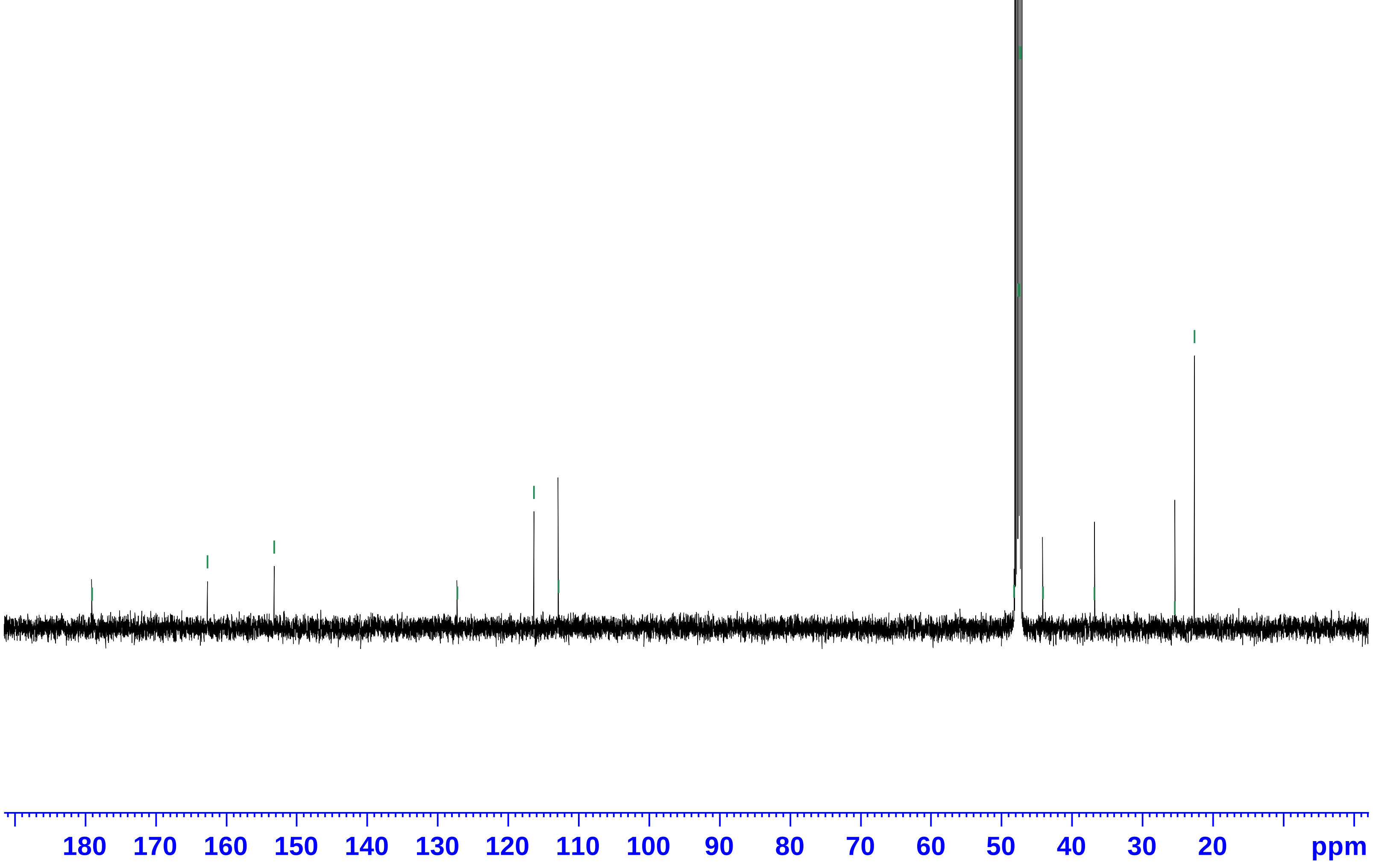

Current Data Parameters
NAME DCM-494-23-A CD3OD
EXPNO 11
PROCNO 1

F2 - Acquisition Parameters
Date_ 20140909
Time 2.00
INSTRUM spect
PROBHD 5 mm PABBO BB-
PULPROG zgpg30
TD 65536
SOLVENT MeOD
NS 2048
DS 2
SWH 29761.904 Hz
FIDRES 0.454131 Hz
AQ 1.1010048 sec
RG 456
DW 16.800 usec
DE 7.68 usec
TE 298.0 K
D1 2.00000000 sec
D11 0.03000000 sec
TD0 1

======== CHANNEL f1 ========

SFO1 125.8131151 MHz
NUC1 13C
P1 9.75 usec
PLW1 82.38999939 W

======== CHANNEL f2 ========

SFO2 500.3020012 MHz
NUC2 1H
CPDPRG[2 waltz16
PCPD2 80.00 usec
PLW2 18.33600044 W
PLW12 0.75654000 W
PLW13 0.48418999 W

F2 - Processing Parameters
SI 65536
SF 125.8005350 MHz
WDW EM
SSB 0
LB 1.00 Hz
GB 0
PC 1.40

17b CDCL3

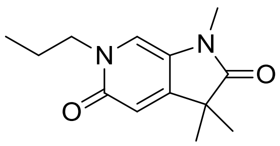

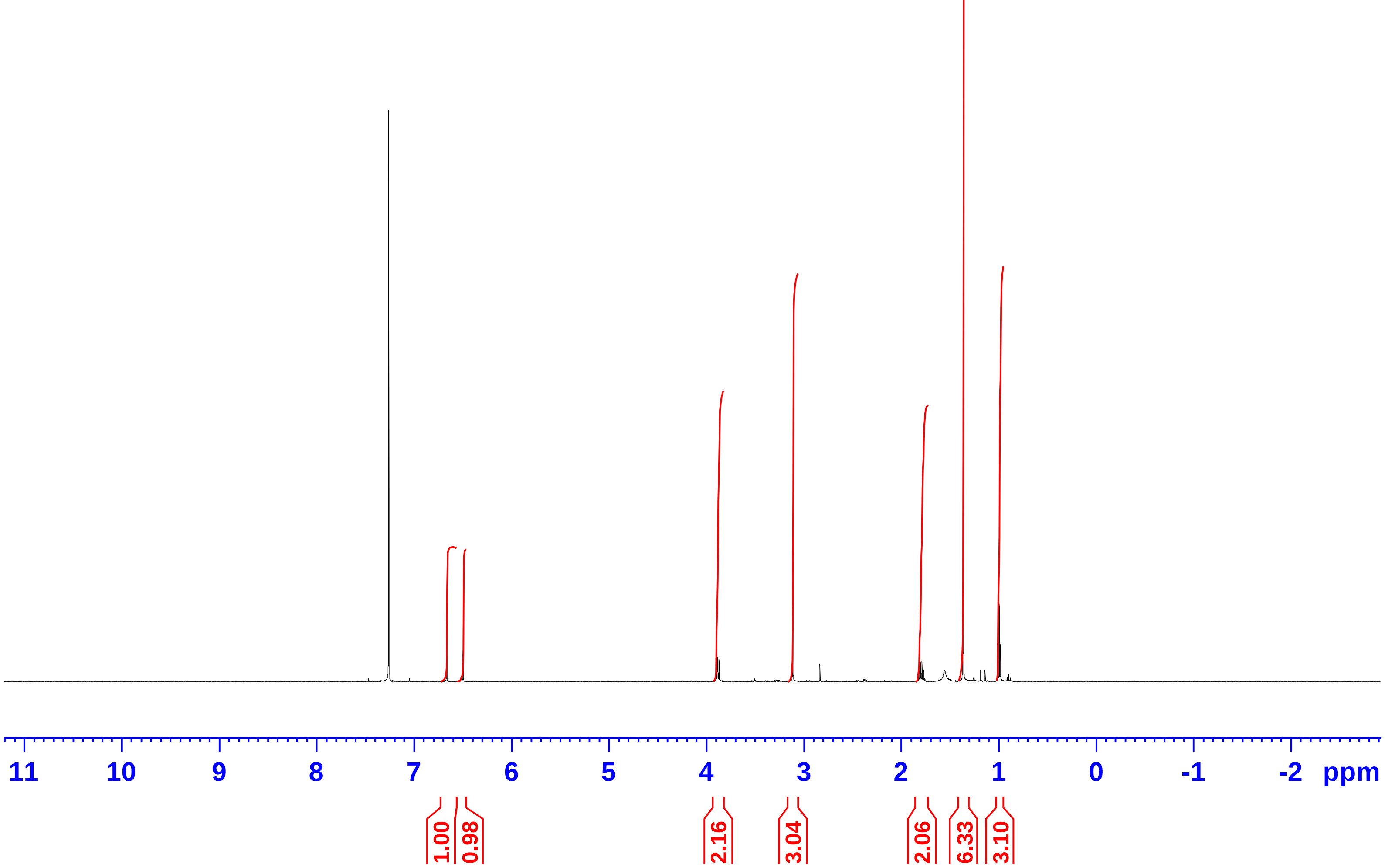

Current Data Parameters
NAME DCM-494-115-A CDCL3
EXPNO 10
PROCNO 1

F2 - Acquisition Parameters
Date_ 20141126
Time 9.59
INSTRUM spect
PROBHD 5 mm PABBO BB-
PULPROG zg30
TD 65536
SOLVENT CDCl3
NS 16
DS 0
SWH 10330.578 Hz
FIDRES 0.157632 Hz
AQ 3.1719425 sec
RG 812
DW 48.400 usec
DE 11.99 usec
TE 298.0 K
D1 1.00000000 sec
TD0 1

======== CHANNEL f1 ========
SFO1 500.3030896 MHz
NUC1 1H
P1 16.25 usec
PLW1 18.33600044 W

F2 - Processing parameters SI 65536
SF 500.3000123 MHz
WDW EM
SSB 0
LB 0.30 Hz
GB 0
PC 1.00

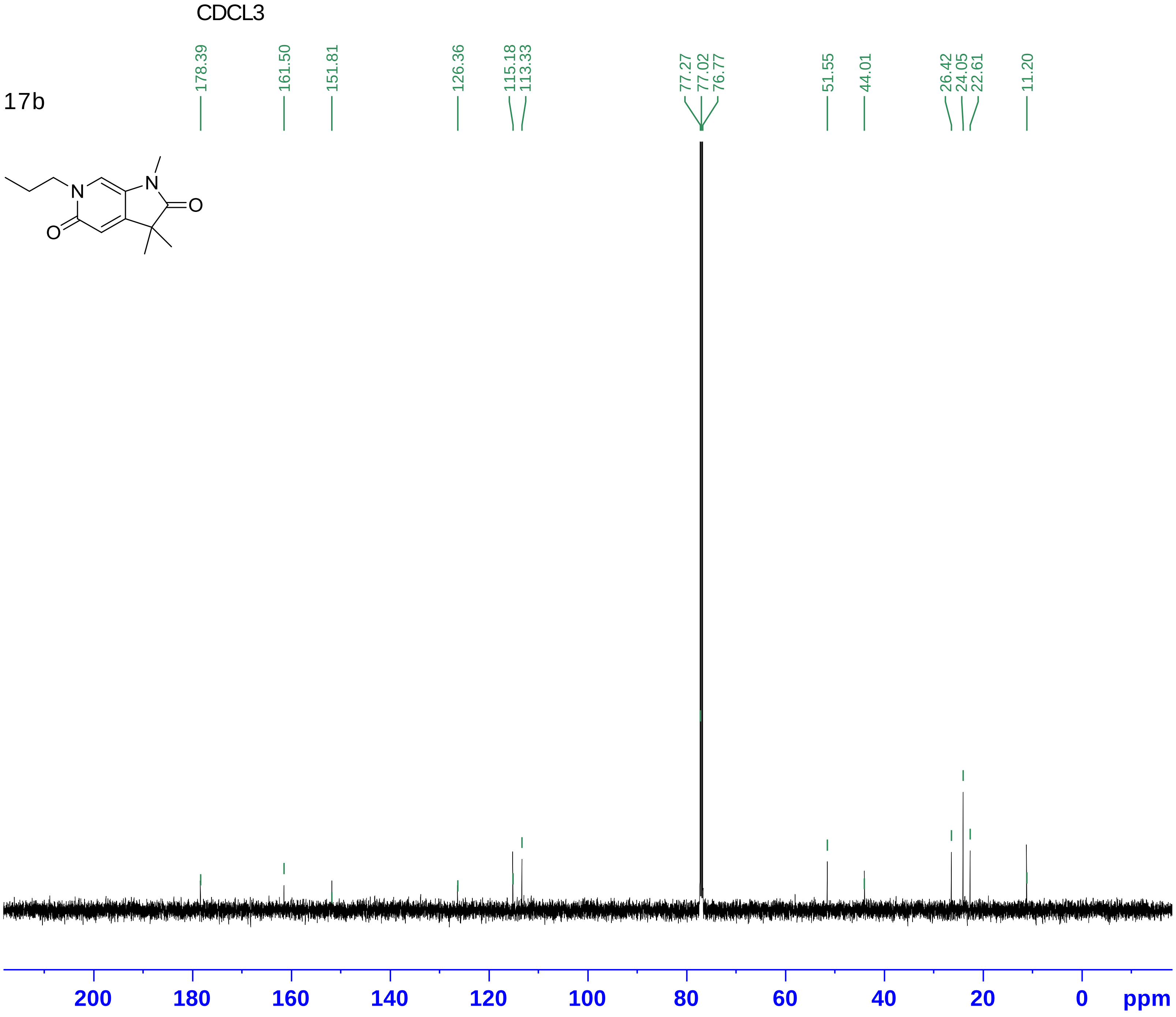

Current Data Parameters
NAME DCM-494-114-A CDCL3
EXPNO 11
PROCNO 1

F2 - Acquisition Parameters
Date_ 20141209
Time 12.25
INSTRUM spect
PROBHD 5 mm PABBO BB-
PULPROG zgpg30
TD 65536
SOLVENT CDCl3
NS 256
DS 2
SWH 29761.904 Hz
FIDRES 0.454131 Hz
AQ 1.1010048 sec
RG 575
DW 16.800 usec
DE 7.68 usec
TE 298.0 K
D1 2.00000000 sec
D11 0.03000000 sec
TD0 1

======== CHANNEL f1 ========

SFO1 125.8131151 MHz
NUC1 13C
P1 9.75 usec
PLW1 82.38999939 W

======== CHANNEL f2 ========
SFO2 500.3020012 MHz
NUC2 1H
CPDPRG[2 waltz16
PCPD2 80.00 usec
PLW2 18.33600044 W
PLW12 0.75654000 W
PLW13 0.48418999 W

F2 - Processing Parameters
SI 65536
SF 125.8005350 MHz
WDW EM
SSB 0
LB 1.00 Hz
GB 0
PC 1.40

17c CDCL3

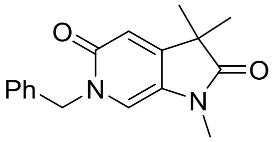

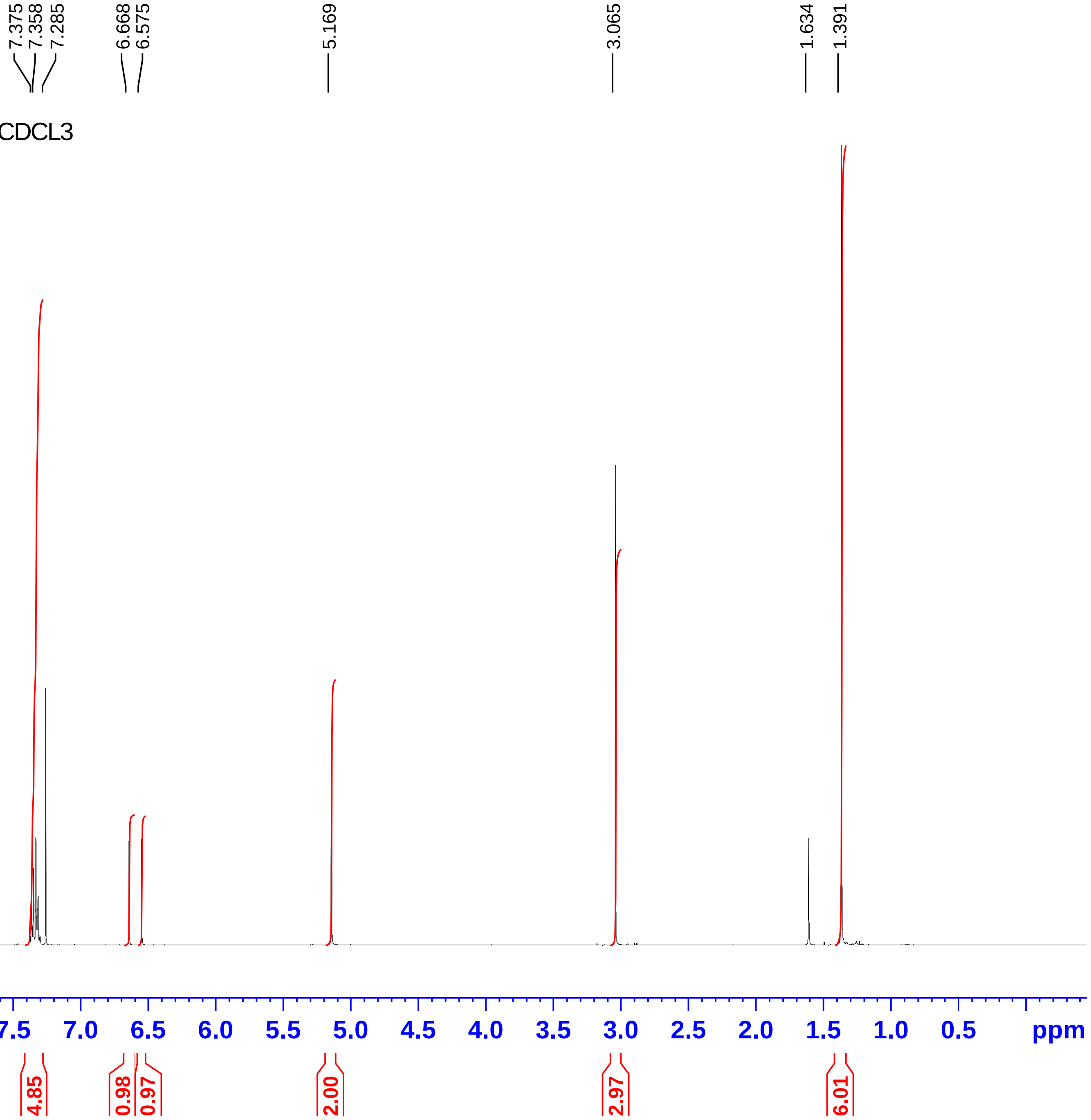

Current Data Parameters
NAME DCM-494-75-A CDCL3 CONC
EXPNO 11
PROCNO 1

F2 - Acquisition Parameters
Date_ 20141029
Time 8.38
INSTRUM spect
PROBHD 5 mm PABBO BB-
PULPROG zg30
TD 65536
SOLVENT CDCl3
NS 16
DS 0
SWH 10330.578 Hz
FIDRES 0.157632 Hz
AQ 3.1719425 sec
RG 575
DW 48.400 usec
DE 11.99 usec
TE 298.1 K
D1 1.00000000 sec
TD0 1

======== CHANNEL f1 ========
SFO1 500.3030896 MHz
NUC1 1H
P1 16.25 usec
PLW1 18.33600044 W

F2 - Processing Parameters
SI 65536
SF 500.3000125 MHz
WDW EM
SSB 0
LB 0.30 Hz
GB 0
PC 1.00

CDCL3

17c

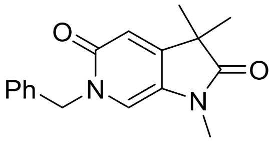

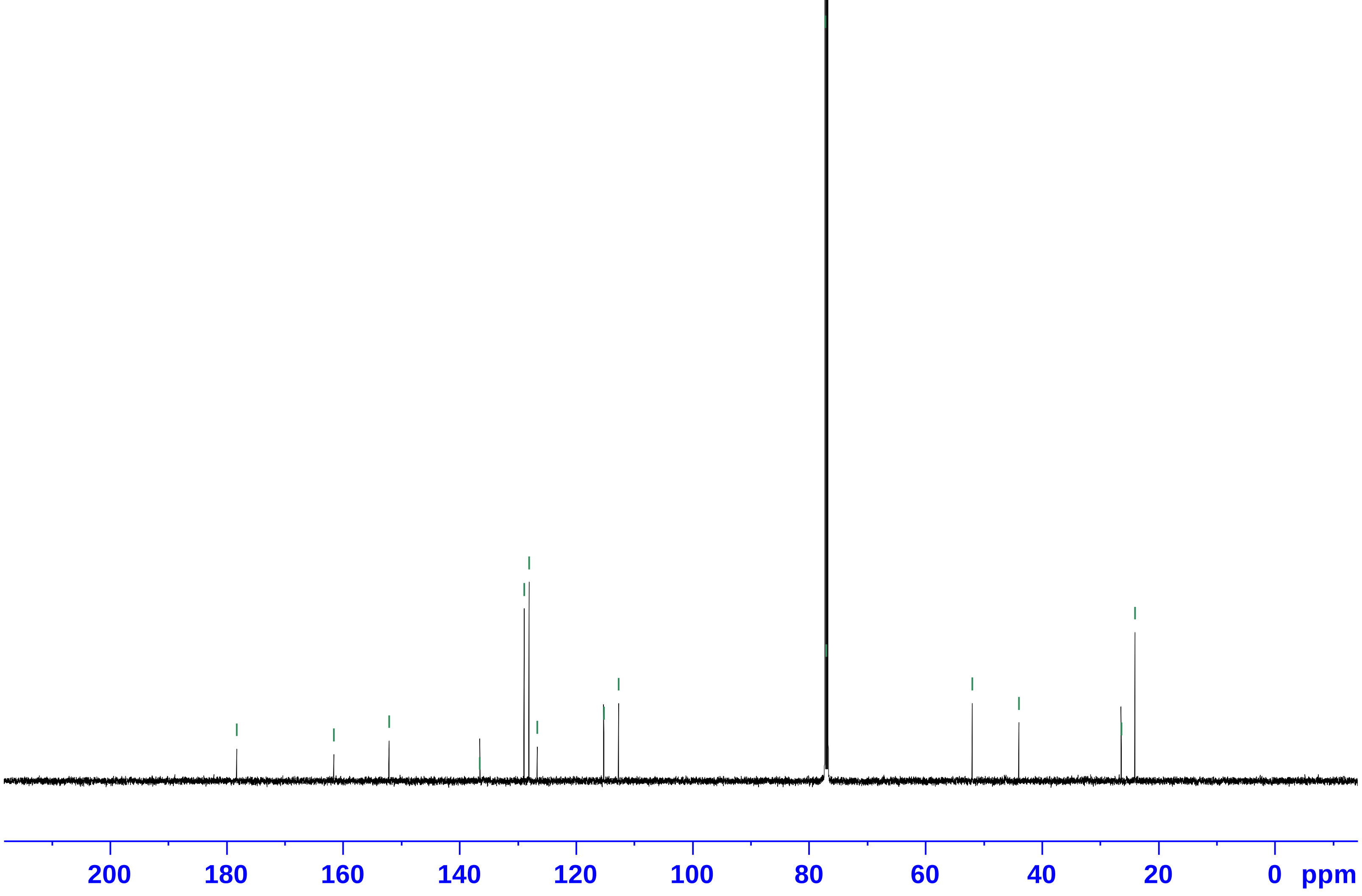

Current Data Parameters
NAME DCM-494-75-A CDCL3 CONC
EXPNO 10
PROCNO 1

F2 - Acquisition Parameters
Date_ 20141029
Time 6.35
INSTRUM spect
PROBHD 5 mm PABBO BB-
PULPROG zgpg30
TD 65536
SOLVENT CDCl3
NS 2048
DS 2
SWH 29761.904 Hz
FIDRES 0.454131 Hz
AQ 1.1010048 sec
RG 575
DW 16.800 usec
DE 7.68 usec
TE 298.0 K
D1 2.00000000 sec
D11 0.03000000 sec
TD0 1

======== CHANNEL f1 ========
SFO1 125.8131151 MHz
NUC1 13C
P1 9.75 usec
PLW1 82.38999939 W

======== CHANNEL f2 ========
SFO2 500.3020012 MHz
NUC2 1H
CPDPRG[2 waltz16
PCPD2 80.00 usec
PLW2 18.33600044 W
PLW12 0.75654000 W
PLW13 0.48418999 W

F2 - Processing parameters
SI 65536
SF 125.8005350 MHz
WDW EM
SSB 0
LB 1.00 Hz
GB 0
PC 1.40

17d CDCL3

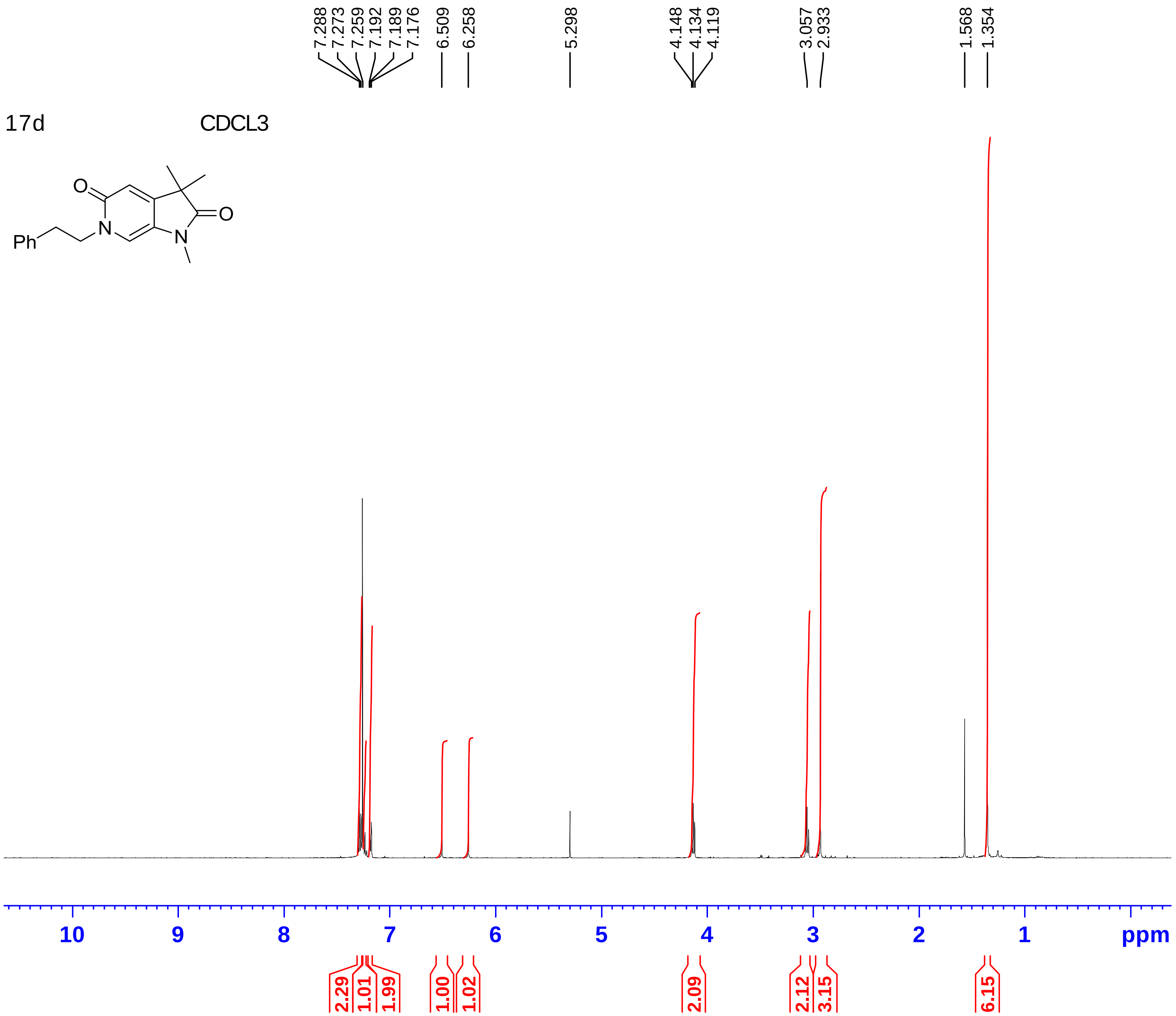

Current Data Parameters
NAME DCM-494-103-A CDCL3
EXPNO 10
PROCNO 1

F2 - Acquisition Parameters
Date_ 20141119
Time 16.56
INSTRUM spect
PROBHD 5 mm PABBO BB-
PULPROG zg30
TD 65536
SOLVENT CDCl3
NS 16
DS 0
SWH 10330.578 Hz
FIDRES 0.157632 Hz
AQ 3.1719425 sec
RG 645
DW 48.400 usec
DE 11.99 usec
TE 298.0 K
D1 1.00000000 sec
TD0 1

======== CHANNEL f1 ========
SFO1 500.3030896 MHz
NUC1 1H
P1 16.25 usec
PLW1 18.33600044 W

F2 - Processing parameters SI 65536
SF 500.3000126 MHz
WDW EM
SSB 0
LB 0.30 Hz
GB 0
PC 1.00

17d

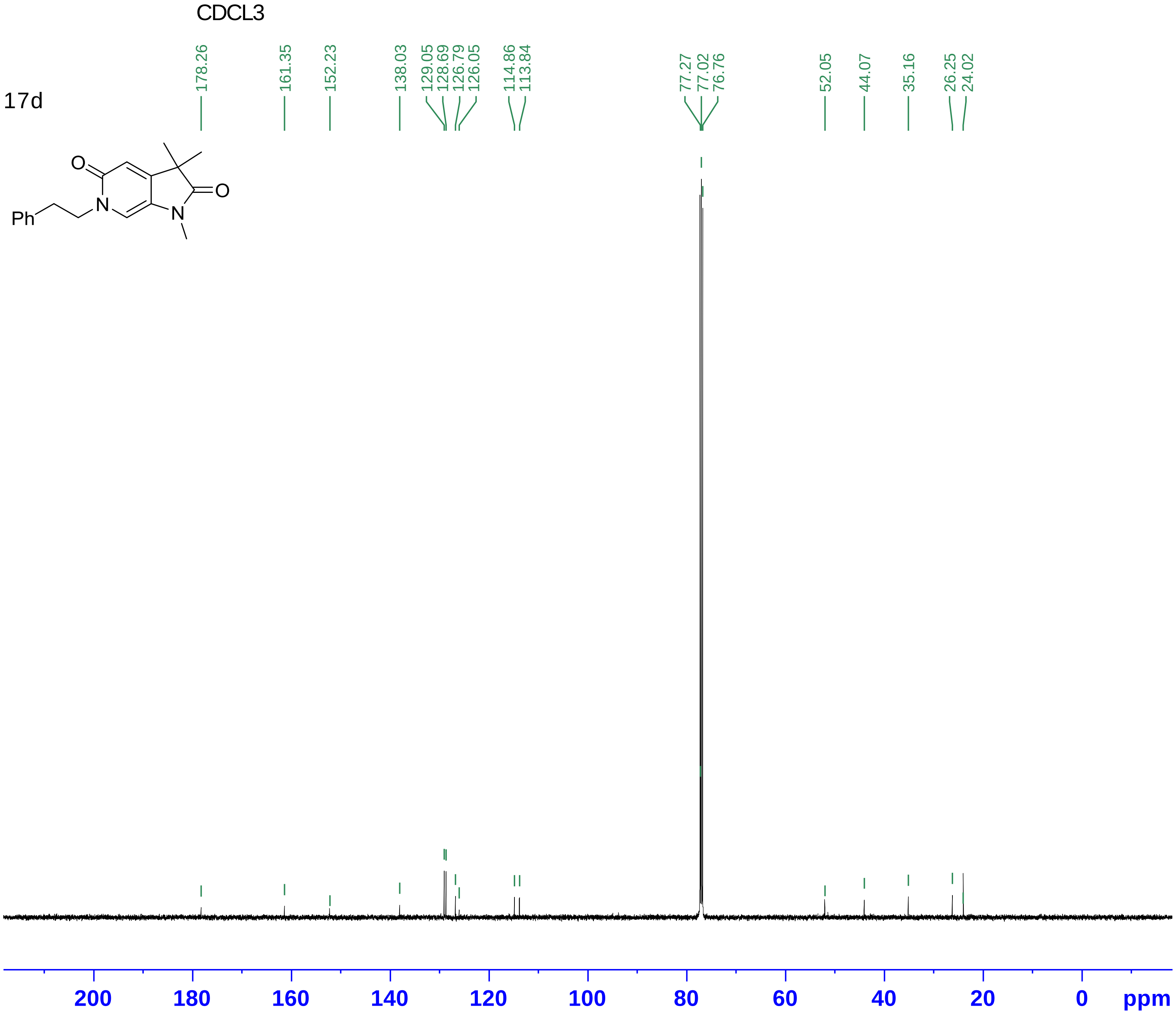

Current Data Parameters
NAME DCM-494-103-A CDCL3
EXPNO 14
PROCNO 1

F2 - Acquisition Parameters
Date_ 20141121
Time 1.04
INSTRUM spect
PROBHD 5 mm PABBO BB-
PULPROG zgpg30
TD 65536
SOLVENT CDCl3
NS 2048
DS 2
SWH 29761.904 Hz
FIDRES 0.454131 Hz
AQ 1.1010048 sec
RG 456
DW 16.800 usec
DE 7.68 usec
TE 298.5 K
D1 2.00000000 sec
D11 0.03000000 sec
TD0 1

======== CHANNEL f1 ========

SFO1 125.8131151 MHz
NUC1 13C
P1 9.75 usec
PLW1 82.38999939 W

======== CHANNEL f2 ========
SFO2 500.3020012 MHz
NUC2 1H
CPDPRG[2 waltz16
PCPD2 80.00 usec
PLW2 18.33600044 W
PLW12 0.75654000 W
PLW13 0.48418999 W

F2 - Processing parameters
SI 65536
SF 125.8005350 MHz
WDW EM
SSB 0
LB 1.00 Hz
GB 0
PC 1.40

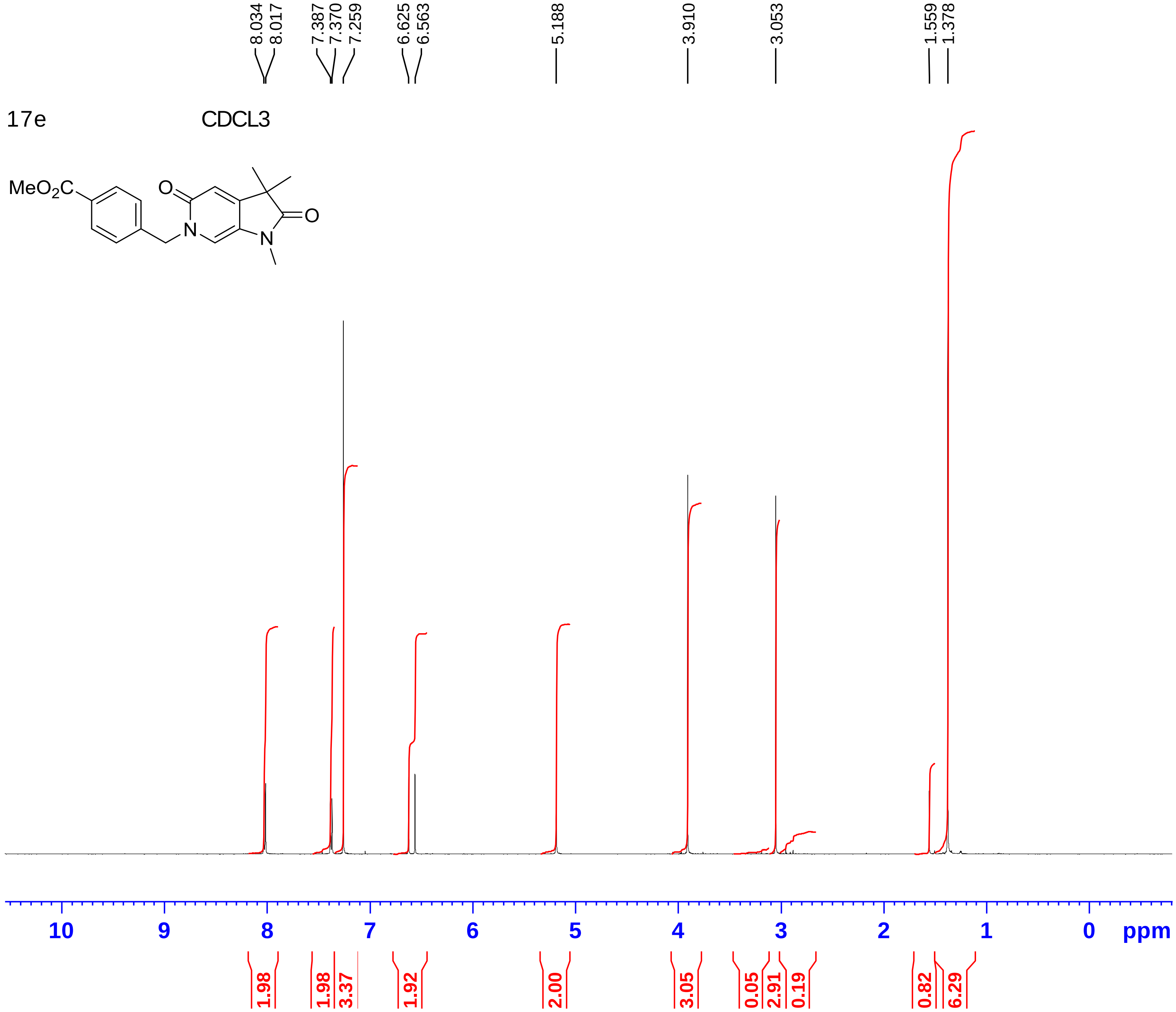

Current Data Parameters

NAME	DCM-494-117-A CDCL3
EXPNO	10
PROCNO	1

F2 - Acquisition Parameters

Date_	20141204
Time	9.31
INSTRUM	spect
PROBHD	5 mm PABBO BB-
PULPROG	zg30
TD	65536
SOLVENT	CDCl3
NS	16
DS	0
SWH	10330.578 Hz
FIDRES	0.157632 Hz
AQ	3.1719425 sec
RG	724
DW	48.400 usec
DE	11.99 usec
TE	298.0 K
D1	1.00000000 sec
TD0	1

======== CHANNEL f1 ========

SFO1	500.3030896 MHz
NUC1	1H
P1	16.25 usec
PLW1	18.33600044 W

F2 - Processing parameters

SI	65536
SF	500.3000125 MHz
WDW	EM
SSB	0
LB	0.30 Hz
GB	0
PC	1.00

CDCL3

17e

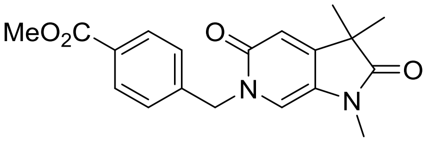

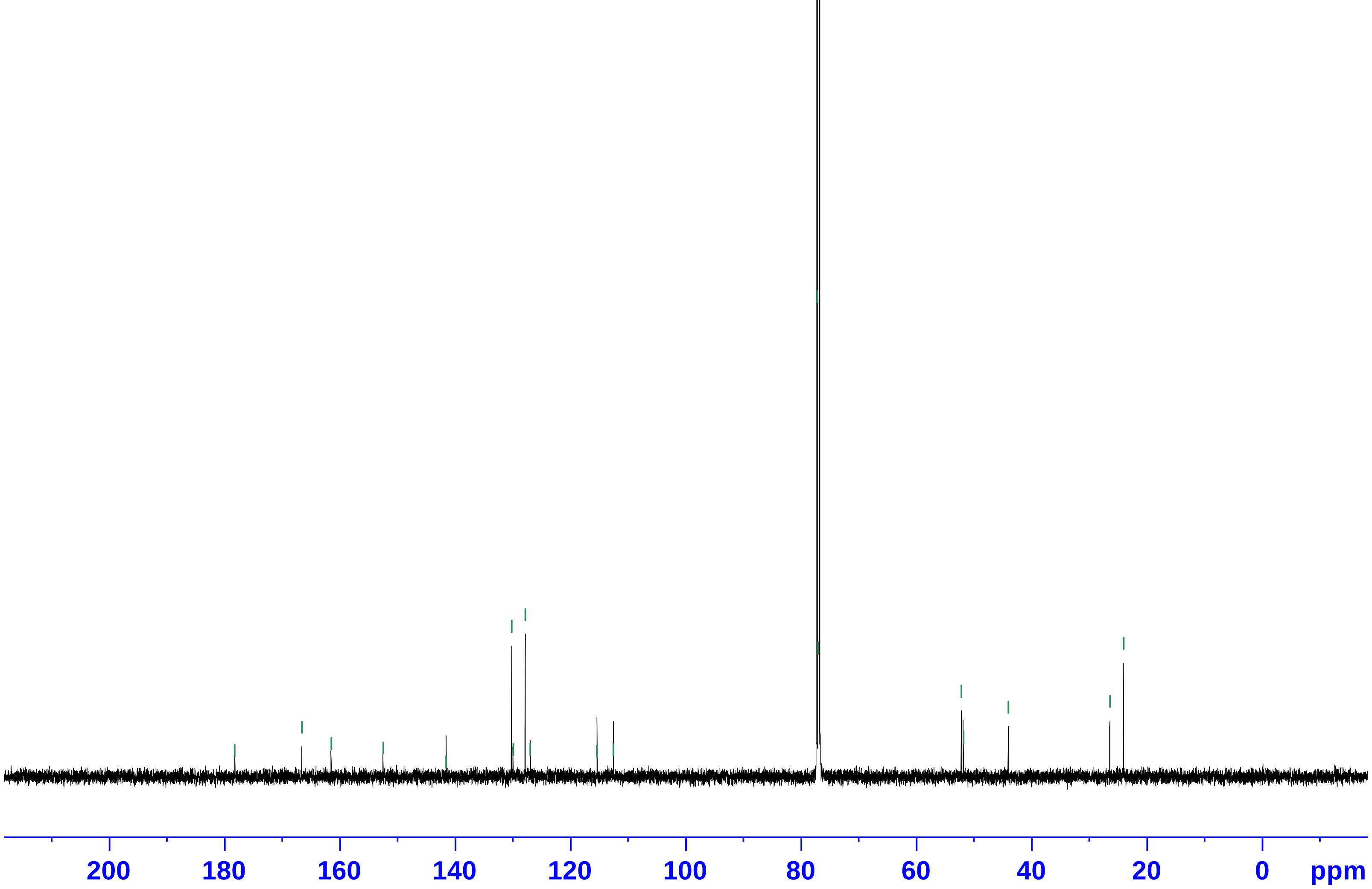

Current Data Parameters
NAME DCM-494-117-A CDCL3
EXPNO 11
PROCNO 1

F2 - Acquisition Parameters
Date_ 20141204
Time 19.54
INSTRUM spect
PROBHD 5 mm PABBO BB-
PULPROG zgpg30
TD 65536
SOLVENT CDCl3
NS 2048
DS 2
SWH 29761.904 Hz
FIDRES 0.454131 Hz
AQ 1.1010048 sec
RG 456
DW 16.800 usec
DE 7.68 usec
TE 298.0 K
D1 2.00000000 sec
D11 0.03000000 sec
TD0 1

======== CHANNEL f1 ========

SFO1 125.8131151 MHz
NUC1 13C
P1 9.75 usec
PLW1 82.38999939 W

======== CHANNEL f2 ========
SFO2 500.3020012 MHz
NUC2 1H
CPDPRG[2 waltz16
PCPD2 80.00 usec
PLW2 18.33600044 W
PLW12 0.75654000 W
PLW13 0.48418999 W

F2 - Processing parameters
SI 65536
SF 125.8005350 MHz
WDW EM
SSB 0
LB 1.00 Hz
GB 0
PC 1.40

17f DMSO

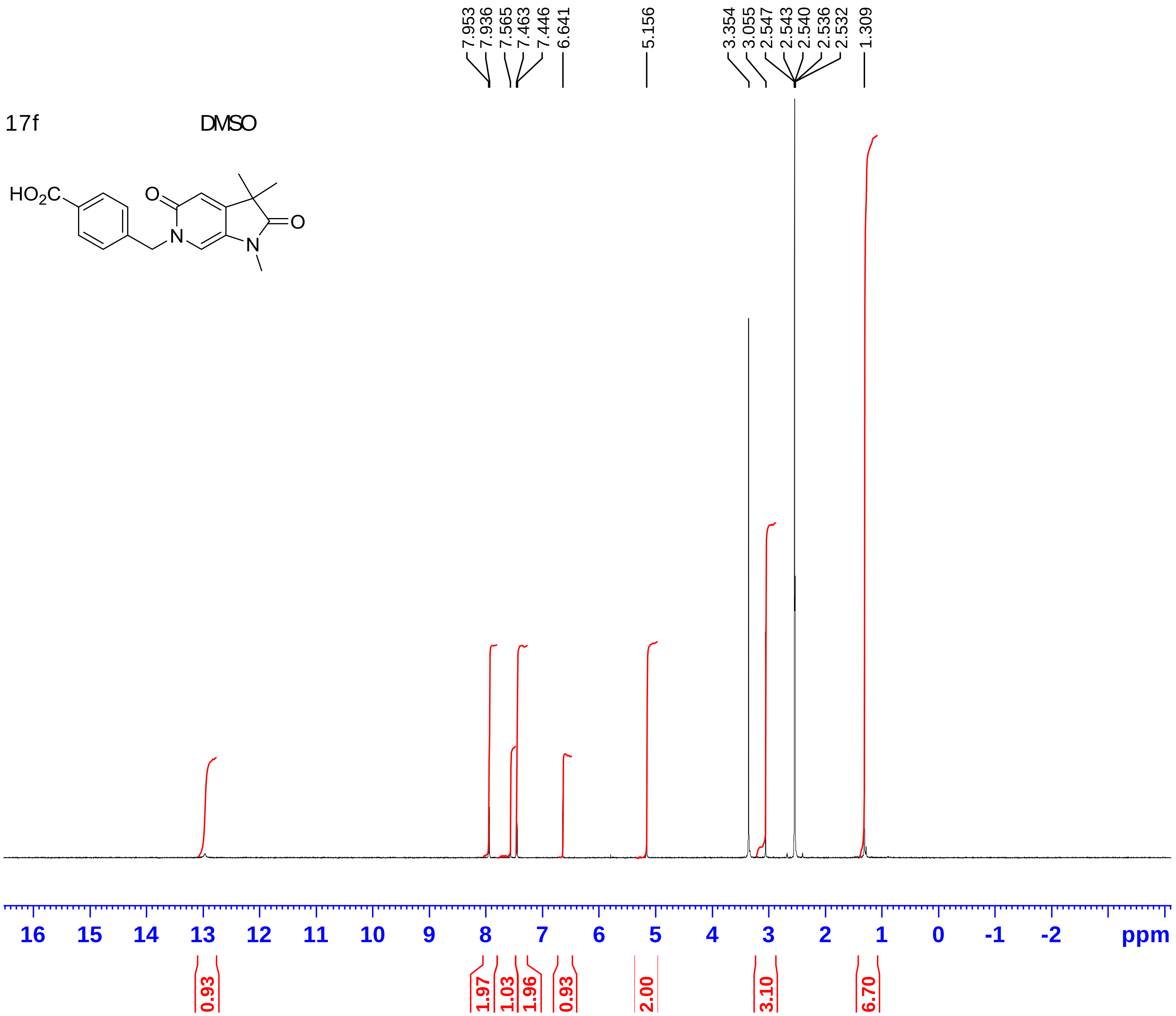

Current Data Parameters

NAME	DCM-494-119-A DMSO
EXPNO	10
PROCNO	1

F2 - Acquisition Parameters

Date_	20141208
Time	16.06
INSTRUM	spect
PROBHD	5 mm PABBO BB-
PULPROG	zg30
TD	65536
SOLVENT	DMSO
NS	16
DS	0
SWH	10330.578 Hz
FIDRES	0.157632 Hz
AQ	3.1719425 sec
RG	812
DW	48.400 usec
DE	11.99 usec
TE	298.0 K
D1	1.00000000 sec
TD0	1

======== CHANNEL f1 ========

SFO1	500.3030896 MHz
NUC1	1H
P1	16.25 usec
PLW1	18.33600044 W

F2 - Processing parameters

SI	65536
SF	500.2999843 MHz
WDW	EM
SSB	0
LB	0.30 Hz
GB	0
PC	1.00

DMSO

17f

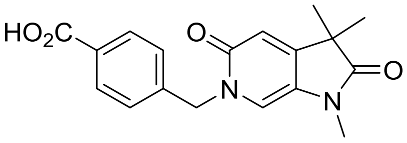

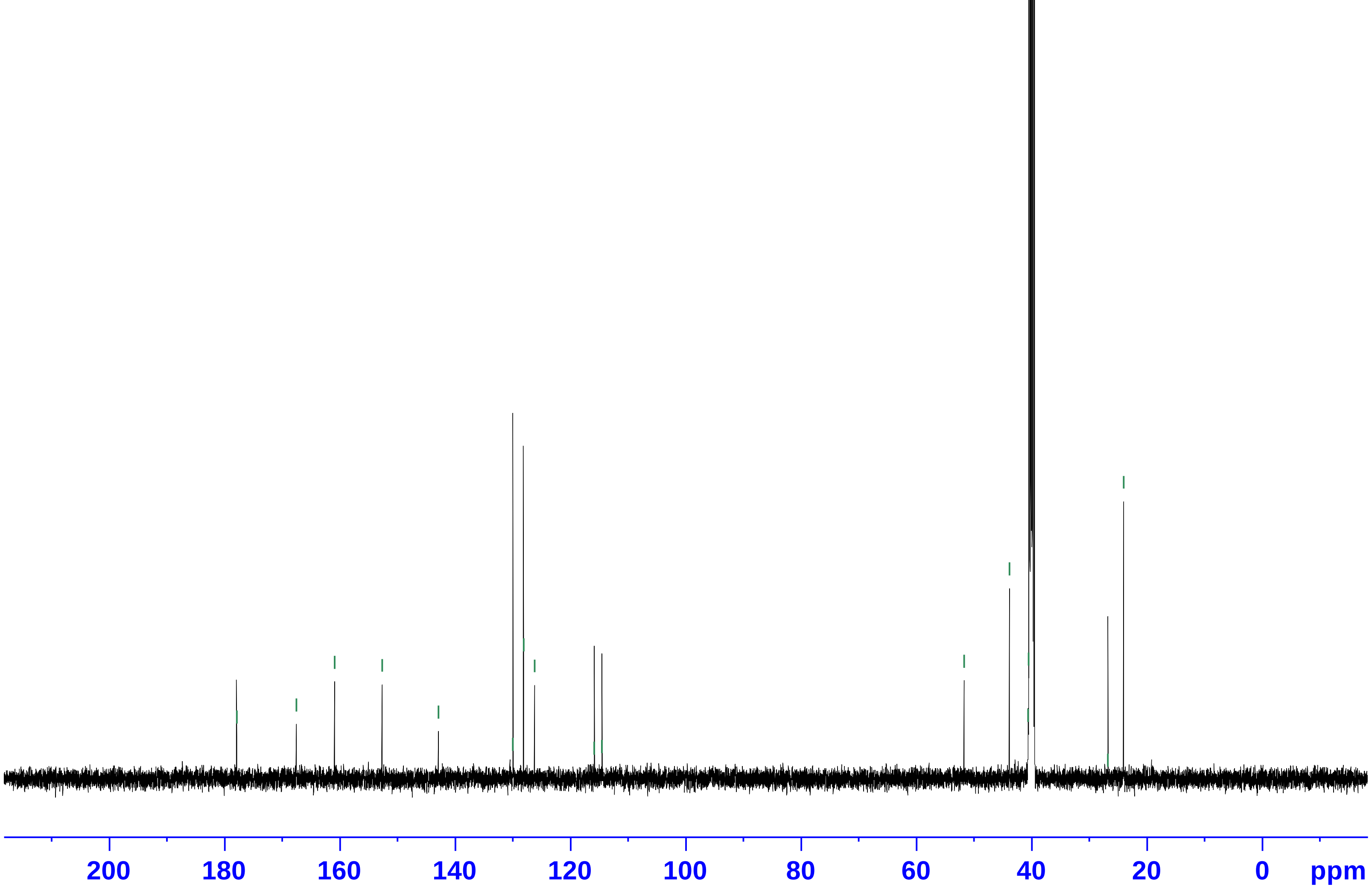

Current Data Parameters
NAME DCM-494-119-A DMSO
EXPNO 11
PROCNO 1

F2 - Acquisition Parameters
Date_ 20141209
Time 5.59
INSTRUM spect
PROBHD 5 mm PABBO BB-
PULPROG zgpg30
TD 65536
SOLVENT DMSO
NS 2048
DS 2
SWH 29761.904 Hz
FIDRES 0.454131 Hz
AQ 1.1010048 sec
RG 456
DW 16.800 usec
DE 7.68 usec
TE 298.0 K
D1 2.00000000 sec
D11 0.03000000 sec
TD0 1

======== CHANNEL f1 ========

SFO1 125.8131151 MHz
NUC1 13C
P1 9.75 usec
PLW1 82.38999939 W

======== CHANNEL f2 ========
SFO2 500.3020012 MHz
NUC2 1H
CPDPRG[2 waltz16
PCPD2 80.00 usec
PLW2 18.33600044 W
PLW12 0.75654000 W
PLW13 0.48418999 W

F2 - Processing Parameters
SI 65536
SF 125.8005350 MHz
WDW EM
SSB 0
LB 1.00 Hz
GB 0
PC 1.40

17g

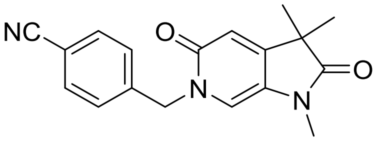

"DCM-494-123-A CDCL3" 10 1 z:\data\DCM\nmr

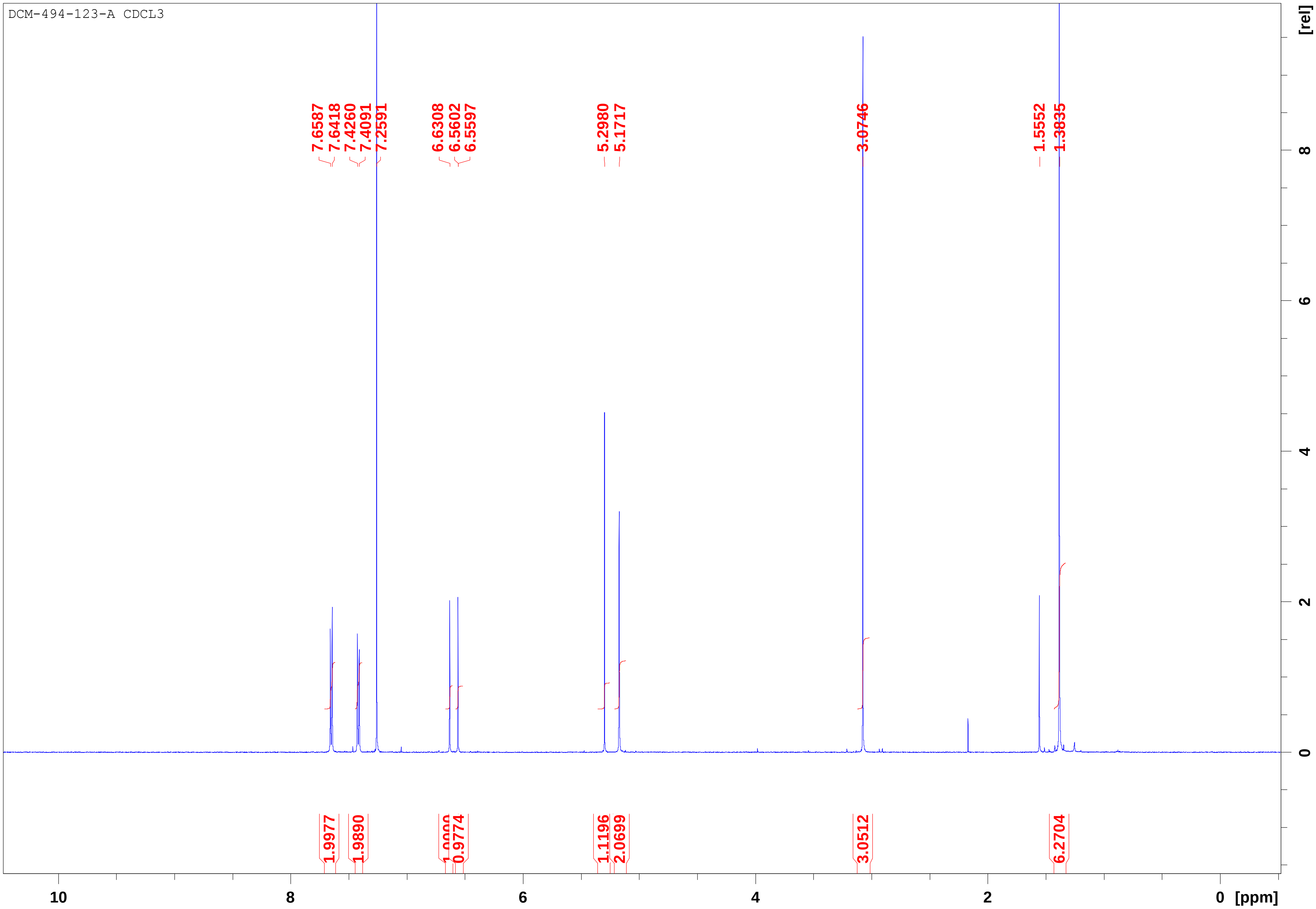

17g

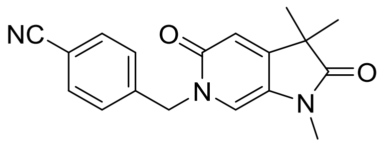
NC
O
O
N
N

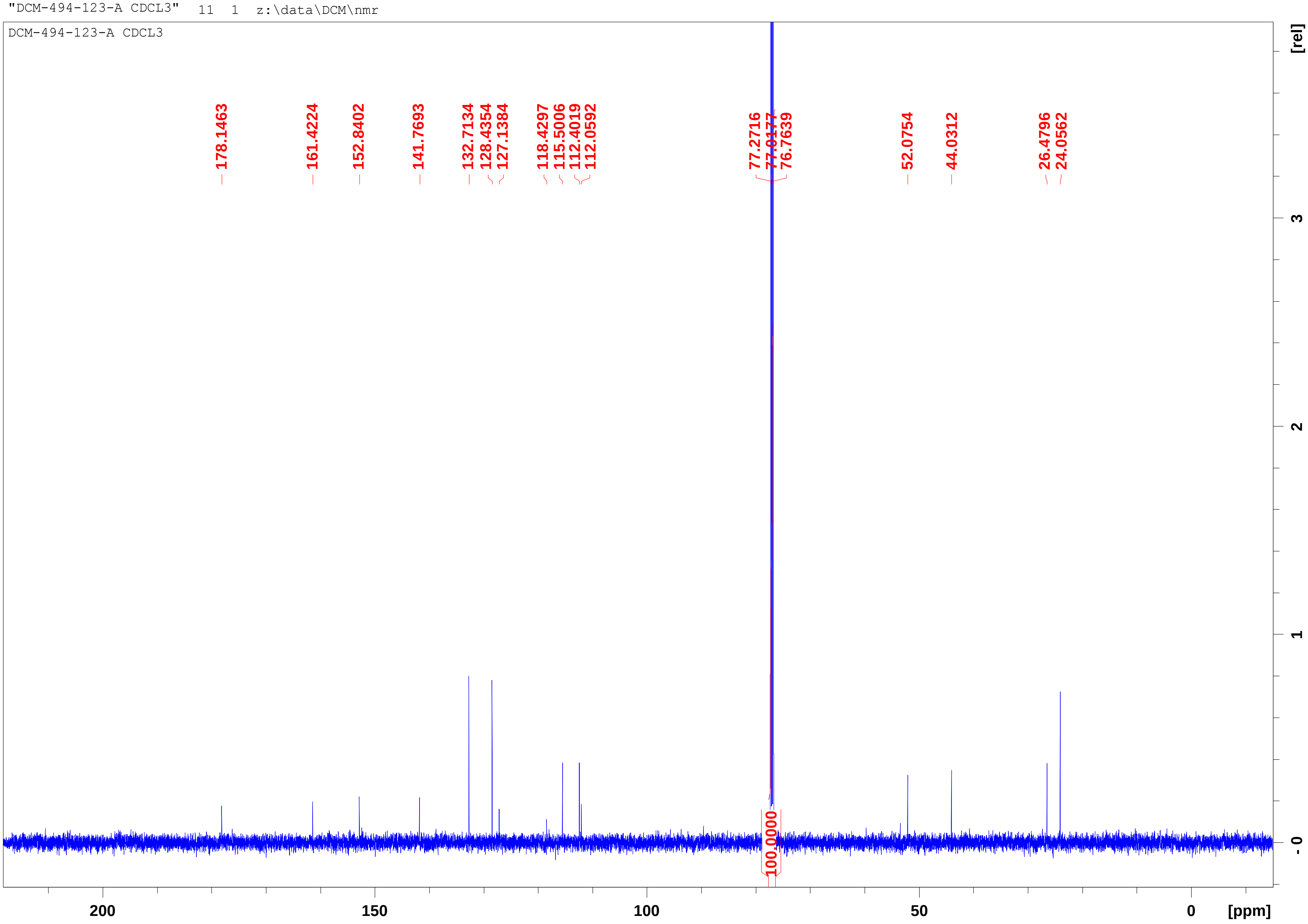
"DCM-494-123-A CDCL3" 11 1 z:\data\DCM\nmr
DCM-494-123-A CDCL3
178.1463
161.4224
152.8402
141.7693
132.7134
128.4354
127.1384
118.4297
115.5006
112.4019
112.0592
77.2716
77.0177
76.7639
52.0754
44.0312
26.4796
24.0562
100.0000
[rel]
3
2
1
0
200
150
100
50
0
[ppm]

17h

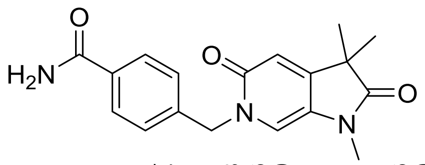

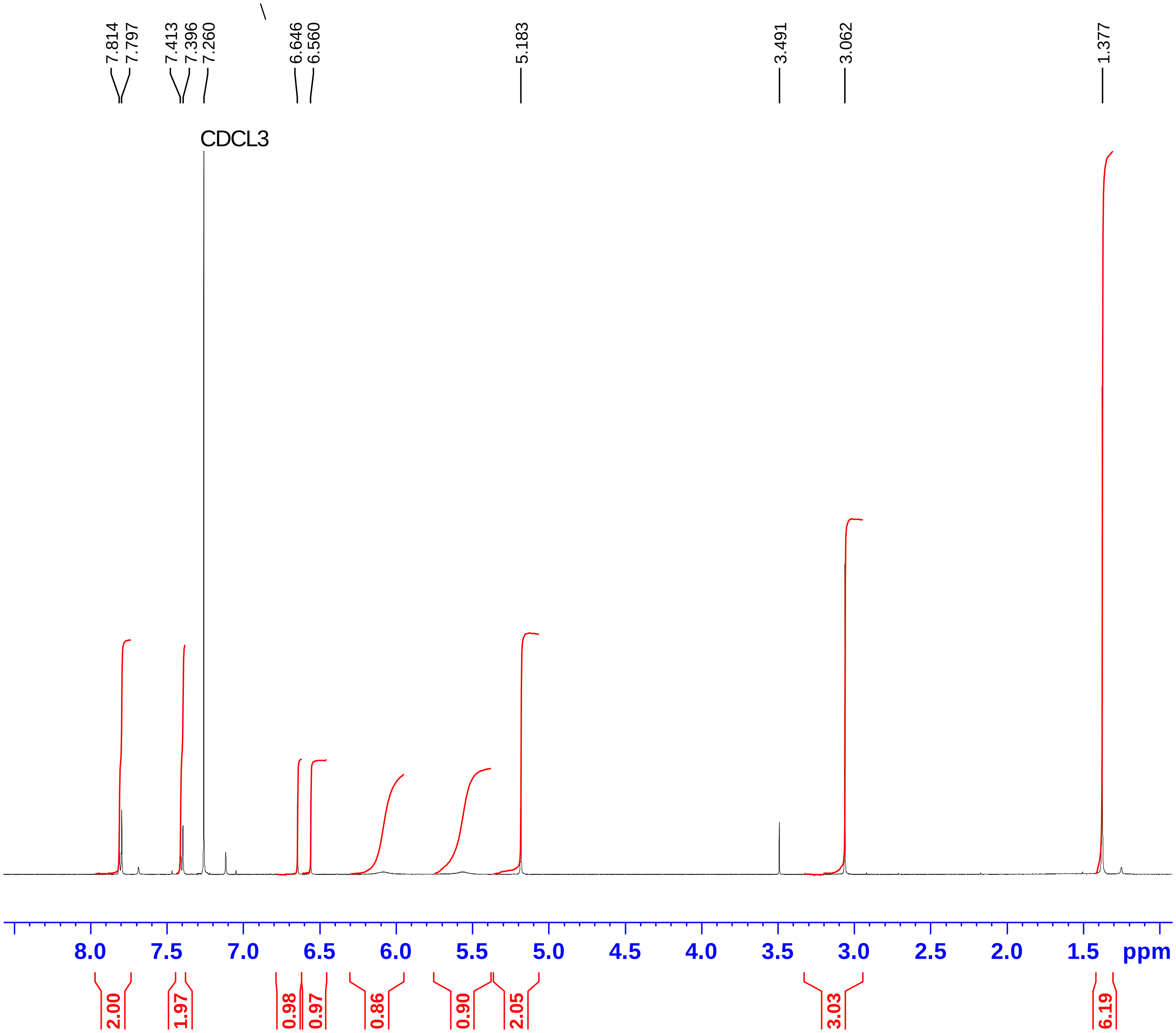

Current Data Parameters

NAME	DCM-494-147-B CDCL3
EXPNO	10
PROCNO	1

F2 - Acquisition Parameters

Date_	20150129
Time	14.56
INSTRUM	spect
PROBHD	5 mm PABBO BB-
PULPROG	zg30
TD	65536
SOLVENT	CDCl3
NS	16
DS	0
SWH	10330.578 Hz
FIDRES	0.157632 Hz
AQ	3.1719425 sec
RG	724
DW	48.400 usec
DE	11.99 usec
TE	293.5 K
D1	1.00000000 sec
TD0	1

======== CHANNEL f1 ========

SFO1	500.3030896 MHz
NUC1	1H
P1	16.25 usec
PLW1	18.33600044 W

F2 - Processing parameters

SI	65536
SF	500.3000124 MHz
WDW	EM
SSB	0
LB	0.30 Hz
GB	0
PC	1.00

17h

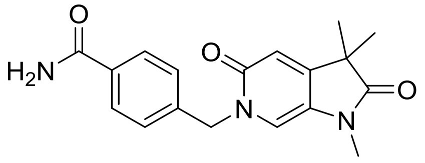

DCM-494-147-B CDCL3

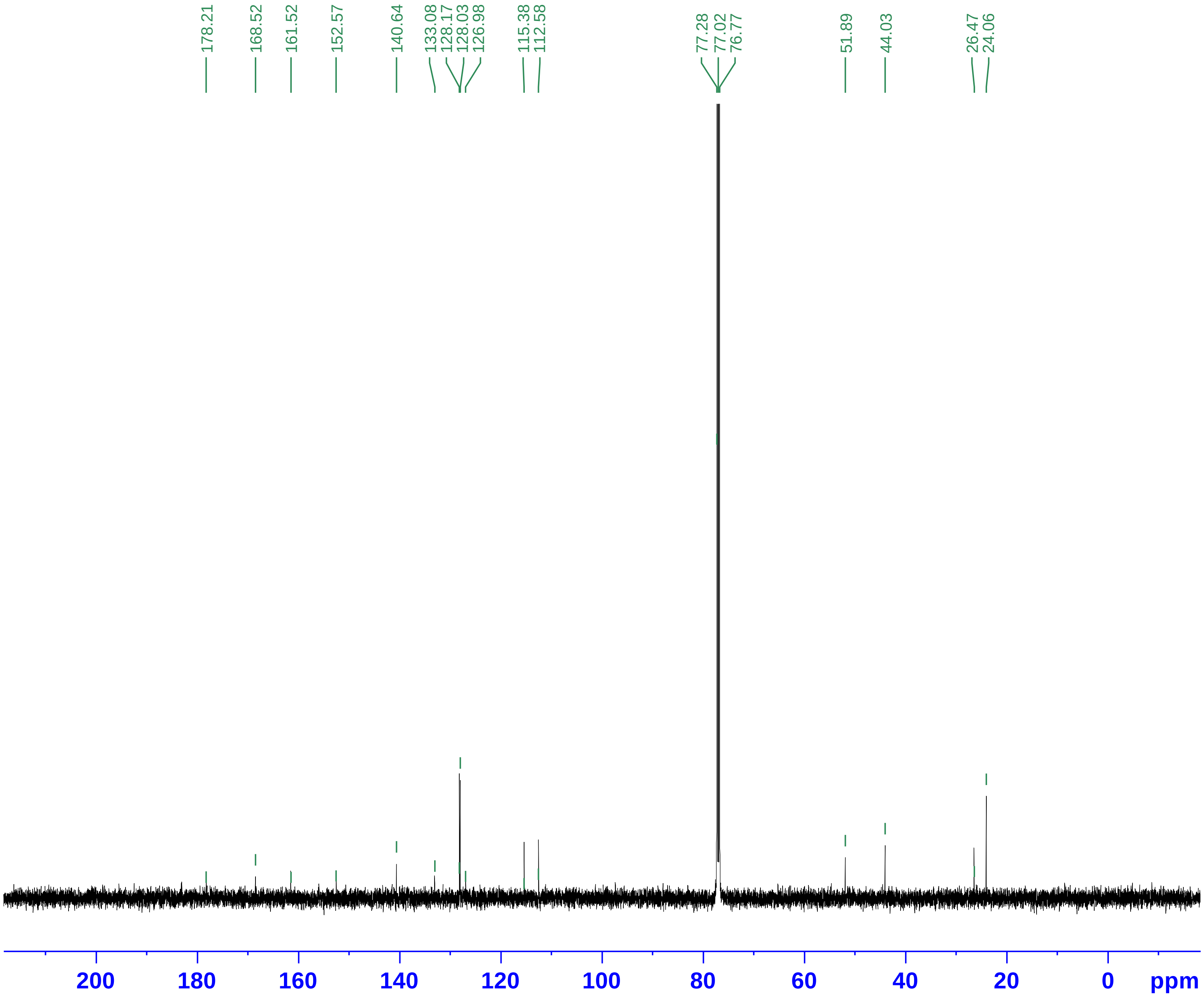

Current Data Parameters
NAME DCM-494-147-B CDCL3
EXPNO 11
PROCNO 1

F2 - Acquisition Parameters
Date_ 20150129
Time 20.33
INSTRUM spect
PROBHD 5 mm PABBO BB-
PULPROG zgpg30
TD 65536
SOLVENT CDCl3
NS 2048
DS 2
SWH 29761.904 Hz
FIDRES 0.454131 Hz
AQ 1.1010048 sec
RG 812
DW 16.800 usec
DE 7.68 usec
TE 296.2 K
D1 2.00000000 sec
D11 0.03000000 sec
TD0 1

======== CHANNEL f1 ========

SFO1 125.8131151 MHz
NUC1 13C
P1 9.75 usec
PLW1 82.38999939 W

======== CHANNEL f2 ========
SFO2 500.3020012 MHz
NUC2 1H
CPDPRG[2 waltz16
PCPD2 80.00 usec
PLW2 18.33600044 W
PLW12 0.75654000 W
PLW13 0.48418999 W

F2 - Processing parameters
SI 65536
SF 125.8005350 MHz
WDW EM
SSB 0
LB 1.00 Hz
GB 0
PC 1.40

17i

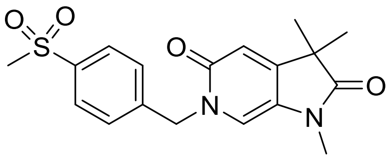

SA-500-34

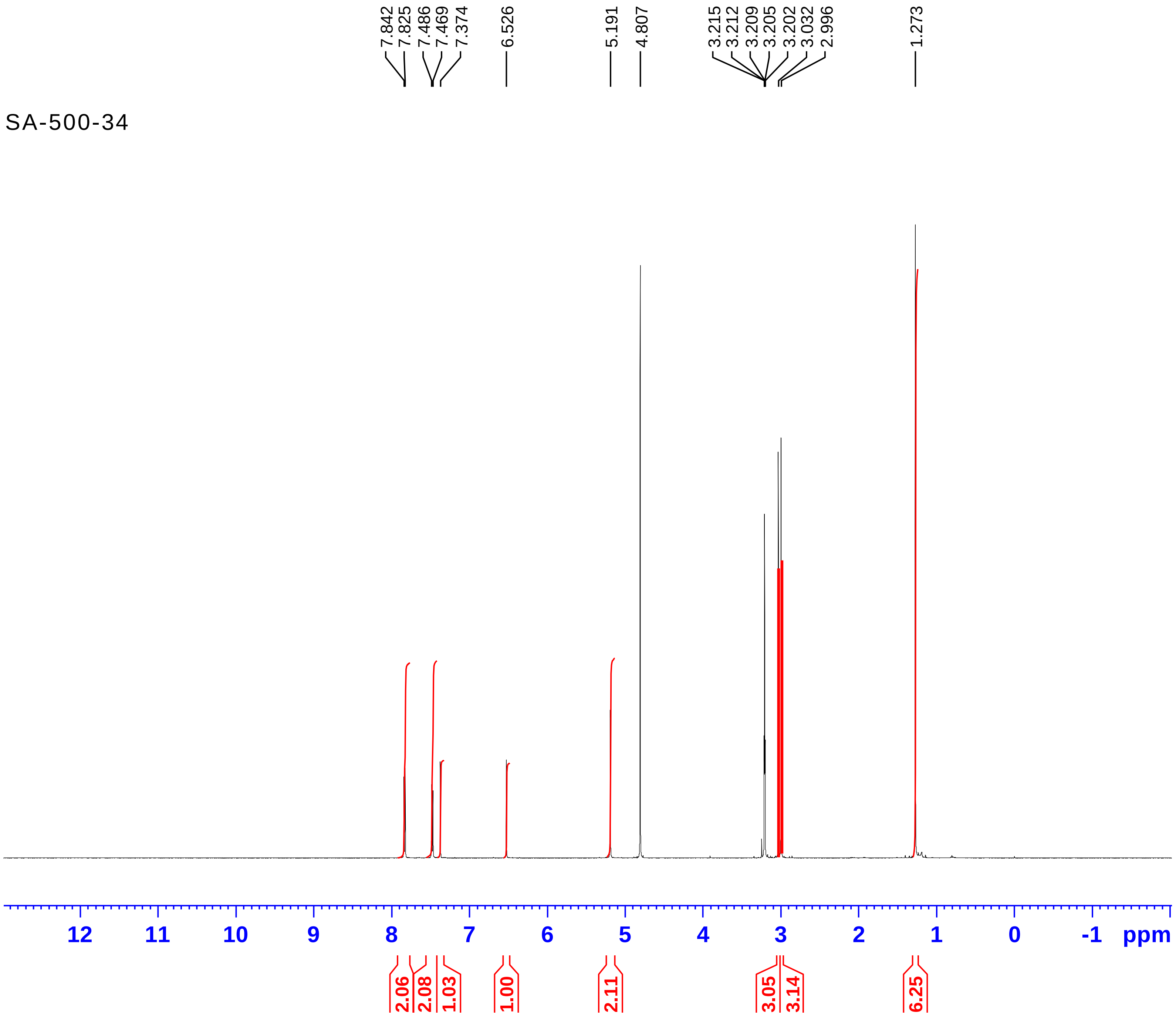

Current Data Parameters

NAME	SA-500-34
EXPNO	10
PROCNO	1

F2 - Acquisition Parameters

Date_	20150122
Time	15.07
INSTRUM	spect
PROBHD	5 mm PABBO BB-
PULPROG	zg30
TD	65536
SOLVENT	MeOD
NS	16
DS	0
SWH	10330.578 Hz
FIDRES	0.157632 Hz
AQ	3.1719425 sec
RG	456
DW	48.400 usec
DE	11.99 usec
TE	292.3 K
D1	1.00000000 sec
TD0	1

======== CHANNEL f1 ========

SFO1	500.3030896 MHz
NUC1	1H
P1	16.25 usec
PLW1	18.33600044 W

F2 - Processing parameters

SI	65536
SF	500.3000603 MHz
WDW	EM
SSB	0
LB	0.30 Hz
GB	0
PC	1.00

17i

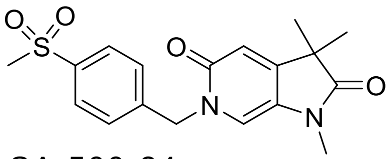

SA-500-34

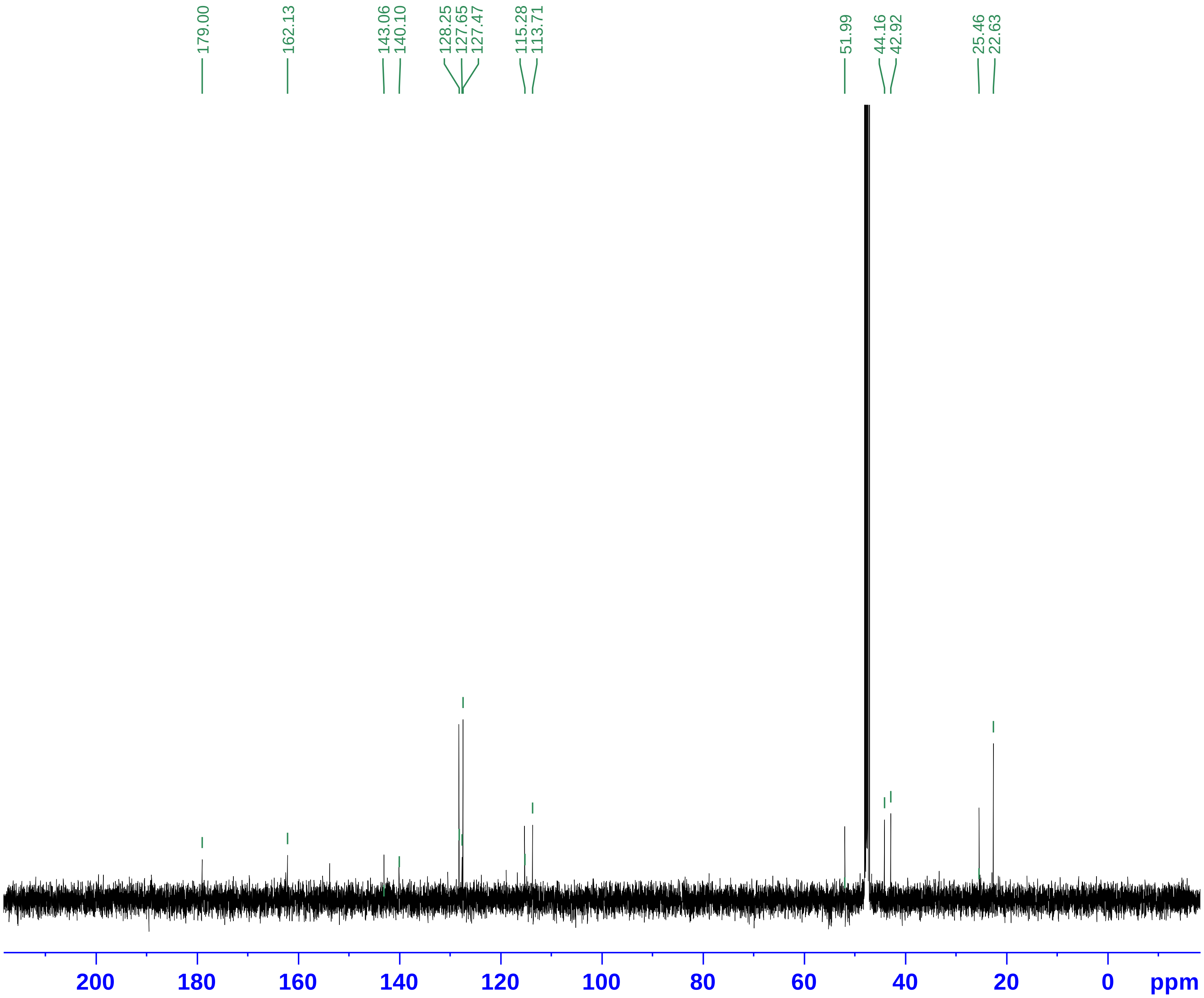

Current Data Parameters
NAME SA-500-34
EXPNO 11
PROCNO 1

F2 - Acquisition Parameters
Date_ 20150122
Time 16.15
INSTRUM spect
PROBHD 5 mm PABBO BB-
PULPROG zgpg30
TD 65536
SOLVENT MeOD
NS 256
DS 2
SWH 29761.904 Hz
FIDRES 0.454131 Hz
AQ 1.1010048 sec
RG 812
DW 16.800 usec
DE 7.68 usec
TE 295.3 K
D1 2.00000000 sec
D11 0.03000000 sec
TD0 1

======== CHANNEL f1 ========

SFO1 125.8131151 MHz
NUC1 13C
P1 9.75 usec
PLW1 82.38999939 W

======== CHANNEL f2 ========
SFO2 500.3020012 MHz
NUC2 1H
CPDPRG[2 waltz16
PCPD2 80.00 usec
PLW2 18.33600044 W
PLW12 0.75654000 W
PLW13 0.48418999 W

F2 - Processing Parameters
SI 65536
SF 125.8005350 MHz
WDW EM
SSB 0
LB 1.00 Hz
GB 0
PC 1.40

17j

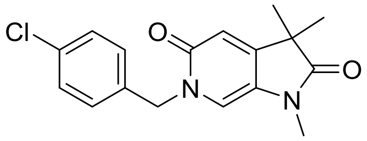

SA-500-42 11 1 Z:\data\SA\nmr

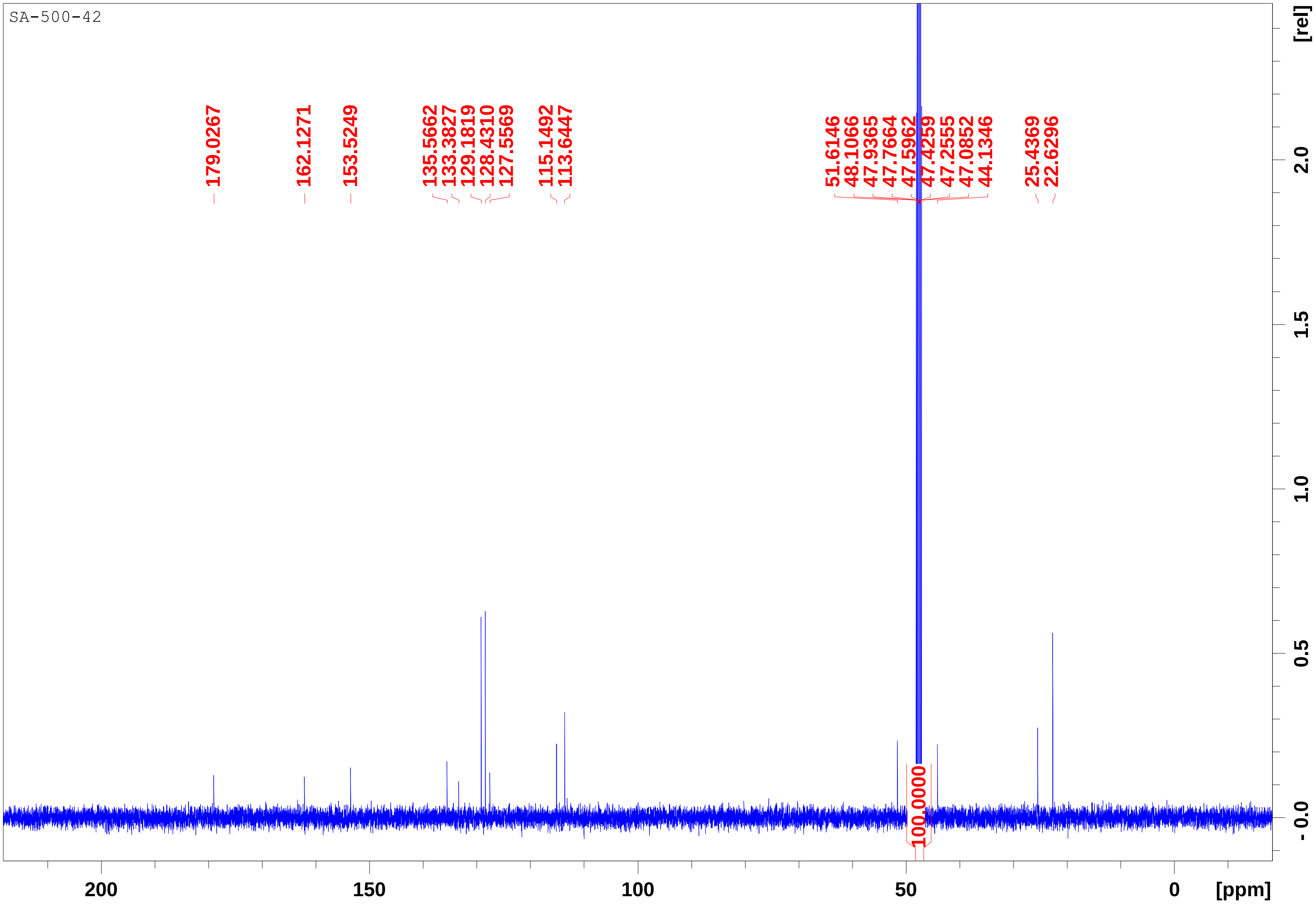

17k

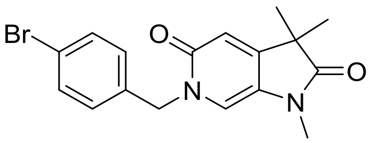

SA-500-64

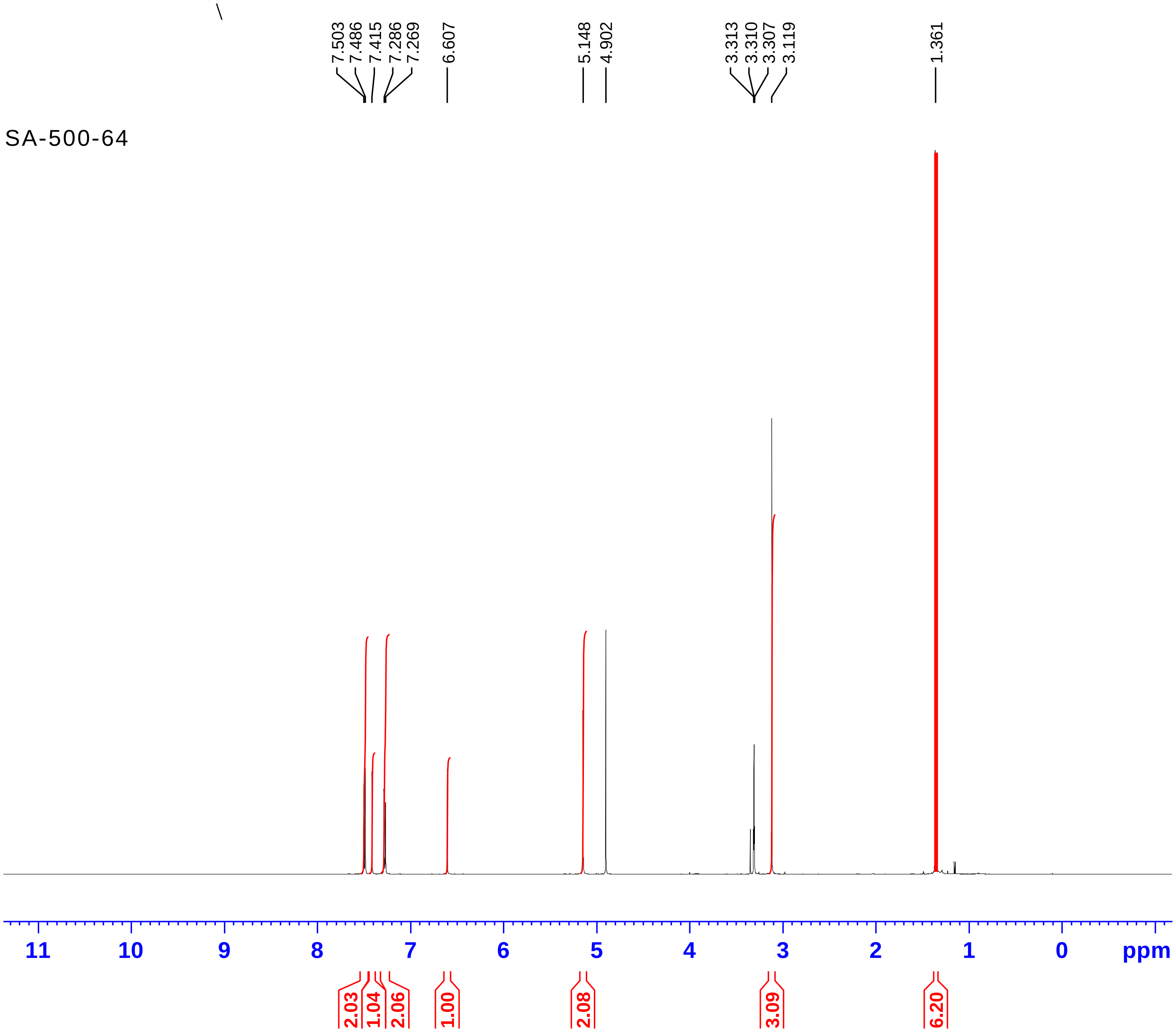

Current Data Parameters
NAME SA-500-64
EXPNO 10
PROCNO 1

F2 - Acquisition Parameters
Date 20150213
Time 13.49
INSTRUM spect
PROBHD 5 mm PABBO BB-
PULPROG zg30
TD 65536
SOLVENT MeOD
NS 16
DS 0
SWH 10330.578 Hz
FIDRES 0.157632 Hz
AQ 3.1719425 sec
RG 406
DW 48.400 usec
DE 11.99 usec
TE 292.9 K
D1 1.00000000 sec
TD0 1

======== CHANNEL f1 ========
SFO1 500.3030896 MHz
NUC1 1H
P1 16.25 usec
PLW1 18.33600044 W

F2 - Processing parameters SI 65536
SF 500.3000098 MHz
WDW EM
SSB 0
LB 0.30 Hz
GB 0
PC 1.00

17k

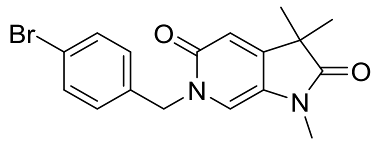

SA-500-64

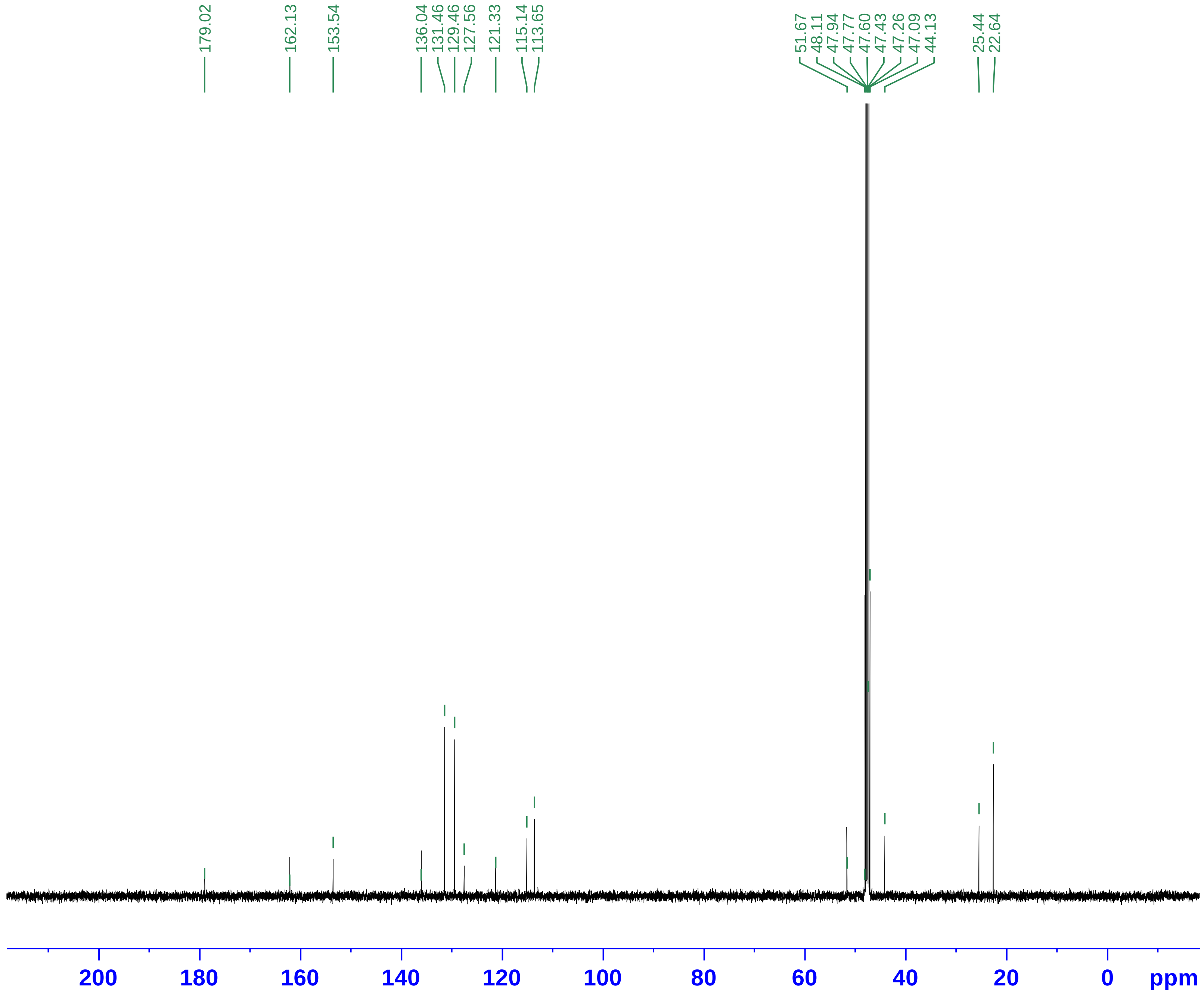

BRUKER

Newcastle University

CANCER RESEARCH UK

Current Data Parameters
NAME SA-500-64
EXPNO 11
PROCNO 1

F2 - Acquisition
Parameters Date_ 20150213
Time 14.25
INSTRUM spect
PROBHD 5 mm PABBO BB-
PULPROG zgpg30
TD 65536
SOLVENT MeOD
NS 256
DS 2
SWH 29761.904 Hz
FIDRES 0.454131 Hz
AQ 1.1010048 sec
RG 456
DW 16.800 usec
DE 7.68 usec
TE 296.0 K
D1 2.00000000 sec
D11 0.03000000 sec
TD0 1

======== CHANNEL f1 ========

SFO1 125.8131151 MHz
NUC1 13C
P1 9.75 usec
PLW1 82.38999939 W

======== CHANNEL f2 ========
SFO2 500.3020012 MHz
NUC2 1H
CPDPRG[2 waltz16
PCPD2 80.00 usec
PLW2 18.33600044 W
PLW12 0.75654000 W
PLW13 0.48418999 W

F2 - Processing
parameters SI 65536
SF 125.8005350 MHz
WDW EM
SSB 0
LB 1.00 Hz
GB 0
PC 1.40

17l

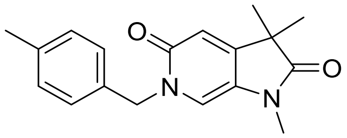

SA-500-74

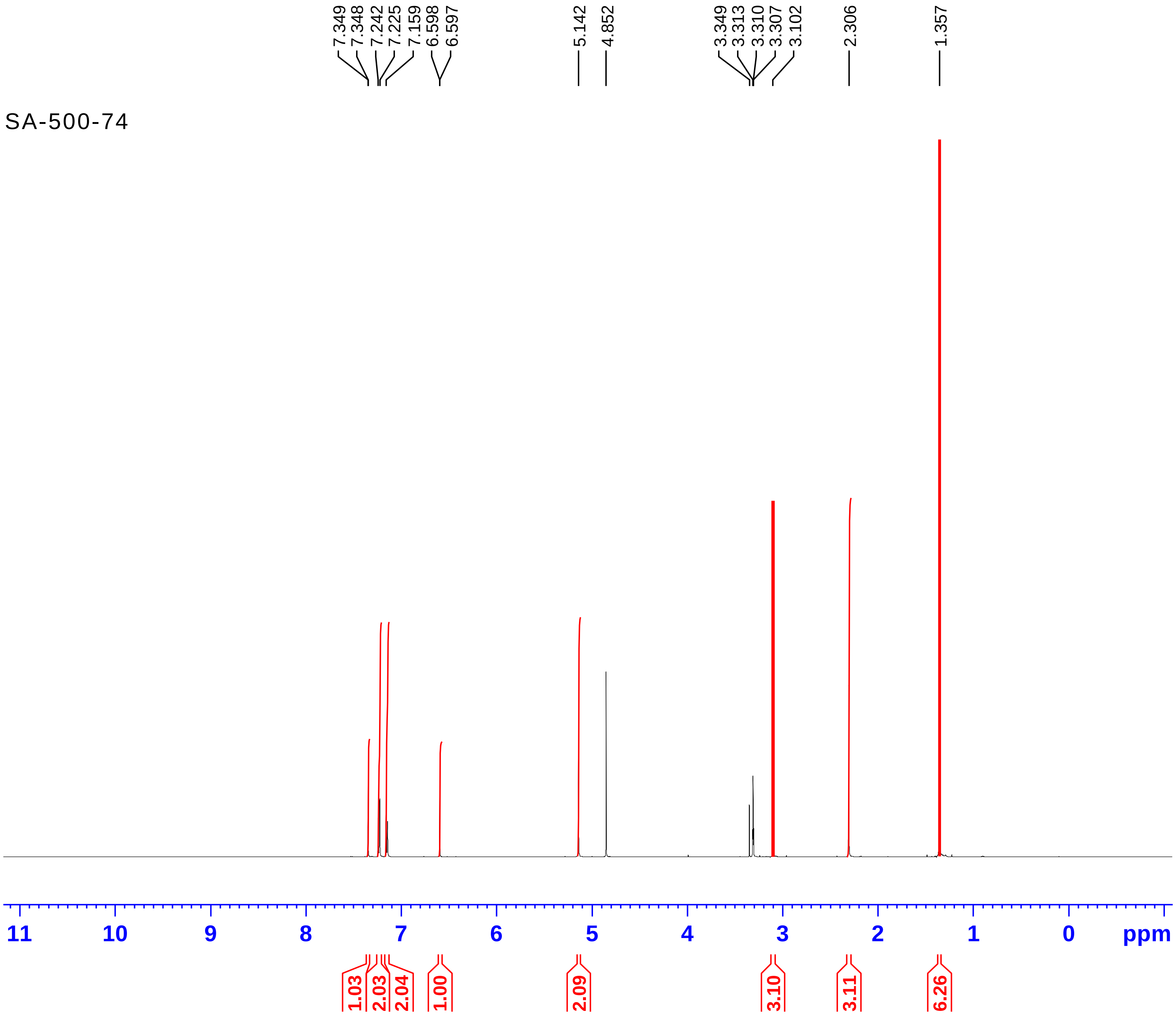

Current Data Parameters

NAME	SA-500-74	
EXPNO	10	
PROCNO	1	

F2 - Acquisition Parameters

Date_	20150218	
Time	14.51	
INSTRUM	spect	
PROBHD	5 mm PABBO BB-	
PULPROG	zg30	
TD	65536	
SOLVENT	MeOD	
NS	16	
DS	0	
SWH	10330.578	Hz
FIDRES	0.157632	Hz
AQ	3.1719425	sec
RG	406	
DW	48.400	usec
DE	11.99	usec
TE	298.0	K
D1	1.00000000	sec
TD0	1	

======== CHANNEL f1 ========

SFO1	500.3030896	MHz
NUC1	1H	
P1	16.25	usec
PLW1	18.33600044	W

F2 - Processing parameters SI 65536

SF	500.3000097	MHz
WDW	EM	
SSB	0	
LB	0.30	Hz
GB	0	
PC	1.00	

17l

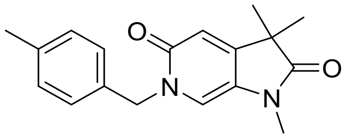

SA-500-74

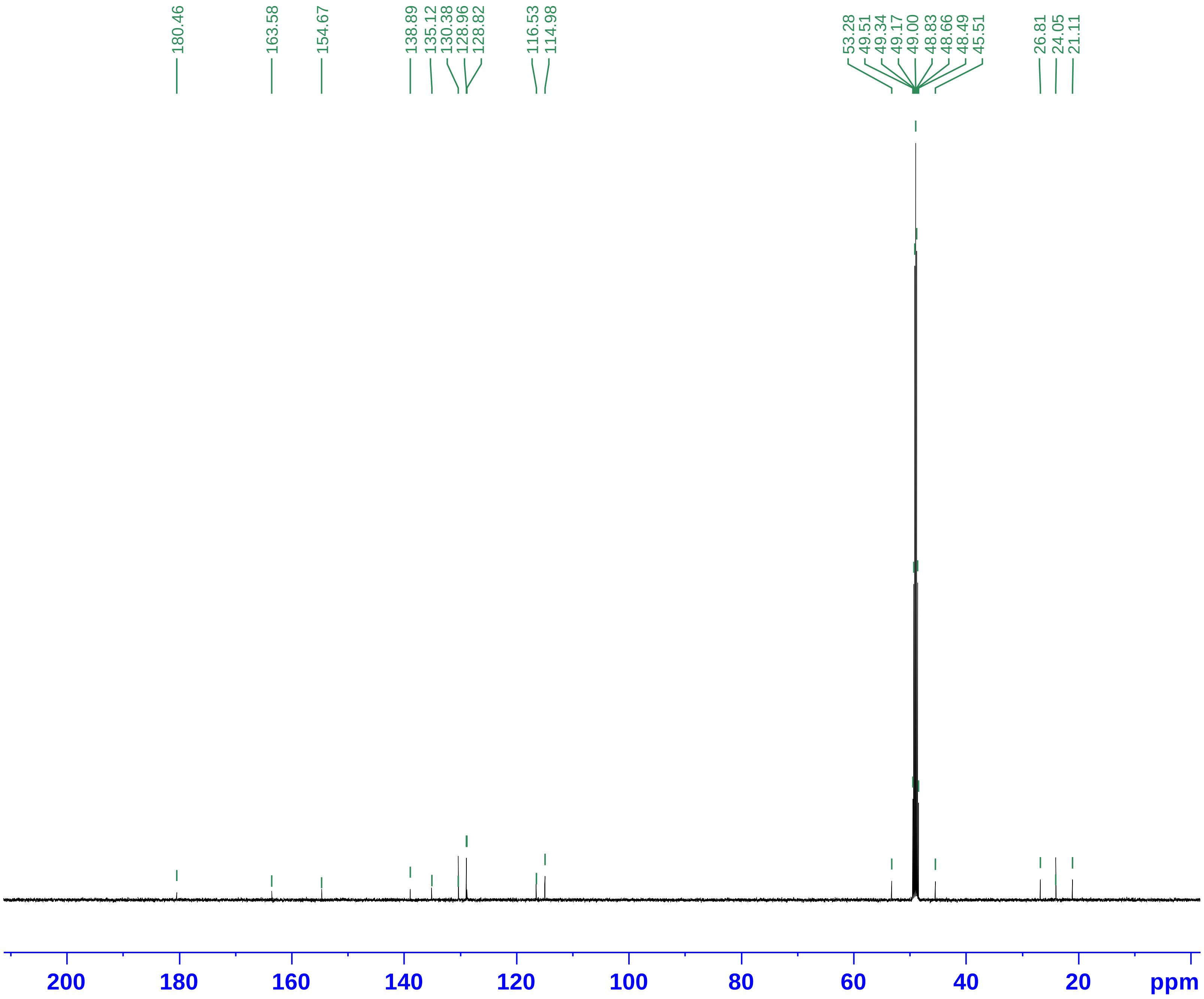

Current Data Parameters
NAME SA-500-74
EXPNO 11
PROCNO 1

F2 - Acquisition Parameters Date_ 20150218
Time 17.40
INSTRUM spect
PROBHD 5 mm PABBO BB-
PULPROG zgpg30
TD 65536
SOLVENT MeOD
NS 256
DS 2
SWH 29761.904 Hz
FIDRES 0.454131 Hz
AQ 1.1010048 sec
RG 456
DW 16.800 usec
DE 7.68 usec
TE 298.0 K
D1 2.00000000 sec
D11 0.03000000 sec
TD0 1

======== CHANNEL f1 ========

SFO1 125.8131151 MHz
NUC1 13C
P1 9.75 usec
PLW1 82.38999939 W

======== CHANNEL f2 ========
SFO2 500.3020012 MHz
NUC2 1H
CPDPRG[2 waltz16
PCPD2 80.00 usec
PLW2 18.33600044 W
PLW12 0.75654000 W
PLW13 0.48418999 W

F2 - Processing parameters
SI 65536
SF 125.8003589 MHz
WDW EM
SSB 0
LB 1.00 Hz
GB 0
PC 1.40

17m

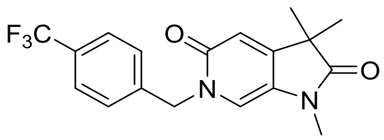

SA-500-68

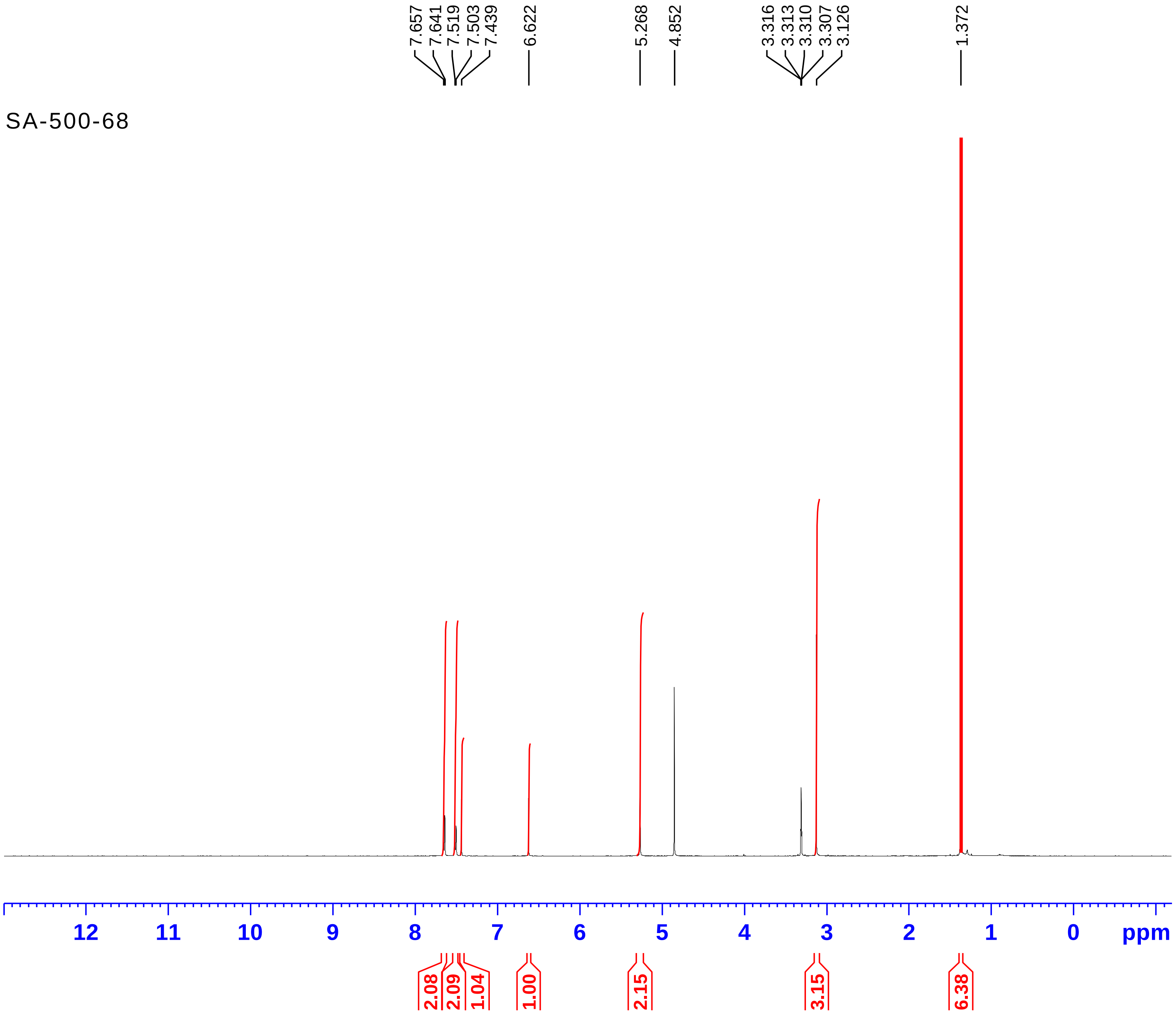

Current Data Parameters
NAME SA-500-68
EXPNO 10
PROCNO 1

F2 - Acquisition Parameters
Date 20150216
Time 16.39
INSTRUM spect
PROBHD 5 mm PABBO BB-
PULPROG zg30
TD 65536
SOLVENT MeOD
NS 16
DS 0
SWH 10330.578 Hz
FIDRES 0.157632 Hz
AQ 3.1719425 sec
RG 406
DW 48.400 usec
DE 11.99 usec
TE 298.1 K
D1 1.00000000 sec
TD0 1

======== CHANNEL f1 ========
SFO1 500.3030896 MHz
NUC1 1H
P1 16.25 usec
PLW1 18.33600044 W

F2 - Processing parameters SI 65536
SF 500.3000097 MHz
WDW EM
SSB 0
LB 0.30 Hz
GB 0
PC 1.00

17m

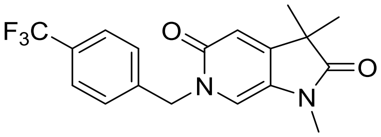

SA-500-68

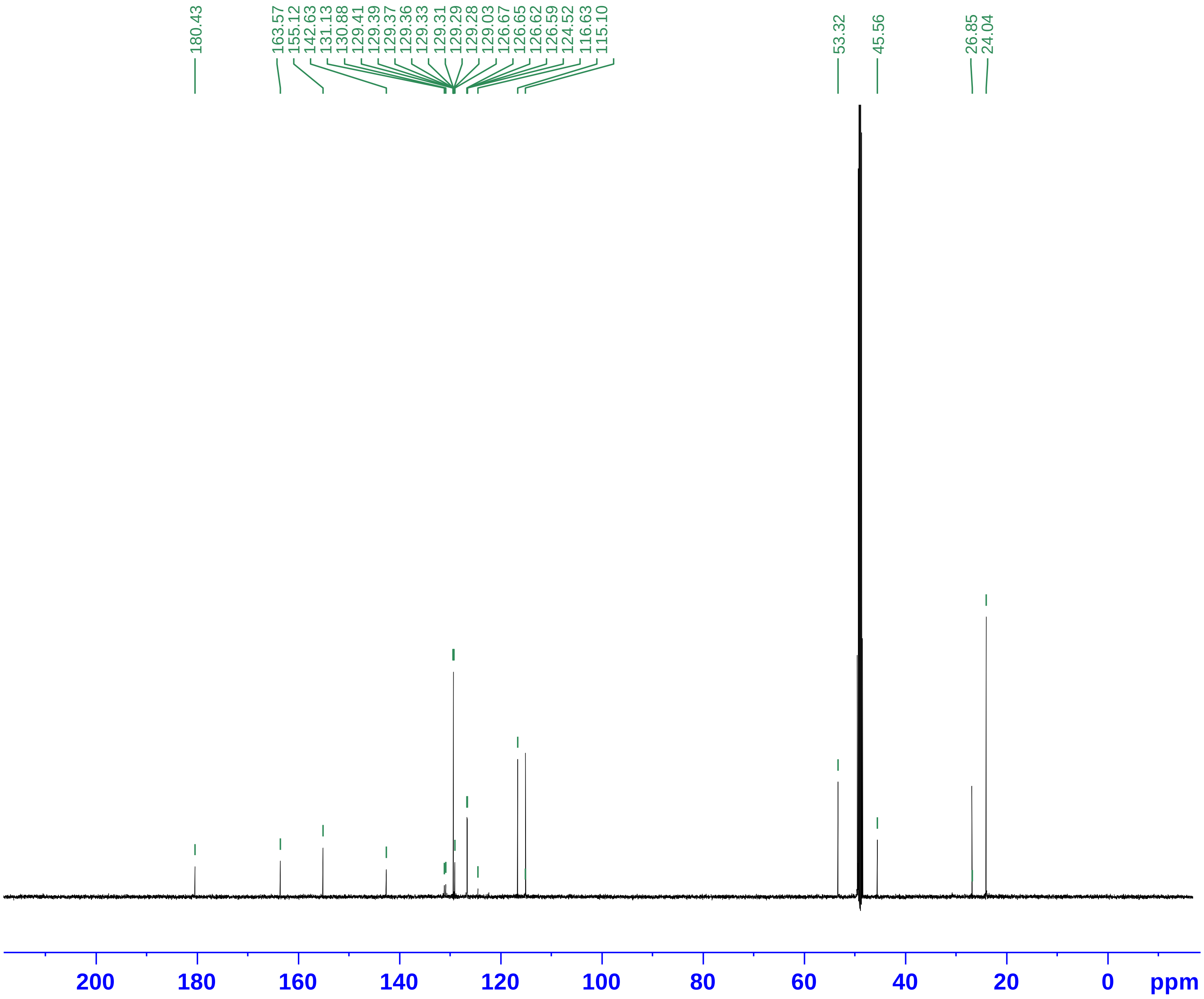

Current Data Parameters
NAME SA-500-68
EXPNO 15
PROCNO 1

F2 - Acquisition Parameters
Date_ 20150216
Time 22.24
INSTRUM spect
PROBHD 5 mm PABBO BB-
PULPROG zgpg
TD 26782
SOLVENT MeOD
NS 2048
DS 2
SWH 29761.904 Hz
FIDRES 1.111265 Hz
AQ 0.4499376 sec
RG 456
DW 16.800 usec
DE 7.72 usec
TE 298.0 K
D1 5.00000000 sec
D11 0.03000000 sec
TD0 1

======== CHANNEL f1 ========
SFO1 125.8131151 MHz
NUC1 13C
P1 9.75 usec
PLW1 82.38999939 W

======== CHANNEL f2 ========
SFO2 500.3020012 MHz
NUC2 1H
CPDPRG[2 waltz16
PCPD2 80.00 usec
PLW2 18.33600044 W
PLW12 0.75654000 W
PLW13 0.48418999 W

F2 - Processing parameters
SI 65536
SF 125.8005350 MHz
WDW EM
SSB 0
LB 1.00 Hz
GB 0
PC 1.40

17n

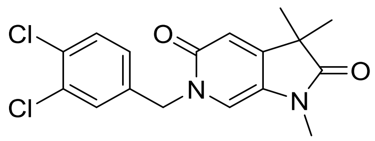

SA-500-100

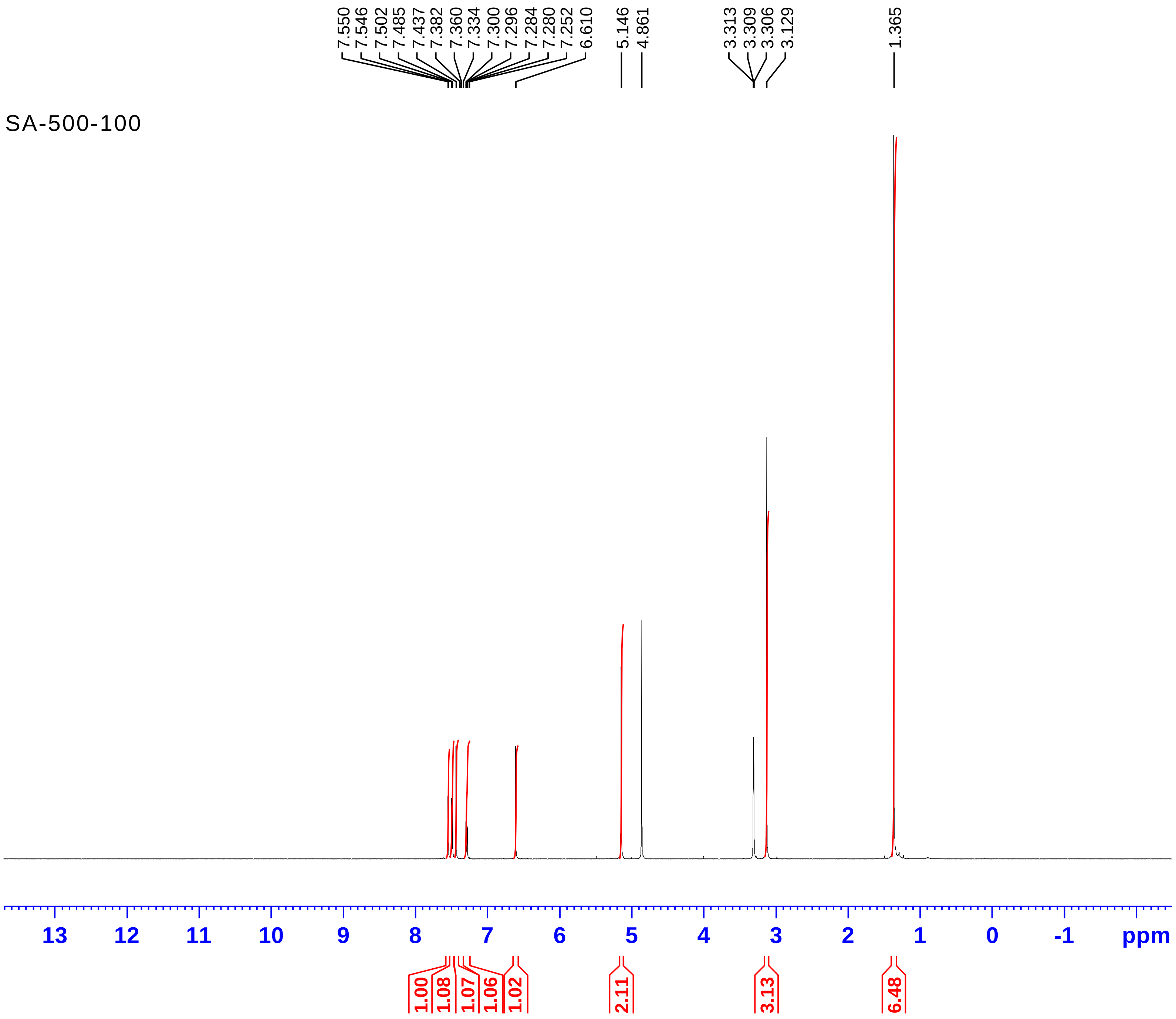

Current Data Parameters
NAME SA-500-100-DRIED
EXPNO 10
PROCNO 1

F2 - Acquisition Parameters
Date 20150317
Time 16.52
INSTRUM spect
PROBHD 5 mm PABBO BB-
PULPROG zg30
TD 65536
SOLVENT MeOD
NS 16
DS 0
SWH 10330.578 Hz
FIDRES 0.157632 Hz
AQ 3.1719425 sec
RG 456
DW 48.400 usec
DE 11.99 usec
TE 296.8 K
D1 1.00000000 sec
TD0 1

======== CHANNEL f1 ========
SFO1 500.3030896 MHz
NUC1 1H
P1 16.25 usec
PLW1 18.33600044 W

F2 - Processing parameters SI 65536
SF 500.3000099 MHz
WDW EM
SSB 0
LB 0.30 Hz
GB 0
PC 1.00

17n

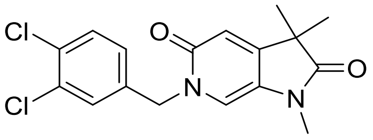

SA-500-100

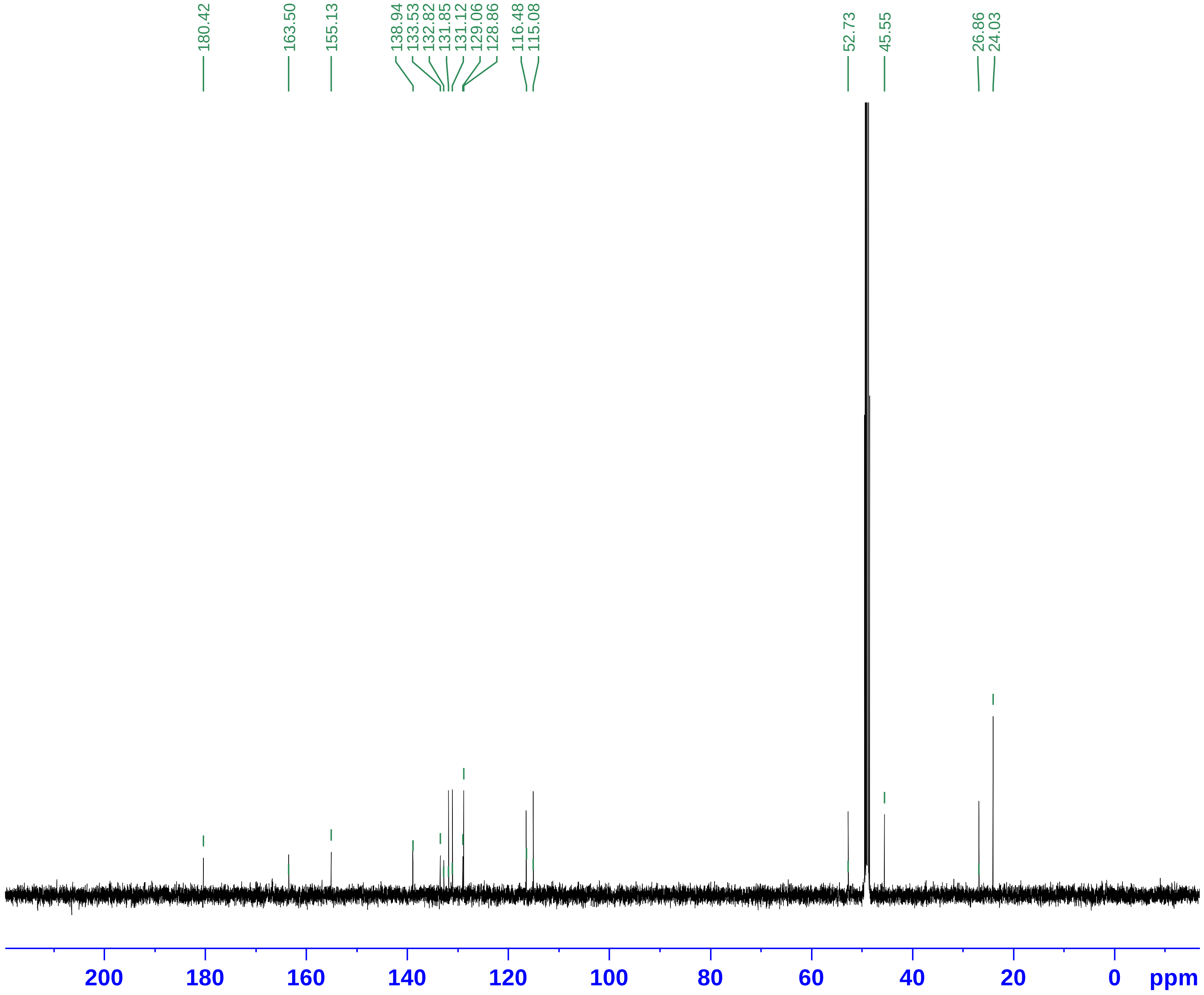

Current Data Parameters
NAME SA-500-100-DRIED
EXPNO 11
PROCNO 1

F2 - Acquisition Parameters
Date_ 20150317
Time 23.06
INSTRUM spect
PROBHD 5 mm PABBO BB-
PULPROG zgpg30
TD 65536
SOLVENT MeOD
NS 256
DS 2
SWH 29761.904 Hz
FIDRES 0.454131 Hz
AQ 1.1010048 sec
RG 575
DW 16.800 usec
DE 7.68 usec
TE 297.0 K
D1 2.00000000 sec
D11 0.03000000 sec
TD0 1

======== CHANNEL f1 ========
SFO1 125.8131151 MHz
NUC1 13C
P1 9.75 usec
PLW1 82.38999939 W

======== CHANNEL f2 ========
SFO2 500.3020012 MHz
NUC2 1H
CPDPRG[2 waltz16
PCPD2 80.00 usec
PLW2 18.33600044 W
PLW12 0.75654000 W
PLW13 0.48418999 W

F2 - Processing parameters
SI 65536
SF 125.8003589 MHz
WDW EM
SSB 0
LB 1.00 Hz
GB 0
PC 1.40

17o

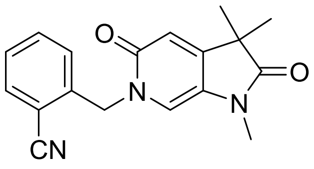

SA-500-26

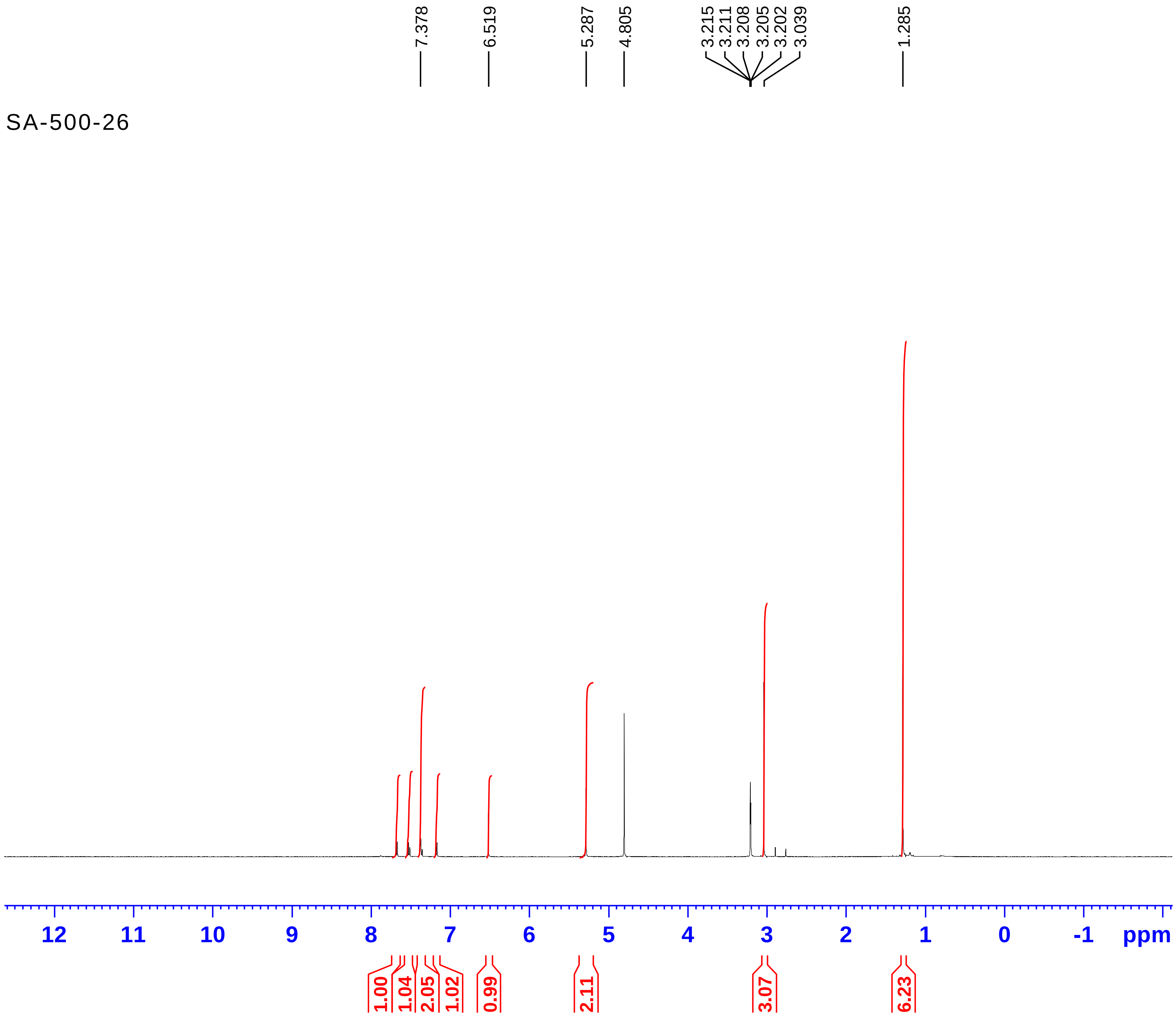

Current Data Parameters

NAME	SA-500-26
EXPNO	10
PROCNO	1

F2 - Acquisition Parameters

Date_	20150122	
Time	15.02	
INSTRUM	spect	
PROBHD	5 mm PABBO BB-	
PULPROG	zg30	
TD	65536	
SOLVENT	MeOD	
NS	16	
DS	0	
SWH	10330.578	Hz
FIDRES	0.157632	Hz
AQ	3.1719425	sec
RG	406	
DW	48.400	usec
DE	11.99	usec
TE	292.3	K
D1	1.00000000	sec
TD0	1	

======== CHANNEL f1 ========

SFO1	500.3030896	MHz
NUC1	1H	
P1	16.25	usec
PLW1	18.33600044	W

F2 - Processing parameters SI 65536

SF	500.3000605	MHz
WDW	EM	
SSB	0	
LB	0.30	Hz
GB	0	
PC	1.00	

17o

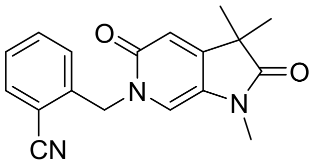

SA-500-26   11   1   Z:\data\SA\nmr

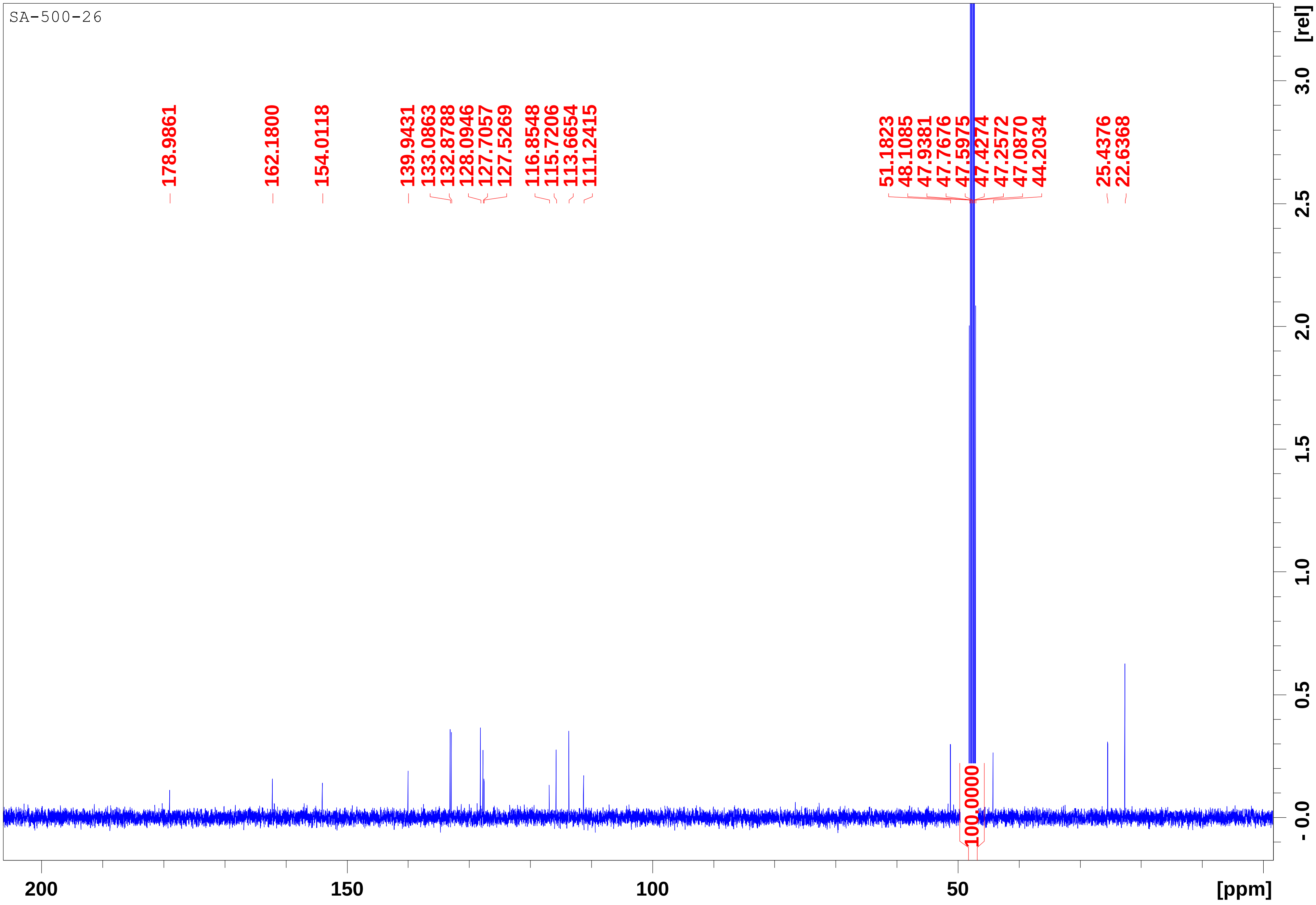

17p

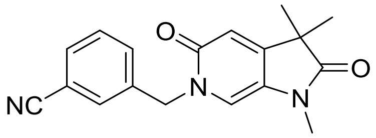

SA-500-32

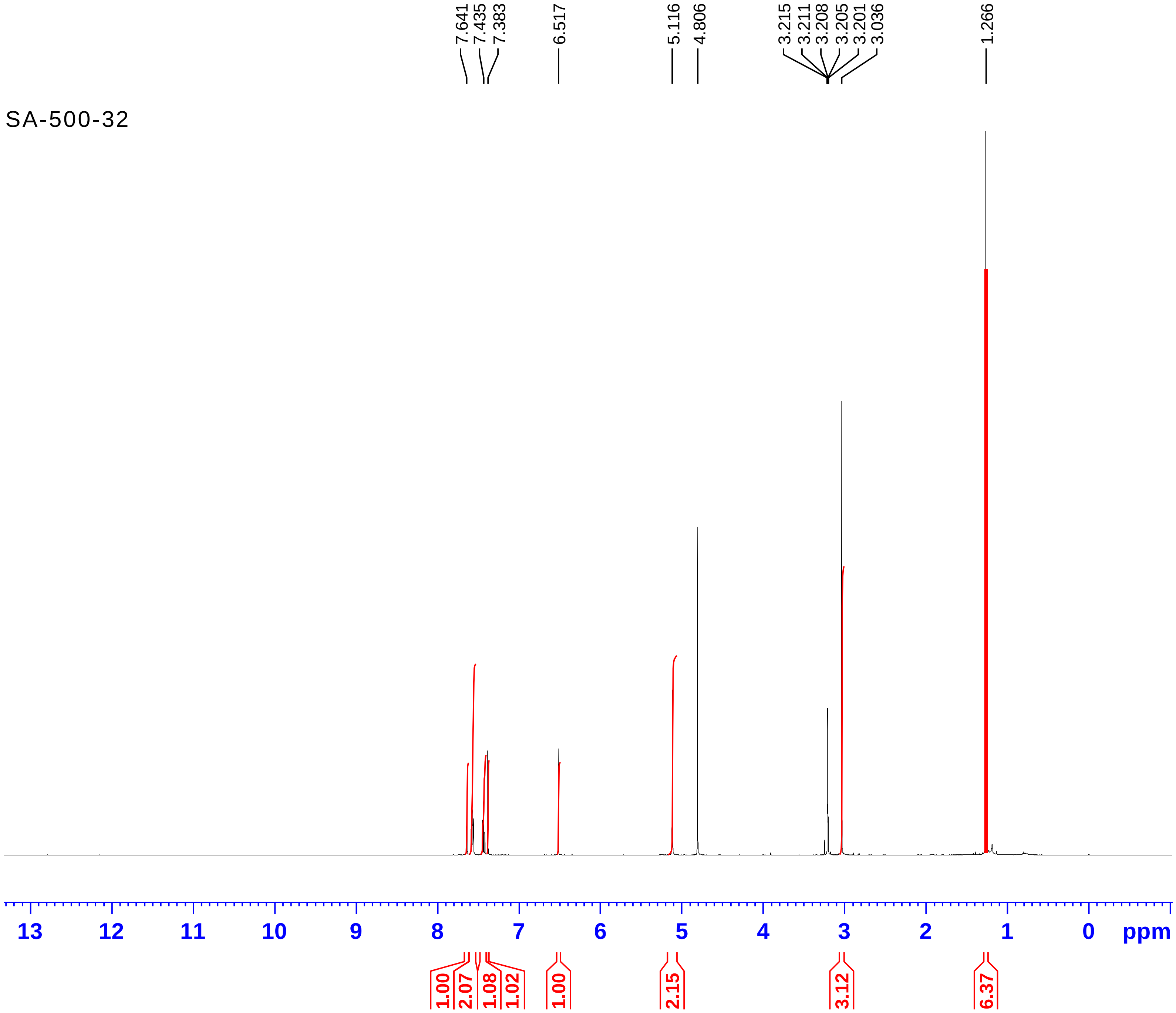

Current Data Parameters

NAME	SA-500-32
EXPNO	10
PROCNO	1

F2 - Acquisition Parameters

Date_	20150122
Time	15.12
INSTRUM	spect
PROBHD	5 mm PABBO BB-
PULPROG	zg30
TD	65536
SOLVENT	MeOD
NS	16
DS	0
SWH	10330.578 Hz
FIDRES	0.157632 Hz
AQ	3.1719425 sec
RG	362
DW	48.400 usec
DE	11.99 usec
TE	292.2 K
D1	1.00000000 sec
TD0	1

======== CHANNEL f1 ========

SFO1	500.3030896 MHz
NUC1	1H
P1	16.25 usec
PLW1	18.33600044 W

F2 - Processing parameters

SI	65536
SF	500.3000606 MHz
WDW	EM
SSB	0
LB	0.30 Hz
GB	0
PC	1.00

17p

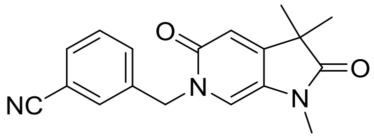

SA-500-32

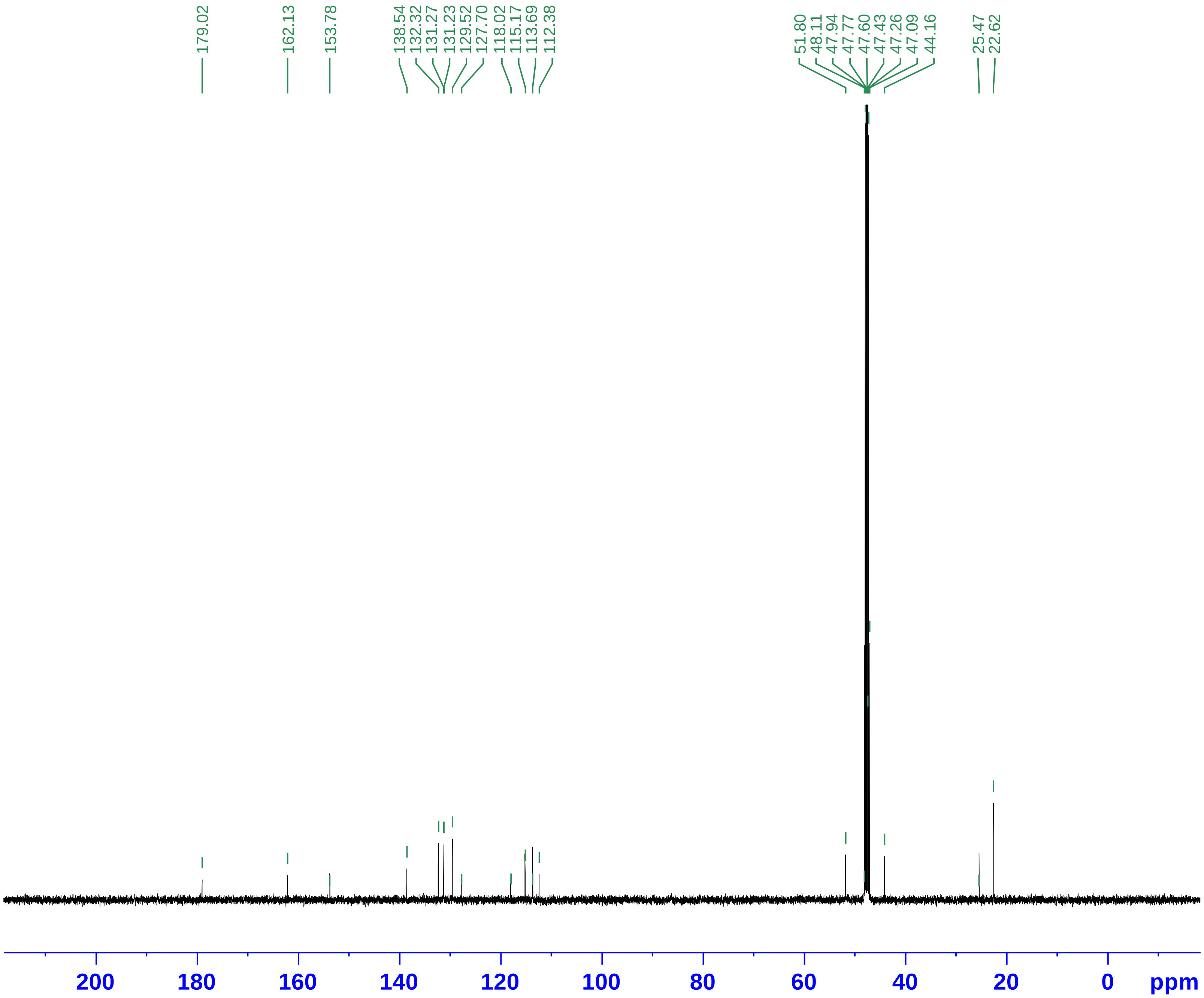

Current Data Parameters
NAME SA-500-32
EXPNO 11
PROCNO 1

F2 - Acquisition Parameters Date_ 20150122
Time 15.49
INSTRUM spect
PROBHD 5 mm PABBO BB-
PULPROG zgpg30
TD 65536
SOLVENT MeOD
NS 256
DS 2
SWH 29761.904 Hz
FIDRES 0.454131 Hz
AQ 1.1010048 sec
RG 456
DW 16.800 usec
DE 7.68 usec
TE 294.9 K
D1 2.00000000 sec
D11 0.03000000 sec
TD0 1

======== CHANNEL f1 ========

SFO1 125.8131151 MHz
NUC1 13C
P1 9.75 usec
PLW1 82.38999939 W

======== CHANNEL f2 ========
SFO2 500.3020012 MHz
NUC2 1H
CPDPRG[2 waltz16
PCPD2 80.00 usec
PLW2 18.33600044 W
PLW12 0.75654000 W
PLW13 0.48418999 W

F2 - Processing Parameters
SI 65536
SF 125.8005350 MHz
WDW EM
SSB 0
LB 1.00 Hz
GB 0
PC 1.40

17q

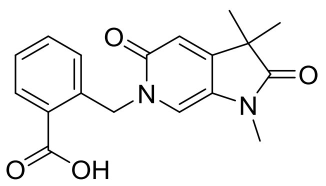

SA-500-44

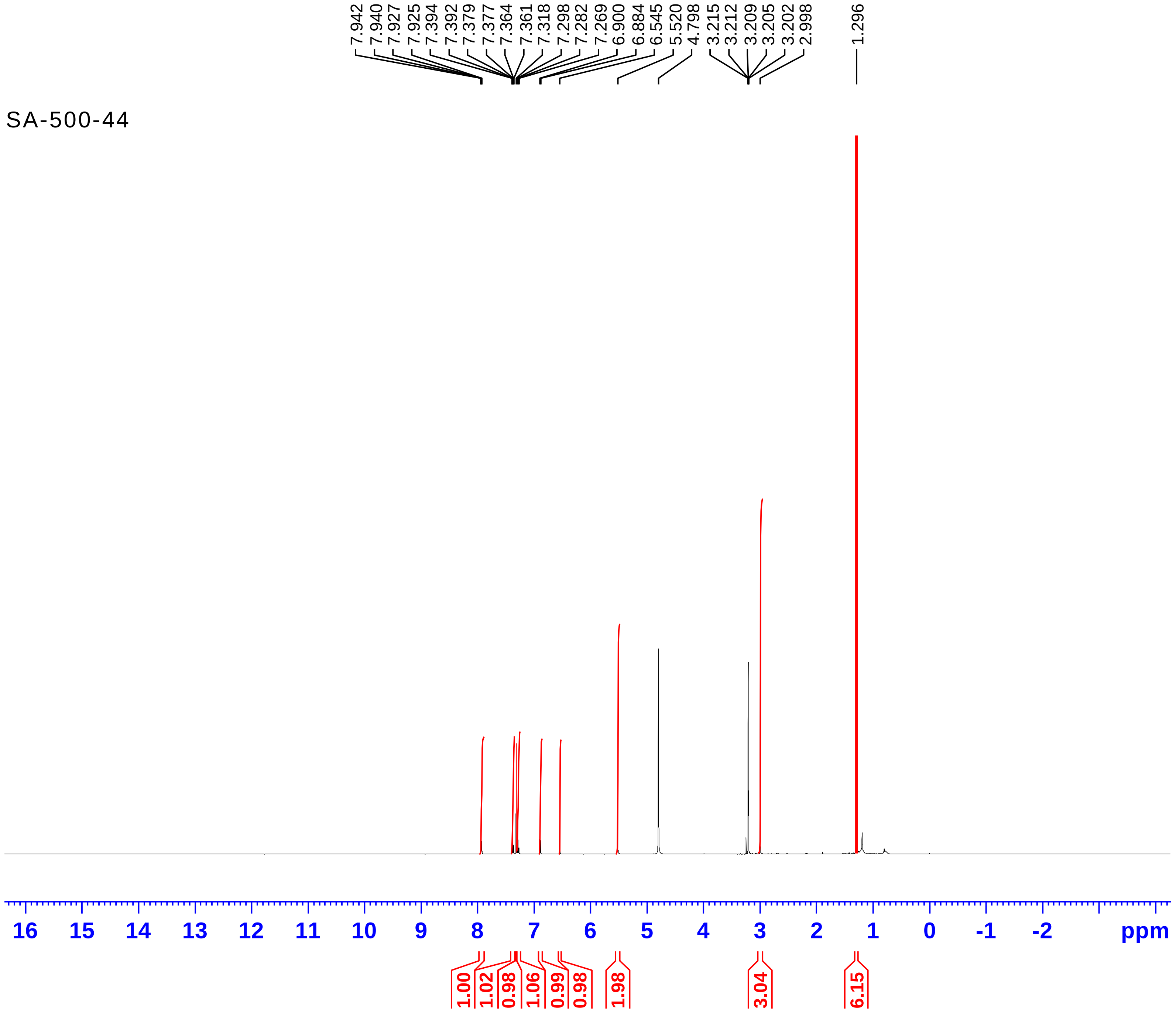

Current Data Parameters

NAME	SA-500-44
EXPNO	10
PROCNO	1

F2 - Acquisition Parameters

Date_	20150128	
Time	10.40	
INSTRUM	spect	
PROBHD	5 mm PABBO BB-	
PULPROG	zg30	
TD	65536	
SOLVENT	MeOD	
NS	16	
DS	0	
SWH	10330.578	Hz
FIDRES	0.157632	Hz
AQ	3.1719425	sec
RG	456	
DW	48.400	usec
DE	11.99	usec
TE	293.7	K
D1	1.00000000	sec
TD0	1	

======== CHANNEL f1 ========

SFO1	500.3030896	MHz
NUC1	1H	
P1	16.25	usec
PLW1	18.33600044	W

F2 - Processing parameters

SI	65536	
SF	500.3000603	MHz
WDW	EM	
SSB	0	
LB	0.30	Hz
GB	0	
PC	1.00	

17q

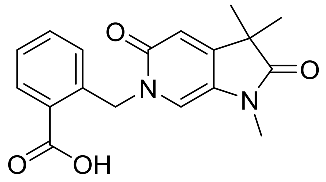

SA-500-44

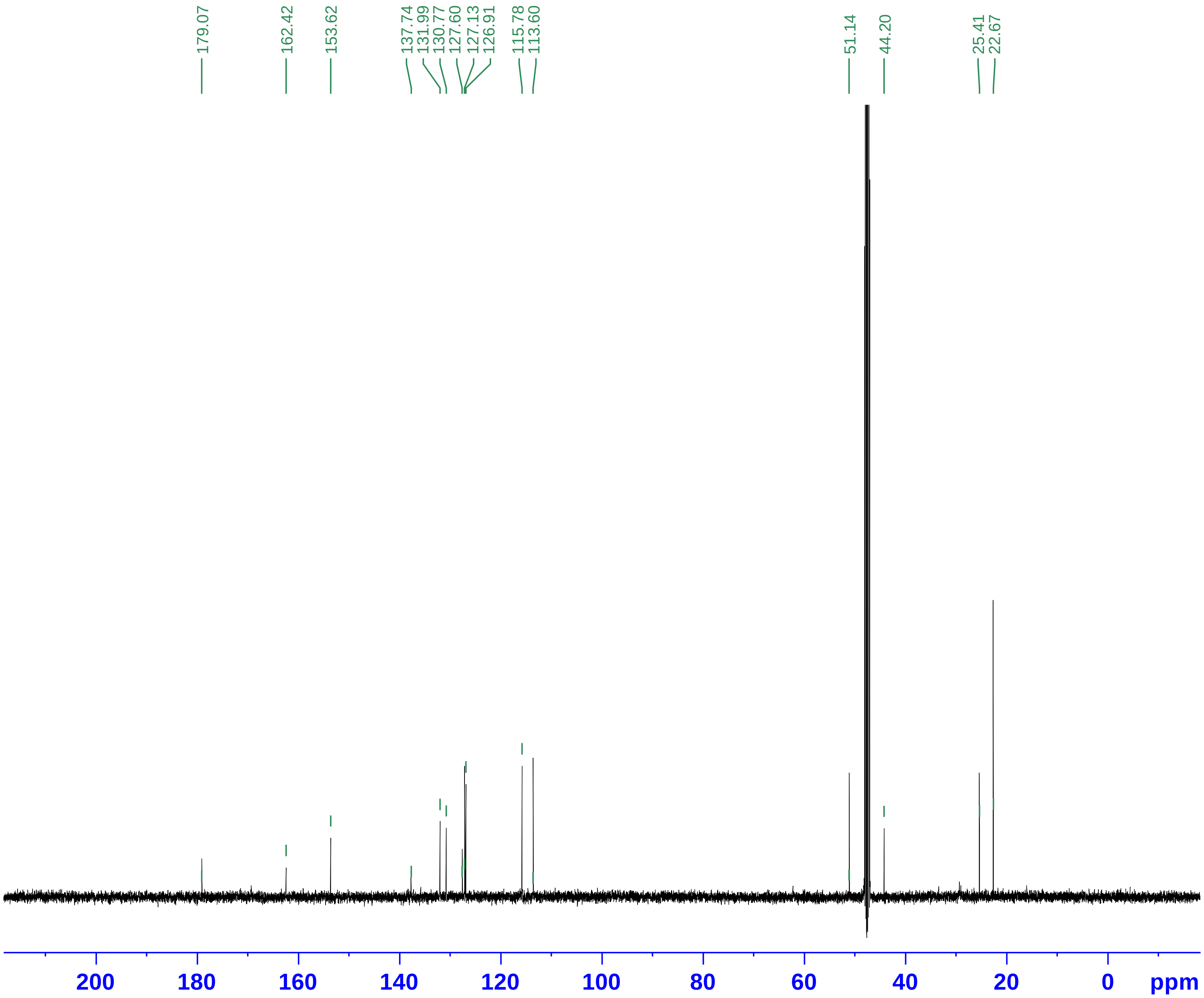

Current Data Parameters
NAME SA-500-44
EXPNO 12
PROCNO 1

F2 - Acquisition Parameters Date_ 20150128
Time 21.14
INSTRUM spect
PROBHD 5 mm PABBO BB-
PULPROG zgpg
TD 26782
SOLVENT MeOD
NS 2048
DS 2
SWH 29761.904 Hz
FIDRES 1.111265 Hz
AQ 0.4499376 sec
RG 724
DW 16.800 usec
DE 7.72 usec
TE 296.4 K
D1 5.00000000 sec
D11 0.03000000 sec
TD0 1

======== CHANNEL f1 ========

SFO1 125.8131151 MHz
NUC1 13C
P1 9.75 usec
PLW1 82.38999939 W

======== CHANNEL f2 ========
SFO2 500.3020012 MHz
NUC2 1H
CPDPRG[2 waltz16
PCPD2 80.00 usec
PLW2 18.33600044 W
PLW12 0.75654000 W
PLW13 0.48418999 W

F2 - Processing Parameters SI 65536
SF 125.8005350 MHz
WDW EM
SSB 0
LB 1.00 Hz
GB 0
PC 1.40

18a

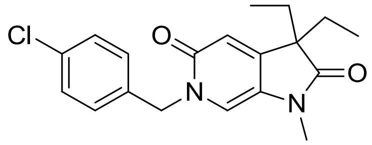

"dcm-494-161-a cdcl3" 10 1 "X:\Old Server\DCM\nmr"

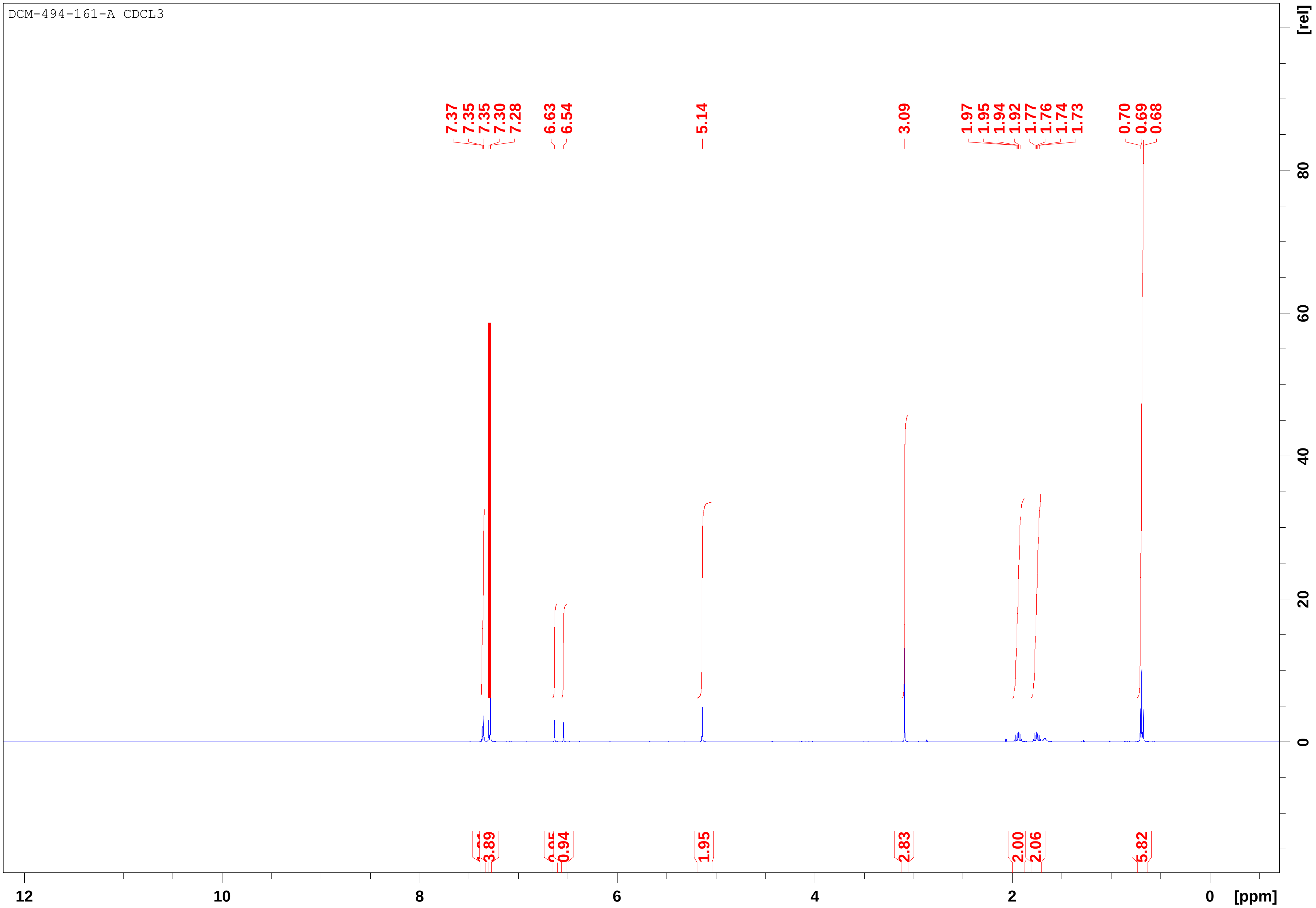

18a

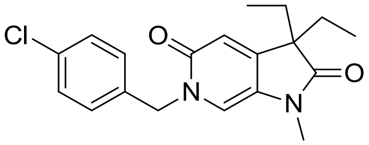

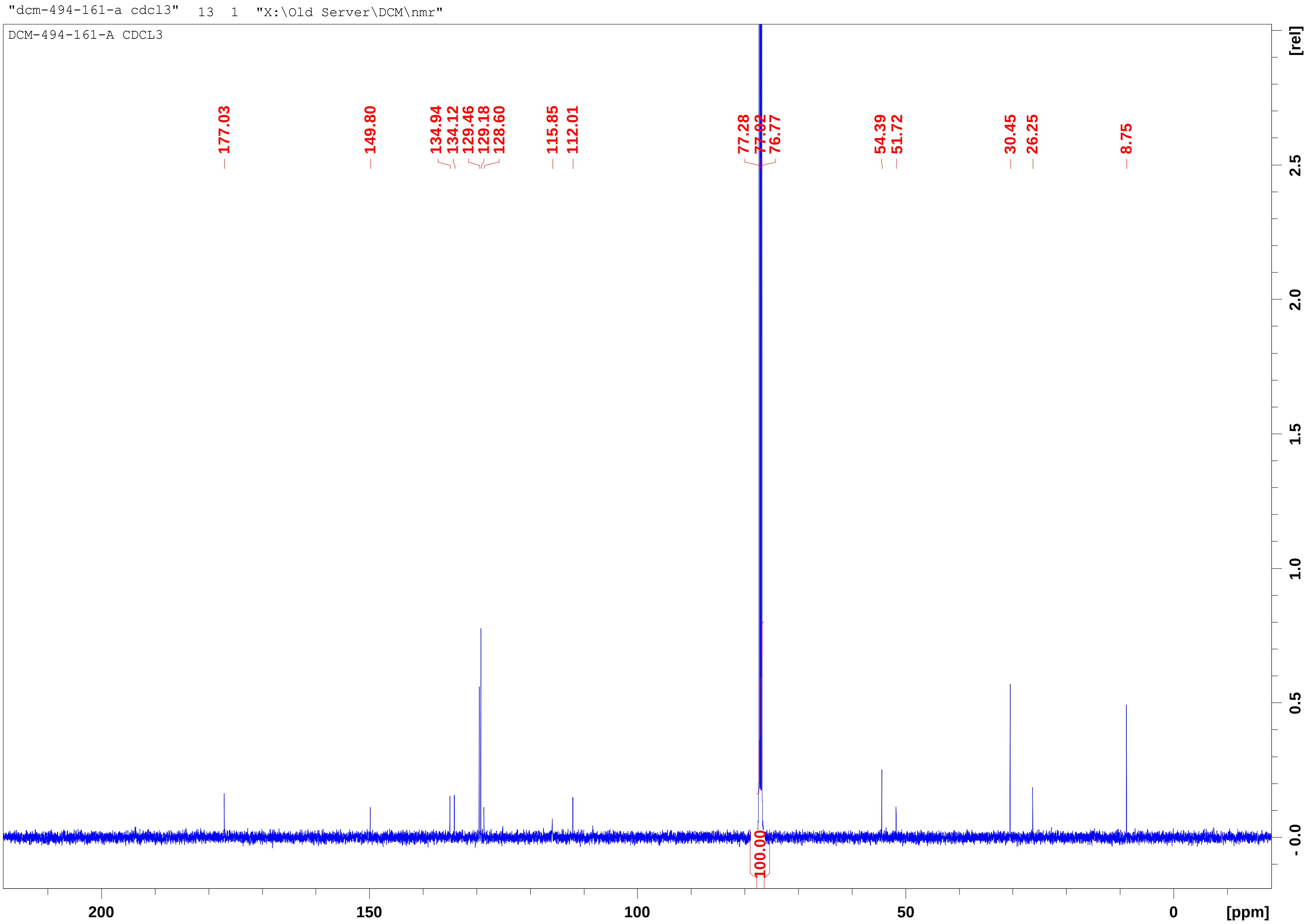

"dcm-494-161-a cdcl3" 13 1 "X:\Old Server\DCM\nmr"
DCM-494-161-A CDCL3
177.03
149.80
134.94
134.12
129.46
129.18
128.60
115.85
112.01
77.28
77.02
76.77
54.39
51.72
30.45
26.25
8.75
100.00
200
150
100
50
0
[ppm]
[rel]
2.5
2.0
1.5
1.0
0.5
-0.0

18b

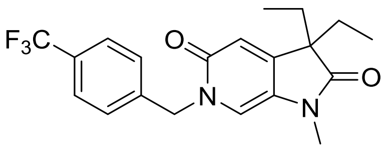

F3C
O
N
O
N

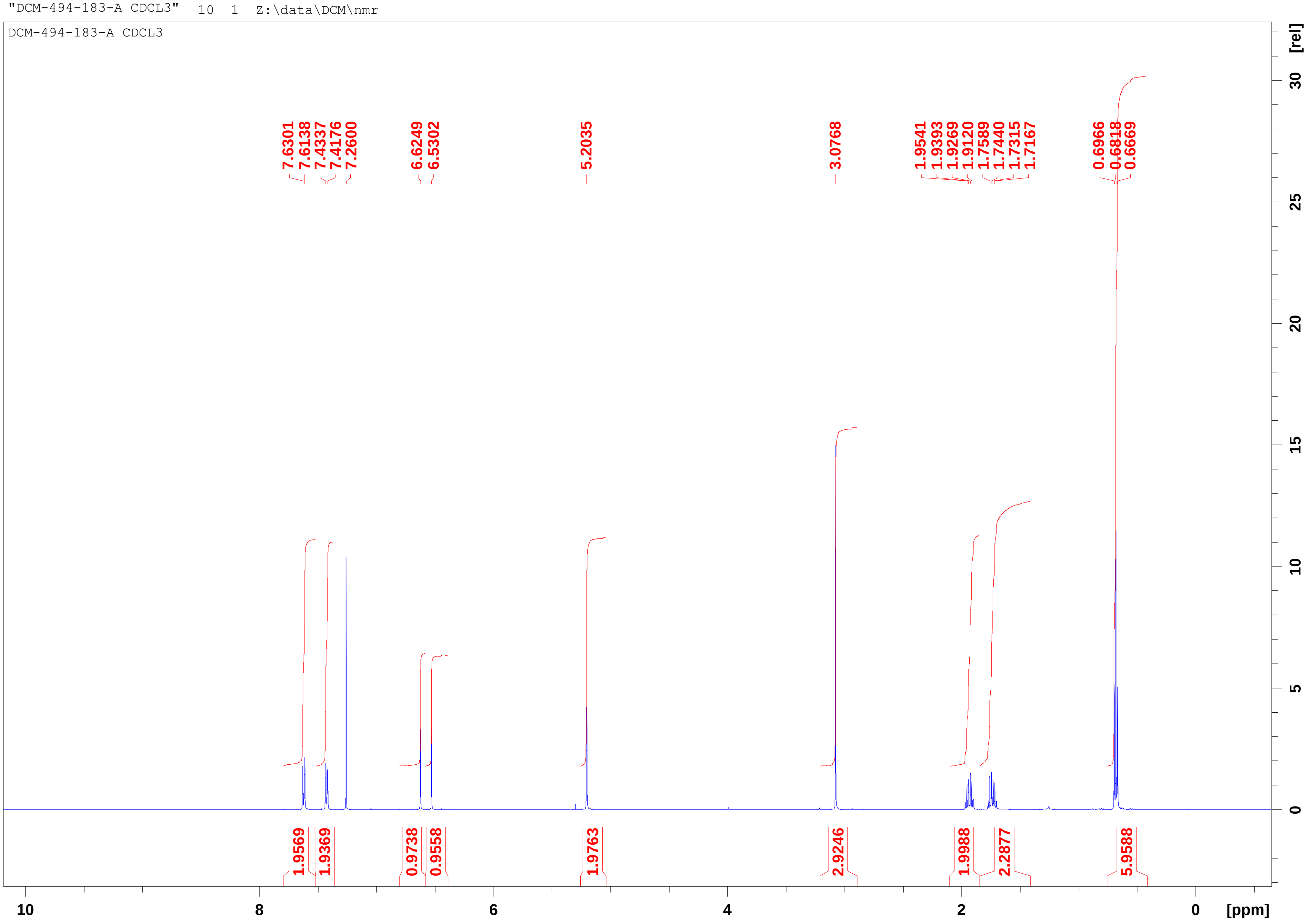

"DCM-494-183-A CDCL3" 10 1 Z:\data\DCM\nmr
DCM-494-183-A CDCL3
7.6301
7.6138
7.4337
7.4176
7.2600
6.6249
6.5302
5.2035
3.0768
1.9541
1.9393
1.9269
1.9120
1.7589
1.7440
1.7315
1.7167
0.6966
0.6818
0.6669
1.9569
1.9369
0.9738
0.9558
1.9763
2.9246
1.9988
2.2877
5.9588
[rel]
[ppm]

18b

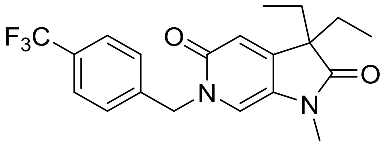

"DCM-494-183-A CDCL3" 11 1 Z:\data\DCM\nmr

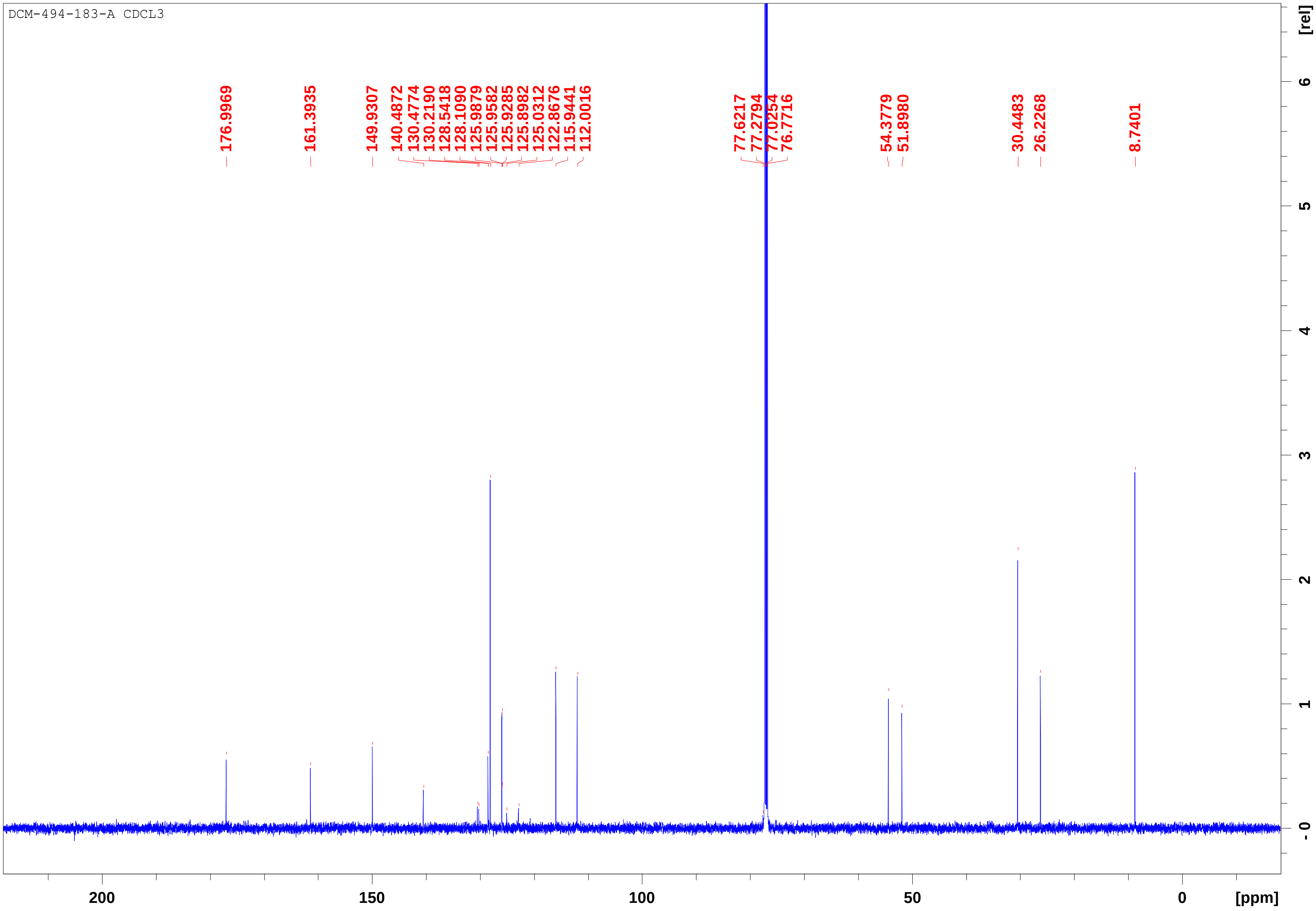

19a

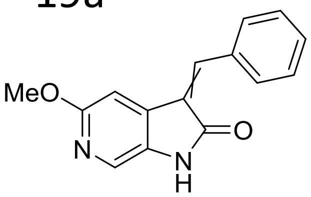

SA-500-90 10 1 "X:\Old Server\SA\nmr"

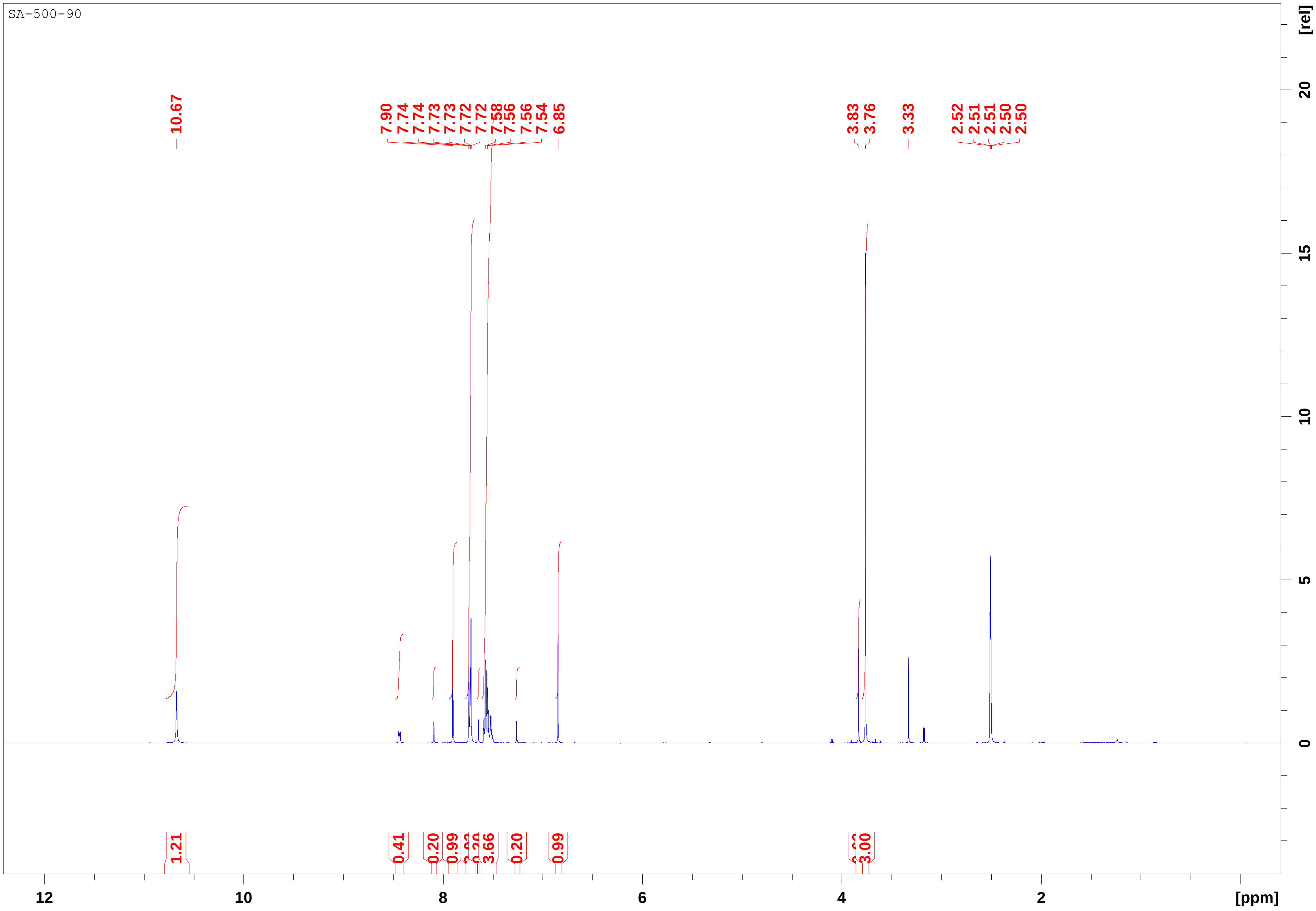

19a

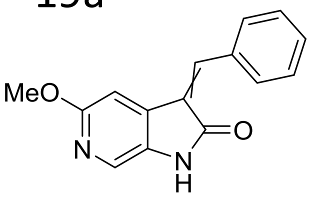
MeO
N
NH
O

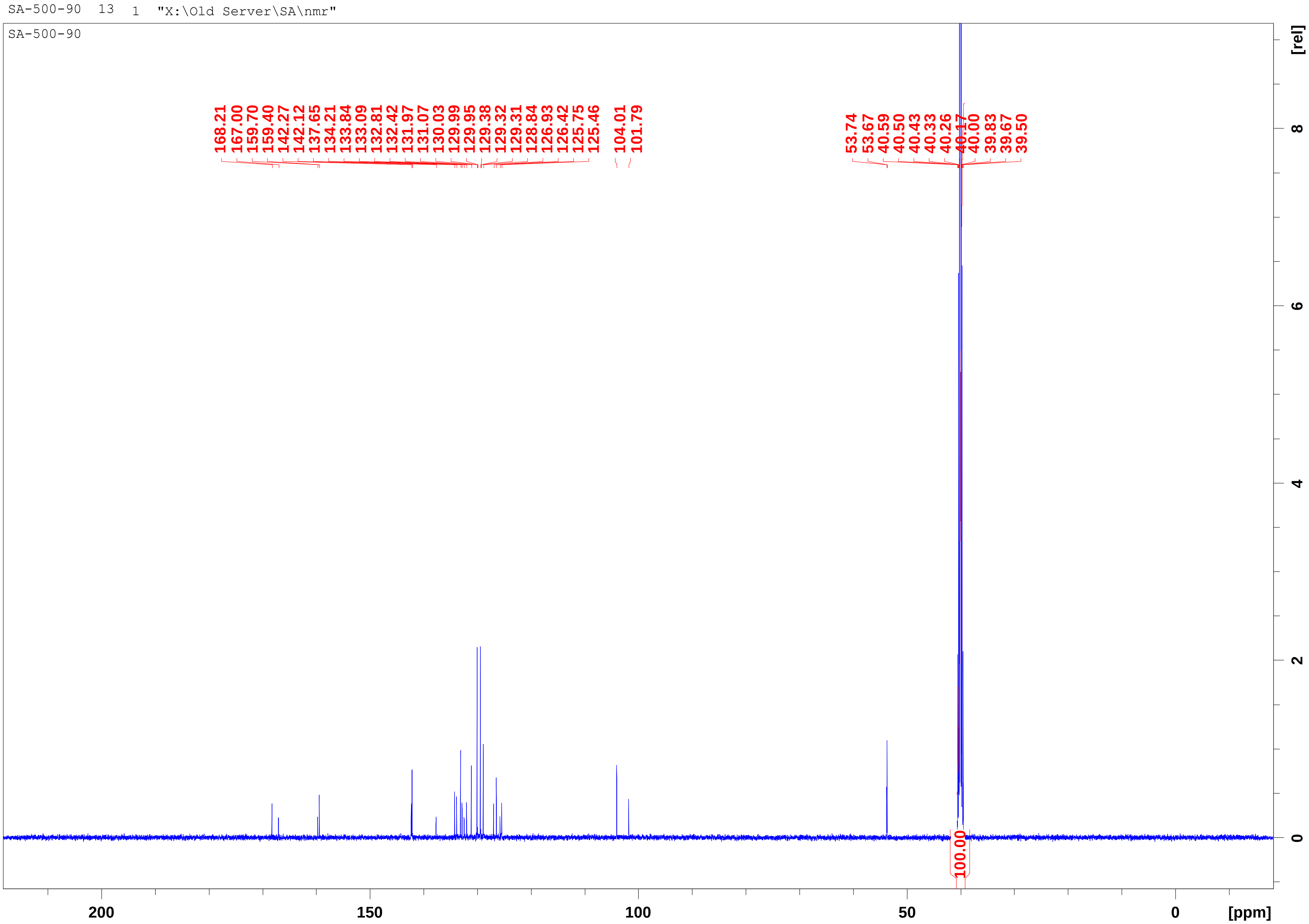
SA-500-90 13 1 "X:\Old Server\SA\nmr"
SA-500-90
168.21
167.00
159.70
159.40
142.27
142.12
137.65
134.21
133.84
133.09
132.81
132.42
131.97
131.07
130.03
129.99
129.95
129.38
129.32
129.31
128.84
126.93
126.42
125.75
125.46
104.01
101.79
53.74
53.67
40.59
40.50
40.43
40.33
40.26
40.17
40.00
39.83
39.67
39.50
100.00
[rel]
8
6
4
2
0
200
150
100
50
0
[ppm]

19b

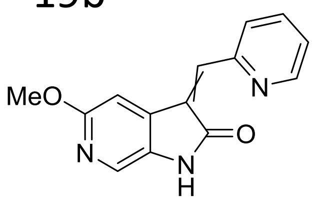
MeO
N
N
H
O
N

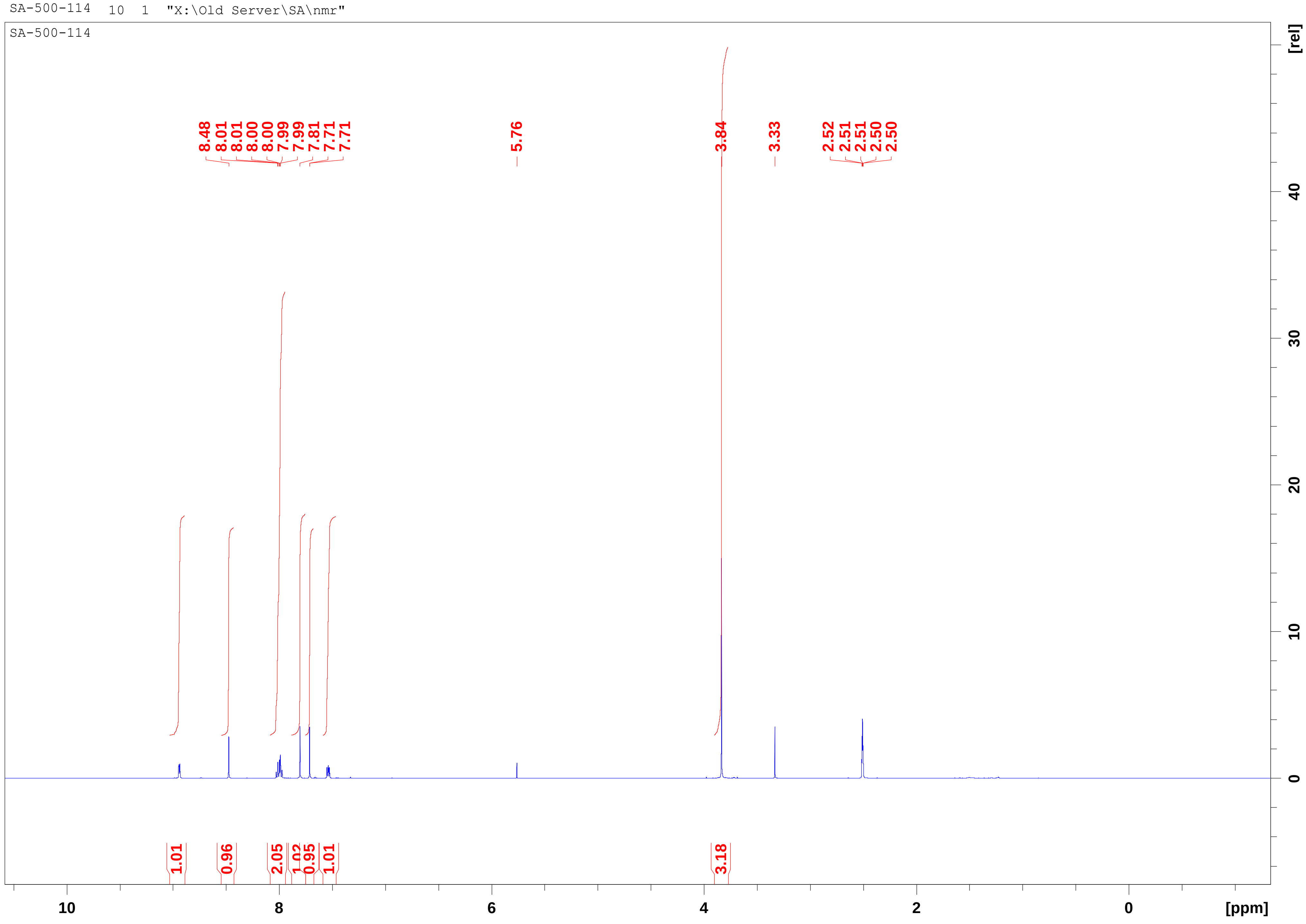
SA-500-114 10 1 "X:\Old Server\SA\nmr"
SA-500-114
8.48
8.01
8.01
8.00
8.00
7.99
7.81
7.71
7.71
5.76
3.84
3.33
2.52
2.51
2.51
2.50
2.50
1.01
0.96
2.05
1.02
0.95
1.01
3.18
[rel]
[ppm]

19b

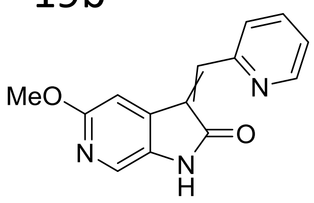

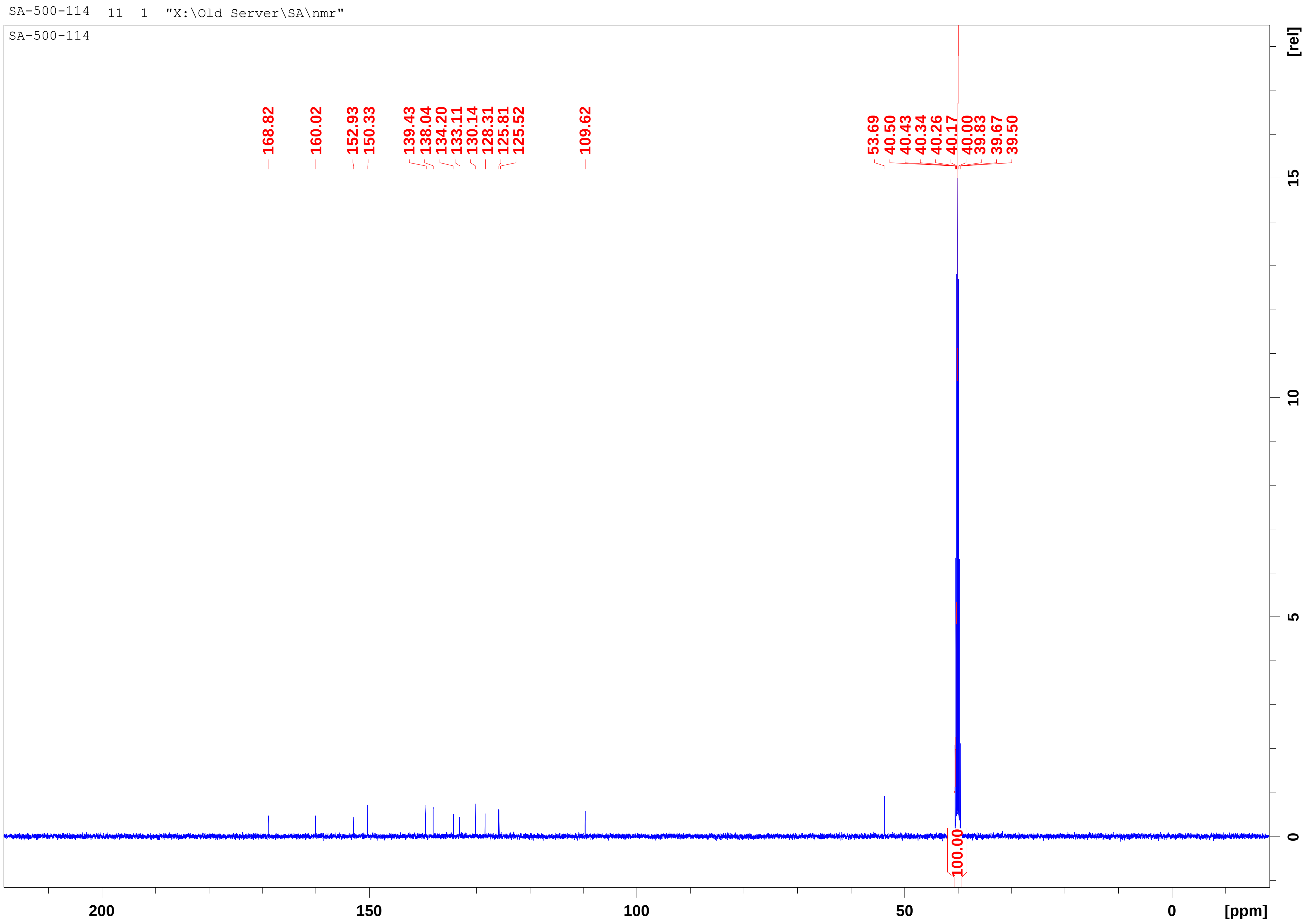

SA-500-114 11 1 "X:\Old Server\SA\nmr"
SA-500-114
168.82
160.02
152.93
150.33
139.43
138.04
134.20
133.11
130.14
128.31
125.81
125.52
109.62
53.69
40.50
40.43
40.34
40.26
40.17
40.00
39.83
39.67
39.50
100.00
[rel]
15
10
5
0
200
150
100
50
0
[ppm]

19c

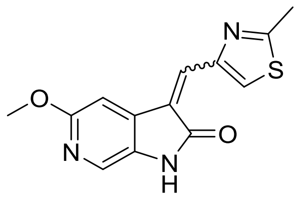

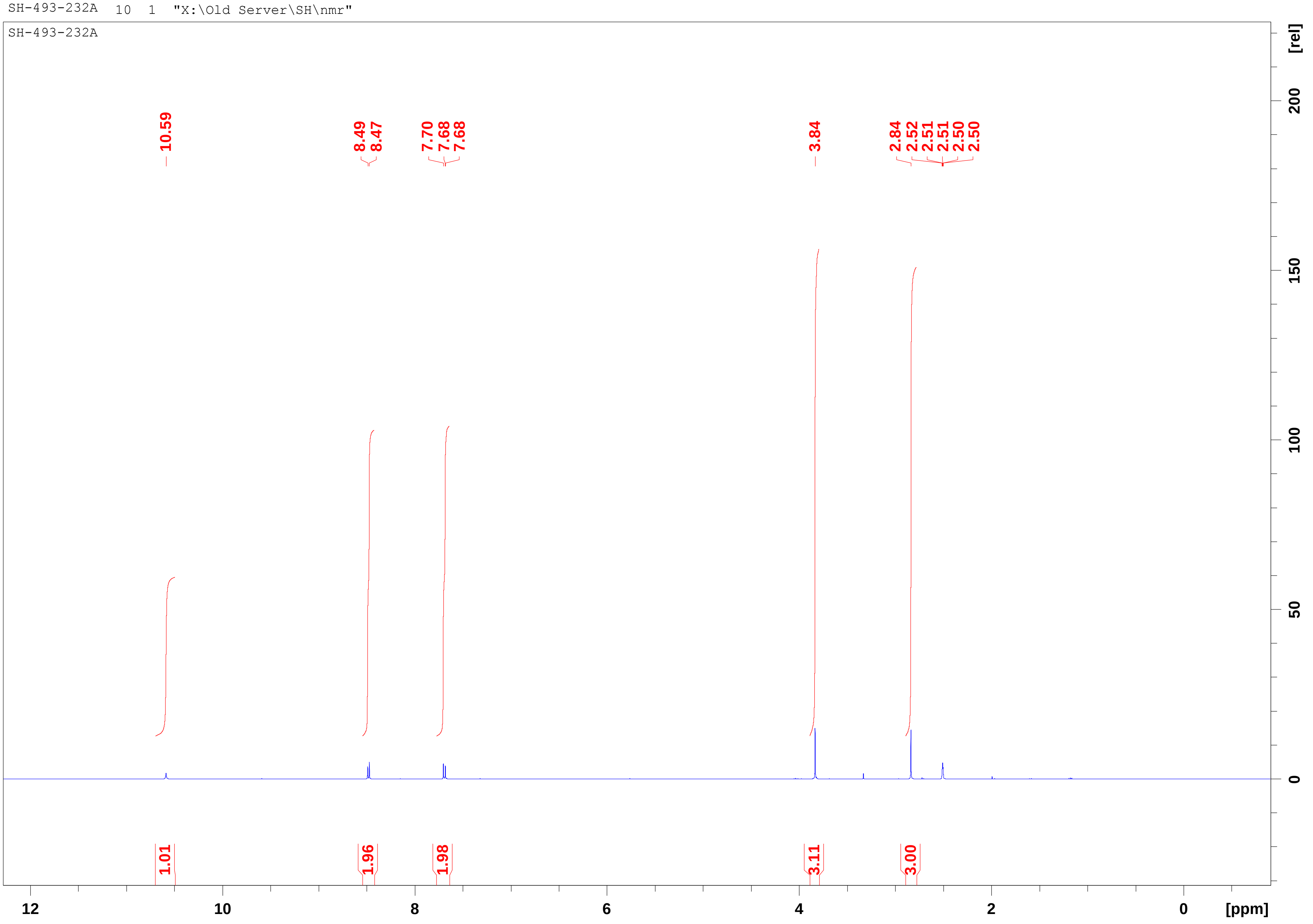

SH-493-232A 10 1 "X:\Old Server\SH\nmr"
SH-493-232A
10.59
8.49
8.47
7.70
7.68
7.68
3.84
2.84
2.52
2.51
2.51
2.50
2.50
1.01
1.96
1.98
3.11
3.00
[rel]
200
150
100
50
0
12
10
8
6
4
2
0
[ppm]

19c

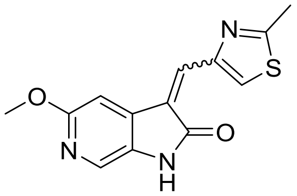

SH-493-232A 11 1 "X:\Old Server\SH\nmr"

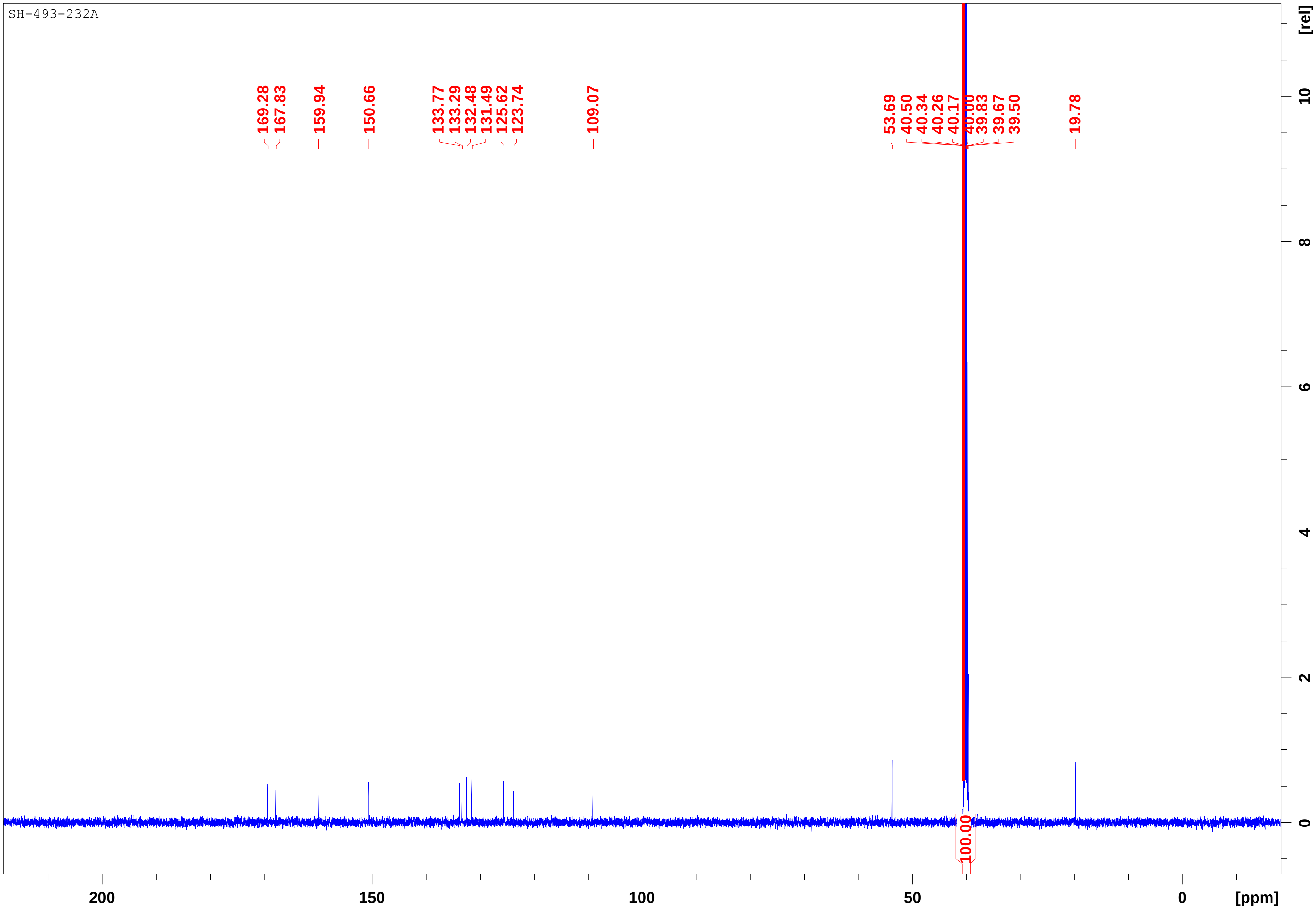

19d

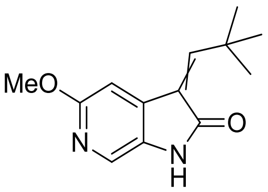

"DCM-494-225-A CDCL3" 10 1 X:\data\DCM\nmr

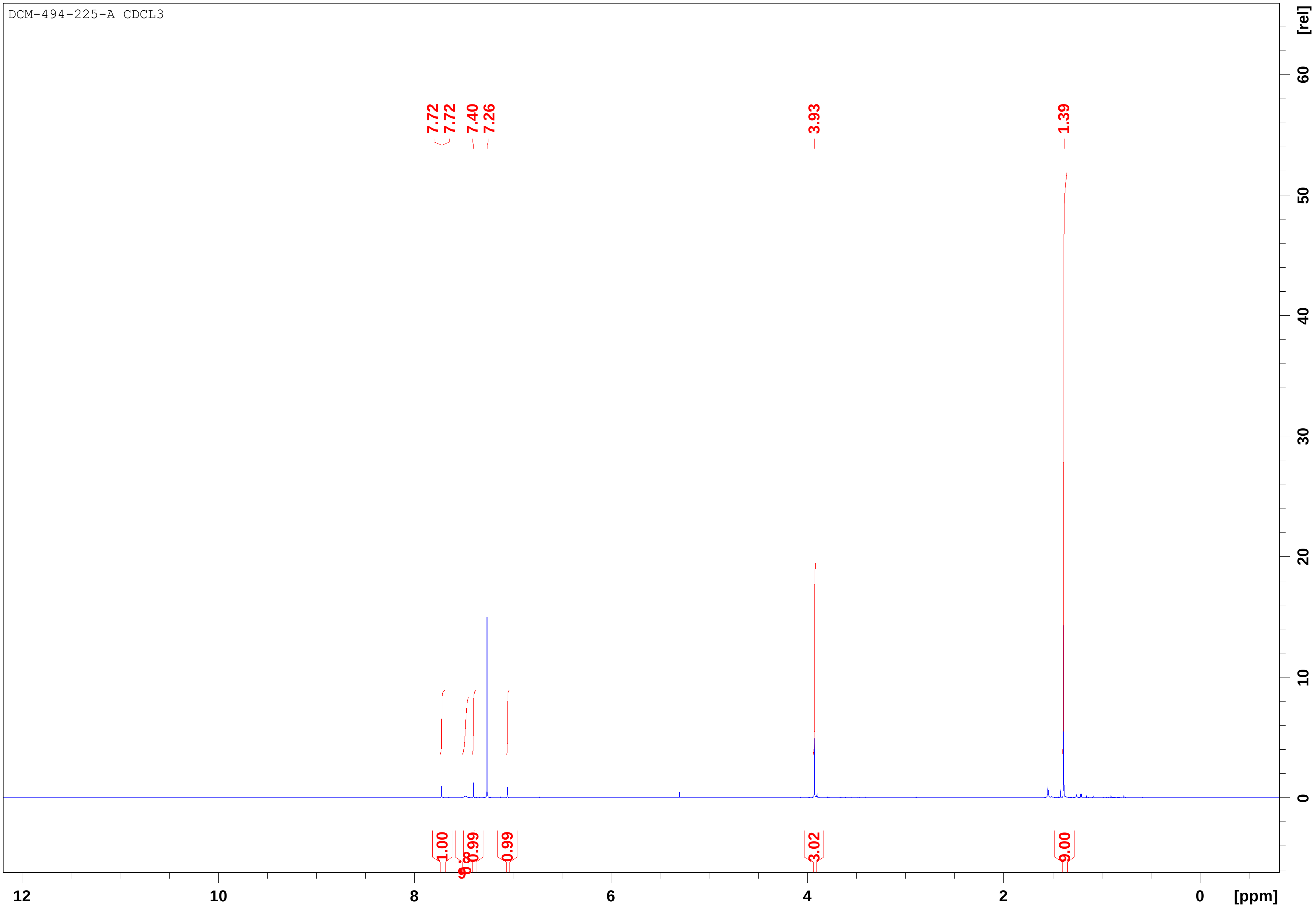

19d

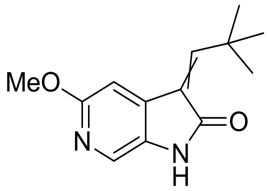
MeO
N
N
H
O

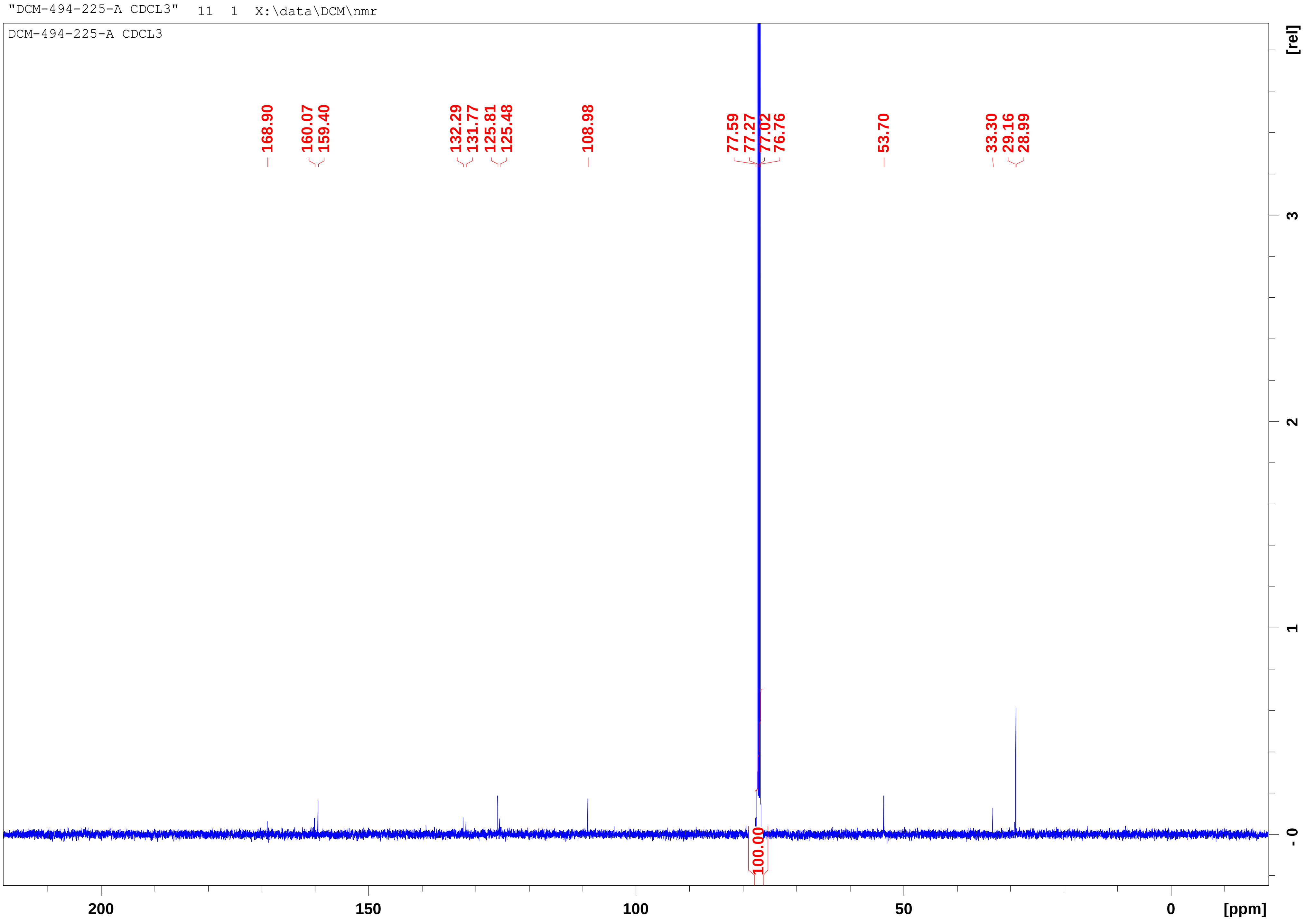
"DCM-494-225-A CDCL3" 11 1 X:\data\DCM\nmr
DCM-494-225-A CDCL3
168.90
160.07
159.40
132.29
131.77
125.81
125.48
108.98
77.59
77.27
77.02
76.76
53.70
33.30
29.16
28.99
100.00
200
150
100
50
0
[ppm]
[rel]
3
2
1
0

19e

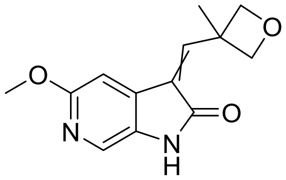

SA-500-152 10 1 "X:\Old Server\SA\nmr"

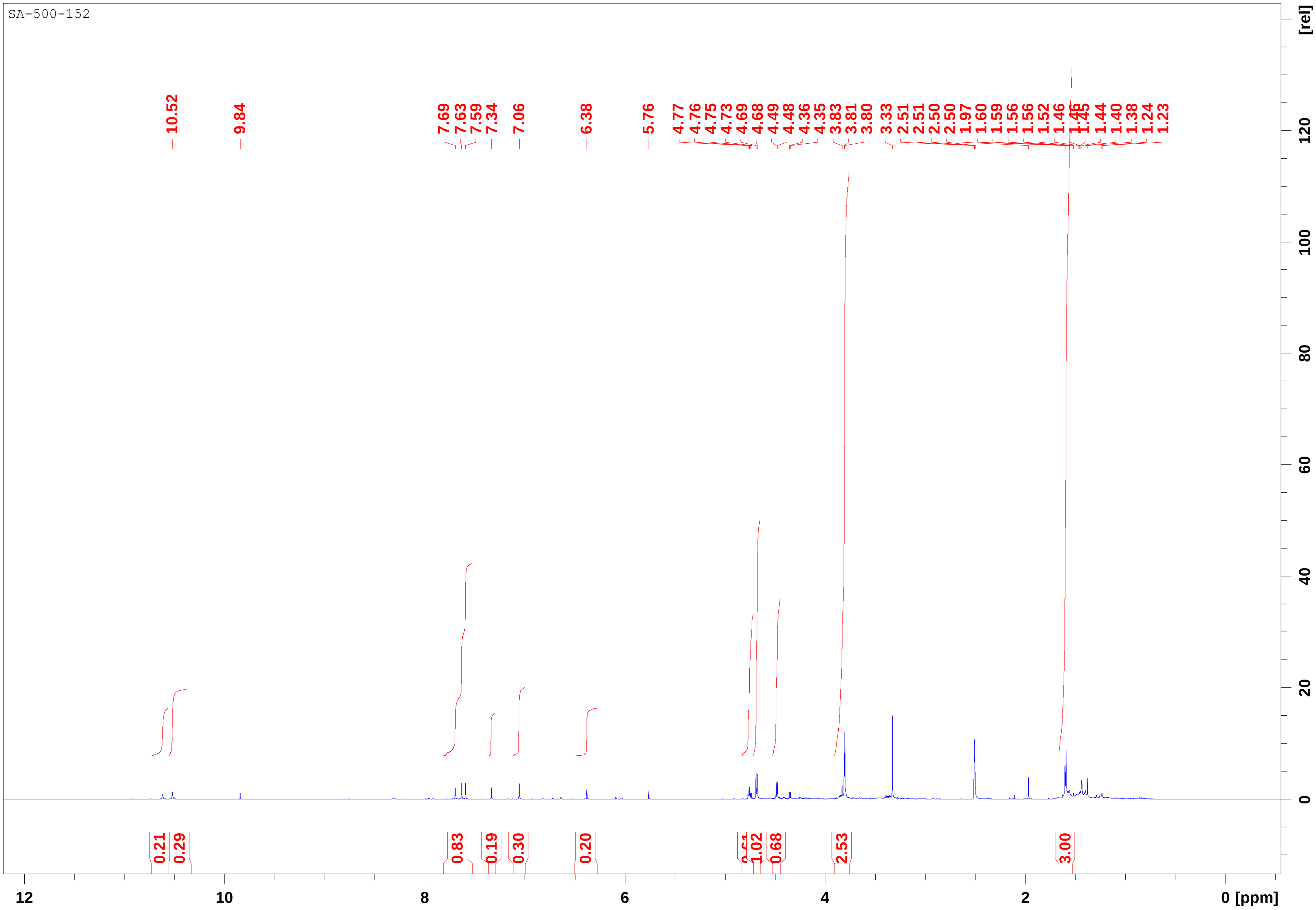

19e

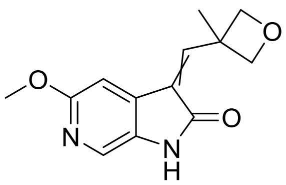

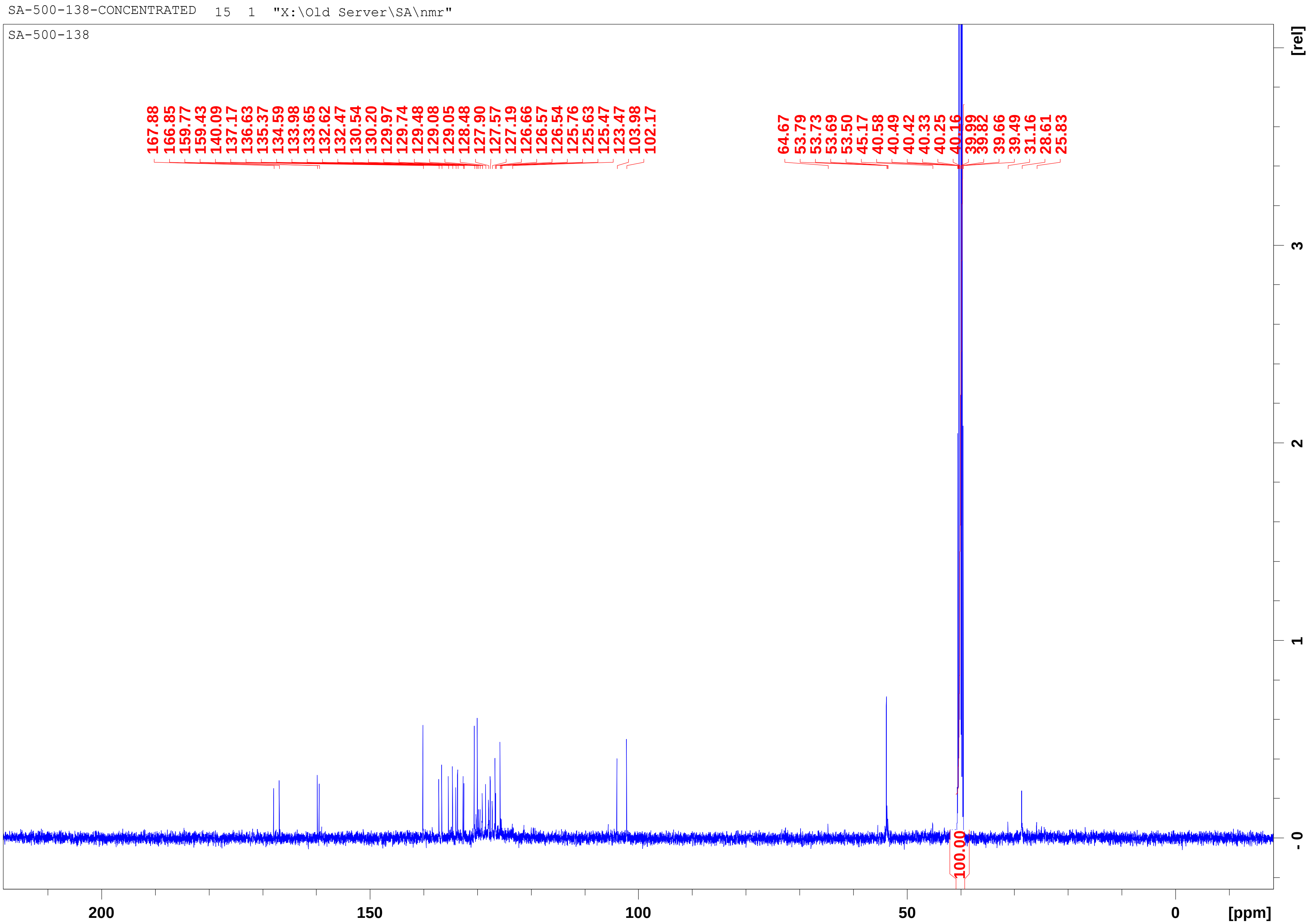
SA-500-138-CONCENTRATED 15 1 "X:\Old Server\SA\nmr"
SA-500-138
167.88
166.85
159.77
159.43
140.09
137.17
136.63
135.37
134.59
133.98
133.65
132.62
132.47
130.54
130.20
129.97
129.74
129.48
129.08
129.05
128.48
127.90
127.57
127.19
126.66
126.57
126.54
125.76
125.63
125.47
123.47
103.98
102.17
64.67
53.79
53.73
53.69
53.50
45.17
40.58
40.49
40.42
40.33
40.25
40.16
39.99
39.82
39.66
39.49
31.16
28.61
25.83
100.00
200
150
100
50
0
[ppm]
[rel]
3
2
1
0

19f

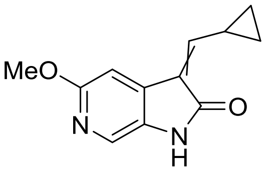

"DCM-494-193-A CDCL3" 10 1 "X:\Old Server\DCM\nmr"

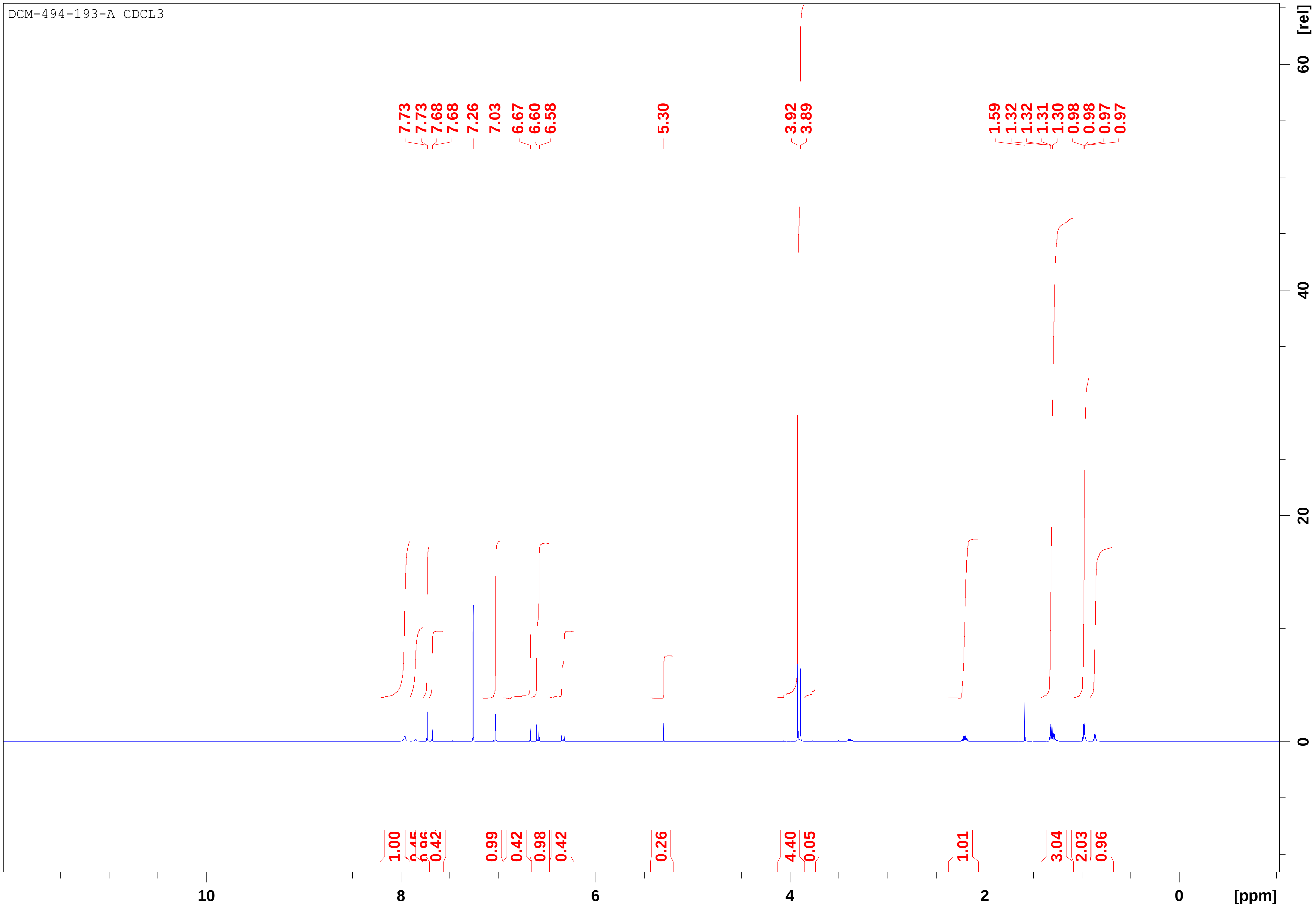

20d

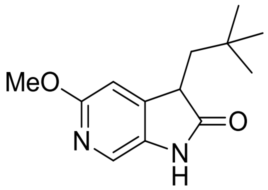

"DCM-494-228-A CDCL3" 10 1 X:\data\DCM\nmr

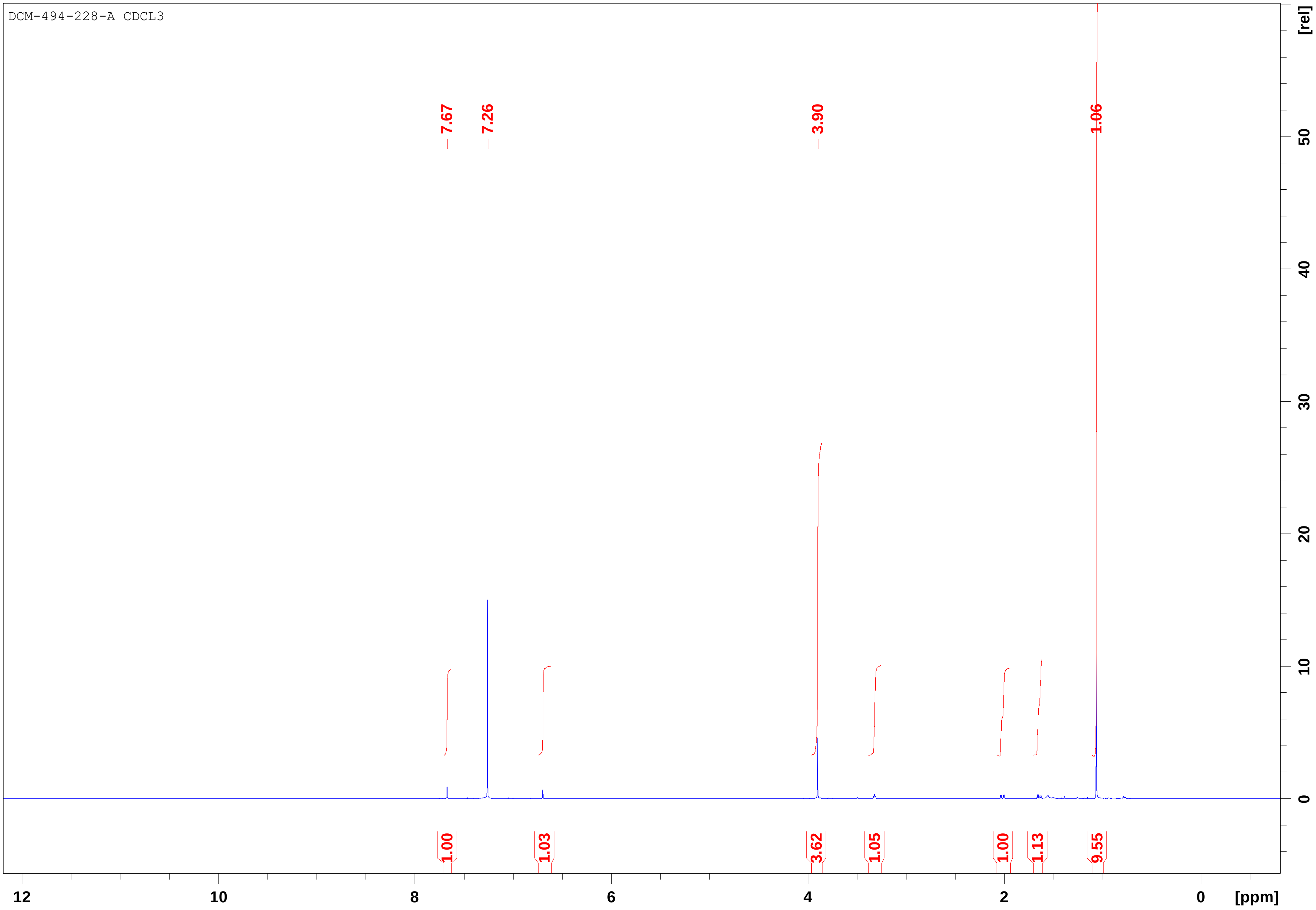

20f

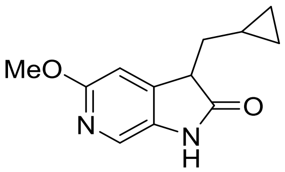

"DCM-494-199-CR CDCL3" 10 1 "X:\Old Server\DCM\nmr"

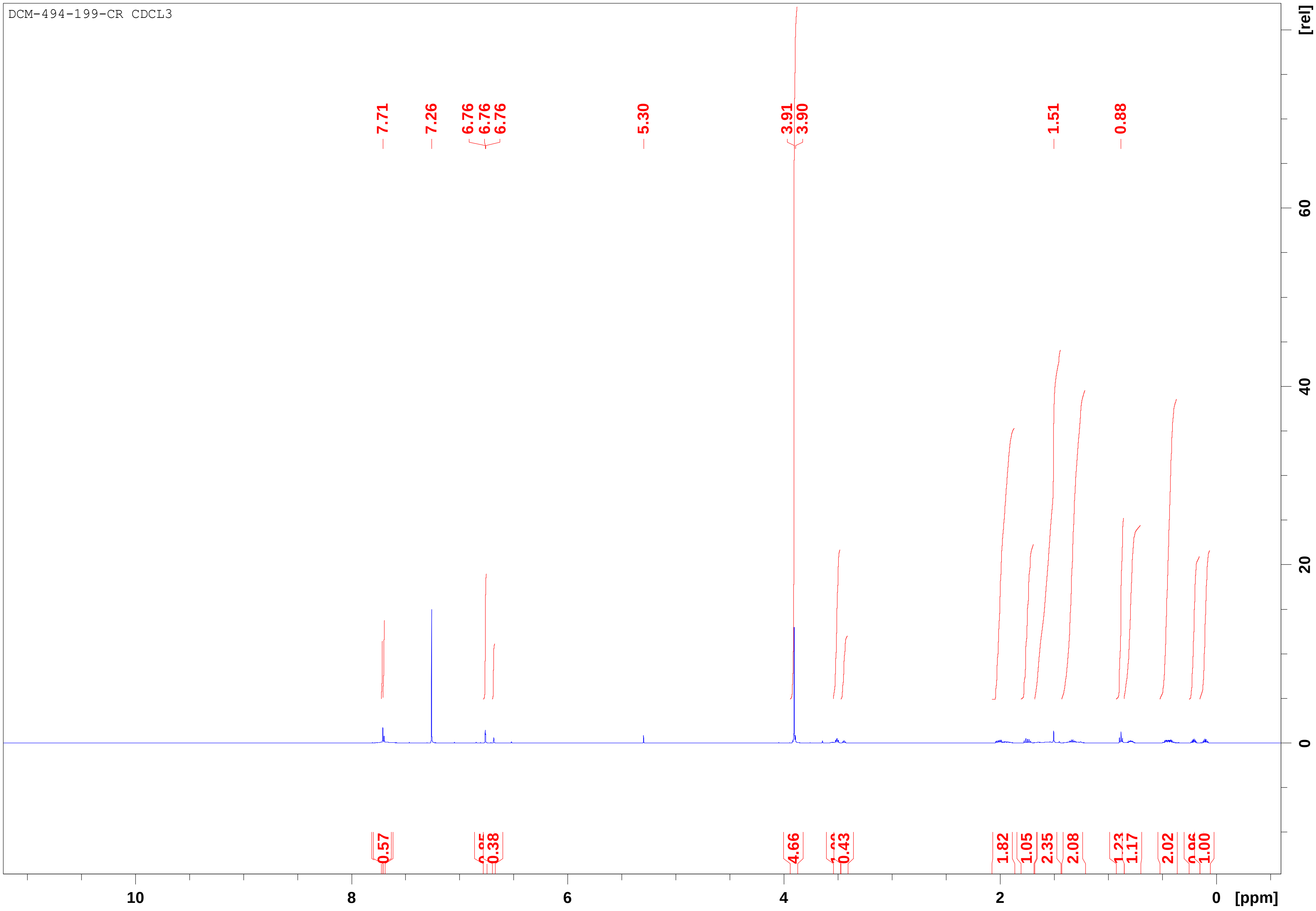

20f

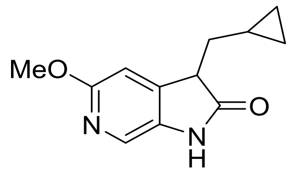

"DCM-494-199 CDCL3" 10 1 "X:\Old Server\DCM\nmr"

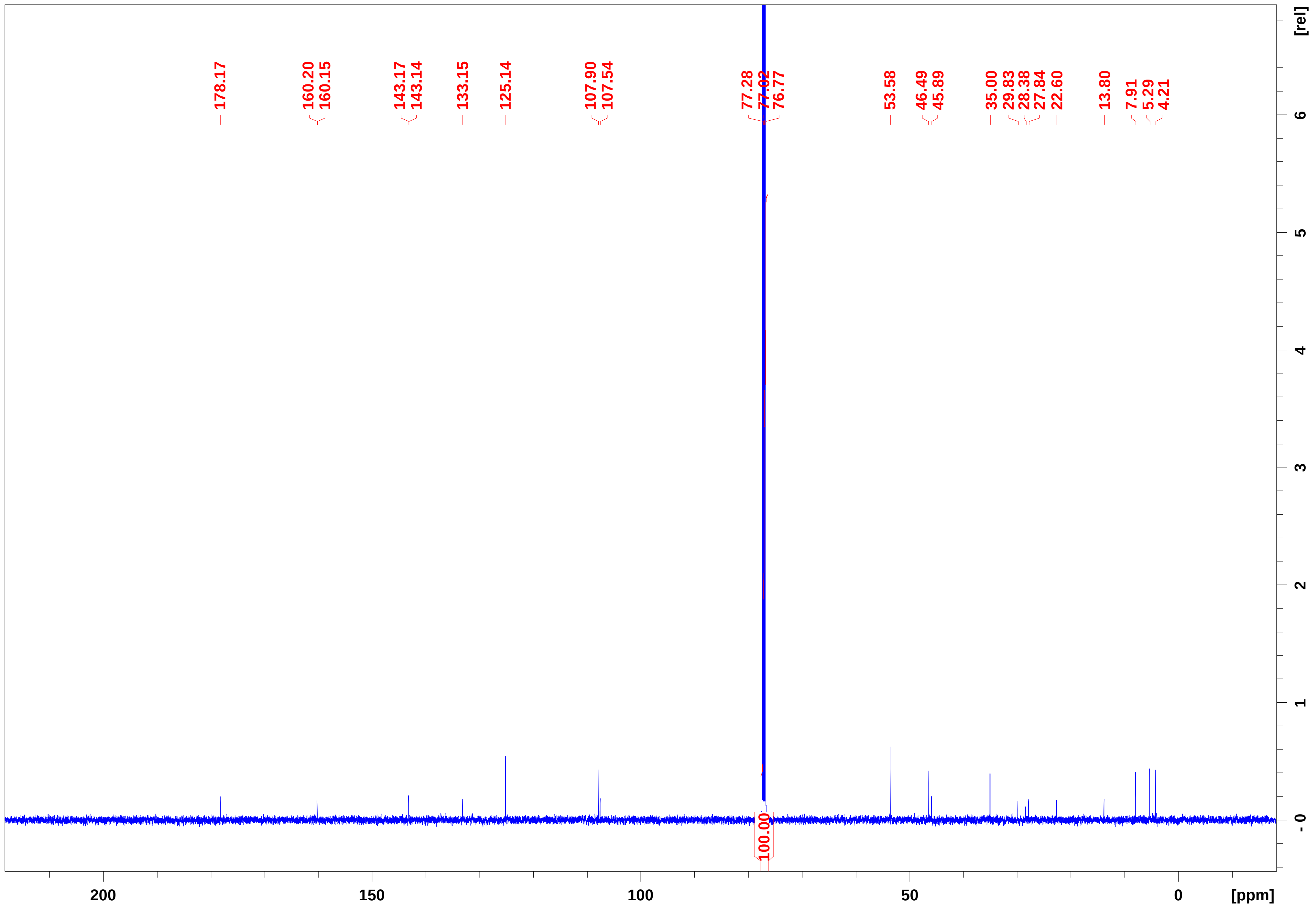

21a

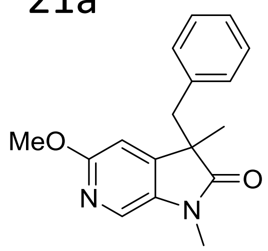

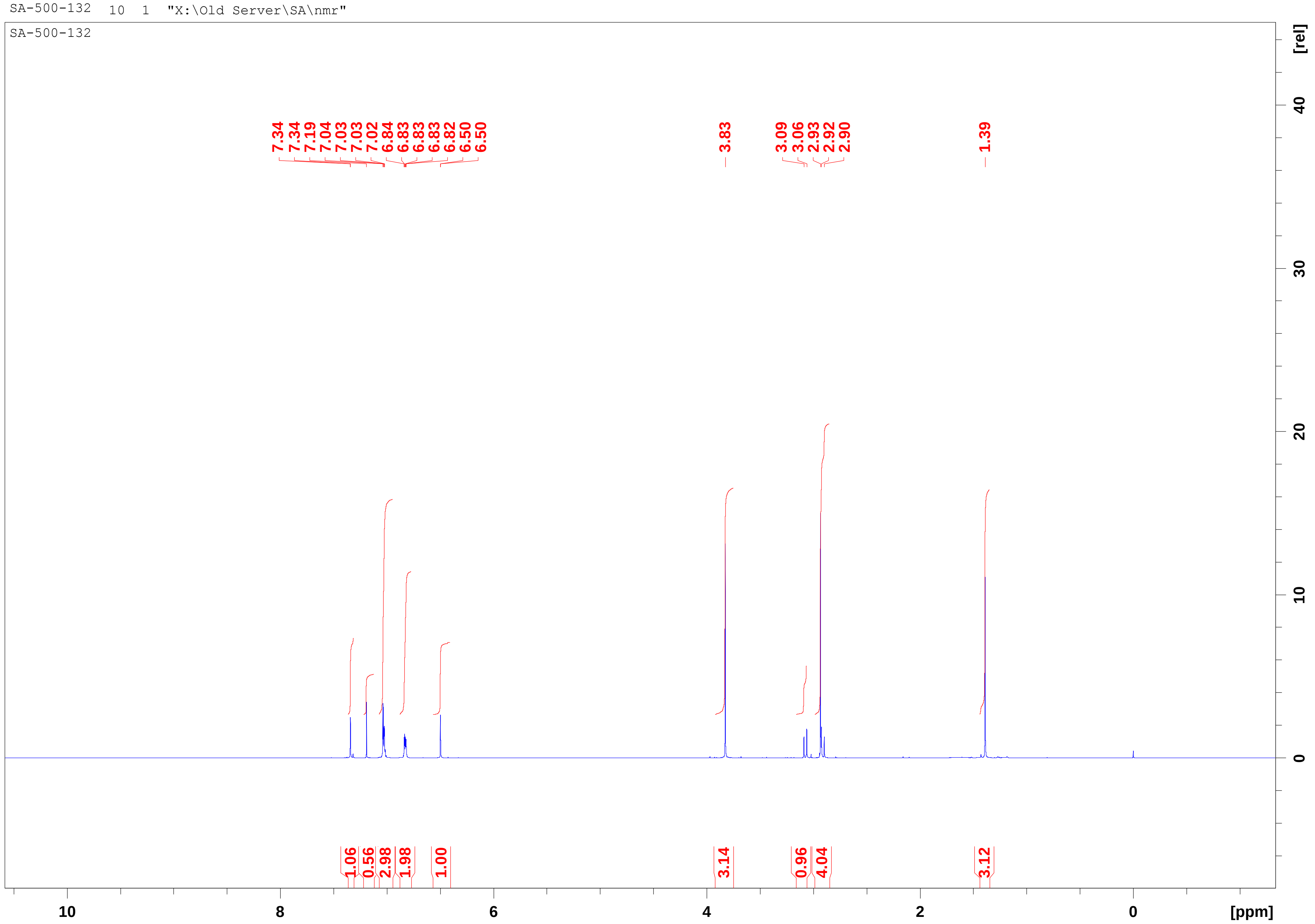
SA-500-132 10 1 "X:\Old Server\SA\nmr"
SA-500-132
7.34
7.34
7.19
7.04
7.03
7.03
7.02
6.84
6.83
6.83
6.83
6.82
6.50
6.50
3.83
3.09
3.06
2.93
2.92
2.90
1.39
1.06
0.56
2.98
1.98
1.00
3.14
0.96
4.04
3.12
[rel]
[ppm]

21a

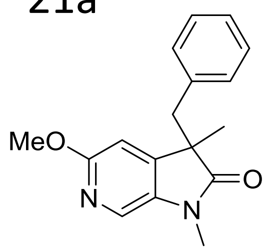

SA-500-132 11 1 "X:\Old Server\SA\nmr"

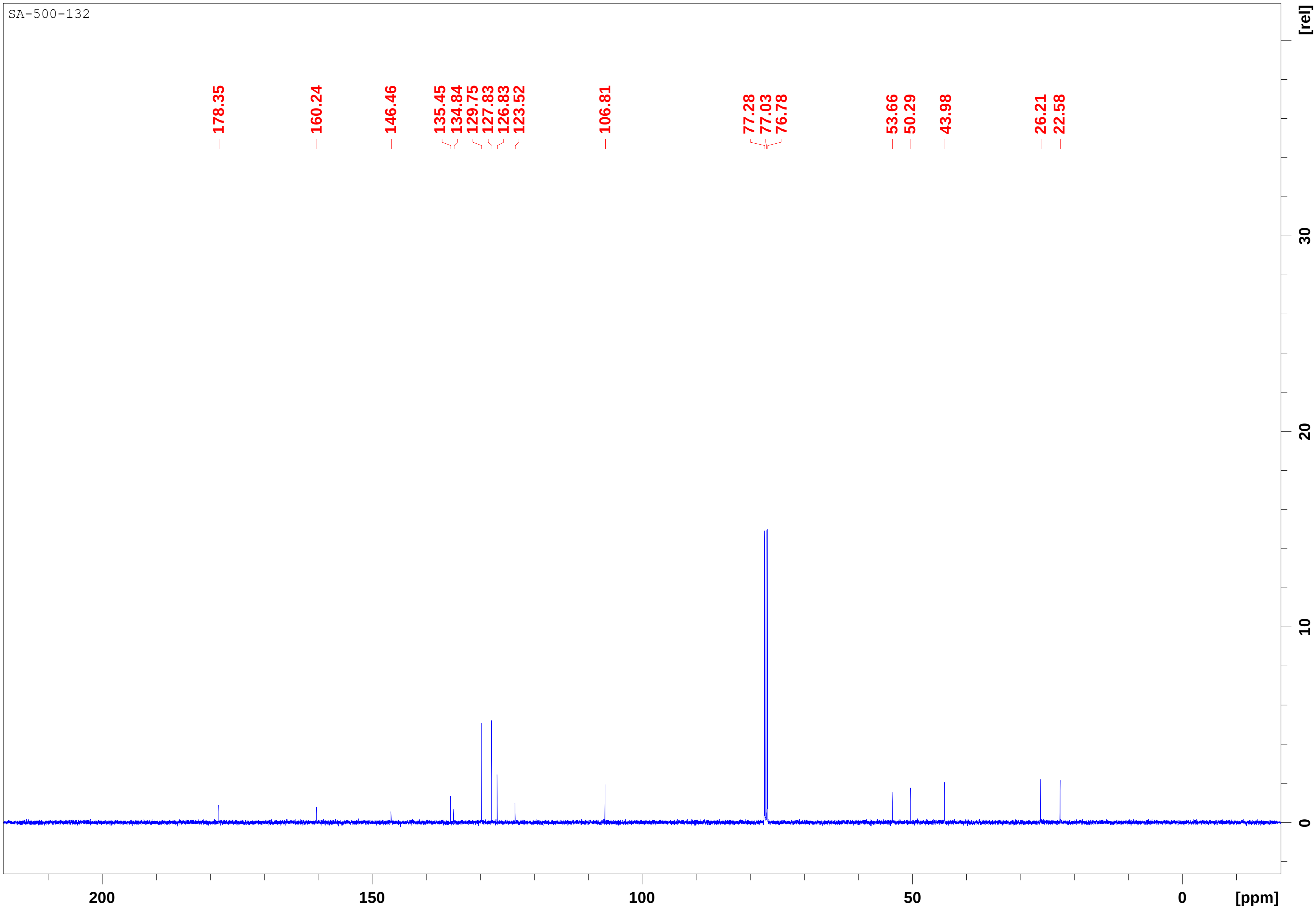

21b

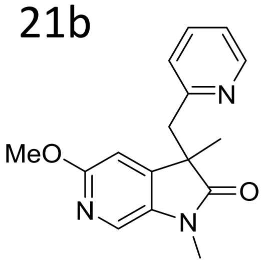

SA-500-122 10 1 "X:\Old Server\SA\nmr"

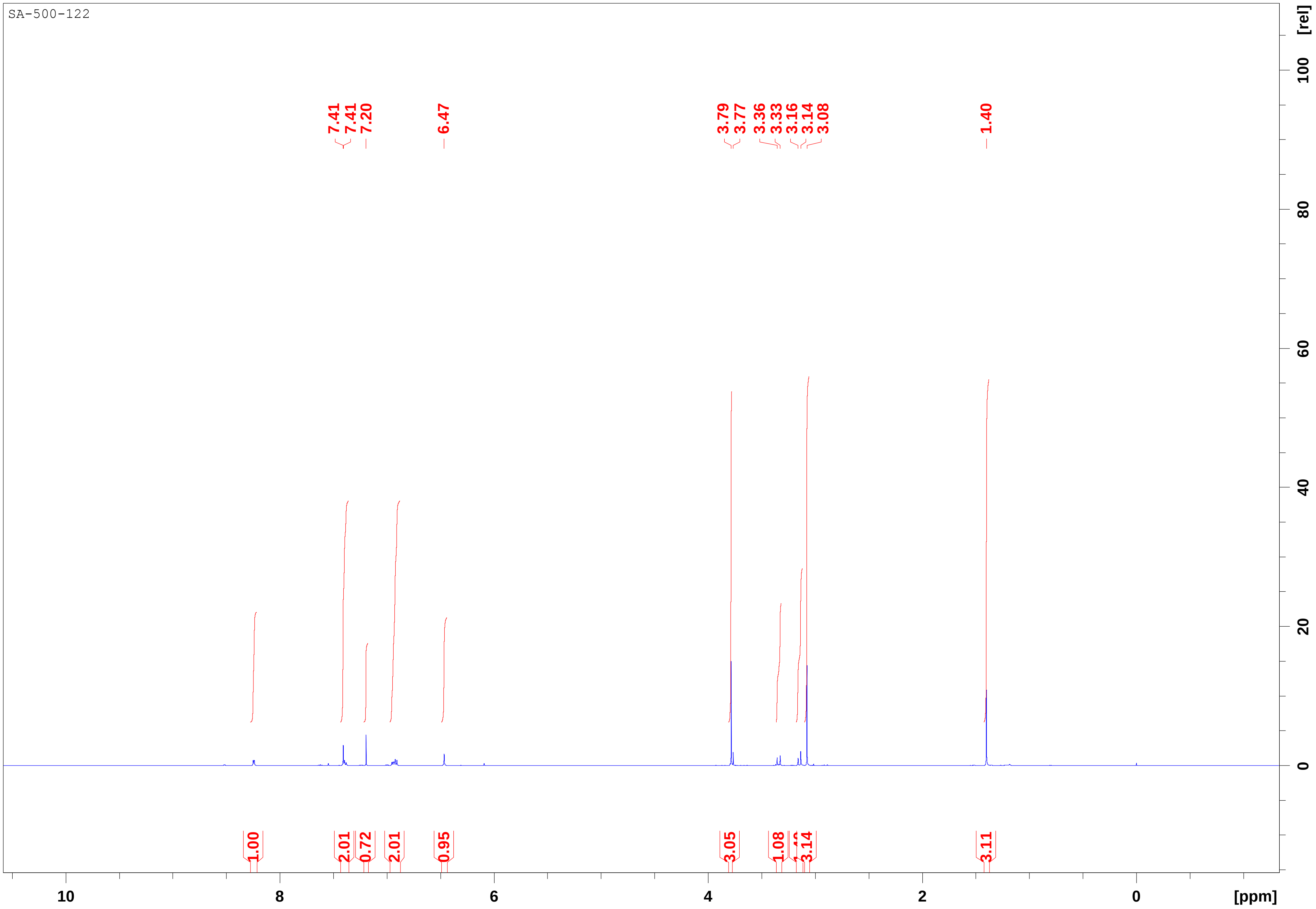

21b

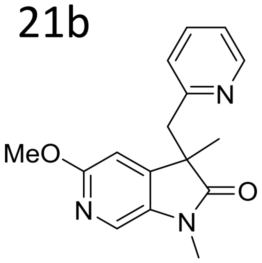
MeO
N
N
O
N

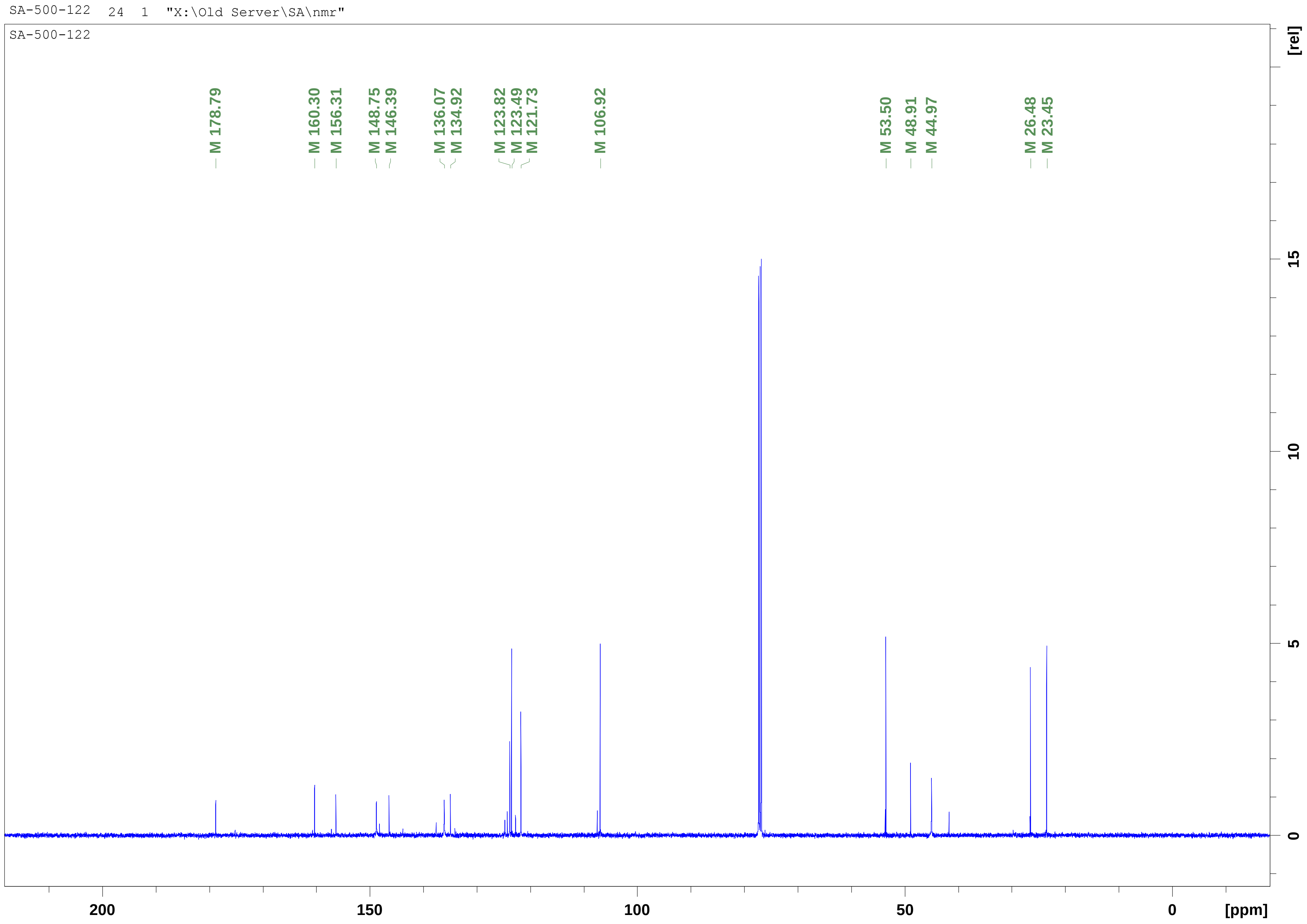
SA-500-122 24 1 "X:\Old Server\SA\nmr"
SA-500-122
M 178.79
M 160.30
M 156.31
M 148.75
M 146.39
M 136.07
M 134.92
M 123.82
M 123.49
M 121.73
M 106.92
M 53.50
M 48.91
M 44.97
M 26.48
M 23.45
[rel]
15
10
5
0
200
150
100
50
0
[ppm]

21c

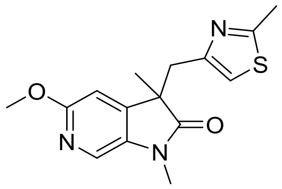

SH-493-233A 10 1 "X:\Old Server\SH\nmr"

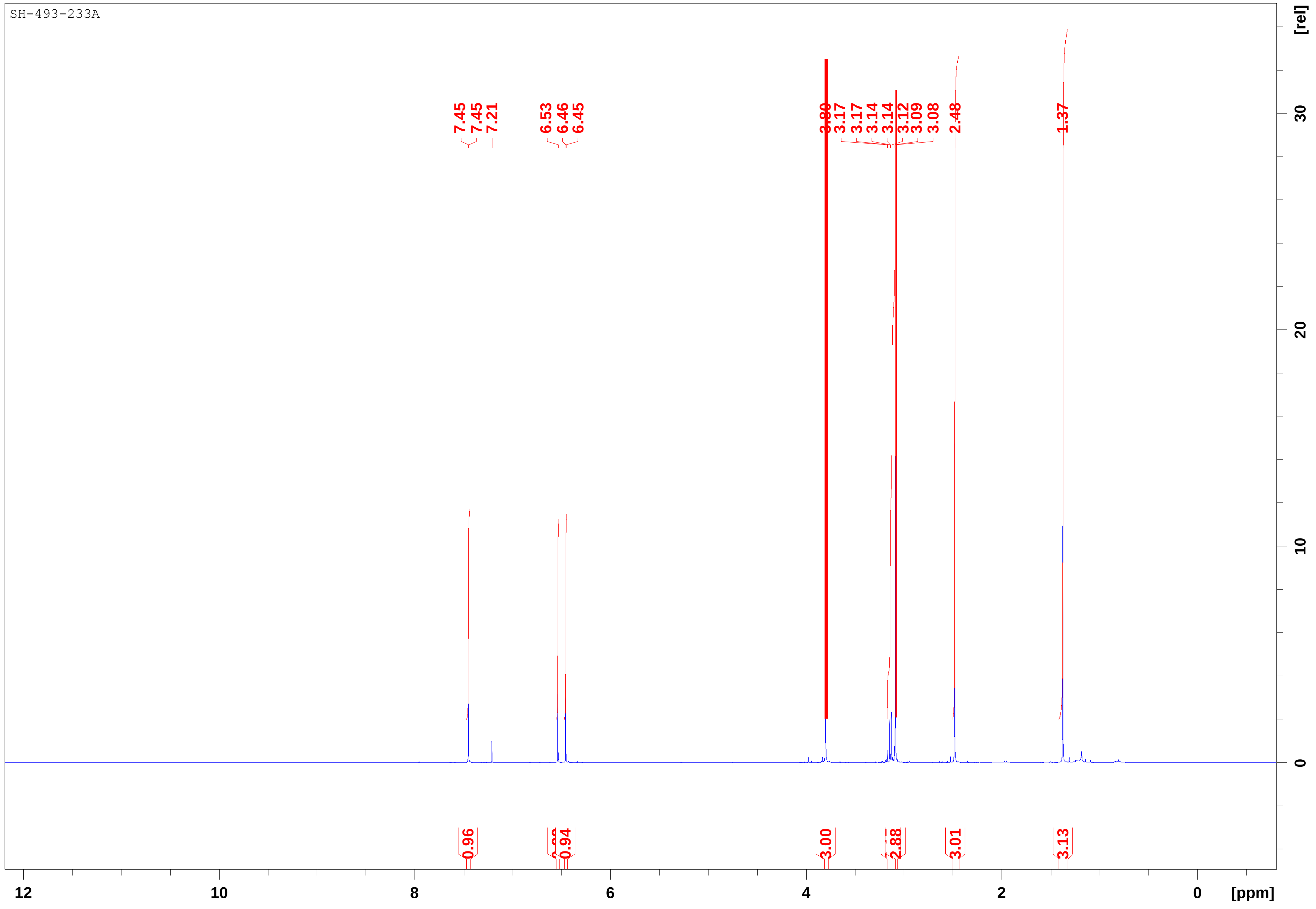

21c

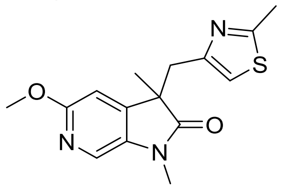

SH-493-233A 11 1 "X:\Old Server\SH\nmr"

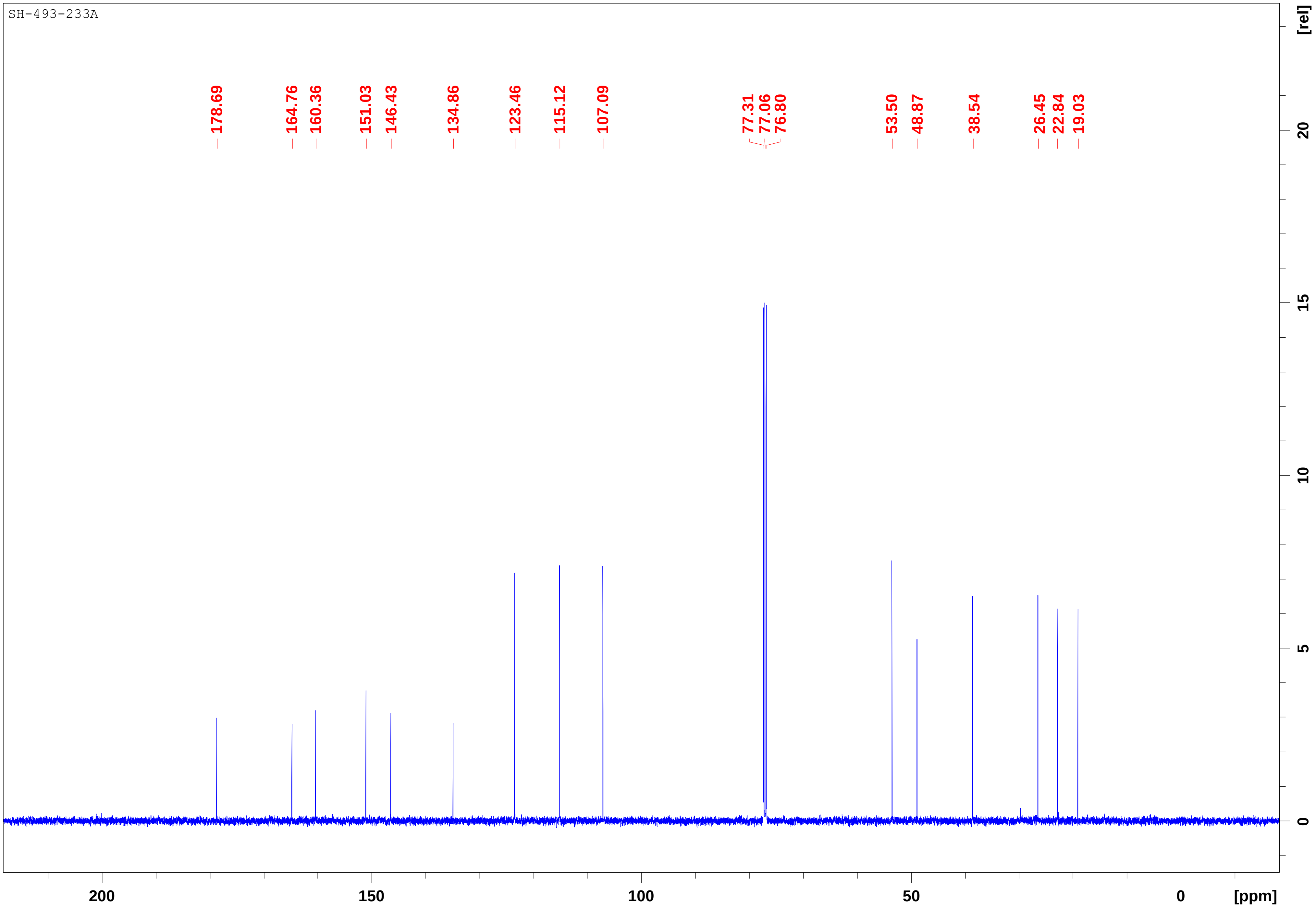

21d

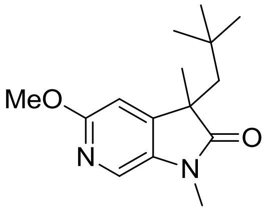

"DCM-494-229-A CDCL3" 10 1 X:\data\DCM\nmr

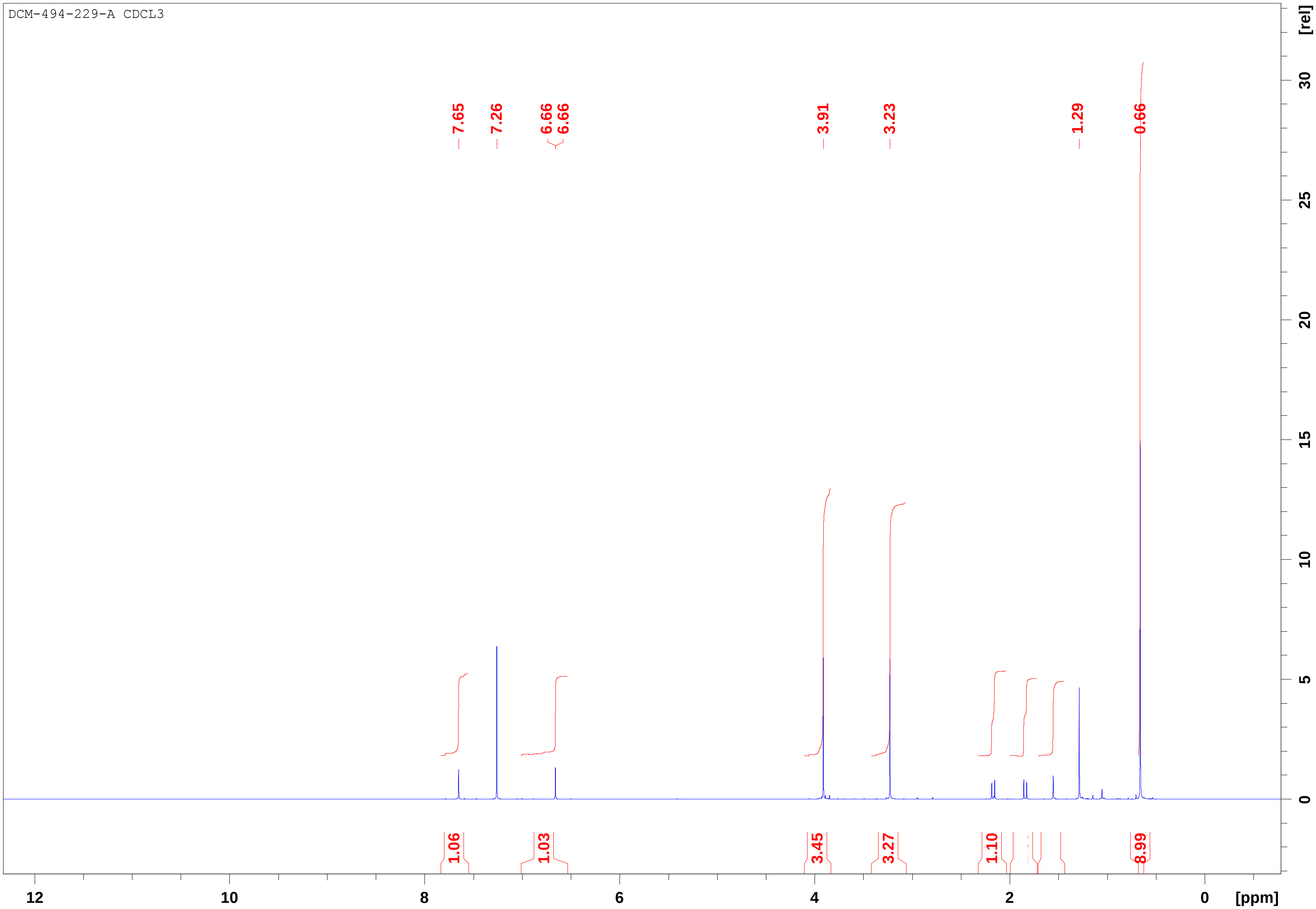

21d

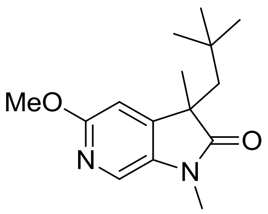

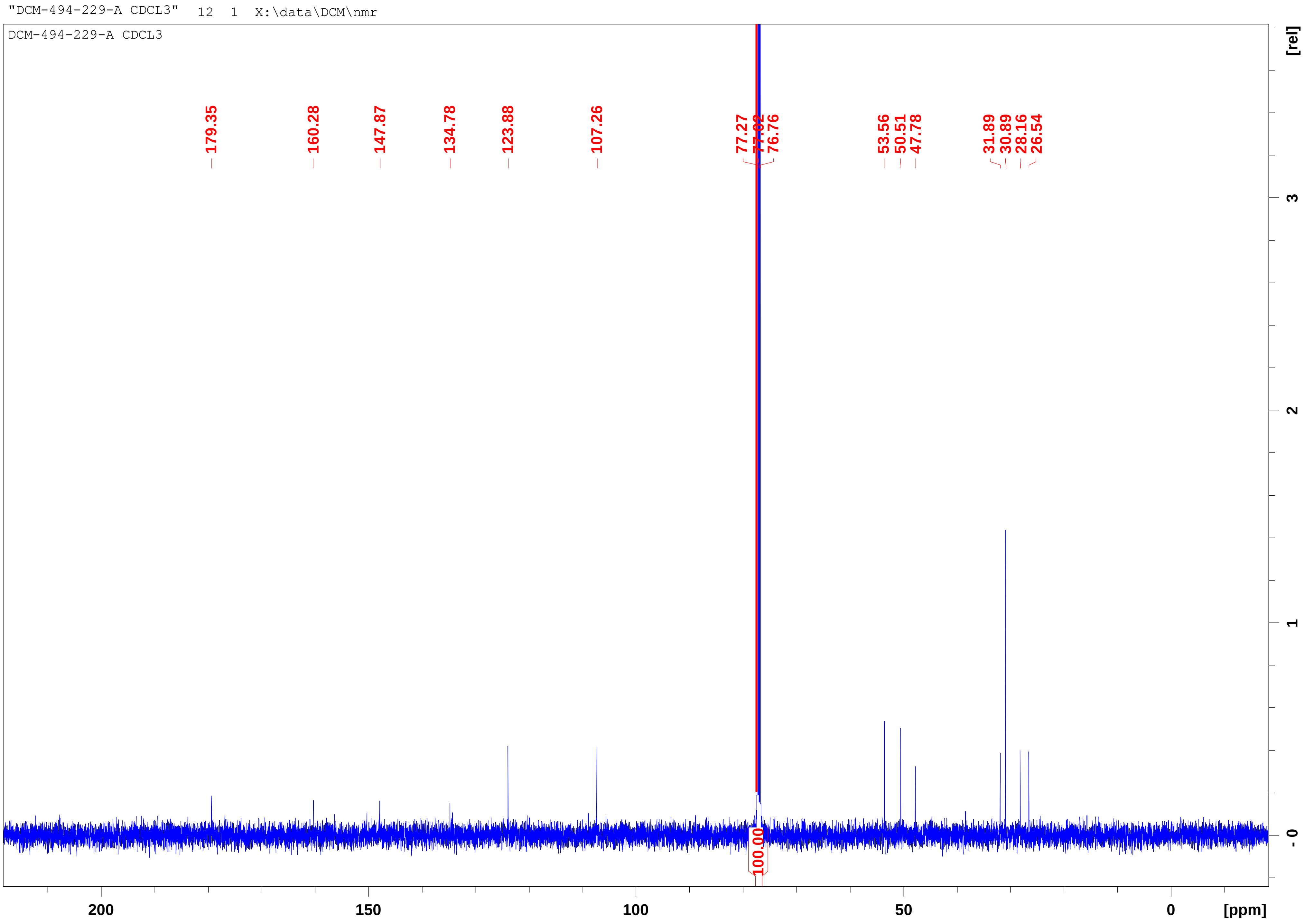

"DCM-494-229-A CDCL3" 12 1 X:\data\DCM\nmr
DCM-494-229-A CDCL3
179.35
160.28
147.87
134.78
123.88
107.26
77.27
77.02
76.76
53.56
50.51
47.78
31.89
30.89
28.16
26.54
100.00
[rel]
3
2
1
0
200
150
100
50
0
[ppm]

21e

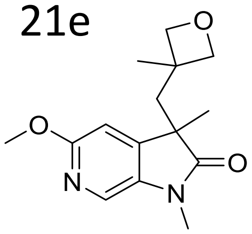

SA-500-156 10 1 "X:\Old Server\SA\nmr"

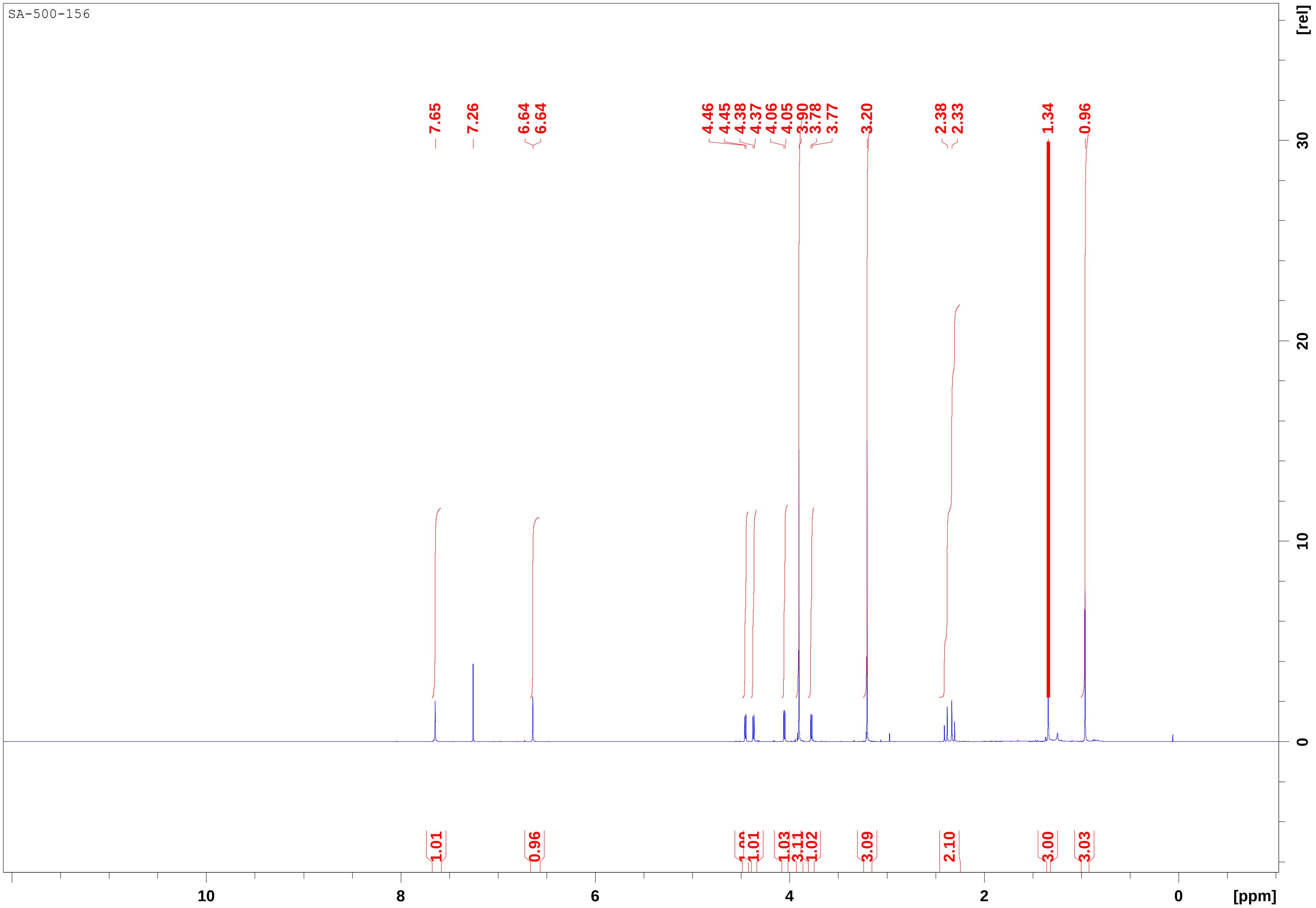

21e

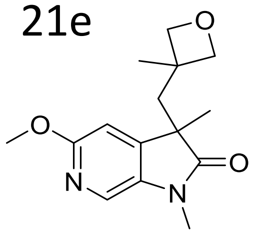

SA-500-156 11 1 "X:\Old Server\SA\nmr"

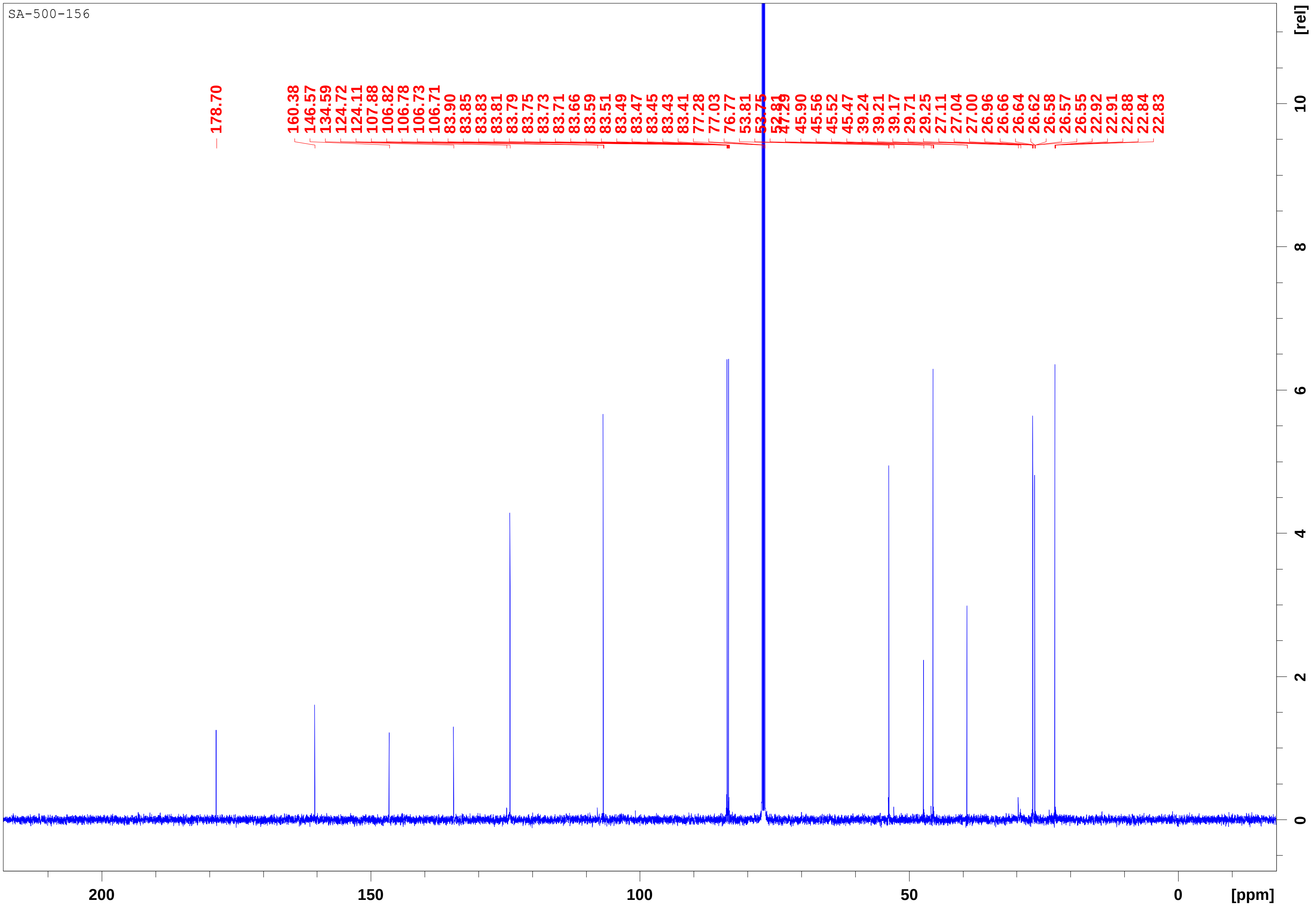

21f

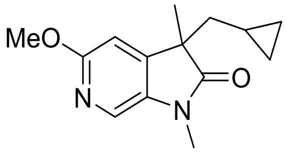
MeO
N
N
O

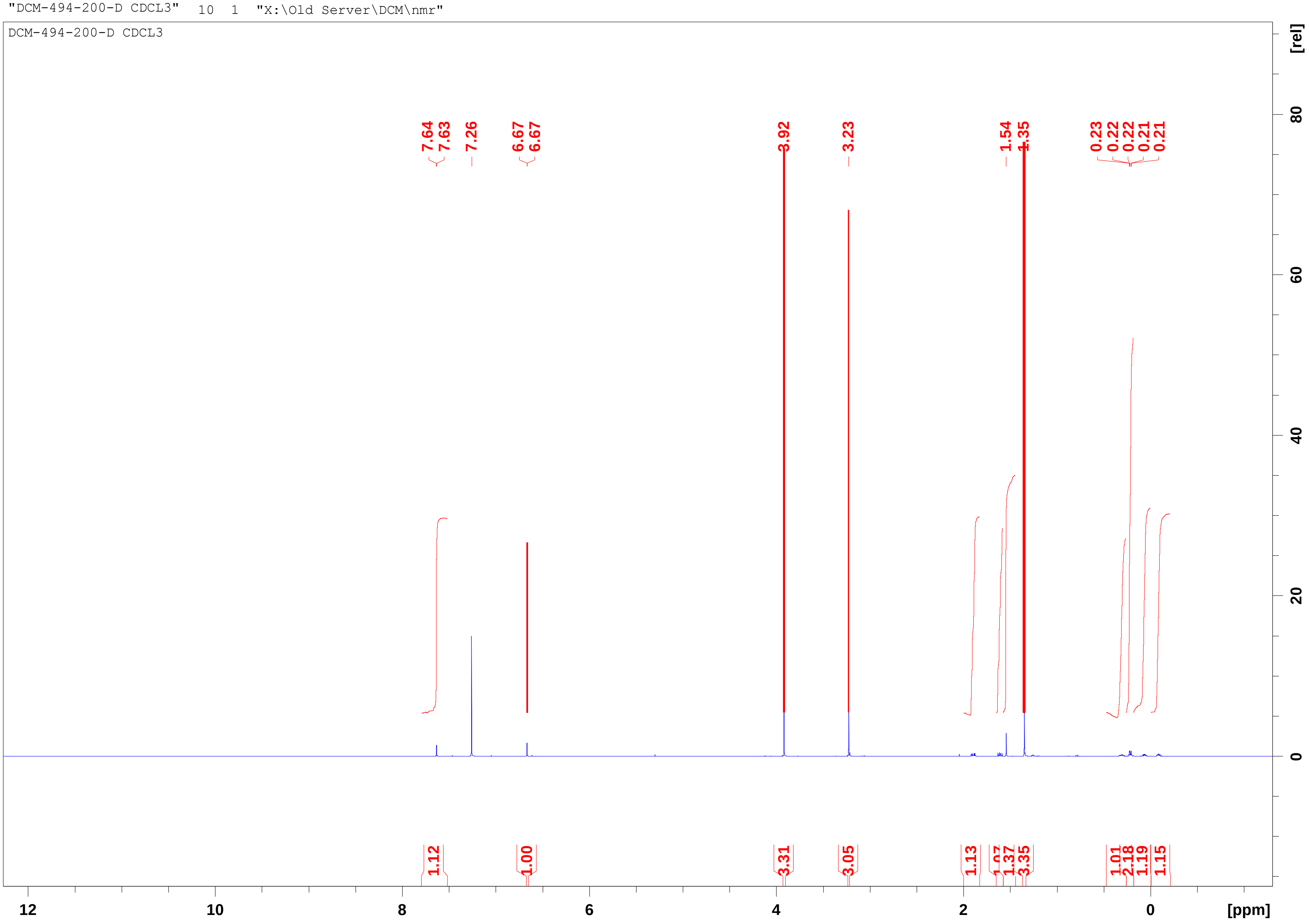
"DCM-494-200-D CDCL3" 10 1 "X:\Old Server\DCM\nmr"
DCM-494-200-D CDCL3
7.64
7.63
7.26
6.67
6.67
3.92
3.23
1.54
1.35
0.23
0.22
0.22
0.21
0.21
1.12
1.00
3.31
3.05
1.13
1.07
1.37
3.35
1.01
2.18
1.19
1.15
[rel]
80
60
40
20
0
12
10
8
6
4
2
0
[ppm]

21f

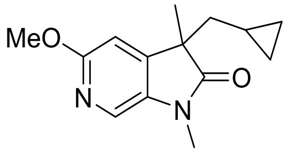

"DCM-494-200-A CDCL3" 20 1 "X:\Old Server\DCM\nmr"

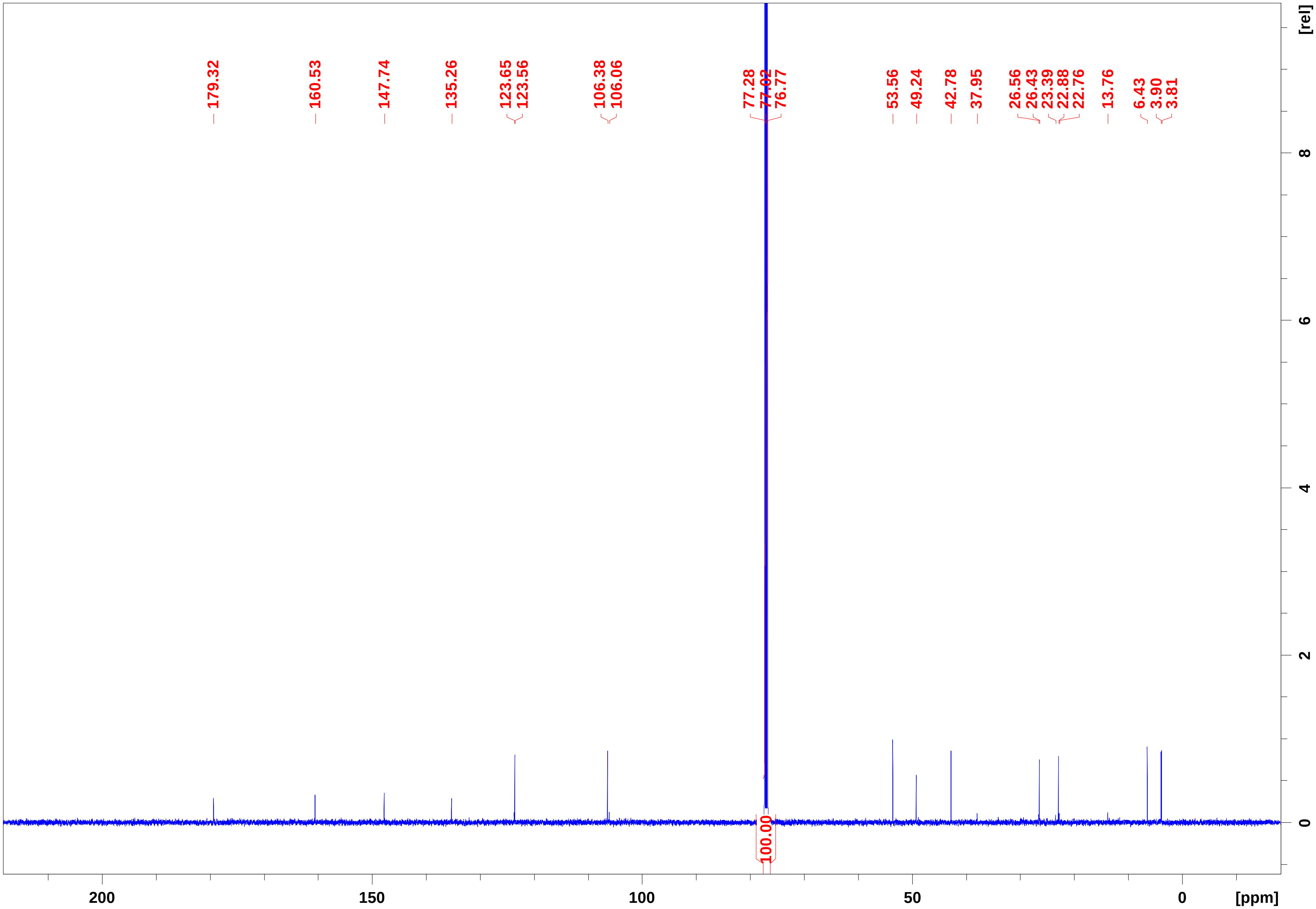

22a

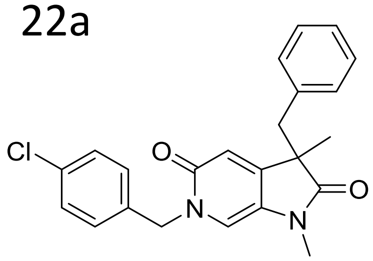

SA-500-136

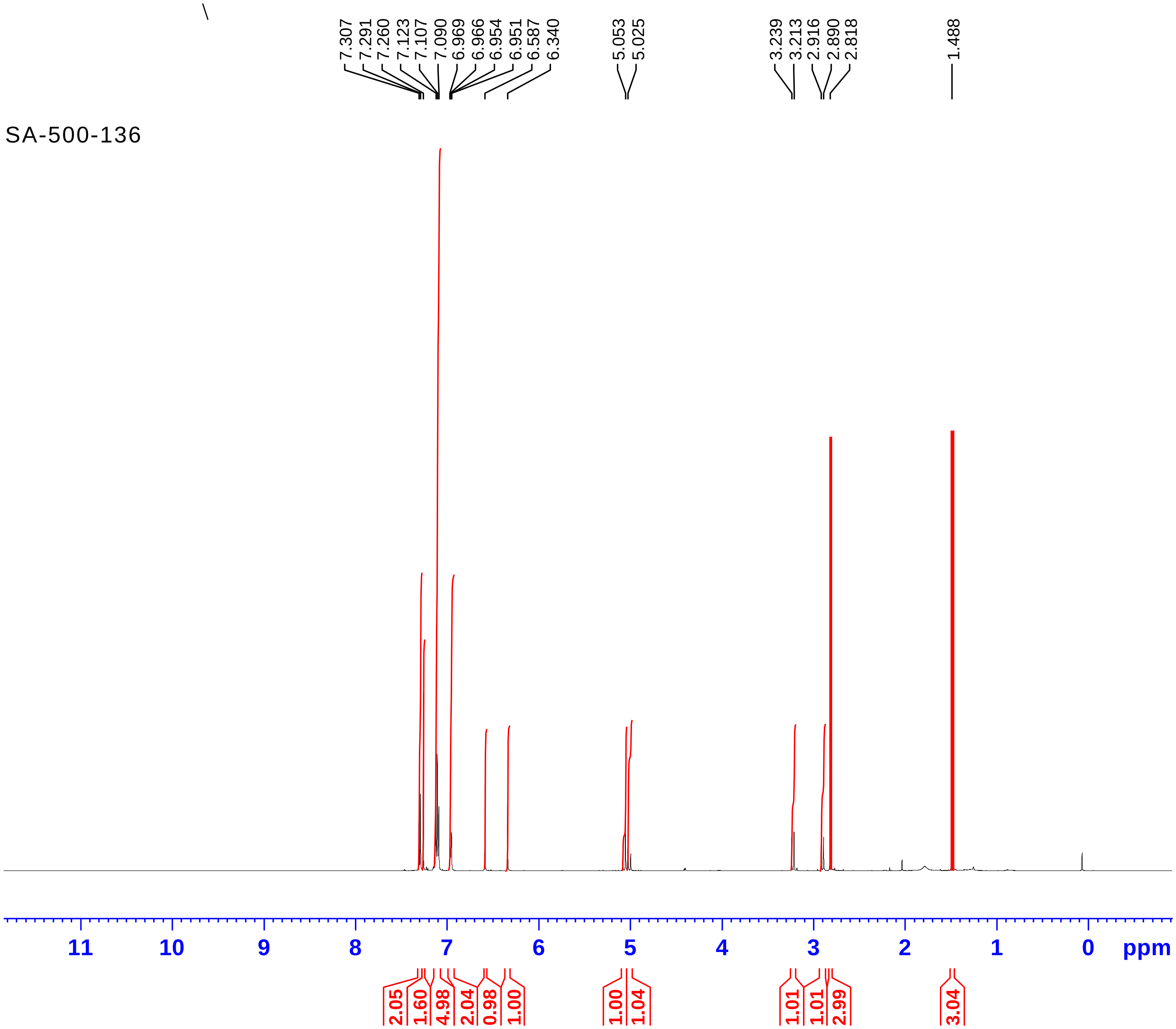

Current Data Parameters
NAME SA-500-136-DRIED
EXPNO 10
PROCNO 1

F2 - Acquisition Parameters
Date_ 20150317
Time 17.13
INSTRUM spect
PROBHD 5 mm PABBO BB-
PULPROG zg30
TD 65536
SOLVENT CDCl3
NS 16
DS 0
SWH 10330.578 Hz
FIDRES 0.157632 Hz
AQ 3.1719425 sec
RG 512
DW 48.400 usec
DE 11.99 usec
TE 296.9 K
D1 1.00000000 sec
TD0 1

======== CHANNEL f1 ========
SFO1 500.3030896 MHz
NUC1 1H
P1 16.25 usec
PLW1 18.33600044 W

F2 - Processing parameters SI 65536
SF 500.3000120 MHz
WDW EM
SSB 0
LB 0.30 Hz
GB 0
PC 1.00

22a

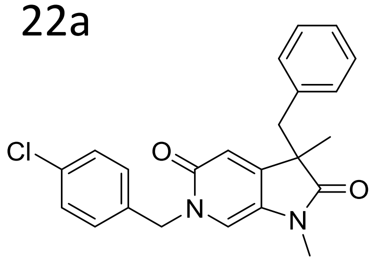

SA-500-136

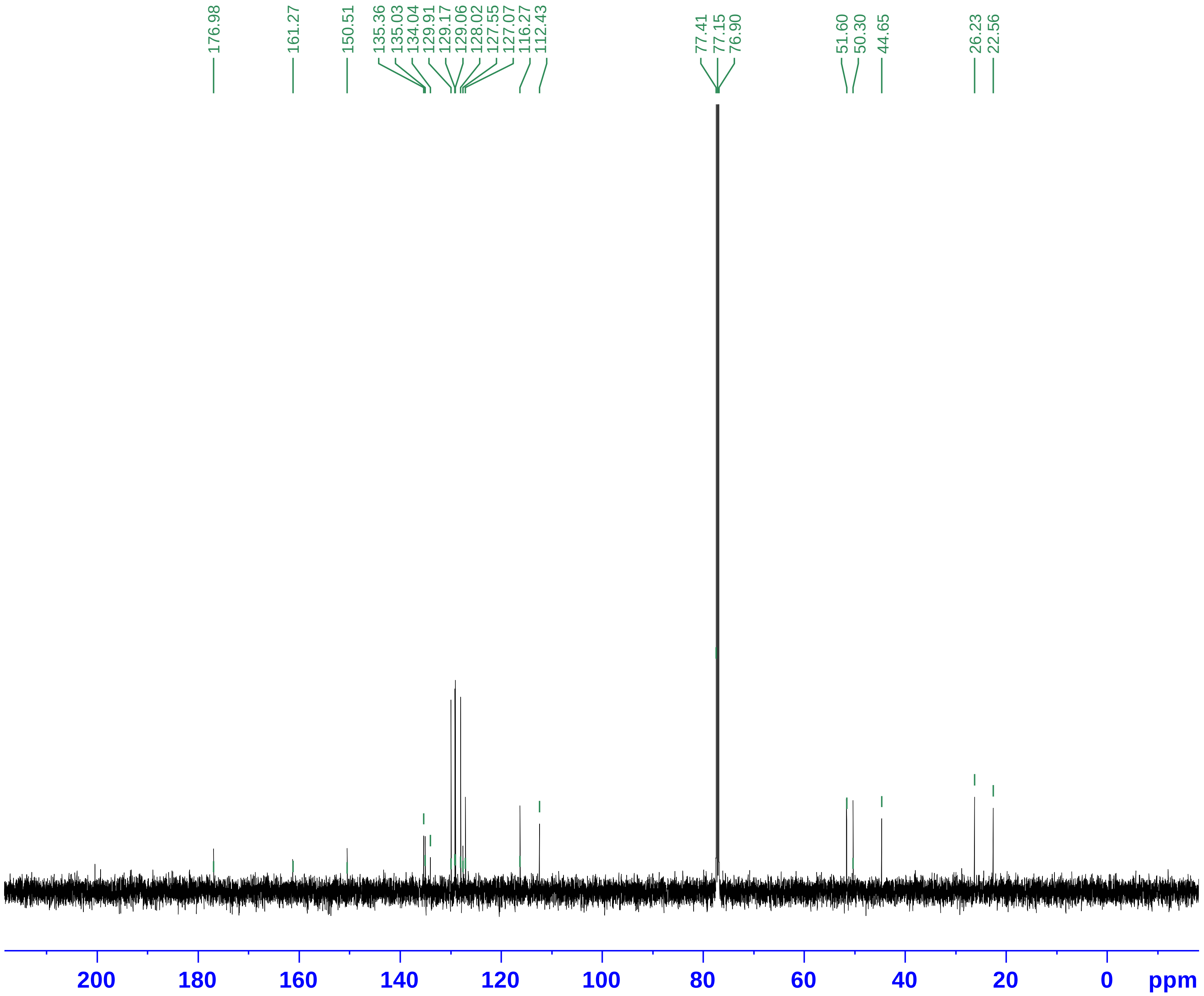

Current Data Parameters
NAME SA-500-136-DRIED
EXPNO 11
PROCNO 1

F2 - Acquisition Parameters
Date_ 20150318
Time 1.50
INSTRUM spect
PROBHD 5 mm PABBO BB-
PULPROG zgpg30
TD 65536
SOLVENT CDCl3
NS 256
DS 2
SWH 29761.904 Hz
FIDRES 0.454131 Hz
AQ 1.1010048 sec
RG 456
DW 16.800 usec
DE 7.68 usec
TE 297.0 K
D1 2.00000000 sec
D11 0.03000000 sec
TD0 1

======== CHANNEL f1 ========
SFO1 125.8131151 MHz
NUC1 13C
P1 9.75 usec
PLW1 82.38999939 W

======== CHANNEL f2 ========
SFO2 500.3020012 MHz
NUC2 1H
CPDPRG[2 waltz16
PCPD2 80.00 usec
PLW2 18.33600044 W
PLW12 0.75654000 W
PLW13 0.48418999 W

F2 - Processing Parameters
SI 65536
SF 125.8005190 MHz
WDW EM
SSB 0
LB 1.00 Hz
GB 0
PC 1.20

22b

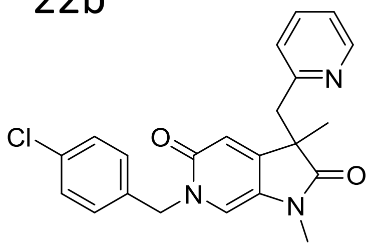

SA-500-128

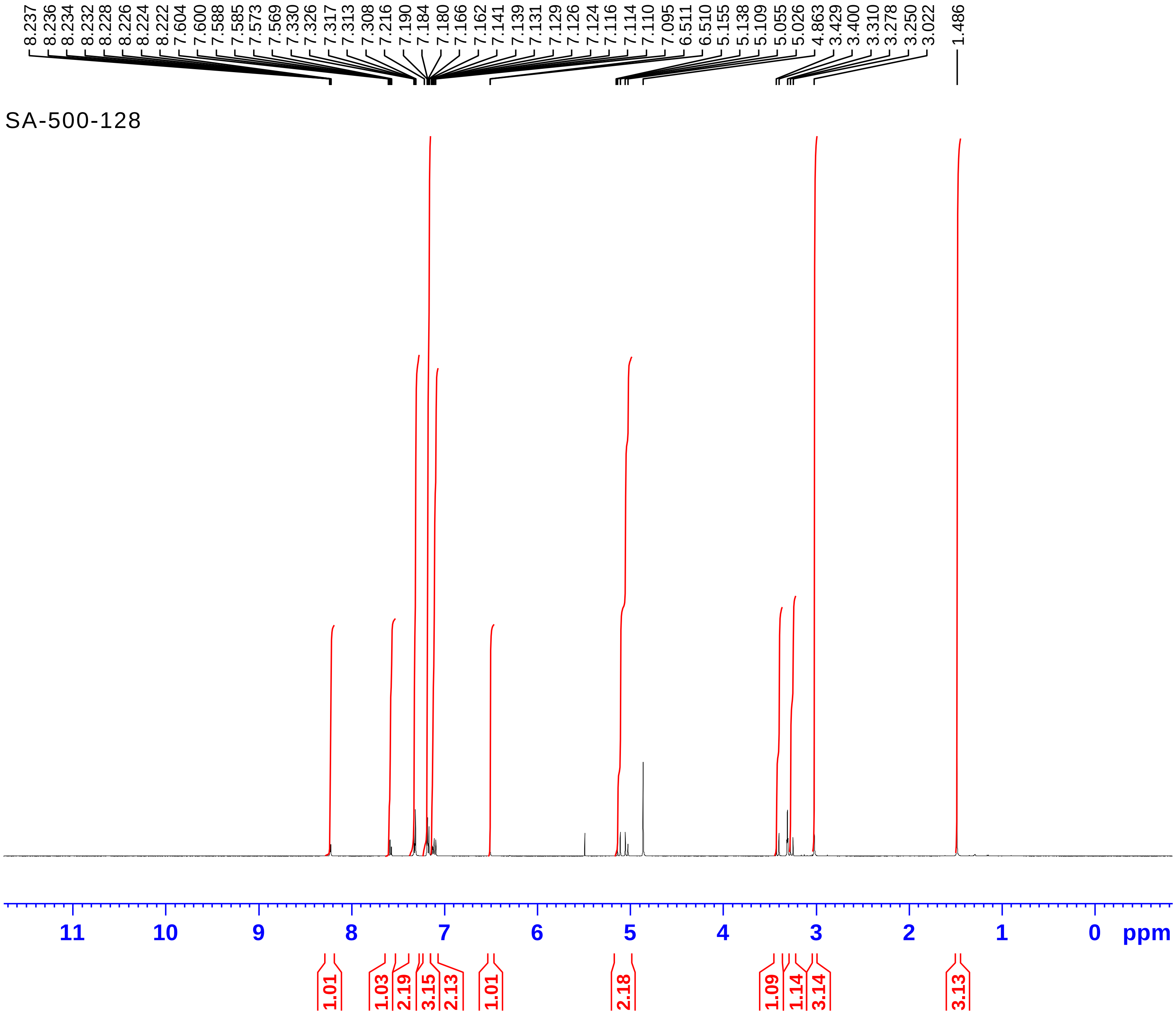

Current Data Parameters

NAME	SA-500-128	
EXPNO	10	
PROCNO	1	

F2 - Acquisition Parameters

Date_	20150312	
Time	19.05	
INSTRUM	spect	
PROBHD	5 mm PABBO BB-	
PULPROG	zg30	
TD	65536	
SOLVENT	MeOD	
NS	16	
DS	0	
SWH	10330.578	Hz
FIDRES	0.157632	Hz
AQ	3.1719425	sec
RG	406	
DW	48.400	usec
DE	11.99	usec
TE	297.0	K
D1	1.00000000	sec
TD0	1	

======== CHANNEL f1 ========

SFO1	500.3030896	MHz
NUC1	1H	
P1	16.25	usec
PLW1	18.33600044	W

F2 - Processing parameters

SI	65536	
SF	500.3000097	MHz
WDW	EM	
SSB	0	
LB	0.30	Hz
GB	0	
PC	1.00	

22b

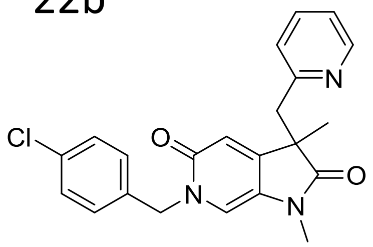
Cl
O
N
N
O
N

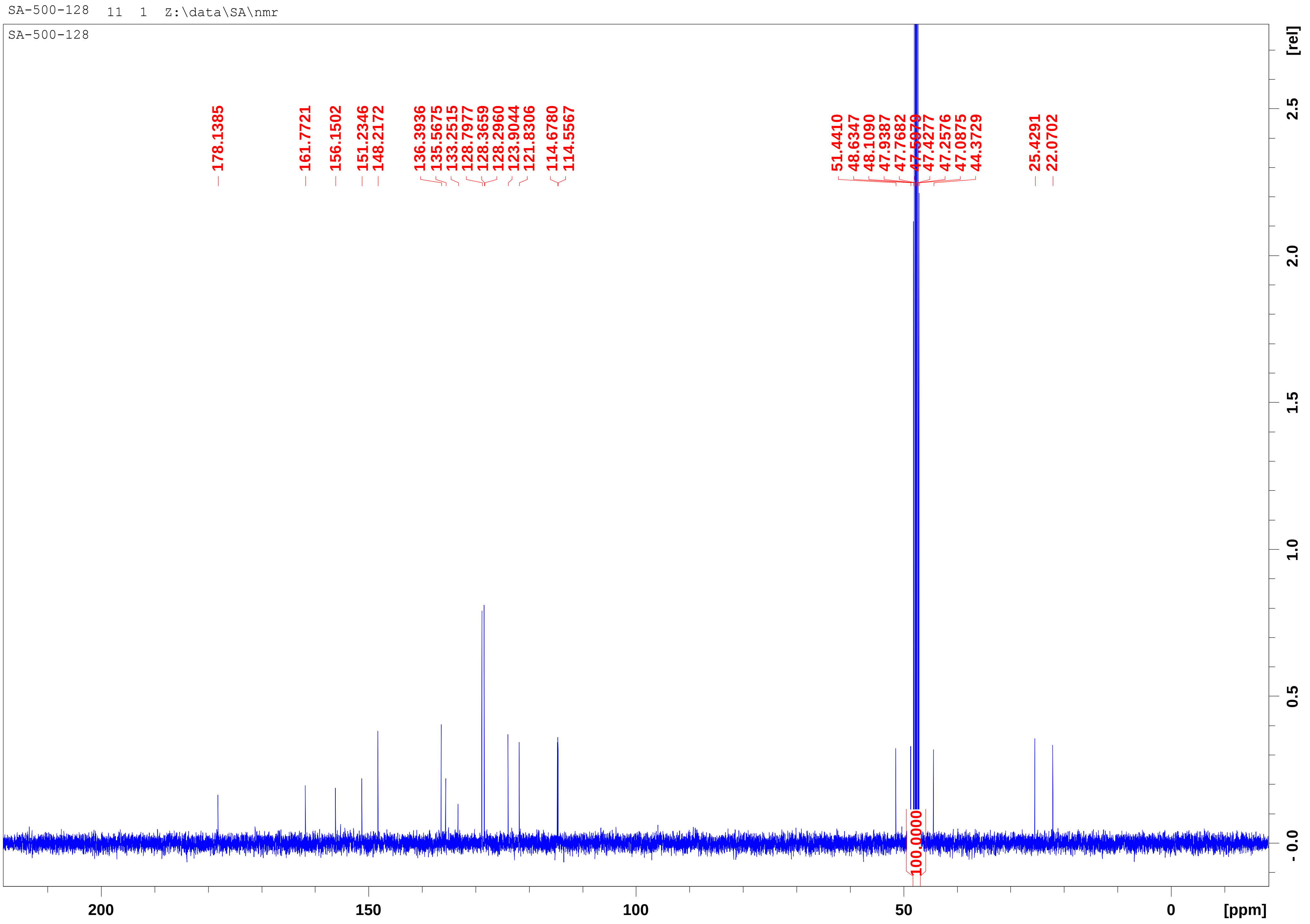
SA-500-128 11 1 Z:\data\SA\nmr
SA-500-128
178.1385
161.7721
156.1502
151.2346
148.2172
136.3936
135.5675
133.2515
128.7977
128.3659
128.2960
123.9044
121.8306
114.6780
114.5567
51.4410
48.6347
48.1090
47.9387
47.7682
47.5979
47.4277
47.2576
47.0875
44.3729
25.4291
22.0702
100.0000
[rel]
2.5
2.0
1.5
1.0
0.5
- 0.0
200
150
100
50
0
[ppm]

22c

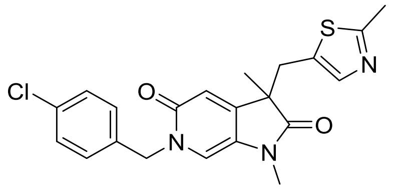

SH-493-239A - diluted

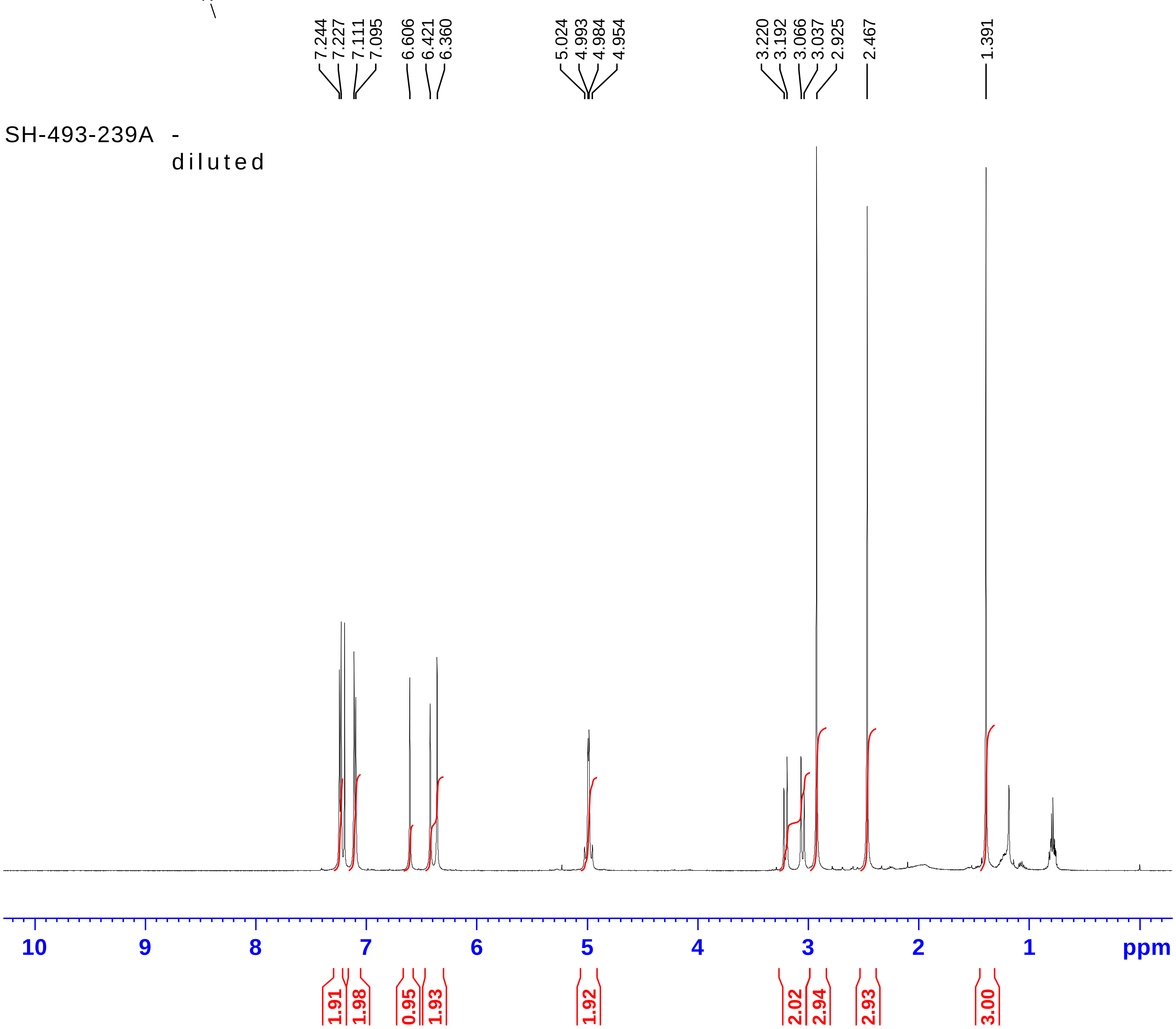

Current Data Parameters
NAME SH-493-239A
EXPNO 16
PROCNO 1

F2 - Acquisition Parameters
Date 20150410
Time 10.16
INSTRUM spect
PROBHD 5 mm PABBO BB-
PULPROG zg30
TD 65536
SOLVENT CDCl3
NS 16
DS 0
SWH 10330.578 Hz
FIDRES 0.157632 Hz
AQ 3.1719425 sec
RG 322
DW 48.400 usec
DE 11.99 usec
TE 297.0 K
D1 1.00000000 sec
TD0 1

======== CHANNEL f1 ========
SFO1 500.3030896 MHz
NUC1 1H
P1 16.25 usec
PLW1 18.33600044 W

F2 - Processing parameters SI 65536
SF 500.3000438 MHz
WDW EM
SSB 0
LB 0.30 Hz
GB 0
PC 1.00

22c

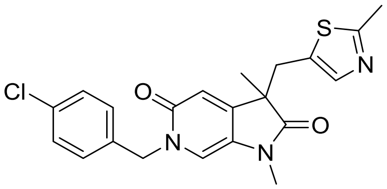

SH-493-239A

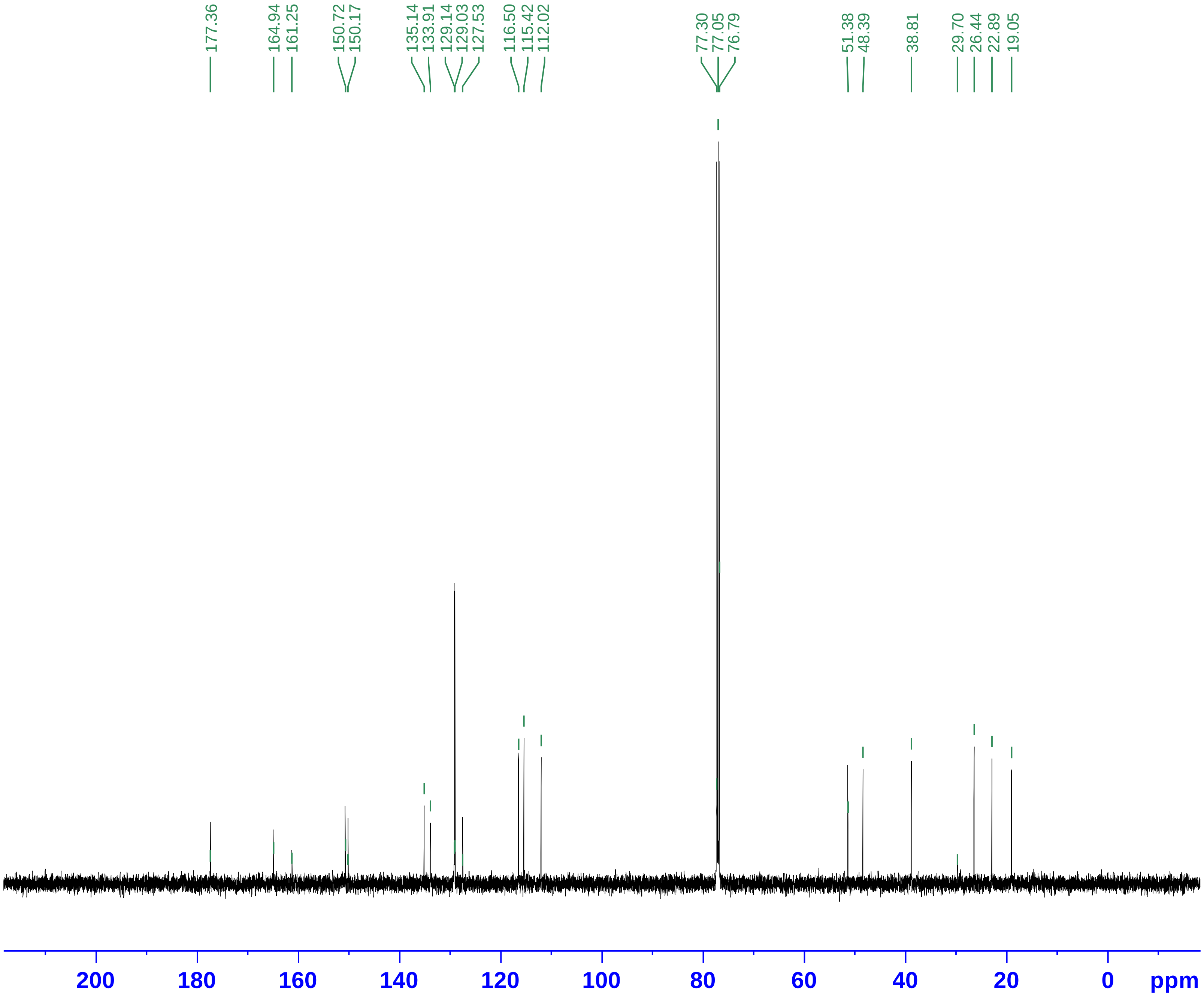

Current Data Parameters
NAME SH-493-239A
EXPNO 11
PROCNO 1

F2 - Acquisition Parameters
Date_ 20150410
Time 8.52
INSTRUM spect
PROBHD 5 mm PABBO BB-
PULPROG zgpg30
TD 65536
SOLVENT CDCl3
NS 256
DS 2
SWH 29761.904 Hz
FIDRES 0.454131 Hz
AQ 1.1010048 sec
RG 575
DW 16.800 usec
DE 7.68 usec
TE 298.7 K
D1 2.00000000 sec
D11 0.03000000 sec
TD0 1

======== CHANNEL f1 ========

SFO1 125.8131151 MHz
NUC1 13C
P1 9.75 usec
PLW1 82.38999939 W

======== CHANNEL f2 ========

SFO2 500.3020012 MHz
NUC2 1H
CPDPRG[2 waltz16
PCPD2 80.00 usec
PLW2 18.33600044 W
PLW12 0.75654000 W
PLW13 0.48418999 W

F2 - Processing parameters
SI 65536
SF 125.8005350 MHz
WDW EM
SSB 0
LB 1.00 Hz
GB 0
PC 1.40

22d

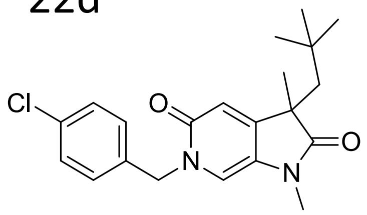

DCM-494-231-A CDCL3

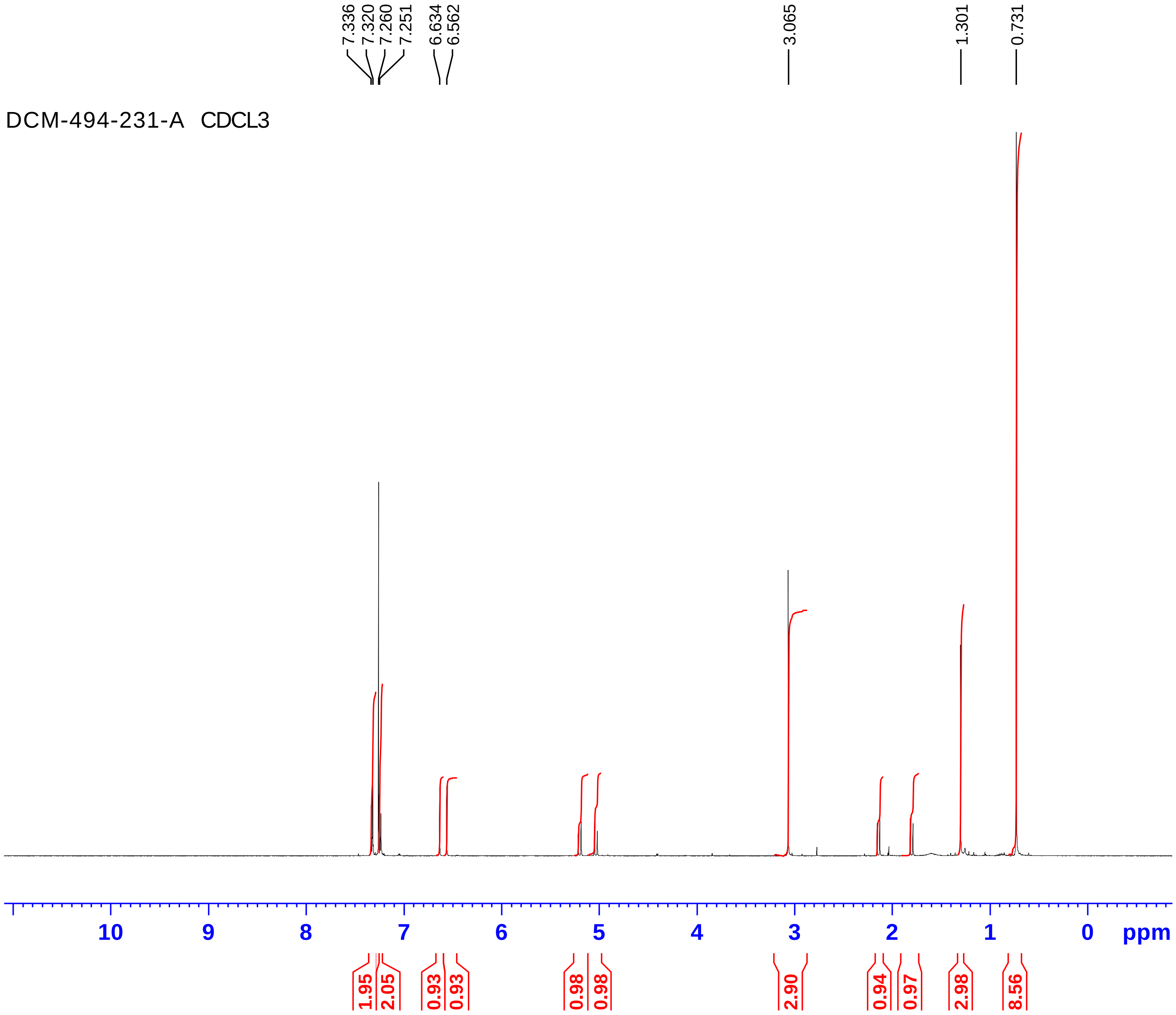

Current Data Parameters
NAME DCM-494-231-A CDCL3
EXPNO 10
PROCNO 1

F2 - Acquisition Parameters
Date 20150506
Time 14.58
INSTRUM spect
PROBHD 5 mm PABBO BB-
PULPROG zg30
TD 65536
SOLVENT CDCl3
NS 16
DS 0
SWH 10330.578 Hz
FIDRES 0.157632 Hz
AQ 3.1719425 sec
RG 645
DW 48.400 usec
DE 11.99 usec
TE 297.0 K
D1 1.00000000 sec
TD0 1

======== CHANNEL f1 ========
SFO1 500.3030896 MHz
NUC1 1H
P1 16.25 usec
PLW1 18.33600044 W

F2 - Processing parameters SI 65536
SF 500.3000118 MHz
WDW EM
SSB 0
LB 0.30 Hz
GB 0
PC 1.00

22d

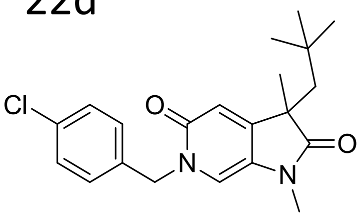

DCM-494-231-A CDCL3

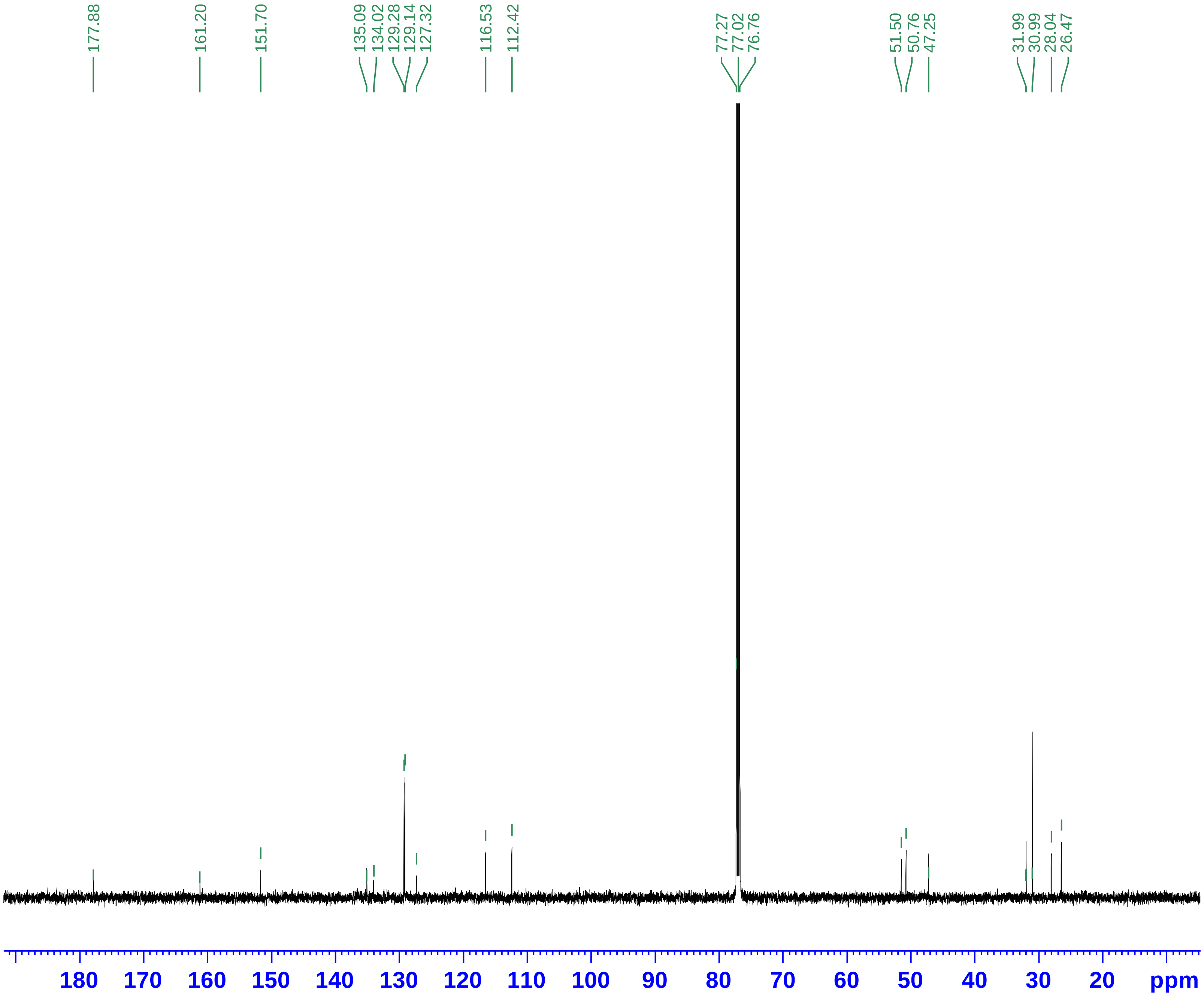

Current Data Parameters
NAME DCM-494-231-A CDCL3
EXPNO 12
PROCNO 1

F2 - Acquisition Parameters
Date_ 20150506
Time 20.01
INSTRUM spect
PROBHD 5 mm PABBO BB-
PULPROG zgpg30
TD 65536
SOLVENT CDCl3
NS 2048
DS 2
SWH 29761.904 Hz
FIDRES 0.454131 Hz
AQ 1.1010048 sec
RG 456
DW 16.800 usec
DE 7.68 usec
TE 297.6 K
D1 2.00000000 sec
D11 0.03000000 sec
TD0 1

======== CHANNEL f1 ========

SFO1 125.8131151 MHz
NUC1 13C
P1 9.75 usec
PLW1 82.38999939 W

======== CHANNEL f2 ========

SFO2 500.3020012 MHz
NUC2 1H
CPDPRG[2 waltz16
PCPD2 80.00 usec
PLW2 18.33600044 W
PLW12 0.75654000 W
PLW13 0.48418999 W

F2 - Processing parameters
SI 65536
SF 125.8005350 MHz
WDW EM
SSB 0
LB 1.00 Hz
GB 0
PC 1.40

22e

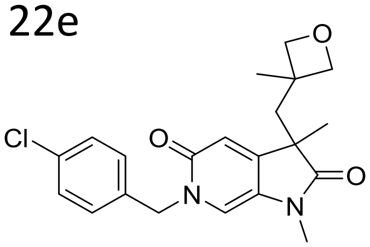

SA-500-160

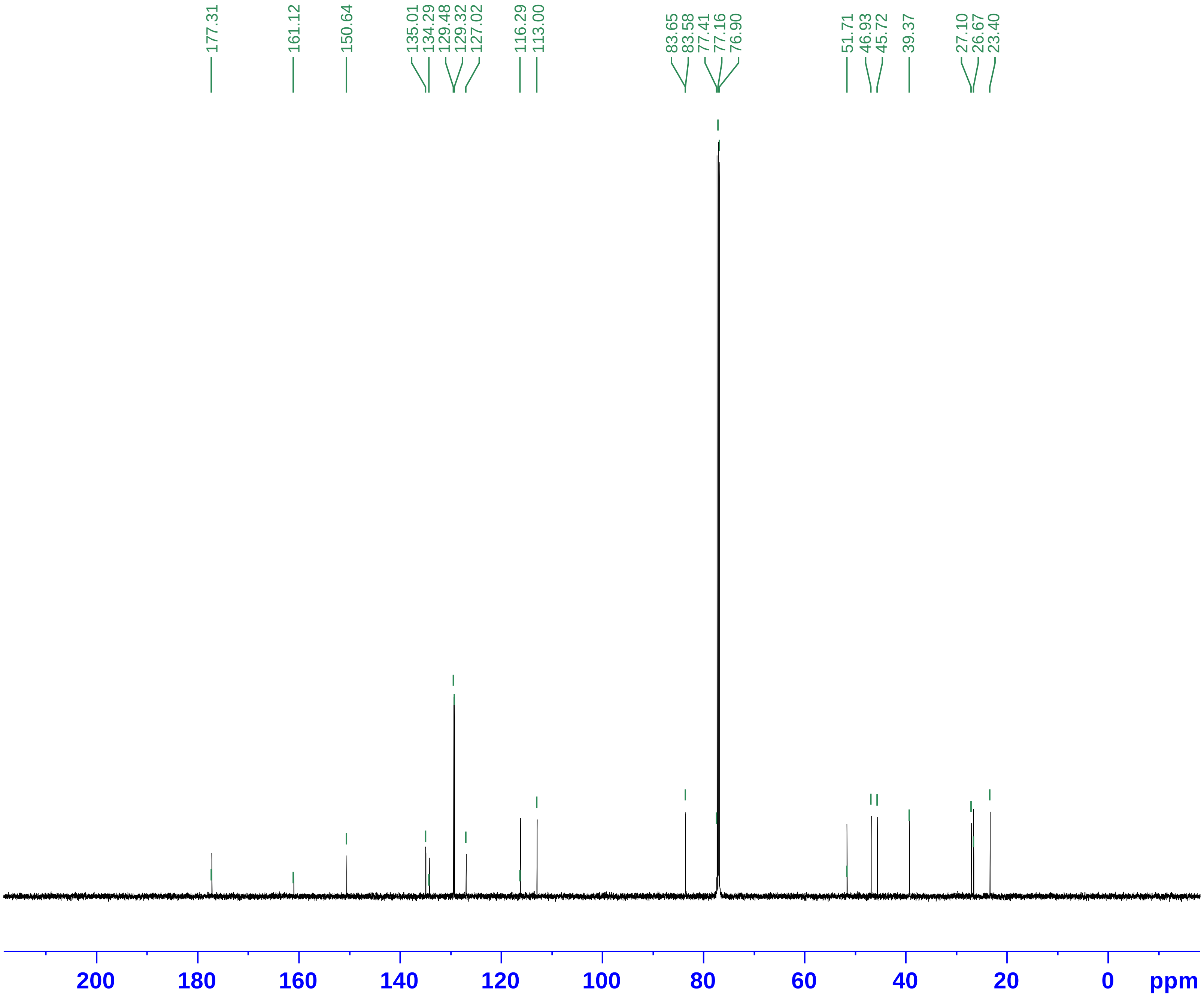

Current Data Parameters
NAME SA-500-160
EXPNO 11
PROCNO 1

F2 - Acquisition Parameters
Date_ 20150402
Time 12.53
INSTRUM spect
PROBHD 5 mm PABBO BB-
PULPROG zgpg30
TD 65536
SOLVENT CDCl3
NS 1024
DS 2
SWH 29761.904 Hz
FIDRES 0.454131 Hz
AQ 1.1010048 sec
RG 575
DW 16.800 usec
DE 7.68 usec
TE 297.9 K
D1 2.00000000 sec
D11 0.03000000 sec
TD0 1

======== CHANNEL f1 ========

SFO1 125.8131151 MHz
NUC1 13C
P1 9.75 usec
PLW1 82.38999939 W

======== CHANNEL f2 ========
SFO2 500.3020012 MHz
NUC2 1H
CPDPRG[2 waltz16
PCPD2 80.00 usec
PLW2 18.33600044 W
PLW12 0.75654000 W
PLW13 0.48418999 W

F2 - Processing parameters
SI 65536
SF 125.8005190 MHz
WDW EM
SSB 0
LB 1.00 Hz
GB 0
PC 1.40

22e

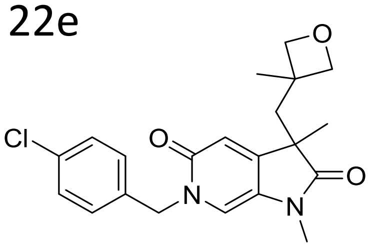

SA-500-160

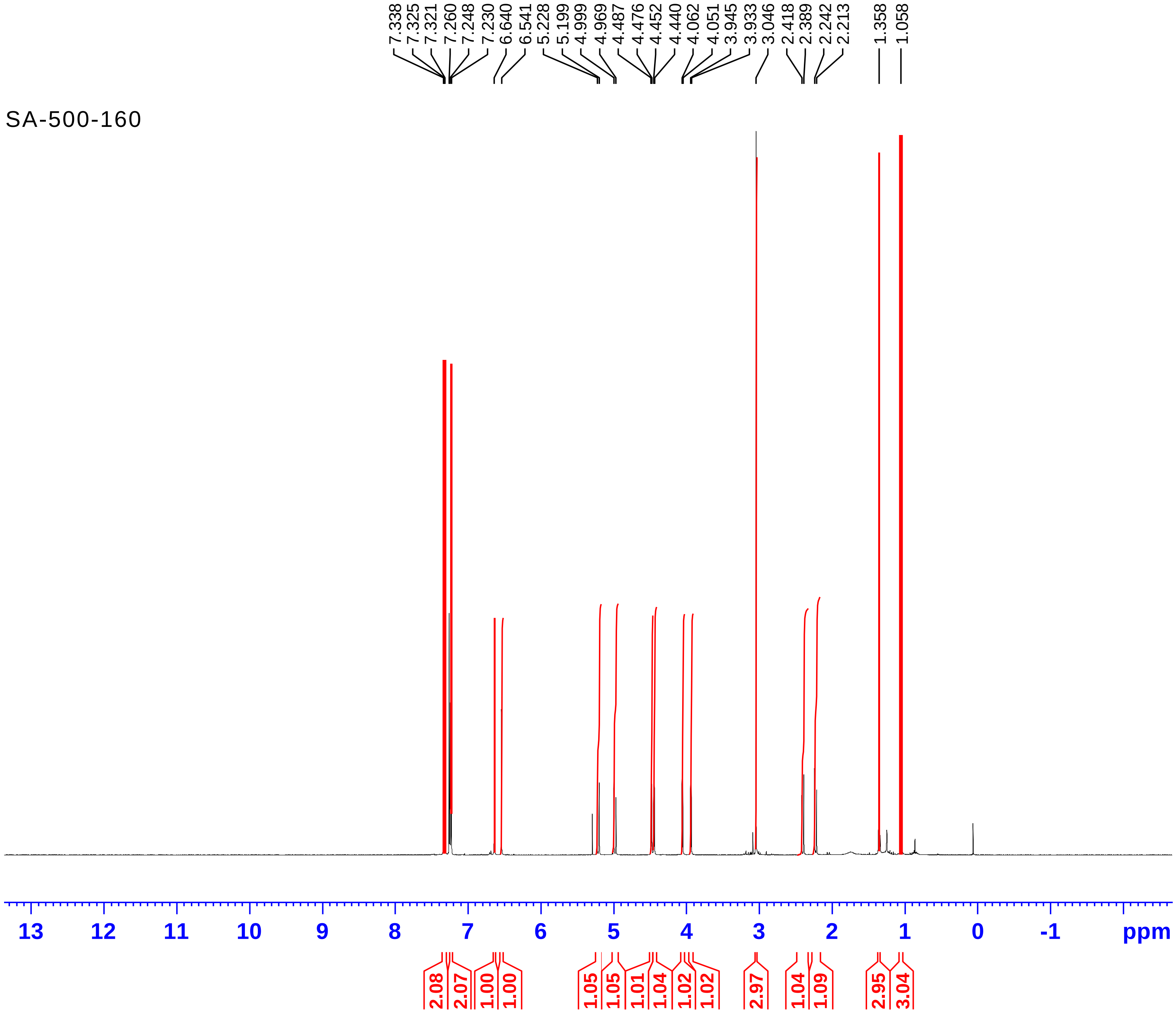

Current Data Parameters
NAME SA-500-160
EXPNO 10
PROCNO 1

F2 - Acquisition Parameters
Date 20150402
Time 11.12
INSTRUM spect
PROBHD 5 mm PABBO BB-
PULPROG zg30
TD 65536
SOLVENT CDCl3
NS 16
DS 0
SWH 10330.578 Hz
FIDRES 0.157632 Hz
AQ 3.1719425 sec
RG 322
DW 48.400 usec
DE 11.99 usec
TE 297.1 K
D1 1.00000000 sec
TD0 1

======== CHANNEL f1 ========
SFO1 500.3030896 MHz
NUC1 1H
P1 16.25 usec
PLW1 18.33600044 W

F2 - Processing parameters
SI 65536
SF 500.3000122 MHz
WDW EM
SSB 0
LB 0.30 Hz
GB 0
PC 1.00

22f

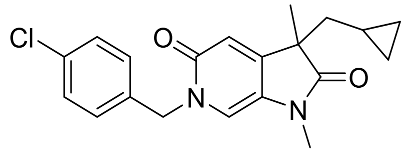

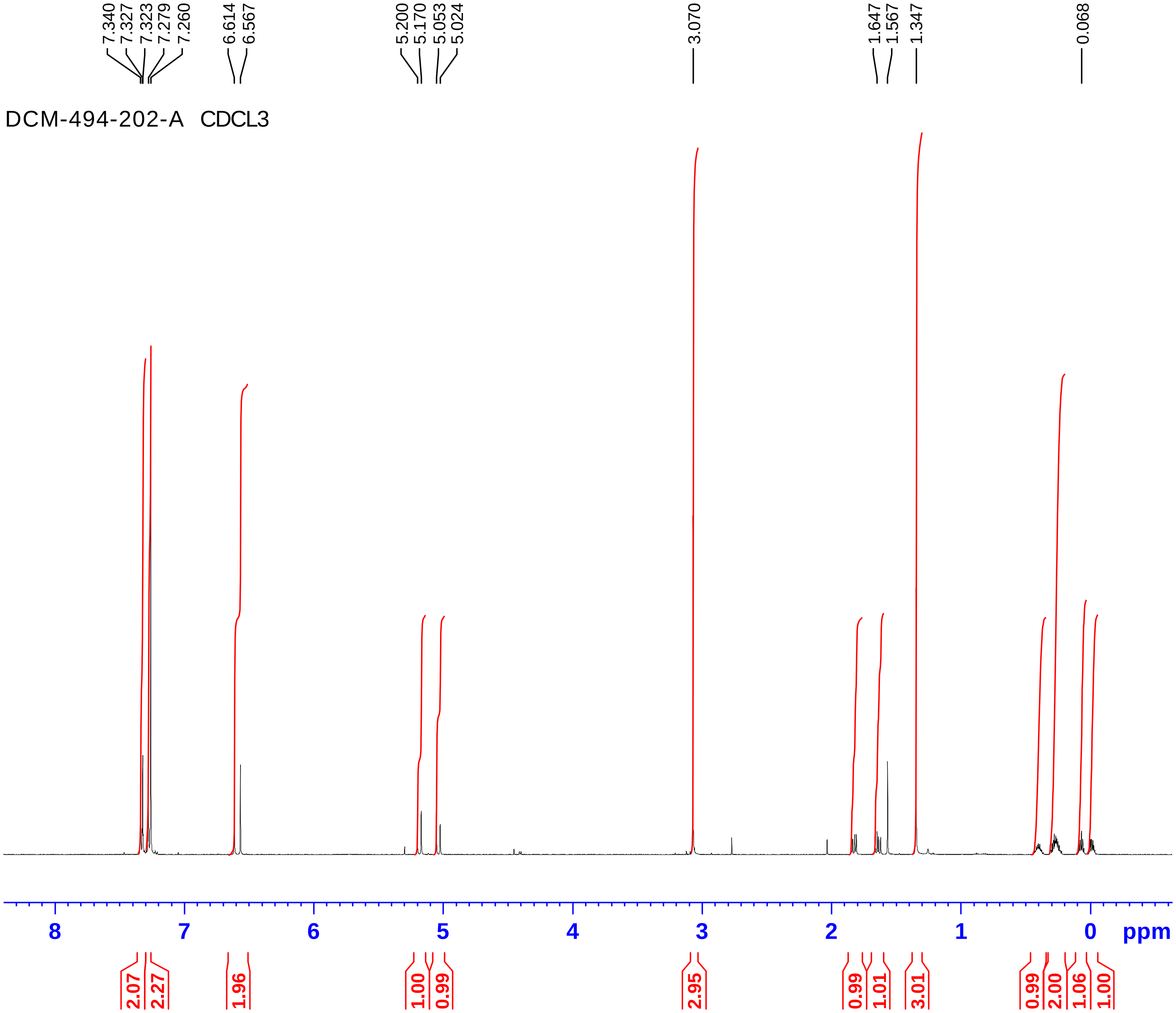

Current Data Parameters
NAME DCM-494-202-A CDCL3
EXPNO 10
PROCNO 1

F2 - Acquisition Parameters
Date 20150317
Time 17.46
INSTRUM spect
PROBHD 5 mm PABBO BB-
PULPROG zg30
TD 65536
SOLVENT CDCl3
NS 16
DS 0
SWH 10330.578 Hz
FIDRES 0.157632 Hz
AQ 3.1719425 sec
RG 645
DW 48.400 usec
DE 11.99 usec
TE 297.0 K
D1 1.00000000 sec
TD0 1

======== CHANNEL f1 ========
SFO1 500.3030896 MHz
NUC1 1H
P1 16.25 usec
PLW1 18.33600044 W

F2 - Processing parameters
SI 65536
SF 500.3000120 MHz
WDW EM
SSB 0
LB 0.30 Hz
GB 0
PC 1.00

22f

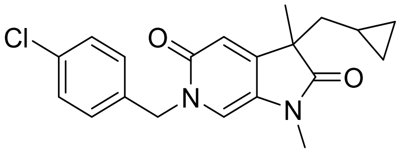

DCM-494-202-A CDCL3

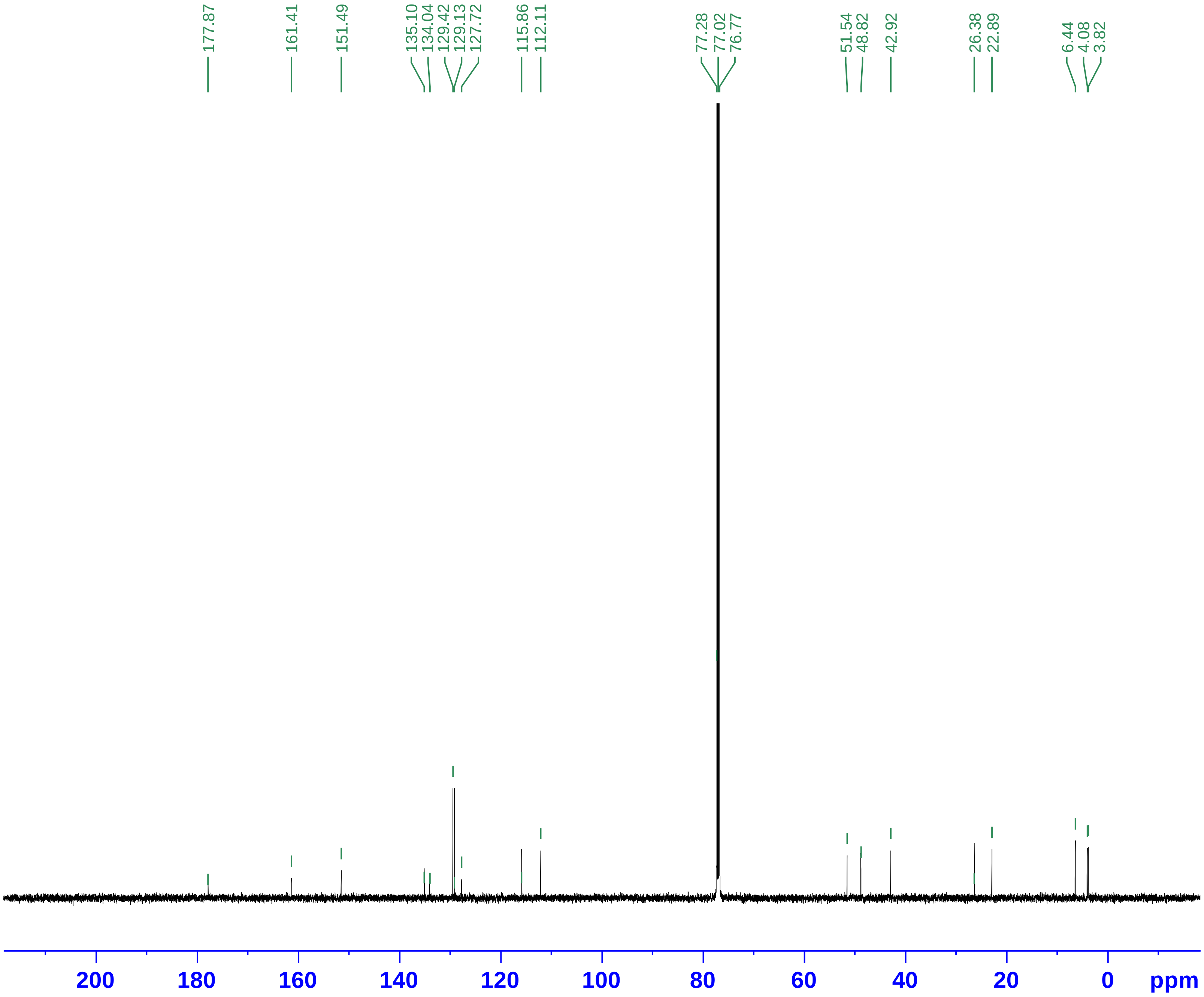

Current Data Parameters
NAME DCM-494-202-A CDCL3
EXPNO 11
PROCNO 1

F2 - Acquisition Parameters
Date_ 20150318
Time 5.44
INSTRUM spect
PROBHD 5 mm PABBO BB-
PULPROG zgpg30
TD 65536
SOLVENT CDCl3
NS 3096
DS 2
SWH 29761.904 Hz
FIDRES 0.454131 Hz
AQ 1.1010048 sec
RG 456
DW 16.800 usec
DE 7.68 usec
TE 297.0 K
D1 2.00000000 sec
D11 0.03000000 sec
TD0 1

======== CHANNEL f1 ========
SFO1 125.8131151 MHz
NUC1 13C
P1 9.75 usec
PLW1 82.38999939 W

======== CHANNEL f2 ========
SFO2 500.3020012 MHz
NUC2 1H
CPDPRG[2 waltz16
PCPD2 80.00 usec
PLW2 18.33600044 W
PLW12 0.75654000 W
PLW13 0.48418999 W

F2 - Processing Parameters
SI 65536
SF 125.8005350 MHz
WDW EM
SSB 0
LB 1.00 Hz
GB 0
PC 1.40

23

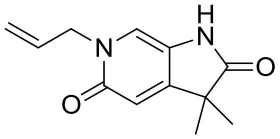

"DCM-494-104-A CDCL3" 10 1 "V:\Old Server\DCM\nmr"

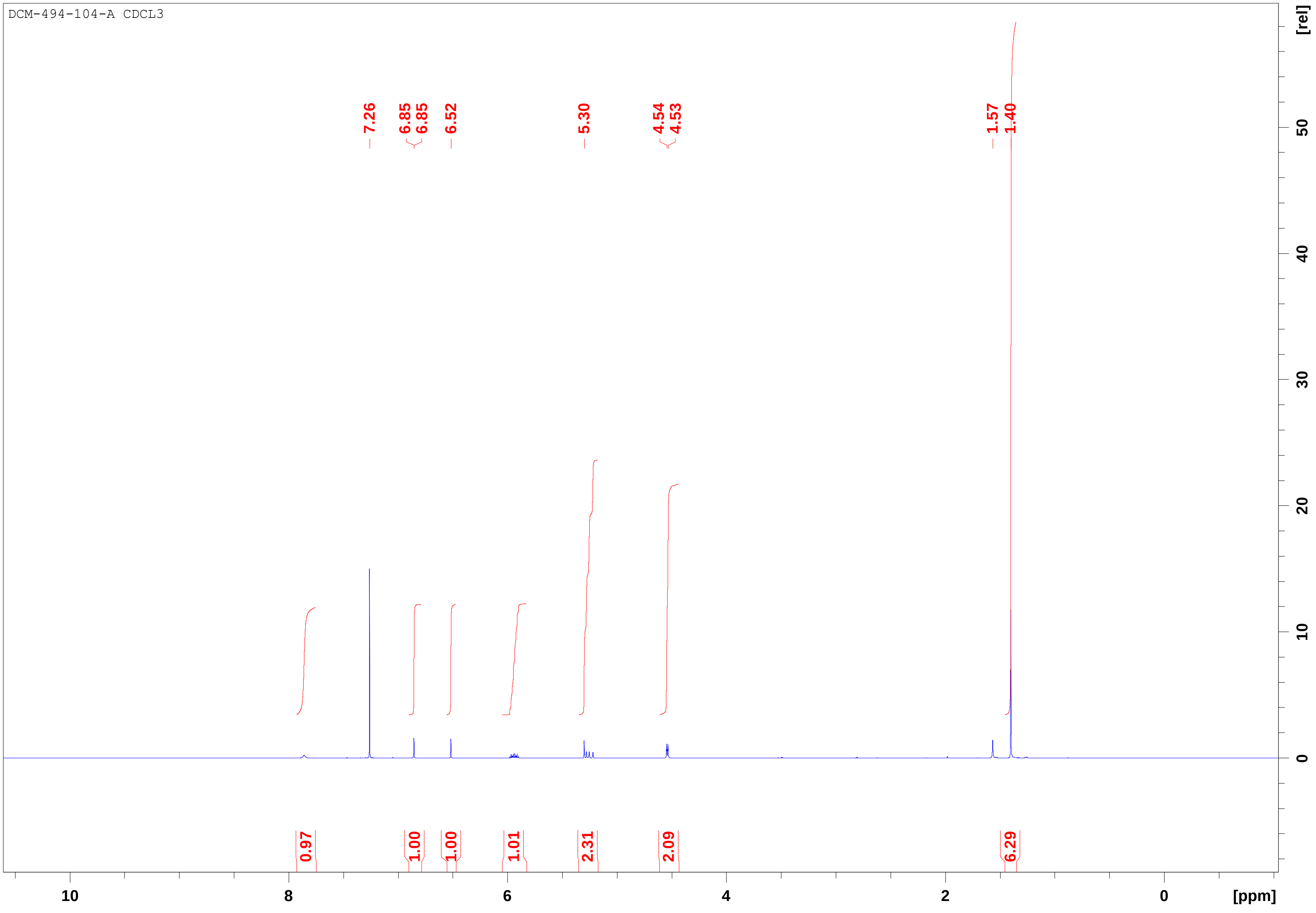

23

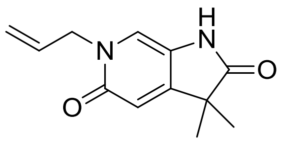

"DCM-494-104-A CDCL3" 14 1 "V:\Old Server\DCM\nmr"

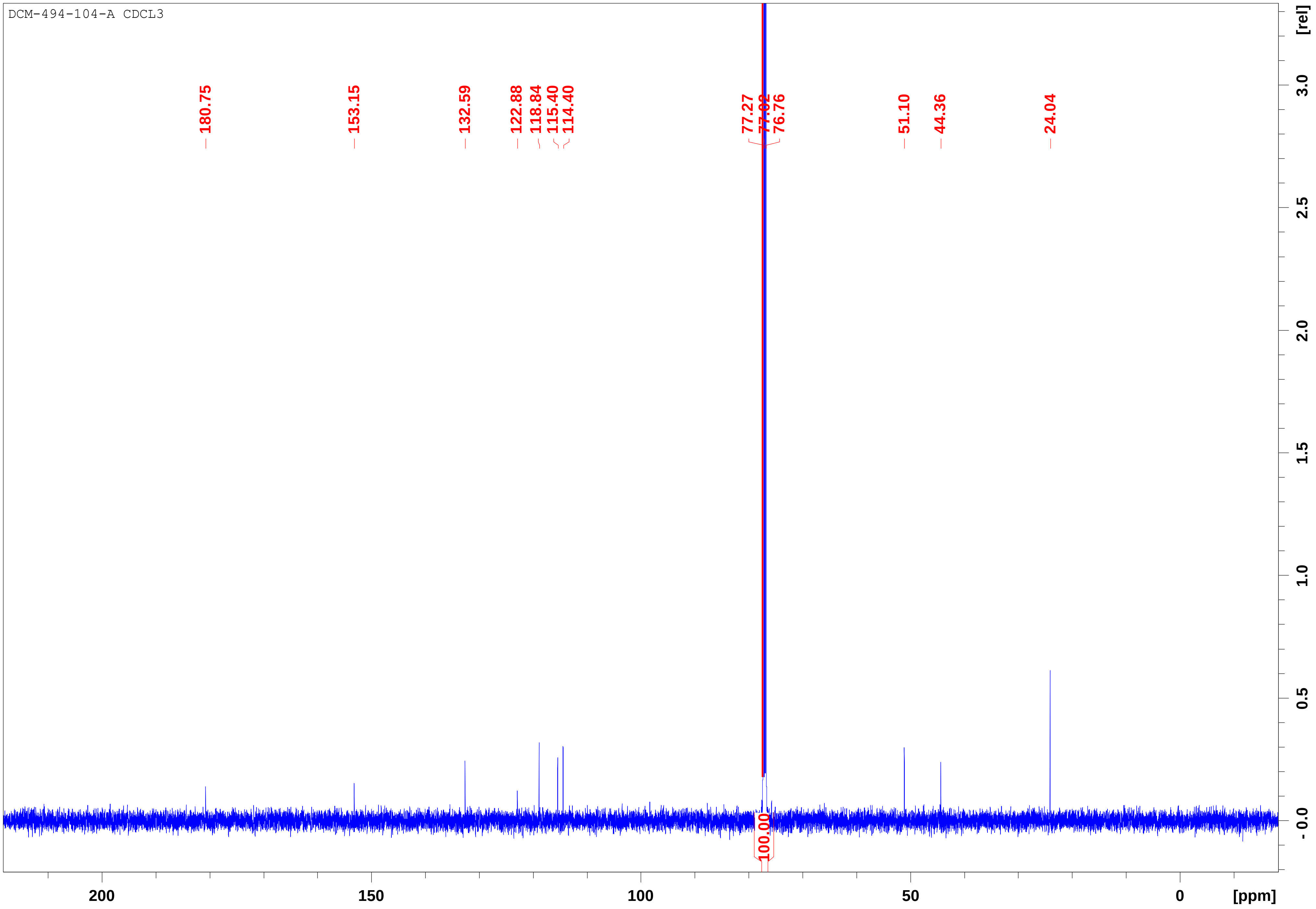

24

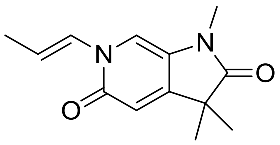

"DCM-494-108-A CDCL3" 10 1 "V:\Old Server\DCM\nmr"

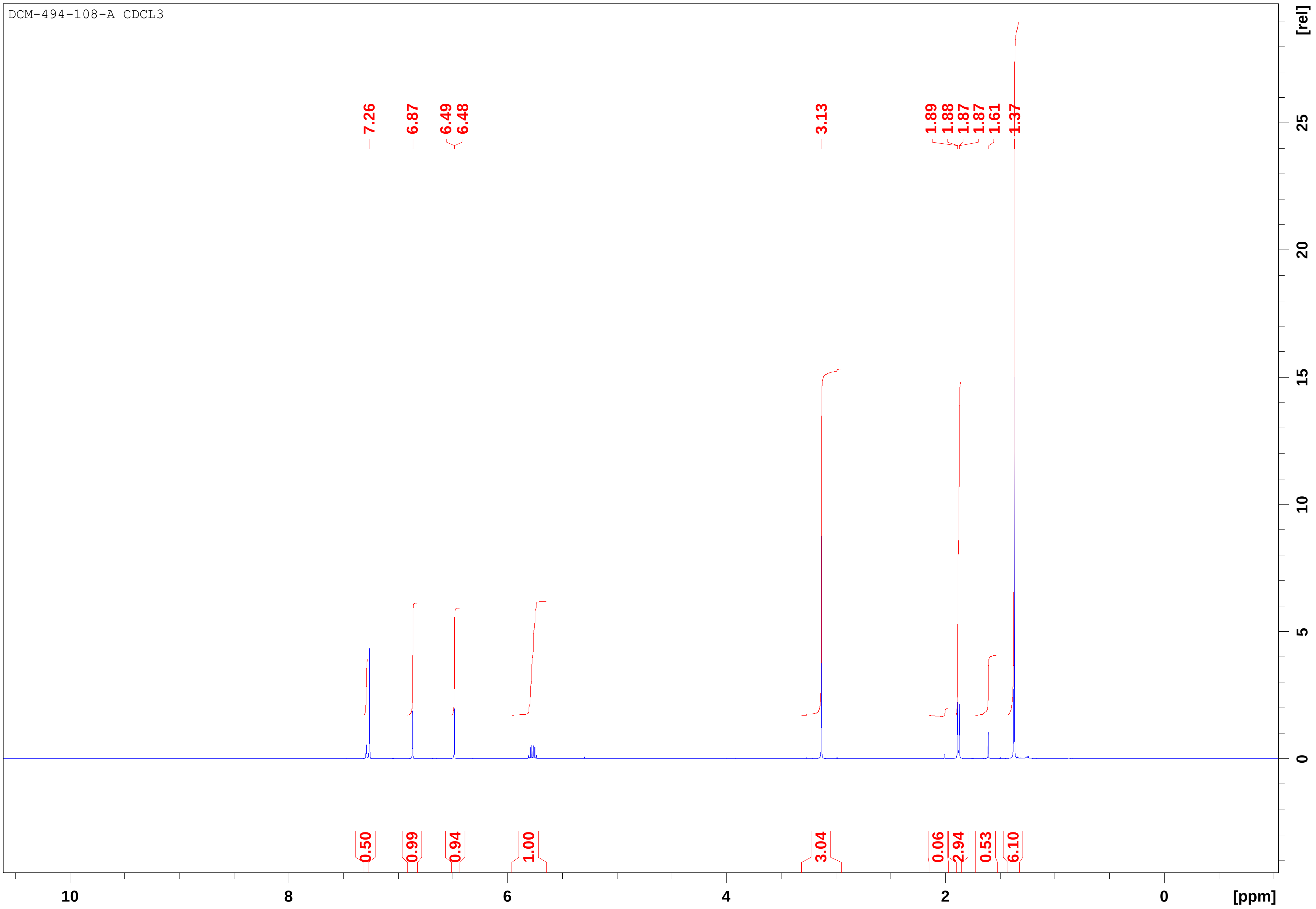

24

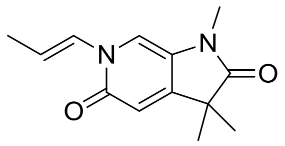

"DCM-494-108-A CDCL3" 11 1 "V:\Old Server\DCM\nmr"

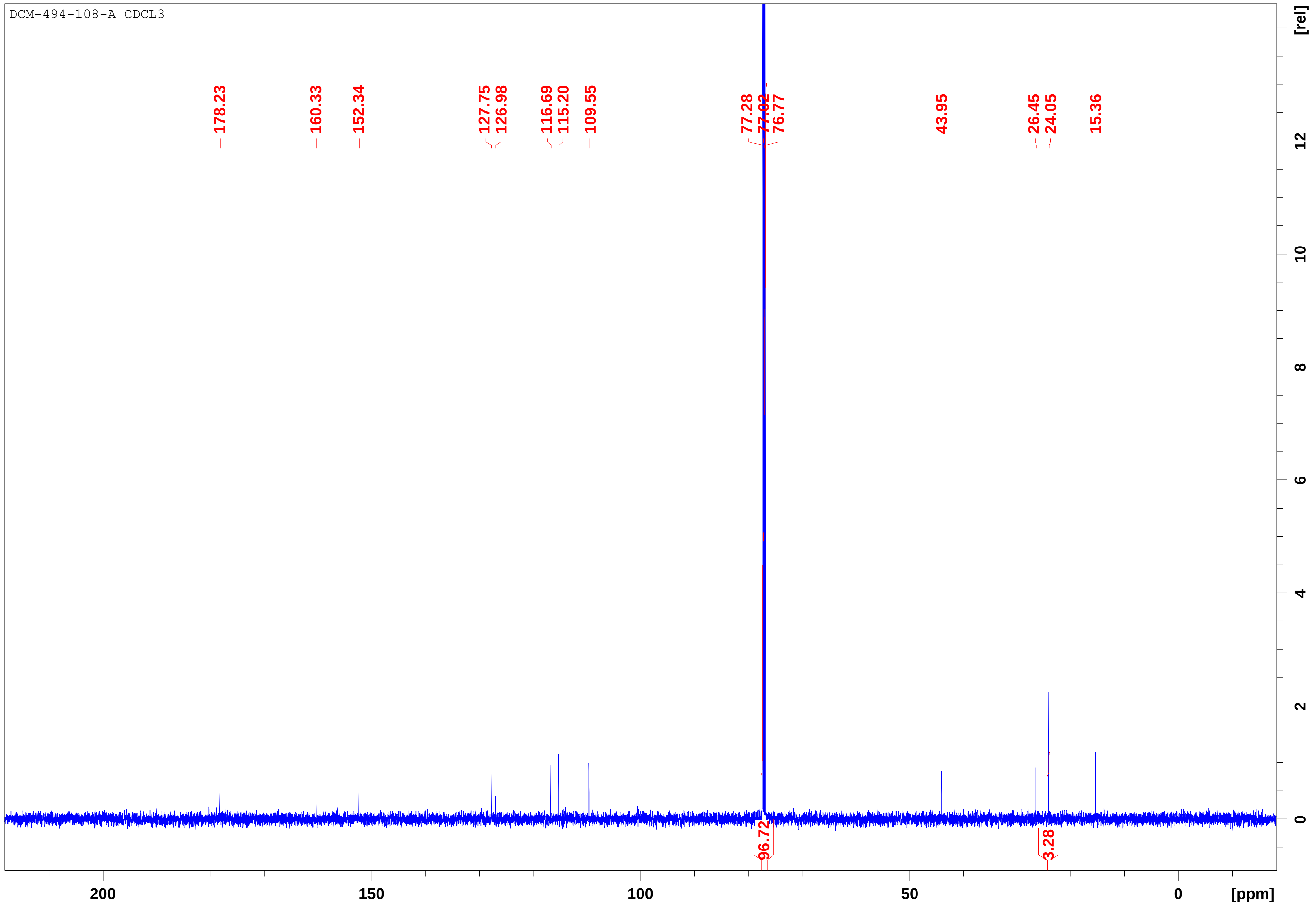

26

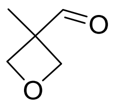

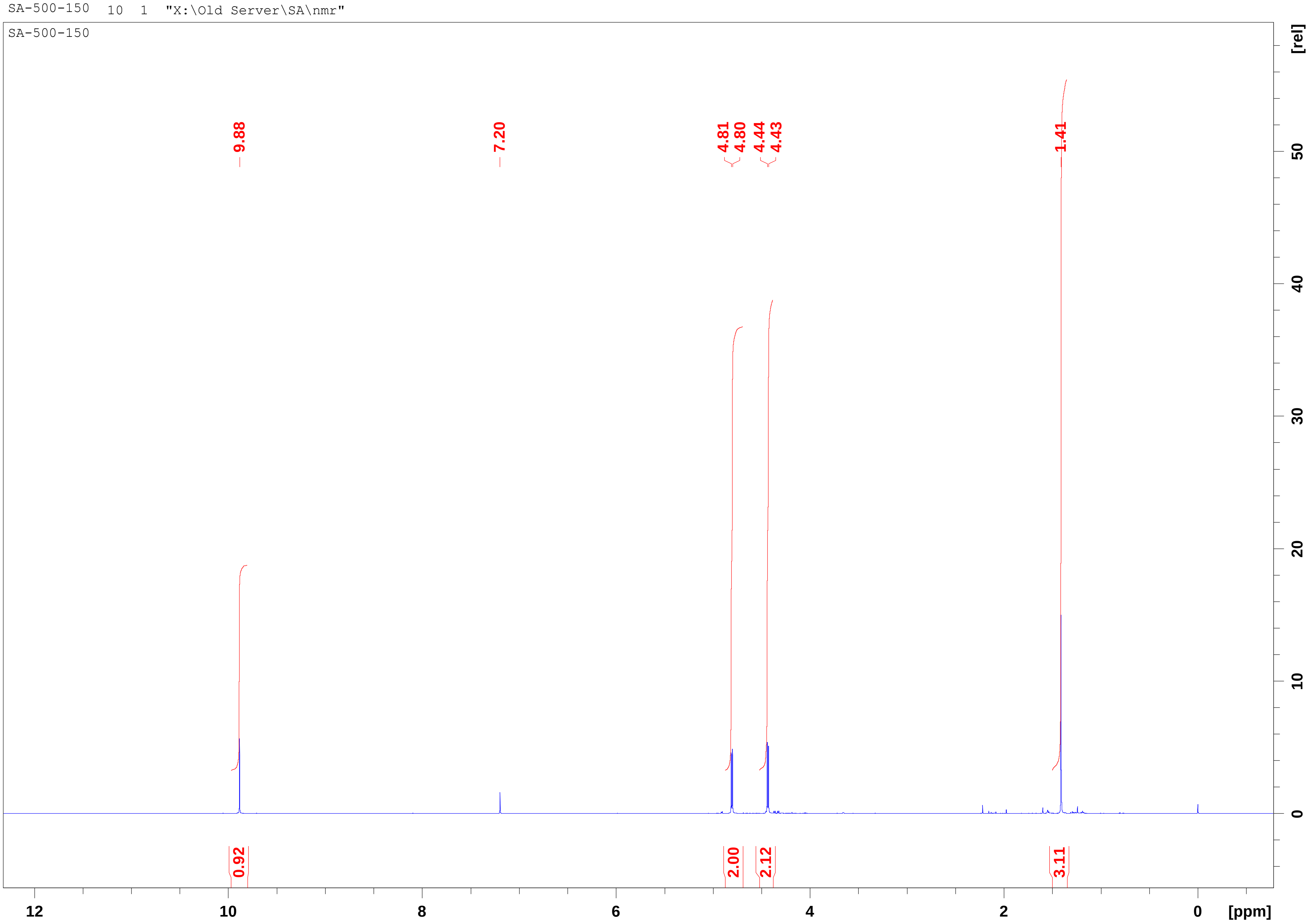

SA-500-150 10 1 "X:\Old Server\SA\nmr"
SA-500-150
9.88
7.20
4.81
4.80
4.44
4.43
1.41
0.92
2.00
2.12
3.11
12
10
8
6
4
2
0
[ppm]
[rel]
50
40
30
20
10
0

26

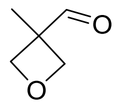

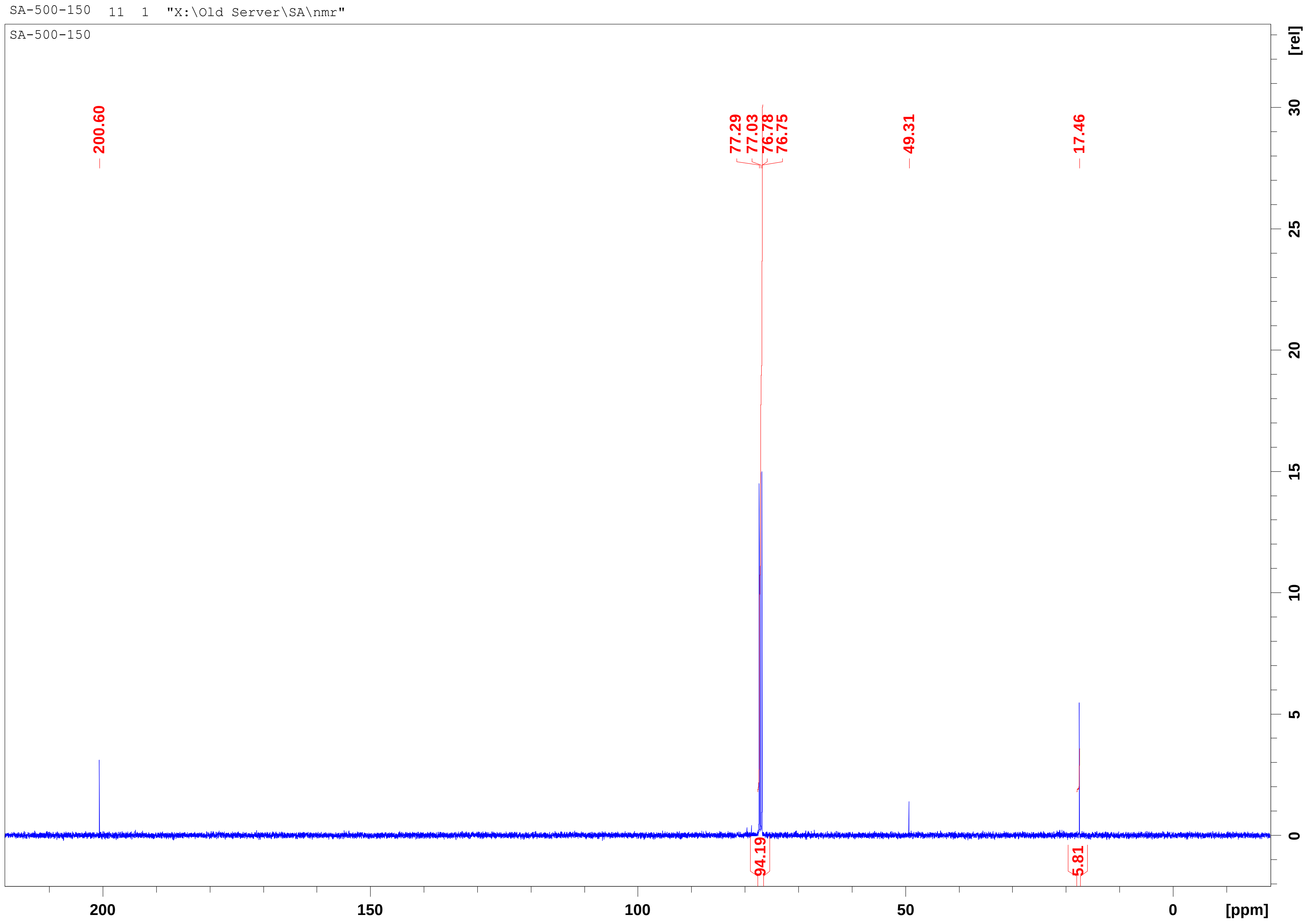
SA-500-150 11 1 "X:\Old Server\SA\nmr"
SA-500-150
200.60
77.29
77.03
76.78
76.75
49.31
17.46
94.19
5.81
[rel]
[ppm]
