# Supplementary material for: Identification of a novel ligand for the ATAD2 bromodomain with selectivity over BRD4 through a fragment growing approach
Source: Org Biomol Chem. 2018 Feb 15;16(11):1843–50. doi: 10.1039/c8ob00099a (PMC6102691; doi:10.1039/c8ob00099a)
Supplement: Supplementary file 2 [file OB-016-C8OB00099A-s002.pdf]

Electronic Supplementary Material (ESI) for Organic & Biomolecular Chemistry.
This journal is © The Royal Society of Chemistry 2018

Supplementary Information

For

Identification of a Novel Ligand for the ATAD2 Bromodomain with Selectivity Over BRD4 Through a Fragment Growing Approach.

Duncan C. Miller, Mathew P. Martin, Santosh Adhikari, Alfie Brennan, Jane a. Endicott, Bernard T. Golding, Ian R. Hardcastle, Amy Heptinstall, Stephen Hobson, Claire Jennings, Lauren Molyneux, Yvonne Ng, Stephen R. Wedge, Martin E. M. Noble, Celine Cano

Table of Contents

1.1 Protein Purification. 7

1.2 Isothermal Titration Calorimetry. 7

1.3 ATAD2-Histone displacement HTRF assay. 7

1.4 Surface Plasmon Resonance (SPR). 8

1.5 Crystallisation. 9

1.6 Supplementay Schemes 9

1.7 Summary of Generic Analytical and Chromatographic Conditions 10

1.8 General Synthetic Procedures 11

1.9 Synthetic Procedures 12

tert-butyl (6-methoxy-4-methylpyridin-3-yl)carbamate (7) 12

2-(5-((*Tert*-butoxycarbonyl)amino)-2-methoxypyridin-4-yl)acetic acid (8) 12

Tert-butyl 5-methoxy-2-oxo-2,3-dihydro-1*H*-pyrrolo[2,3-*c*]pyridine-1-carboxylate (9) 13

Tert-butyl 5-methoxy-3,3-dimethyl-2-oxo-2,3-dihydro-1*H*-pyrrolo[2,3-*c*]pyridine-1-carboxylate (10) 14

3,3,6-Trimethyl-1,6-dihydro-2*H*-pyrrolo[2,3-*c*]pyridine-2,5(3*H*)-dione (11) 14

1-Ethyl-3,3,6-trimethyl-1*H*-pyrrolo[2,3-c]pyridine-2,5(3*H*,6*H*)-dione (12a) 15

1-Benzyl-3,3,6-trimethyl-1,6-dihydro-2*H*-pyrrolo[2,3-*c*]pyridine-2,5(3*H*)-dione (12b) 15

3,3,6-Trimethyl-1-phenethyl-1,6-dihydro-2*H*-pyrrolo[2,3-*c*]pyridine-2,5(3*H*)-dione (12c) 16

3,3,6-Trimethyl-1-(2-(pyrrolidin-1-yl)ethyl)-1,6-dihydro-2*H*-pyrrolo[2,3-*c*]pyridine-2,5(3*H*)-dione (12d) 16

3,3,6-Trimethyl-1-(2-morpholinoethyl)-1,6-dihydro-2*H*-pyrrolo[2,3-*c*]pyridine-2,5(3*H*)-dione (12e) 17

1-(2-((*Tert*-butyldimethylsilyl)oxy)ethyl)-3,3,6-trimethyl-1,6-dihydro-2*H*-pyrrolo[2,3-*c*]pyridine-2,5(3*H*)-dione (12f) 18

1-(2-Hydroxyethyl)-3,3,6-trimethyl-1,6-dihydro-2*H*-pyrrolo[2,3-*c*]pyridine-2,5(3*H*)-dione (12g) 18

tert-Butyl 3,3-diethyl-5-methoxy-2-oxo-2,3-dihydro-1*H*-pyrrolo[2,3-c]pyridine-1-carboxylate (13) 19

1,3,3,6-Tetramethyl-1,6-dihydro-2*H*-pyrrolo[2,3-c]pyridine-2,5(3*H*)-dione (15a).........19

6-Allyl-3,3-dimethyl-1*H*-pyrrolo[2,3-c]pyridine-2,5(3*H*,6*H*)-dione (14b) 20

1,3,3-Trimethyl-6-(prop-1-en-1-yl)-1*H*-pyrrolo[2,3-c]pyridine-2,5(3*H*,6*H*)-dione (14a) 20

1,3,3-Trimethyl-6-propyl-1*H*-pyrrolo[2,3-c]pyridine-2,5(3*H*,6*H*)-dione (15b) 21

6-Benzyl-3,3-dimethyl-1*H*-pyrrolo[2,3-c]pyridine-2,5(3*H*,6*H*)-dione (14c) 21

6-Benzyl-1,3,3-trimethyl-1*H*-pyrrolo[2,3-c]pyridine-2,5(3*H*,6*H*)-dione (15c)..............22

3,3-Dimethyl-6-phenethyl-1*H*-pyrrolo[2,3-c]pyridine-2,5(3*H*,6*H*)-dione (14d)............22

1,3,3-Trimethyl-6-phenethyl-1*H*-pyrrolo[2,3-c]pyridine-2,5(3*H*,6*H*)-dione (15d).........23

Methyl 4-((3,3-dimethyl-2,5-dioxo-2,3-dihydro-1*H*-pyrrolo[2,3-c]pyridin-6(5*H*)-yl)methyl)benzoate (14e) 23

Methyl 4-((1,3,3-trimethyl-2,5-dioxo-2,3-dihydro-1*H*-pyrrolo[2,3-c]pyridin-6(5*H*)-yl)methyl)benzoate (15e) 24

4-((1,3,3-Trimethyl-2,5-dioxo-2,3-dihydro-1*H*-pyrrolo[2,3-c]pyridin-6(5*H*)-yl)methyl)benzoic acid (15f)..24

4-((3,3-Dimethyl-2,5-dioxo-1,2,3,5-tetrahydro-6*H*-pyrrolo[2,3-c]pyridin-6-yl)methyl)benzonitrile (14g) 25

4-((1,3,3-Trimethyl-2,5-dioxo-1,2,3,5-tetrahydro-6H-pyrrolo[2,3-c]pyridin-6-yl)methyl)benzonitrile (15g) 25

4-((1,3,3-Trimethyl-2,5-dioxo-1,2,3,5-tetrahydro-6*H*-pyrrolo[2,3-c]pyridin-6-yl)methyl)benzamide (15h)..26

3,3-Dimethyl-6-(4-(methylsulfonyl)benzyl)-1,6-dihydro-2*H*-pyrrolo[2,3-*c*]pyridine-2,5(3*H*)-dione (14i) 26

1,3,3-Trimethyl-6-(4-(methylsulfonyl)benzyl)-1,6-dihydro-2*H*-pyrrolo[2,3-*c*]pyridine-2,5(3*H*)-dione (15i) 27

6-(4-Chlorobenzyl)-3,3-dimethyl-1,6-dihydro-2*H*-pyrrolo[2,3-*c*]pyridine-2,5(3*H*)-dione (14j) 28

6-(4-Chlorobenzyl)-1,3,3-trimethyl-1,6-dihydro-2*H*-pyrrolo[2,3-*c*]pyridine-2,5(3*H*)-dione (15j) 28

6-(4-Bromobenzyl)-3,3-dimethyl-1,6-dihydro-2*H*-pyrrolo[2,3-*c*]pyridine-2,5(3*H*)-dione (14k) 29

6-(4-Bromobenzyl)-1,3,3-trimethyl-1,6-dihydro-2*H*-pyrrolo[2,3-*c*]pyridine-2,5(3*H*)-dione (15k) 29

3,3-Dimethyl-6-(4-methylbenzyl)-1,6-dihydro-2*H*-pyrrolo[2,3-*c*]pyridine-2,5(3*H*)-dione (14l) 30

1,3,3-Trimethyl-6-(4-methylbenzyl)-1,6-dihydro-2*H*-pyrrolo[2,3-*c*]pyridine-2,5(3*H*)-dione (15l) 30

3-Dimethyl-6-(4-(trifluoromethyl)benzyl)-1,6-dihydro-2*H*-pyrrolo[2,3-*c*]pyridine-2,5(3*H*)-dione (14m) 31

1,3,3-Trimethyl-6-(4-(trifluoromethyl)benzyl)-1,6-dihydro-2*H*-pyrrolo[2,3-*c*]pyridine-2,5(3*H*)-dione (15m) 31

6-(3,4-Dichlorobenzyl)-3,3-dimethyl-1,6-dihydro-2*H*-pyrrolo[2,3-*c*]pyridine-2,5(3*H*)-dione (14n) 32

6-(3,4-Dichlorobenzyl)-1,3,3-trimethyl-1,6-dihydro-2*H*-pyrrolo[2,3-*c*]pyridine-2,5(3*H*)-dione (15n) 32

2-((3,3-Dimethyl-2,5-dioxo-1,2,3,5-tetrahydro-6*H*-pyrrolo[2,3-*c*]pyridin-6-yl)methyl)benzonitrile (14o) 33

2-((1,3,3-Trimethyl-2,5-dioxo-1,2,3,5-tetrahydro-6*H*-pyrrolo[2,3-*c*]pyridin-6-yl)methyl)benzonitrile (15o) 33

3-((3,3-Dimethyl-2,5-dioxo-1,2,3,5-tetrahydro-6*H*-pyrrolo[2,3-*c*]pyridin-6-yl)methyl)benzonitrile (14p) 34

3-((1,3,3-trimethyl-2,5-dioxo-1,2,3,5-tetrahydro-6*H*-pyrrolo[2,3-*c*]pyridin-6-yl)methyl)benzonitrile (15p) 35

2-((1,3,3-Trimethyl-2,5-dioxo-1,2,3,5-tetrahydro-6*H*-pyrrolo[2,3-*c*]pyridin-6-yl)methyl)benzoic acid (15q) 35

6-(4-Chlorobenzyl)-3,3-diethyl-1*H*-pyrrolo[2,3-c]pyridine-2,5(3*H*,6*H*)-dione (16a) 36

6-(4-Chlorobenzyl)-3,3-diethyl-1-methyl-1*H*-pyrrolo[2,3-c]pyridine-2,5(3*H*,6*H*)-dione (17a) 36

3,3-Diethyl-6-(4-(trifluoromethyl)benzyl)-1*H*-pyrrolo[2,3-c]pyridine-2,5(3*H*,6*H*)-dione (16b) 37

3,3-Diethyl-1-methyl-6-(4-(trifluoromethyl)benzyl)-1*H*-pyrrolo[2,3-c]pyridine-2,5(3*H*,6*H*)-dione (17b) 37

3-Benzylidene-5-methoxy-1,3-dihydro-2*H*-pyrrolo[2,3-*c*]pyridin-2-one (20a) 38

3-Benzyl-5-methoxy-1,3-dimethyl-1,3-dihydro-2*H*-pyrrolo[2,3-*c*]pyridin-2-one (22a) .39

3-Benzyl-6-(4-chlorobenzyl)-1,3-dimethyl-1,6-dihydro-2*H*-pyrrolo[2,3-*c*]pyridine-2,5(3*H*)-dione (23a) 39

5-Methoxy-3-(pyridin-2-ylmethylene)-1,3-dihydro-2*H*-pyrrolo[2,3-*c*]pyridin-2-one (20b) 40

5-Methoxy-1,3-dimethyl-3-(pyridin-2-ylmethyl)-1,3-dihydro-2*H*-pyrrolo[2,3-*c*]pyridin-2-one (22b) 40

6-(4-Chlorobenzyl)-1,3-dimethyl-3-(pyridin-2-ylmethyl)-1,6-dihydro-2*H*-pyrrolo[2,3-*c*]pyridine-2,5(3*H*)-dione (23b) 41

5-Methoxy-3-((2-methylthiazol-4-yl)methylene)-1,3-dihydro-2H-pyrrolo[2,3-c]pyridin-2-one (20c) 42

5-Methoxy-1,3-dimethyl-3-((2-methylthiazol-4-yl)methyl)-1,3-dihydro-2H-pyrrolo[2,3-c]pyridin-2-one (22c) 42

6-(4-Chlorobenzyl)-1,3-dimethyl-3-((2-methylthiazol-4-yl)methyl)-1,6-dihydro-2H-pyrrolo[2,3-c]pyridine-2,5(3H)-dione (23c) 43

3-(2,2-Dimethylpropylidene)-5-methoxy-1*H*-pyrrolo[2,3-c]pyridin-2(3*H*)-one (20d) ...43

5-Methoxy-3-neopentyl-1*H*-pyrrolo[2,3-c]pyridin-2(3*H*)-one (22d) 44

5-Methoxy-1,3-dimethyl-3-neopentyl-1*H*-pyrrolo[2,3-c]pyridin-2(3*H*)-one (22d) 44

6-(4-Chlorobenzyl)-1,3-dimethyl-3-neopentyl-1*H*-pyrrolo[2,3-c]pyridine-2,5(3*H*,6*H*)-dione (23d) 45

3-Methyloxetane-3-carbaldehyde (26) 45

5-Methoxy-3-((3-methyloxetan-3-yl)methylene)-1,3-dihydro-2*H*-pyrrolo[2,3-*c*]pyridin-2-one (20e) 46

5-Methoxy-1,3-dimethyl-3-((3-methyloxetan-3-yl)methyl)-1,3-dihydro-2*H*-pyrrolo[2,3-*c*]pyridin-2-one (22e) 46

6-(4-Chlorobenzyl)-1,3-dimethyl-3-((3-methyloxetan-3-yl)methyl)-1,6-dihydro-2*H*-pyrrolo[2,3-*c*]pyridine-2,5(3*H*)-dione (23e) 47

3-(Cyclopropylmethylene)-5-methoxy-1,3-dihydro-2*H*-pyrrolo[2,3-c]pyridin-2-one (20f)..48

3-(Cyclopropylmethyl)-5-methoxy-1,3-dihydro-2*H*-pyrrolo[2,3-c]pyridin-2-one (21f).48

3-(Cyclopropylmethyl)-5-methoxy-1,3-dimethyl-1,3-dihydro-2*H*-pyrrolo[2,3-c]pyridin-2-one (22f)..49

6-(4-Chlorobenzyl)-3-(cyclopropylmethyl)-1,3-dimethyl-1,6-dihydro-2*H*-pyrrolo[2,3-c]pyridine-2,5(3*H*)-dione (23f) 49

1.1 Protein Purification.

The ATAD2 bromodomain 981-1108 was expressed with an N-terminal GST fusion from the pGEX-6P-1 plasmid. The plasmids were transformed into BL21 (DE3) pLysS Escherichia coli (Novagen) and then the cells grown in luria bertani (LB) media at 37 ˚C until an OD600 of 0.6 was reached. The temperature was reduced to 20 ˚C and expression induced using 0.2 mM Isopropyl β-D-1-thiogalactopyranoside (IPTG). The cells were harvested after overnight incubation and resuspended in buffer A (20 mM HEPES pH 7.4, 100 mM NaCl, 5 mM Dithiothreitol (DTT)). The cells were lysed by sonication and the lysate cleared by centrifugation (50,000 x g, 4 ˚C, 1 hr). The protein was passed over 5ml of glutathione Sepharose 4B resin (GE Healthcare) at 4 ˚C. The resin was then washed with buffer A and the protein eluted with buffer A supplemented with 10 mM glutathione. The protein was then applied to a Superdex 200 26/60 gel filtration column (GE Healthcare) equilibrated in buffer A.

1.2 Isothermal Titration Calorimetry.

ATAD2 was exchanged into ITC buffer (50 mM HEPES pH7.4, 200 mM NaCl, 1 mM DTT). ITC measurements were carried out at 25°C on a Microcal ITC 200 (Malvern Instruments Ltd, Malvern, UK). Compound **11** at 40mM, 2% DMSO was titrated into ATAD2 prepared in the cell at 150 μM, 2% DMSO. Titrations began with an initial 0.5 μL injection followed by 19x 2 μL injections of compound, with 120s spacing between each injection, and stirred at 1000 rpm throughout the experiment. Data were then analysed using Origin 7.0 (OriginLab Corp., Burlington, NC, USA) and fit with the one-set-of-sites model.

1.3 ATAD2-Histone displacement HTRF assay.

Compounds (dissolved to 100 mM in DMSO) were dispensed into black 384 well assay plates (Corning) over a final concentration range of 1000, 625, 500, 375, 200, 100, 50, 37.5, 25, and

12.5 µM using an Echo 550 (Labcyte). Each well was backfilled to a final volume of 200 nl (for the MDM2 assay), resulting in final DMSO concentrations of 2 %. 5 µl of GST-ATAD2 was added to each well and incubated for 30mins resulting in a final concentration of 5 nM. 5µl of biotinylated-histone H4 peptide (SGRG-K(Ac)-GG-K(Ac)-GLG-K(Ac)-GGA-K(Ac)-RHRKVGG-K(Biotin)) was then added to each well at a final concentration of 500nM. Both GST-ATAD2 and peptide were diluted in buffer B (50 mM Tris pH 7.5, 100 nM NaCl, 1 mM DTT, 100 µg/ml bovine serum albumin (BSA)). The plate was then incubated at room temperature for 30mins and then 5 µl LanthaScreen® Tb-anti-GST Antibody (Life Technologies) at final concentration of 5 nM was added to each well and incubated for an additional 30mins. 5 µl of streptavidin-XLL65 (Cisbio Assay) at a final concentration of 62.5 µM was added to each assay well. Both dyes were diluted in buffer C (50 mM Tris pH 7.5, 100 nM NaCl, 100 µg/ml BSA). The plate was incubated at room temperature for a further 30mins and then read using a PheraStar FS (BMG Labtech). The data were analysed using Graphpad Prism.

1.4 Surface Plasmon Resonance (SPR).

SPR-based ligand binding assays were performed using Biacore S200 (GE Healthcare) at 25 ºC using single cycle affinity. Immobilisation of ATAD2, BRD4, and inactive mutant ATAD2N1064A was achieved using standard amine coupling on a CM5 chip surface. The surface was prepared through activation with EDC/NHS, followed by injection of 10 µg/ml of respective proteins until target level 8000 RU was reached. The surface was then quenched using 1 M Ethanolamine and washed with running buffer 10 mM HEPES, 150mM NaCl, 0.01% TWEEN20, 0.5 mM TCEP and 1% DMSO with a flow rate of 30 µg/ml. Compounds were injected in a dose-response manner (8 concentration points ranging from 0-200 µM) in series across the control, ATAD2, BRD4, and ATAD2-N1064A flow cells using solvent correction to account for bulk refractive index changes. The reference control channel was subtracted

from immobilised channels and dose-response data was fitted using a steady state constant R_{max} 1:1 binding model, using an extrapolated R_{max} determined from a binding control compound in those cases where the extrapolated K_d value was greater than the top concentration used in the experiment (200 μM). K_d values are reported to the nearest 100th of micromolar.[1]

1.5 Crystallisation.

Crystallisation was performed at 20 °C using the hanging drop vapor diffusion method. Crystals of ATAD2 were grown by mixing 2μl ATAD2 at 7mg/ml and 2μl precipitant containing 0.1 M BIS-TRIS pH 6.5 and 2.0 M ammonium sulfate. Crystals were harvested in a diffusion solution containing 10 mM compound, 5% DMSO, 25 mM HEPES pH 7.5, 75 mM NaCl, 50 mM BIS-TRIS pH 6.5 and 1.0 M Ammonium Sulfate for 48hrs. Prior to data collection the crystals were cryo protected with the addition of 25% (v/v) ethylene glycol to the diffusion solution. X-ray diffraction data were recorded at Diamond Light Source (Harwell, Oxfordshire, UK). Data processing was carried out using XDS, POINTLESS/AIMLESS[2] and other programs of the CCP4i suite[3] run through the CCP4i2 gui. Structures were solved by molecular replacement using PHASER[4] and pdb 3DAI as a starting model. REFMAC[5] was employed for refinement, and model building was performed using COOT[6]. PDBs were deposited within the protein database www.pdb.org using accession codes: **11** XXXX, **15a** XXXX, **12a** XXXX, **12b** XXXX, **15c** XXXX, **15d** XXXX, **15f** XXXX, **15k** XXXX, **23e** XXXX, **23c** XXXX, **17a** XXXX.

1.6 Supplementay Schemes

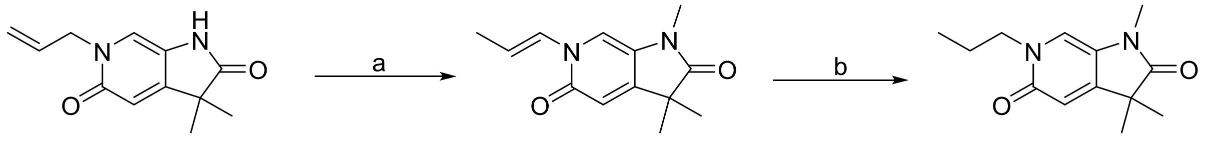

23 **24** **17b**

Scheme S1: *Reagents and conditions:* (a) NaH, MeI, DMF, r.t. 1 h, 44%; (b) H_2, 10% Pd/C, 40 °C, 2 h, 64%.

1.7 Summary of Generic Analytical and Chromatographic Conditions

All commercial reagents were purchased from Sigma-Aldrich Chemical Company, Alfa Aesar, Apollo Scientific or Tokyo Chemical Industry UK Ltd. The chemicals were of the highest available purity. Unless otherwise stated, chemicals were used as supplied without further purification. Anhydrous solvents were obtained from AcroSealTM or Aldrich SureSeal™ bottles and were stored under nitrogen. Petrol refers to the fraction with a boiling point between 40 and 60 °C. Thin layer chromatography utilised to monitor reaction progress was conducted on plates pre-coated with silica gel Merck 60F254 or Merck NH2F254S. The eluent was as stated (where this consisted of more than one solvent, the ratio is stated as volume:volume) and visualisation was either by short wave (254 nm) ultraviolet light, or by treatment with the visualisation reagent stated followed by heating. 'Flash' medium pressure liquid chromatography (MPLC) was carried out either on a Biotage SP4 automated purification system or a Varian 971-FP automated purification system, using pre-packed Varian or Grace silica or amino-bonded silica cartridges. All reactions carried out in a microwave were performed in a Biotage Initiator with Sixty robot. Melting points were determined using a VWR Stuart SMP40 apparatus and are uncorrected. ^{1}H, ^{13}C and ^{19}F nuclear magnetic resonance (NMR) spectra were obtained as either $CDCl_3$, CD_3OD or DMSO-d_6 solutions and recorded at 500 MHz, 126 MHz and 471 MHz, respectively, on a Bruker Avance III 500 spectrometer. Where ^{13}C NMR data are not quoted, insufficient material was available or problems obtaining high resolution spectra were encountered. Chemical shifts are quoted in parts per million (δ) referenced to the appropriate deuterated solvent employed. Multiplicities are indicated by s (singlet), d (doublet), t (triplet), q (quartet), quin (quintet), m (multiplet), br (broad) or combinations thereof. Coupling constant values are given in Hz. Homonuclear and heteronuclear two dimensional NMR experiments were used where appropriate to facilitate assignment of chemical shifts. LC-MS was carried out on a Waters Acquity UPLC system with PDA and ELSD employing positive or negative electrospray modes as appropriate to the individual 9 compound. Where LRMS data is not quoted, the mass was not recognised for that compound. High resolution mass spectrometry was performed by the EPSRC UK National Mass Spectrometry Facility, University of Wales Swansea, Singleton Park, Swansea, SA2 8PP. FTIR spectra were recorded on either a Bio-

Rad FTS 3000MX diamond ATR or an Agilent Cary 630 FTIR as a neat sample. UV spectra were obtained using a U-2001 Hitachi Spectrophotometer with the sample dissolved in ethanol.

1.8 General Synthetic Procedures

General Procedure A: Substitution at *N*¹

The relevant compound (1 eq.) and Cs_2CO_3 (3 eq.) were suspended in DMF. The relevant alkyl halide (2.5 eq.) was added and stirred at 100 ˚C under microwave irradiation for 30 min. The reaction was quenched with water (20 mL) and the aqueous layer was extracted with EtOAc (3×25 mL). The organic layers were combined, dried over $MgSO_4$ and the solvent removed *in vacuo*.

General Procedure A1: Substitution at *N*¹

The relevant compound (1 eq.) was dissolved in DMF. NaH (60% dispersion in oil) was added portion-wise and the mixture was stirred at r.t. for 15 min. The relevant alkyl halide (3 eq.) was added and the reaction was stirred at r.t. for 3 h. The reaction was quenched with water (20 mL) and the aqueous layer was extracted with EtOAc (3 × 20 mL). The organic layers were combined, dried over $MgSO_4$, the solvent removed *in vacuo* and the residue was purified by MPLC on SiO_2

General Procedure B: Pyridone formation

The relevant pyridine (1 eq.) and alkyl halide (2 eq.) were dissolved in MeCN and the mixture was stirred at 170 ˚C under microwave irradiation for 1 h. The solvent was removed *in vacuo* and the residue was purified by MPLC.

General Procedure C: Knoevenagel condensation

The relevant compound (1 eq.) was dissolved in THF, the relevant aldehyde (1.1 eq.) and piperidine (1.5-3.1 eq.) were added. The reaction mixture was stirred at 100 ˚C until the starting material was completely consumed. The solvent was removed in vacuo and the residue was purified by MPLC.

General Procedure D: Reduction of an alkene and subsequent methylation

Step A: The relevant Knoevenagel product (1 eq.) was dissolved in THF and MeOH. 10% Pd/C was added and the mixture was stirred at r.t. under an atmosphere of hydrogen until the starting

material was completely consumed. The mixture was filtered through a Celite plug and the plug washed with MeOH. The solvent was removed *in vacuo* and the crude was taken forward without further purification.

Step B: The crude was dissolved in DMF, Cs_2CO_3 (2-2.5 eq.) and MeI (1.8-2.1 eq.) were added and the mixture was stirred at 60 ˚C for 1 h. The reaction was quenched with water (20 mL) and the aqueous layer was extracted with EtOAc (3×25 mL). The organic layers were combined, dried over $MgSO_4$ and the solvent removed *in vacuo*.

1.9 Synthetic Procedures

***tert*-butyl (6-methoxy-4-methylpyridin-3-yl)carbamate (9)**[7]

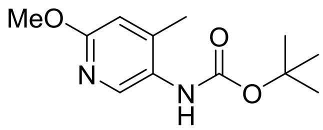

5-Amino-2-methoxy-4-picoline (2.03 g, 14.7 mmol) and di-*tert*-butyl dicarbonate (3.20 g, 14.7 mmol) were dissolved in THF (32.6 ml). Saturated aqueous Na_2CO_3 (8.5 ml) was added and the mixture was stirred at room temperature for 24 hours. The volatiles were removed *in vacuo* and the residue partitioned between DCM and water. The aqueous layer was washed with DCM (×3), the combined organic extracts washed with brine, dried *via* phase separator, and evaporated *in vacuo*. The mixture was purified by MPLC on silica gel (10-50% EtOAc/petrol) to give the product as a white solid (3.45 g, 98%). ^{1}H-NMR (500 MHz, $CDCl_3$) δ ppm: 1.50 (9H, s, *tert*-butyl C*H*$_3$), 2.23 (3H, s, Ar-C*H*$_3$), 3.89 (3H, s, Ar-OC*H*$_3$), 5.96 (1H, br s), 6.58 (1H, s, *H*-5), 8.17 (1H, br s, N-*H*); ^{13}C-NMR (125 MHz, $CDCl_3$) δ ppm: 17.8 (Ar-*C*H$_3$), 28.3 (*tert*-butyl *C*H$_3$), 53.6 (Ar-O*C*H$_3$), 80.7 (*tert*-butyl *C*(CH$_3$)$_3$), 111.3 (*C*-5), 127.3 (*C*-3), 142.5 (*C*-2), 145.1 (*C*-4), 154.0 (*C*-6), 161.8 (*C*=O); MS (ES$^+$) *m/z* 239.3 [M+H]$^+$.

2-(5-((Tert-butoxycarbonyl)amino)-2-methoxypyridin-4-yl)acetic acid (10)[8]

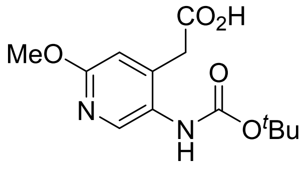

Compound **9** (4 g, 16.78 mmol) was dissolved in THF (71 mL) and cooled to -78 ˚C. *sec*-BuLi in cyclohexane (36 mL, 1.4 M, 50.34 mmol) was added dropwise and the reaction mixture was

stirred at -78 ˚C for 15 min. Five pellets of dry ice were added and the reaction mixture was allowed to warm to r.t. over a period of 45 min. The reaction was quenched with water (50 mL) and saturated aqueous $NaHCO_3$ (50 mL). The aqueous layer was washed with EtOAc (2 × 100 mL), and then acidified with 2 M aqueous HCl, stirred for 15 min and extracted with EtOAc (2×100 mL). The organic layers were combined, dried over $MgSO_4$ and the solvent removed *in vacuo* to give an off-white solid (3.5 g, 74%). R_f = 0.42 (20% MeOH/DCM); m.p. 116.120 °C; λ_{max} (EtOH)/nm 284.2, 234.6; IR ν_{max}/cm^{-1} 3309, (NH), 2981, 2939, 2872, 2782, 1690 (C=O); ^{1}H NMR (500 MHz, DMSO-*d*$_6$) δ_H 1.43 (9H, s, C(C*H*$_3$)$_3$), 3.58 (2H, s, C*H*$_2$COOH), 3.82 (3H, s, OC*H*$_3$), 6.74 (1H, s, 1×pyridine-*H*), 7.97 (1H, s, 1×pyridine-*H*), 8.62 (1H, br s, N*H*), 12.44 (1H, br s, COO*H*); ^{13}C NMR (125 MHz, DMSO-*d*$_6$) δ_C 28.6 (C(*C*H$_3$)$_3$), 36.9 (*C*H$_2$CO), 53.7 (O*C*H$_3$), 79.4 (*C*(CH$_3$)$_3$), 111.9 (CH-pyridine), 128.4 (C-pyridine), 143.7 (CH-pyridine), 144.4 (C-pyridine), 154.5 (*C*OO^{t}Bu), 161.6 (pyridine-*C*-OMe), 171.6 (*C*OOH); HRMS calcd for $C_{13}H_{19}N_2O_5$ [M+H]$^+$ 283.1288, found 283.1288.

***Tert*-butyl 5-methoxy-2-oxo-2,3-dihydro-1*H*-pyrrolo[2,3-*c*]pyridine-1-carboxylate (11)[8]**

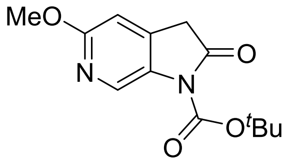

Compound **10** (1.47 g, 5.20 mmol), and tetrabutylammonium acetate (50 mg, 0.16 mmol) were dissolved in acetic anhydride (27 mL) and the mixture was stirred at 65 ˚C for 1 h. The solvent was removed *in vacuo*, water (30 mL) was added and the aqueous layer was extracted with DCM (3×40 mL). The organic layers were combined, washed with $NaHCO_3$ (3×30 mL), dried over $MgSO_4$ and evaporated *in vacuo*. Purification by MPLC on SiO_2 (DCM:MeOH, 0-5%) gave a white solid (1.11 g, 81%). R_f = 0.71 (5% MeOH/DCM); m.p. 161-163 °C; λ_{max} (EtOH)/nm 338.2, 230.0; IR ν_{max}/cm^{-1} 3309, (NH), 2981, 2939, 2872, 2782, 1690 (C=O), 1628 (C=O); ^{1}H NMR (500 MHz, DMSO-*d*$_6$) δ_H 1.57 (9H, s, C(C*H*$_3$)$_3$), 3.76 (2H, d, *J* = 1.0 Hz, C*H*$_2$CO), 3.83 (3H, s, OC*H*$_3$), 6.82 (1H, m, 1×pyridine-*H*), 8.37 (1H, s, 1×pyridine-*H*); ^{13}C NMR (125 MHz, DMSO-*d*$_6$) δ_C 28.1 (C(*C*H$_3$)$_3$), 36.6 (*C*H$_2$CO), 53.8 (O*C*H$_3$), 84.2 (*C*(CH$_3$)$_3$), 106.9 (CH-pyridine), 131.1 (CH-pyridine), 133.4 (C-pyridine), 138.4 (C-pyridine), 148.9 (*C*OO^{t}Bu), 160.3 (pyridine-*C*-OMe), 171.7 (*C*=O pyrrolidinone); HRMS calcd for $C_{13}H_{17}N_2O_4$ [M+H]$^+$ 265.1183, found 265.1187.

***Tert*-butyl 5-methoxy-3,3-dimethyl-2-oxo-2,3-dihydro-1*H*-pyrrolo[2,3-*c*]pyridine-1-carboxylate (12)**[8]

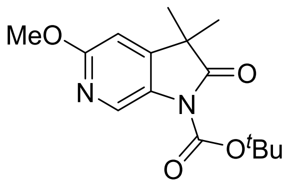

Compound **11** (1.10 g, 4.16 mmol) and Cs_2CO_3 (3 g, 9.15 mmol) were suspended in MeCN (29 mL). MeI (0.65 mL, 10.4 mmol) was added and the mixture was stirred at 60 ˚C for 3 h. The insoluble solids were removed by filtration, washed with EtOAc and the filtrate was evaporated *in vacuo*. The residue was purified by MPLC on SiO_2 (EtOAc:petrol, 0-20%) to give a white solid (945 mg, 78%). R_f = 0.77 (5% MeOH/DCM); m.p. 175-178 °C; λ_{max} (EtOH)/nm 295.0, 233.0; IR ν_{max}/cm^{-1} 2983, 2936, 1785 (C=O), 1630 (C=O); ^{1}H NMR (500 MHz, $CDCl_3$) δ_H 1.41 (6H, s, 2×C*H*$_3$), 1.65 (9H, s, C(C*H*$_3$)$_3$), 3.92 (3H, s, OC*H*$_3$), 6.62 (1H, d, *J* = 0.6 Hz, 1×pyridine-*H*), 8.54 (1H, d, *J* = 0.6 Hz, 1×pyridine-*H*); ^{13}C NMR (125 MHz, $CDCl_3$) δ_C 24.8 (2×*C*H$_3$), 28.1 (C(*C*H$_3$)$_3$), 44.7 (*C*(CH$_3$)$_2$), 53.7 (O*C*H$_3$), 84.8 (*C*(CH$_3$)$_3$), 104.8 (CH-pyridine), 130.2 (C-pyridine), 132.1 (CH-pyridine), 147.3 (C-pyridine), 148.9 (*C*OO^{t}Bu), 161.1 (pyridine-*C*-OMe), 178.4 (C=O pyrrolidinone); HRMS calcd for $C_{15}H_{21}N_2O_4$ $[M+H]^+$ 293.1496, found 293.1497.

3,3,6-Trimethyl-1,6-dihydro-2*H*-pyrrolo[2,3-*c*]pyridine-2,5(3*H*)-dione (7)

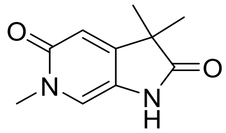

Prepared according to general procedure B using compound **12** (630 mg, 2.15 mmol), MeCN (15 mL) and MeI (0.33 mL, 5.37 mmol). The crude material was purified by MPLC on SiO_2 (MeOH/DCM, 0-10%) to give a white solid (148 mg, 100%). R_f = 0.15 (5% MeOH:DCM); m.p. 162-163°C; λ_{max} (EtOH)/nm 336.2, 255.2, 218.6; IR ν_{max}/cm^{-1} 3455 (NH), 3081, 3019, 2960, 2926, 2705, 2663, 1705 (C=O), 1589 (C=O); ^{1}H NMR (500 MHz, DMSO-*d*$_6$) δ_H 1.25 (6H, s, 2×C*H*$_3$), 3.40 (3H, s, OC*H*$_3$), 6.55 (1H, s, 1×pyridone-*H*), 7.21 (1H, s, 1×pyridone-*H*), 7.78 (1H, br s, N*H*); ^{13}C NMR (125 MHz, DMSO-*d*$_6$) δ_C 24.0 (2×*C*H$_3$), 37.4 (*C*(CH$_3$)$_2$), 44.1 (N*C*H$_3$), 113.7 (CH-pyridone), 117.2 (CH-pyridone), 124.1 (C-pyridone), 154.1 (C-pyridone), 160.8 (C=O pyridone), 180.2 (C=O pyrrolidinone); HRMS calcd for $C_{10}H_{13}N_2O_2$ $[M+H]^+$ 193.0972, found 193.0968.

1-Ethyl-3,3,6-trimethyl-1*H*-pyrrolo[2,3-c]pyridine-2,5(3*H*,6*H*)-dione (14a)

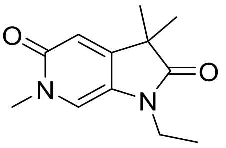

Prepared according to general procedure A1 using **7** (40 mg, 0.21 mmol), NaH (20 mg, 80% dispersion in oil, 0.66 mmol), THF (1 mL), DMF (2 mL) and EtI (33 μL, 0.42 mmol). Purification by MPLC on silica (MeOH:DCM, 0-5%) gave the product as a white solid (28 mg, 65%); R_f 0.30 (5% MeOH:DCM); m.p. 128.9-131.7 ºC; IR ν_{max}/cm^{-1} 3044, 2973, 1718 (C=O), 1591 (C=O); ^{1}H NMR (500 MHz; CD_3OD) δ_H 1.25 (3H, t, J = 7.1 Hz, CH_3CH_2), 1.38 (6H, s, 2 × Me), 3.60 (3H, s, NMe), 3.72 (2H, q, J = 7.1 Hz, CH_3CH_2), 6.61 (1H, s, H-Ar), 7.45 (1H, s, H-Ar); ^{13}C NMR (125 MHz; CD_3OD) δ_C 10.9, 22.6, 34.7, 36.8, 44.1, 113.1, 116.5, 125.9, 153.5, 162.6, 178.8; HRMS calcd for $C_{12}H_{17}N_2O_2$ [M+H]$^+$ 221.1285, found 221.1279.

1-Benzyl-3,3,6-trimethyl-1,6-dihydro-2*H*-pyrrolo[2,3-*c*]pyridine-2,5(3*H*)-dione (14b)

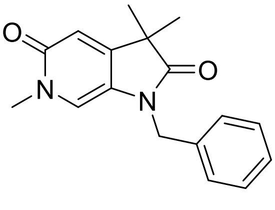

Prepared according to general procedure A1 using compound **7** (50 mg, 0.26 mmol) DMF (3 mL), NaH (60% in mineral oil, 31 mg, 0.78 mmol) and benzyl bromide (62 μL, 0.52 mmol) The crude material was purified by MPLC on SiO_2 (MeOH:DCM, 0-5%) to give a beige solid (45 mg, 61%). R_f= 0.35 (5% MeOH/DCM); m.p. 122-124°C; λ_{max} (EtOH)/nm 257.8, 236.8; IR ν_{max}/cm^{-1} 3090, 3056, 2972, 2924, 2852, 1703 (C=O), 1593 (C=O); ^{1}H NMR (500 MHz, DMSO-d_6) δ_H 1.32 (6H, s, 2×CH_3), 3.32 (3H, s, N-CH_3), 4.75 (2H, s, CH_2Ph), 6.57 (1H, s, 1×pyridone-*H*), 7.28-7.37 (6H, m, 6×Ar*H*); ^{13}C NMR (125 MHz, DMSO-d_6) δ_C 24.2 (2×CH_3), 37.3 (N-CH_3), 43.2 (CH_2-phenyl), 43.9 ($C(CH_3)_2$), 114.2 (CH-pyridone), 117.2 (CH-pPyridone), 124.3 (C-Ar), 127.6 (2×*C*-Ar), 127.9 (C-Ar), 129.2 (2×*C*-Ar), 136.3 (C-Ar), 152.2 (C-Ar), 161.2 (C=O pyridone), 178.3 (C=O pyrrolidinone); HRMS calcd for $C_{17}H_{19}N_2O_2$ [M+H]$^+$ 283.1441, found 283.1440.

3,3,6-Trimethyl-1-phenethyl-1,6-dihydro-2*H*-pyrrolo[2,3-*c*]pyridine-2,5(3*H*)-dione (14c)

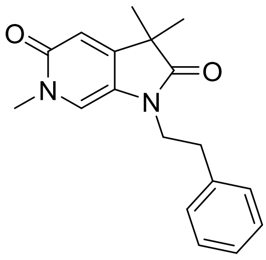

Prepared according to general procedure A1 using **7** (50 mg, 0.26 mmol), DMF (3 mL), NaH (60% in mineral oil, 31 mg, 0.78 mmol) and (2-bromoethyl)benzene (90 µL, 0.65 mmol). Purification by MPLC on SiO_2 (DCM:MeOH, 0-5%) gave a beige solid (23 mg, 30%). R_f = 0.36 (5% MeOH/DCM); λ_{max} (EtOH)/nm 337.0, 258.2; IR ν_{max}/cm^{-1} 3059, 2967, 2926, 2866, 1706 (C=O), 1590 (C=O); ^{1}H NMR (500 MHz, $CDCl_3$) δ_H 1.28 (6H, s, 2×C*H$_3$*), 2.94 (2H, t, *J* = 6.9 Hz, CH_2CH_2Ph), 3.39 (3H, s, N-C*H$_3$*), 3.82 (2H, t, *J* = 6.9 Hz, CH_2CH_2Ph), 6.26 (1H, s, 1×pyridone-*H*), 6.44 (1H, s, 1×pyridone-*H*), 7.15-7.17 (2H, m, 2×Ar*H*), 7.22-7.29 (3H, m, 3×Ar*H*); ^{13}C NMR (125 MHz, $CDCl_3$) δ_C 24.0 (2×*C*H$_3$), 33.7 (CH_2CH_2Ph), 37.6 (N-*C*H$_3$), 42.0 (CH_2CH_2Ph), 43.8 (*C*($CH_3)_2$), 114.6 (CH-pyridone), 114.7 (CH-pyridone), 125.6 (C-Ar), 126.9 (*C*-Ar), 128.7 (2×C-Ar), 129.0 (2×*C*-Ar), 138.2 (C-Ar), 152.2 (C-Ar), 161.7 (C=O pyridone), 178.3 (C=O pyrrolidinone); HRMS calcd for $C_{18}H_{21}N_2O_2$ $[M+H]^+$ 297.1598, 297.1597.

3,3,6-Trimethyl-1-(2-(pyrrolidin-1-yl)ethyl)-1,6-dihydro-2*H*-pyrrolo[2,3-*c*]pyridine-2,5(3*H*)-dione (14d)

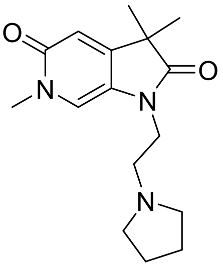

Prepared according to general procedure A using compound **7** (43 mg, 0.22 mmol), Cs_2CO_3 (287 mg, 0.88 mmol), 1-(2-chloroethyl)pyrrolidine hydrochloride (94 mg, 0.55 mmol) and DMF (2.5 mL). The product is insoluble in EtOAc, therefore aqueous layer was collected and the evaporated *in vacuo*. The residue was purified by MPLC (MeCN:H_2O, reversed phase with 0.1% HCOOH modifier, 0-50%) to give a white solid (25 mg, 40%). R_f = 0.15 (5% MeOH/DCM); λ_{max} (EtOH)/nm 336.0, 257.8, 220.0; IR ν_{max}/cm^{-1} 3029, 2962, 2923, 2787, 1712 (C=O), 1587 (C=O); ^{1}H NMR (500 MHz, CD_3OD) δ_H 1.26 (6H, s, 2×C*H$_3$*), 1.69-1.72

(4H, m, 4×pyrrolidine-*H*), 2.51-2.54 (4H, m, 4×pyrrolidine-*H*), 2.65 (2H, t, *J* = 6.9 Hz, 2×CON-CH_2CH_2), 3.47 (3H, s, CH_3), , 3.70 (2H, t, *J* = 6.9 Hz, 2×CON-CH_2), 6.47 (1H, s, 1×pyridone-*H*), 7.32 (1H, s, 1×pyridone-*H*); ^{13}C NMR (125 MHz, CD_3OD) δ_C 22.7 (2×CH_3), 22.9 (2×CH_2-pyrrolidine), 36.9 (N-CH_3), 38.9 (CON-CH_2), 44.1 ($C(CH_3)_2$), 51.6 (CON-CH_2CH_2), 53.7 (2× CH_2-pyrrolidine), 113.1 (CH-pyridone), 116.6 (CH-pyridone), 126.2 (C-pyridone), 153.5 (C-Pyridone), 162.5 (C=O pyridone), 179.2 (C=O pyrrolidine); HRMS calcd for $C_{16}H_{24}N_3O_2$ $[M+H]^+$ 290.1863, found 290.1865.

3,3,6-Trimethyl-1-(2-morpholinoethyl)-1,6-dihydro-2*H*-pyrrolo[2,3-*c*]pyridine-2,5(3*H*)-dione (14e)

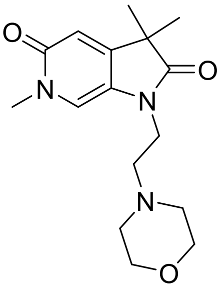

Prepared according to general procedure A using compound **7** (43 mg, 0.22 mmol), Cs_2CO_3 (287 mg, 0.88 mmol), 4-(2-chloroethyl)morpholine hydrochloride (102 mg, 0.55 mmol) and DMF (2.5 mL). The product is insoluble in EtOAc, therefore aqueous layer was collected and the evaporated *in vacuo*. The residue was purified by MPLC (MeCN:H_2O, reversed phase with 0.1% HCOOH modifier, 0-50%) to give a white solid (55 mg, 82%). R_f = 0.15 (5% MeOH/DCM); m.p. 172-174°C; λ_{max} (EtOH)/nm 336.8, 258.2, 220.6; IR ν_{max}/cm^{-1} 3034, 2939, 2852, 2811, 1712 (C=O), 1590 (C=O); ^{1}H NMR (500 MHz, CD_3OD) δ_H 1.27 (6H, s, 2×CH_3), 2.41 (4H, br s, 4×morpholine-*H*), 2.52 (2H, t, *J* = 6.4 Hz, 2×CON-CH_2CH_2), 3.46 (3H, s, CH_3), 3.51 (4H, br t, *J* = 4.5 Hz, 4×morpholine-*H*), 3.70 (2H, t, *J* = 6.4 Hz, 2×CON-CH_2), 6.47 (1H, s, 1×pyridone-*H*), 7.34 (1H, s, 1×pyridone-*H*); ^{13}C NMR (125 MHz, CD_3OD) δ_C 22.8 (2×CH_3), 36.8 (CON-CH_2), 36.9 (N-CH_3), 44.0 ($C(CH_3)_2$), 53.2 (2×CH_2-morpholine), 53.9 (CON-CH_2CH_2), 66.6 (2×CH_2-morpholine), 113.0 (CH-pyridone), 116.8 (CH-pyridone), 126.1 (C-pyridone), 153.5 (C-pyridone), 162.5 (C=O pyridone), 179.4 (C=O pyrrolidinone); HRMS calcd for $C_{16}H_{24}N_3O_3$ $[M+H]^+$ 306.1812, found 306.1812.

1-(2-((*Tert*-butyldimethylsilyl)oxy)ethyl)-3,3,6-trimethyl-1,6-dihydro-2*H*-pyrrolo[2,3-*c*]pyridine-2,5(3*H*)-dione (14f)

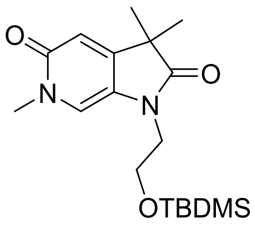

Prepared according to general procedure A using compound **7** (100 mg, 0.52 mmol), Cs_2CO_3 (508 mg, 1.56 mmol), (2-bromoethoxy)(tert-butyl)dimethylsilane (311 mg, 1.30 mmol) and DMF (4.5 mL). Purification by MPLC on SiO_2 (MeOH:DCM, 0-5%) gave an orange oil (120 mg, 55%). R_f = 0.20 (5% MeOH/DCM); λ_{max} (EtOH)/nm 336.2, 257.6, 219.8; IR ν_{max}/cm^{-1} 2929, 2855, 1703 (C=O), 1589 (C=O); ^{1}H NMR (500 MHz, DMSO-d_6) δ_H 0.00 (6H, s, 2×C*H*-$_3$ of TBDMS), 0.82 (9H, s, C(CH_3)$_3$ of TBDMS), 1.30 (6H, s, 2×CH_3), 3.42 (3H, s, N-CH_3), 3.71 (2H, t, *J* = 5.3 Hz, CH_2CH$_2$OTBDMS), 3.83 (2H, t, *J* = 5.3 Hz, CH$_2$CH_2OTBDMS), 6.56 (1H, s, 1×pyridone-*H*), 7.48 (1H, s, 1×pyridone-*H*); ^{13}C NMR (125 MHz, DMSO-d_6) δ_C -5.1 (2×*C*H$_3$ of TBDMS), 18.2 (*C*(CH$_3$)$_3$ of TBDMS), 24.2 (2×*C*H$_3$), 26.0 (C(*C*H$_3$)$_3$ of TBDMS), 37.2 (N-*C*H$_3$), 42.6 (CH$_2$*C*H$_2$OTBDMS), 43.7 (*C*(CH$_3$)$_2$), 60.0 (*C*H$_2$CH$_2$OTBDMS) 113.7 (CH-pyridone), 117.6 (CH-pyridone), 125.1 (C-pyridone), 152.2 (C-pyridone), 161.1 (C=O pyridone), 178.1 (C=O pyrrolidinone); HRMS calcd for $C_{18}H_{31}N_2O_3Si$ [M+H]$^+$ 351.2098, found 351.2100.

1-(2-Hydroxyethyl)-3,3,6-trimethyl-1,6-dihydro-2*H*-pyrrolo[2,3-*c*]pyridine-2,5(3*H*)-dione (14g)

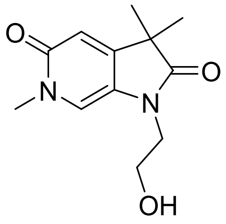

Compound **14f** (98 mg, 0.28 mmol) was dissolved in THF (2.5 mL). TBAF (1 M in THF, 2.5 mL) was added and the reaction mixture was stirred at r.t. for 18 h. The solvent was removed *in vacuo* and the residue was purified by MPLC on SiO_2 (MeOH:DCM, 0-20%) to give a white solid (56 mg, 90%). R_f = 0.30 (15% MeOH/DCM); m.p. 140-142°C; λ_{max} (EtOH)/nm 238.0, 258.6; IR ν_{max}/cm^{-1} 3271 (OH), 3043, 2973, 2932, 2870, 1708, (C=O), 1571 (C=O); ^{1}H NMR

(500 MHz, DMSO-d_6) δ_H 1.26 (6H, s, 2×C*H₃*), 3.39 (3H, s, C*H*₃), 3.57-3.60 (4H, m, C*H₂*), 4.82 (1H, t, *J* = 5.4 Hz, O*H*), 6.52 (1H, s, 1×pyridone-*H*), 7.44 (1H, s, 1×pyridone-*H*).; ^{13}C NMR (125 MHz, DMSO-d_6) δ_C 24.2 (2×*C*H₃), 37.3 (N-*C*H₃), 42.9 (CH_2CH_2N), 43.7 (*C*(CH_3)₂), 58.0 (CH_2*C*H_2O), 113.7 (CH-pyridone), 117.3 (CH-pyridone), 125.2 (C-pyridone), 152.4 (C-pyridone), 161.1 (C=O pyridone), 178.2 (C=O pyrrolidinone); HRMS calcd for $C_{12}H_{17}N_2O_3$ [M+H]$^+$ 237.1234, found 237.1232.

***tert*-Butyl 3,3-diethyl-5-methoxy-2-oxo-2,3-dihydro-1*H*-pyrrolo[2,3-c]pyridine-1-carboxylate (13)**

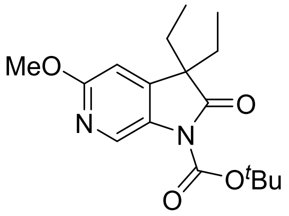

Compound **11** (200 mg, 0.76 mmol) and Cs_2CO_3 (517 mg, 1.59 mmol, 2.1 eq.) were suspended in MeCN (5 mL). EtI (0.152 mL, 1.89 mmol, 2.5 eq.) was added and the mixture was stirred at 60 ˚C for 3 h. The insoluble solids were removed by filtration and the filtrate was evaporated *in vacuo*. Purification by MPLC on SiO_2 (EtOAc:petrol, 0-20%) gave a clear gum (125 mg, 51%). R_f 0.4 (5% MeOH/DCM); ^{1}H NMR (500 MHz, $CDCl_3$) δ_H 0.66 (6H, t, *J* = 7.4 Hz, 2 × C*H*₃CH₂), 1.65 (9H, s, tBu), 1.72-1.81 (2H, m, CH_2CH_3), 1.94-2.03 (2H, m, CH_2CH_3), 3.96 (3H, s, OMe), 6.56 (1H, d, *J* = 0.7 Hz, H-Ar), 8.54 (1H, d, *J* = 0.7 Hz, H-Ar); MS (ES+) 207.2 [M+H]$^+$.

1,3,3,6-Tetramethyl-1,6-dihydro-2*H*-pyrrolo[2,3-c]pyridine-2,5(3*H*)-dione (17a)

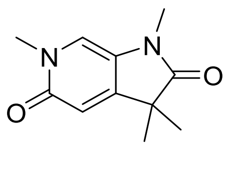

Prepared according to general procedure A1 using **7** (40 mg, 0.21 mmol), THF (1 mL), DMF (2 mL), NaH (8 mg, 80% dispersion in oil, 0.27 mmol) and MeI (17 µL, 0.27 mmol). Further NaH (8 mg, 80% dispersion in oil, 0.27 mmol) in THF (1 mL), and MeI (17 µL, 0.27 mmol) were added and the mixture was stirred at r.t. for 18 h. Purification by MPLC on silica (0-5% MeOH/DCM) gave the product as a white solid (28 mg, 65%); R_f 0.25 (5% MeOH/DCM); m.p. 55-60 ºC; IR ν_{max}/cm^{-1} 3357, 3245, 1695 (C=O), 1589 (C=O); ^{1}H NMR (500 MHz; CD_3OD)

δ_H 1.38 (6H, s, 2 Me), 3.16 (3H, s, NMe), 3.59 (3H, s, NMe), 6.59 (1H, s, H-Ar), 7.37 (1H, s, H-Ar); ^{13}C NMR (125 MHz; CD_3OD) δ_C 22.6, 25.4, 36.8, 44.2, 112.9, 116.4, 127.3, 153.2, 162.7, 179.1; HRMS calcd for $C_{11}H_{15}N_2O_2$ $[M+H]^+$ 207.1126, found 207.1124.

6-Allyl-3,3-dimethyl-1*H*-pyrrolo[2,3-c]pyridine-2,5(3*H*,6*H*)-dione (23)

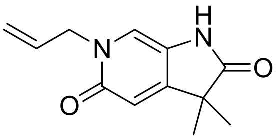

Compound **12** (100 mg, 0.34 mmol), allyl bromide (59 µL, 0.68 mmol, 2 eq.), NaI (103 mg, 0.68 mmol, 2 eq.) and MeCN (2 mL) were heated at 170 °C in a sealed vessel for 1 h under microwave irradiation. The solvent was removed *in vacuo*. Purification by MPLC on silica (0-10% MeOH/DCM) gave the target compound as a yellow solid (36 mg, 48%); R_f 0.25 (5% MeOH/DCM); ^{1}H NMR (500 MHz; $CDCl_3$) δ_H 1.40 (6H, s, CMe_2), 4.54 (2H, app dt, J = 6.0 and 1.4 Hz, NCH_2), 5.20-5.31 (2H, m,), 5.89-5.99 (1H, m, CH-alkene), 6.52 (1H, s, H-pyridone), 6.85 (1H, d, J = 0.3 Hz, H-pyridone), 7.86 (1H, br s, NH); ^{13}C NMR (125 MHz; $CDCl_3$) δ_C 24.0 ($C(CH_3)_2$), 44.4, 51.1, 114.4, 115.4, 118.8, 122.9, 132.6, 153.1, 180.7 (CO lactam);

1,3,3-Trimethyl-6-(prop-1-en-1-yl)-1*H*-pyrrolo[2,3-c]pyridine-2,5(3*H*,6*H*)-dione (24)

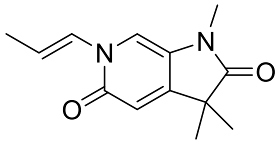

NaH (6 mg, 80% in oil, 0.206 mmol, 1.5 eq.) was added to a solution of **23** (30 mg, 0.137 mmol, 1 eq.) in DMF (1 mL) at r.t. and stirred for 15 min. MeI (13 µL, 0.206 mmol, 1.5 eq.) was added and the reaction was stirred at r.t. for 1 h. The mixture was quenched with MeOH and the solvent removed *in vacuo*. Purification by MPLC on silica (0-15% MeOH/DCM), followed by a second purification by MPLC on silica (70-100% EtOAc/petrol) gave the title compound (14 mg, 44%); ^{1}H NMR (500 MHz; $CDCl_3$) δ_H 1.37 (6H, s, CMe_2), 1.88 (3H, dd, J = 1.8 and 6.8 Hz, $C{=}CHCH_3$), 3.13 (3H, s, NMe), 5.72-5.82 (1H, m, $C{=}CHCH_3$), 6.49 (1H, s, H-pyridone), 6.87 (1H, s, H-pyridone), 7.25-7.31 (1H, m, $CH{=}CHCH_3$); ^{13}C NMR (125 MHz; $CDCl_3$) δ_C 15.4, 24.0, 26.5, 43.9, 109.6, 115.2, 116.7, 127.0, 127.7, 152.3, 160.3, 178.2; MS ES+ 233.2 $[M+H]^+$

1,3,3-Trimethyl-6-propyl-1*H*-pyrrolo[2,3-c]pyridine-2,5(3*H*,6*H*)-dione (17b)

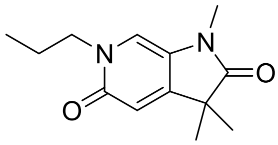

Compound **24** (14 mg, 0.060 mmol) was dissolved in MeOH (5 mL) and hydrogenated on a Thales H-cube at 40 °C/Full H_2 mode with constant recycling of reaction mixture through a 10% Pd/C Catcart™ for 2 h. The solvent was removed *in vacuo* to give the title compound as an off-white solid (9 mg, 64%); R_f 0.45 (5% MeOH/DCM); IR ν_{max}/cm^{-1} 3035, 2969, 2929, 1717 (C=O), 1586 (C=O); ^1H NMR (500 MHz; $CDCl_3$) δ_H 0.99 (3H, t, J = 7.4 Hz, $NCH_2CH_2CH_3$), 1.36 (6H, s, CMe_2), 1.74-1.84 (2H, m, $NCH_2CH_2CH_3$), 3.11 (3H, s, NMe), 3.85-3.92 (2H, m, $NCH_2CH_2CH_3$), 6.50 (1H, s, H-pyridone), 6.66 (1H, s, H-pyridone); ^{13}C NMR (125 MHz; $CDCl_3$) δ_C 11.2 (CH_2CH_3), 22.6, 24.0 ($C(CH_3)_2$), 26.4, 44.0 (NCH_2), 51.5 ($C(Me)_2$), 113.3, 115.2, 126.4, 151.8, 161.5, 178.4 (C=O lactam); MS ES+ 235.2 $[M+H]^+$

6-Benzyl-3,3-dimethyl-1*H*-pyrrolo[2,3-c]pyridine-2,5(3*H*,6*H*)-dione (15c)

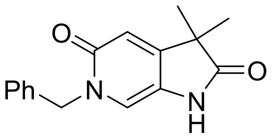

Prepared according to general procedure B using **12** (110 mg, 0.38 mmol), benzyl bromide (89 µL, 0.76 mmol, 2 eq.), NaI (113 mg, 0.76 mmol, 2 eq.) and MeCN (2.5 mL). The reaction was partitioned between water (20 mL) and EtOAc (3 x 30 mL) with addition of brine to aid separation. The organic extracts were combined, dried ($MgSO_4$) and the solvent was removed *in vacuo*. Purification by MPLC on silica (50-100% EtOAc/petrol) gave the target compound as a brown solid (97 mg, 96%). 50 mg of this material was further purified by MPLC on silica (0-5% MeOH/DCM) to give the target compound as a white solid (26 mg); R_f 0.2 (5% MeOH/DCM); m.p. 213 ºC dec.; IR ν_{max}/cm^{-1} 3019, 2734, 1708 (C=O), 1545 (C=O); ^1H NMR (500 MHz; $CDCl_3$) δ_H 1.38 (6H, s, $C(CH_3)_2$), 5.10 (2H, s, NCH_2Ph), 6.54 (1H, s, H-pyridone), 6.85 (1H, s, H-pyridone), 7.28-7.38 (5H, m, H-phenyl), 8.69 (1H, s, NH); ^{13}C NMR (125 MHz; $CDCl_3$) δ_C 24.0, 44.5, 51.9, 114.8, 115.4, 123.2, 128.2, 129.0, 136.4, 153.2, 161.4, 181.3; HRMS calcd for $C_{16}H_{17}N_2O_2$ $[M+H]^+$ 269.1285, found 269.1284.

6-Benzyl-1,3,3-trimethyl-1*H*-pyrrolo[2,3-c]pyridine-2,5(3*H*,6*H*)-dione (17c)

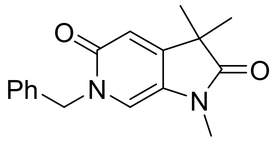

Prepared according to general procedure A1 using **15c** (45 mg, 0.17 mmol), NaH (10 mg, 80% disp. in oil, 0.34 mmol, 2 eq.), DMF (2 mL) and MeI (21 µL, 0.34 mmol, 2 eq.). Purification by MPLC on silica (50-100% EtOAc/petrol) gave the title compound as a white solid (30 mg, 64%); R_f 0.2 (EtOAc); m.p. 156-158 ºC; IR ν_{max}/cm^{-1} 3036, 1712 (C=O), 1590 (C=O); ^{1}H NMR (500 MHz; $CDCl_3$) δ_H 1.37 (6H, s, C(CH_3)$_2$), 3.04 (3H, s, N-Me), 5.14 (2H, s, NCH_2Ph), 6.55 (1H, s, H-pyridone), 6.64 (1H, s, H-pyridone), 7.29-7.39 (5H, m, H-phenyl); ^{13}C NMR (125 MHz; $CDCl_3$) δ_C 24.1 (Me), 26.4 (N-Me), 44.0 (N*C*H$_2$Ph), 52.0 (*C*(Me)$_2$), 112.7, 115.3, 126.7, 128.2, 129.0, 136.5, 152.2, 161.6 (CO pyridine), 178.3 (CO amide); HRMS calcd for $C_{17}H_{19}N_2O_2$ [M+H]$^+$ 283.1441, found 283.1435.

3,3-Dimethyl-6-phenethyl-1*H*-pyrrolo[2,3-c]pyridine-2,5(3*H*,6*H*)-dione (15d)

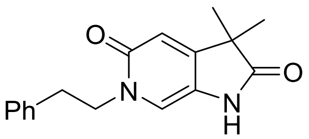

Prepared according to general procedure B using **12** (110 mg, 0.38 mmol), 2-bromoethylbenzene (102 µL, 0.76 mmol, 2 eq.), NaI (113 mg, 0.76 mmol, 2 eq.) and MeCN (2 mL) with heating at 170 °C in a sealed vessel for 1.5 h under microwave irradiation. The reaction was partitioned between water (20 mL) and EtOAc (4 × 20 mL) with addition of brine to aid separation. The aqueous layer was further extracted with DCM (4 × 20 mL). The organic extracts were combined, dried ($MgSO_4$) and the solvent was removed *in vacuo*. Purification by MPLC on silica (0-6% MeOH/DCM) gave the target compound as a yellow solid (21 mg, 20%); R_f 0.35 (5% MeOH/DCM); m.p. 210 ºC dec.; IR ν_{max}/cm^{-1} 3418, 2960, 1704 (C=O), 1540 (C=O); ^{1}H NMR (500 MHz; $CDCl_3$) δ_H 1.39 (6H, s, C(CH_3)$_2$), 3.04 (2H, t, J = 7.4 Hz, CH_2Ph), 4.09 (2H, t, J = 7.4 Hz, CH_2Ph), 6.52 (1H, s, H-pyridone), 6.60 (1H, s, H-pyridone), 7.16-7.33 (5H, m, H-phenyl), 7.98 (1H, s, NH); ^{13}C NMR (125 MHz; $CDCl_3$) δ_C 24.0, 35.2, 44.4, 52.0, 115.3, 115.4, 122.5, 126.8, 128.7, 128.9, 137.9, 153.1, 161.2, 180.9; HRMS calcd for $C_{17}H_{19}N_2O_2$ [M+H]$^+$ 283.1441, found 283.1436.

1,3,3-Trimethyl-6-phenethyl-1*H*-pyrrolo[2,3-c]pyridine-2,5(3*H*,6*H*)-dione (17d)

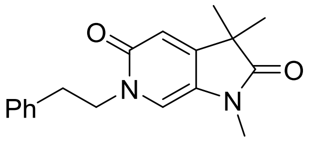

Prepared according to general procedure A1 using **15d** (26 mg, 0.092 mmol, 1 eq.), NaH (4 mg, 80% in oil, 0.138 mmol, 1.5 eq.), DMF (1 mL) and MeI (8 µL, 0.138 mmol, 1.5 eq.) The reaction was quenched with methanol, and the solvent was removed *in vacuo*. Purification by MPLC on silica (0-7% MeOH/DCM) gave the title compound as a white solid (19 mg, 70%); R_f 0.40 (5% MeOH/DCM); m.p. 141-145 ºC; IR ν_{max}/cm^{-1} 3040, 2923, 1714 (C=O), 1586 (C=O); ^{1}H NMR (500 MHz; $CDCl_3$) δ_H 1.35 (6H, s, CMe_2), 2.93 (3H, s, NMe), 3.06 (2H, t, *J* = 7.1 Hz, CH_2), 4.13 (2H, t, *J* = 7.1 Hz, CH_2), 6.26 (1H, s, H-pyridone), 6.51 (1H, s, H-pyridone), 7.16-7.20 (2H, m, H-Ar), 7.21-7.31 (3H, m, H-Ar); ^{13}C NMR (125 MHz; $CDCl_3$) δ_C 24.0 (C($C$$H_3$)$_2$), 26.2, 35.2, 44.1, 52.0, 113.8, 114.9, 126.1, 126.8, 128.7, 129.0, 138.0, 152.2, 161.4, 178.3; HRMS calcd for $C_{18}H_{21}N_2O_2$ $[M+H]^+$ 297.1598, found 297.1590.

Methyl 4-((3,3-dimethyl-2,5-dioxo-2,3-dihydro-1*H*-pyrrolo[2,3-c]pyridin-6(5*H*)-yl)methyl)benzoate (15e)

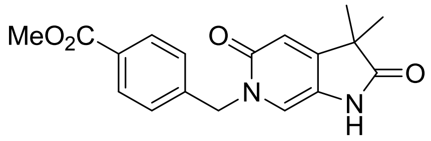

Prepared according to general procedure B using **12** (100 mg, 0.34 mmol, 1 eq.), NaI (102 mg, 0.68 mmol, 2 eq.), methyl-4-bromobenzoate (157 mg, 0.68 mmol, 2 eq.) and MeCN (2 mL) and heated to 170 °C for 1.5 h under microwave irradiation. The solvent was removed *in vacuo* and the residue was purified by MPLC on silica (0-10% MeOH/DCM) to give a yellow solid (70 mg, 63%); R_f 0.3 (5% MeOH/DCM); ^{1}H NMR (500 MHz; $CDCl_3$) δ_H 1.40 (6H, s, CMe_2), 3.91 (3H, s, CO_2Me), 5.15 (2H, s, CH_2N), 6.55 (1H, s, H-pyridone), 6.81 6.55 (1H, s, H-pyridone), 7.37 (1H, d, *J* = 8.5 Hz, 2 × H-phenyl), 7.63 (1H, br s, NH), 8.02 (1H, d, *J* = 8.5 Hz, 2 × H-phenyl); ^{13}C NMR (125 MHz; $CDCl_3$) δ_C 24.0 (C($C$$H_3$)$_2$), 44.4 ($CH_2$), 51.8 ($C$($CH_3$)$_2$), 52.2 (OMe), 114.4, 115.6, 123.3. 128.0, 130.0, 130.2, 141.4, 153.5, 161.3 (C-O pyridone), 166.6 (C-O ester), 180.9 (C=O lactam); MS ES+ 327.3 $[M+H]^+$

Methyl 4-((1,3,3-trimethyl-2,5-dioxo-2,3-dihydro-1*H*-pyrrolo[2,3-c]pyridin-6(5*H*)-yl)methyl)benzoate (17e)

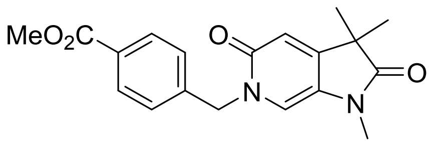

Prepared according to general procedure A1 using **15e** (60 mg, 0.18 mmol, 1 eq.), NaH (17 mg, 80% in oil, 0.54 mmol, 3 eq.), DMF (1 mL) and MeI (17 µL, 0.28 mmol, 1.5 eq.) The reaction quenched with MeOH and the solvent was removed *in vacuo*. Purification by MPLC on silica (0-8% MeOH/DCM) gave the title compound as an off-white solid (50 mg, 79%); R_f 0.4 (5% MeOH/DCM); IR ν_{max}/cm^{-1} 3028, 2966, 1706 (C=O), 1577 (C=O); ^{1}H NMR (500 MHz; $CDCl_3$) δ_H 1.38 (6H, s, CMe_2), 3.05 (3H, s, N-Me), 3.91 (3H, s, OMe), 5.18 (2H, s, N-CH_2), 6.56 (1H, s, H-pyridone), 6.62 (1H, s, H-pyridone), 7.38 (2H, d, *J* = 8.5 Hz, H-phenyl), 8.03 (2H, d, *J* = 8.5 Hz, H-phenyl); ^{13}C NMR (125 MHz; $CDCl_3$) δ_C 24.0 (C(CH$_3$)$_2$), 26.4 (NMe), 44.0 (CH_2), 51.9 (C(CH$_3$)$_2$), 52.2 (OMe), 112.5, 115.4, 126.9, 127.9, 130.0, 130.2, 141.5, 152.5, 161.5 (C-O pyridone), 166.6 (C-O ester), 178.2 (C-O pyrrolidinone); HRMS calcd for $C_{19}H_{21}N_2O_4$ $[M+H]^+$ 341.1496, found 341.1484.

4-((1,3,3-Trimethyl-2,5-dioxo-2,3-dihydro-1*H*-pyrrolo[2,3-c]pyridin-6(5*H*)-yl)methyl)benzoic acid (17f)

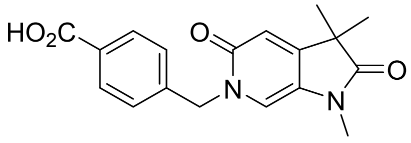

Compound **17e** (40 mg, 0.118 mmol), THF (0.5 mL) and NaOH (aq) (2 M, 0.5 mL) were combined and stirred at r.t. for 18 h. The mixture was partitioned between EtOAc (3 × 20 mL) and HCl (aq) (1 M). The organic extracts were combined, dried ($MgSO_4$) and the solvent was removed *in vacuo*. Purification by MPLC on silica (0-7% MeOH/DCM) gave the title compound as a white solid (33 mg, 87%); R_f 0.3 (95/5/1 DCM/MeOH/HCO_2H); IR ν_{max}/cm^{-1} 3059, 2970, 2925, 1717, 1695, 1583, 1538; ^{1}H NMR (500 MHz; DMSO-d_6) δ_H 1.31 (6H, s, CMe_2), 3.05 (3H, s, N-Me), 5.16 (2H, s, N-CH_2), 6.64 (1H, s, H-pyridone), 7.45 (2H, d, *J* = 8.4 Hz, H-phenyl), 7.57 (1H, s, H-pyridone), 7.94 (2H, d, *J* = 8.4 Hz, H-phenyl), 12.97 (1H, br s, CO_2H); ^{13}C NMR (125 MHz; DMSO-d_6) δ_C 24.0 (C(CH$_3$)$_2$), 26.8 (NMe), 43.9 (CH_2), 51.7 (C(CH$_3$)$_2$), 114.6, 115.9, 126.2, 128.2, 130.0, 142.9, 152.7, 160.9 (C-O pyridone), 167.6

(CO_2H), 177.9 (C-O pyrrolidinone); HRMS calcd for $C_{18}H_{19}N_2O_4$ $[M+H]^+$ 327.1339, found 327.1337.

4-((3,3-Dimethyl-2,5-dioxo-1,2,3,5-tetrahydro-6*H*-pyrrolo[2,3-c]pyridin-6-yl)methyl)benzonitrile (15g)

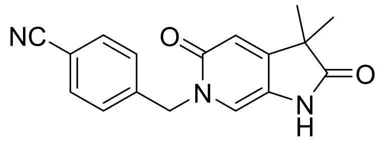

Prepared according to general procedure B using **12** (100 mg, 0.34 mmol, 1 eq.), NaI (102 mg, 0.68 mmol, 2 eq.), 4-(bromomethyl)benzonitrile (134 mg, 0.68 mmol, 2 eq.) and MeCN (2 mL) with heating to 170 °C for 1.5 h under microwave irradiation in a sealed vessel. The mixture was partitioned between EtOAc (3 × 20 mL) and water (10 mL), washed with brine, and the organic extracts were combined, dried ($MgSO_4$) and the solvent was removed *in vacuo*. Purification by MPLC on silica (0-7% MeOH/DCM) gave the title compound as a yellow solid (70 mg, 70%); R_f 0.25 (5% MeOH/DCM); ^{1}H NMR (500 MHz; DMSO-d_6) δ_H 1.29 (6H, s, CMe_2) 5.17 (2H, s, NCH_2), 6.60 (1H, s, CH-pyridone), 7.28 (1H, s, CH-pyridone), 7.48 (2H, d, J = 8.4 Hz, H-phenyl), 7.85 (2H, d, J = 8.4 Hz, H-phenyl), 10.31 (1H, s, NH); ^{13}C NMR (125 MHz; DMSO-d_6) δ_C 24.0 ($C(CH_3)_2$), 44.1 (CH_2), 51.3 ($C(CH_3)_2$), 110.6 (CN), 114.8, 115.9, 119.2; 124.1, 128.9, 132.9, 143.9, 160.7 (C-O pyridone), 180.2 (C-O pyrrolidinone); MS ES+ 294.2 $[M+H]^+$

4-((1,3,3-Trimethyl-2,5-dioxo-1,2,3,5-tetrahydro-6H-pyrrolo[2,3-c]pyridin-6-yl)methyl)benzonitrile (17g)

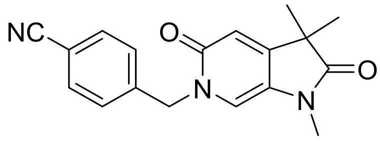

Prepared according to general procedure A using **15g** (60 mg, 0.20 mmol), Cs_2CO_3 (199 mg, 0.61 mmol, 3 eq.), MeI (25 µL, 0.41 mmol, 2 eq.) and DMF (3 mL). Purification by MPLC on silica (0-7% MeOH/DCM) gave the title compound as an off-white solid (37 mg, 59%); R_f 0.45 (5% MeOH/DCM); IR ν_{max}/cm^{-1} 2967, 2930, 2225 (CN), 1720 (C=O), 1594 (C=O); ^{1}H NMR (500 MHz; $CDCl_3$) δ_H 1.38 (6H, s, CMe_2), 3.07 (3H, s, NMe), 5.17 (2H, s, CH_2N), 6.56 (1H, s, H-pyridone), 6.63 (1H, s, H-pyridone), 7.42 (2H, d, J = 8.4 Hz, H-phenyl), 7.65 (2H, d, J =

8.4 Hz, H-phenyl); ^{13}C NMR (125 MHz; $CDCl_3$) δ_C 24.1 (C(CH$_3$)$_2$), 26.5 (NMe), 44.0 (CH_2), 52.1 (C(CH_3)$_2$), 112.1, 112.4, 115.5, 118.4, 127.1, 128.4, 132.7, 141.8, 152.8, 161.4 (C-O pyridone), 178.1 (C-O pyrrolidinone); HRMS calcd for $C_{18}H_{18}N_2O_3$ $[M+H]^+$ 308.1394, found 308.1383.

4-((1,3,3-Trimethyl-2,5-dioxo-1,2,3,5-tetrahydro-6*H*-pyrrolo[2,3-c]pyridin-6-yl)methyl)benzamide (17h)

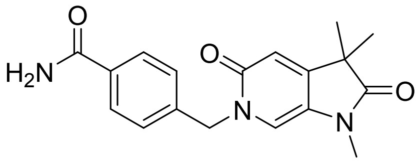

Compound **17g** (60 mg, 0.184 mmol, 1 eq.), CDI (60 mg, 0.368 mmol, 2 eq.) and THF (1 mL) were combined in a sealed vessel and heated to 80 °C for 2 h. The mixture was allowed to cool, NH_4OH (0.880, 100 µL) was added and the mixture was heated to 50 °C for 1 h, and then stirred at r.t. for 18 h. The solvent was removed *in vacuo*, and the crude material was purified by MPLC on silica (1-12% MeOH/DCM). Impure product thus obtained was partitioned between water and DCM (5 × 20 mL), the organic extracts were combined, dried over $MgSO_4$ and the solvent removed *in vacuo* to give the title compound as a white solid (18 mg, 30%); R_f 0.5 (5% MeOH/DCM); m.p. 206-209 ºC; IR ν_{max}/cm^{-1} 3534, 3405, 3126, 2973, 1698, 1672, 1562; ^1H NMR (500 MHz; $CDCl_3$) δ_H 1.38 (6H, s, 2 x Me), 3.06 (3H, s, N-Me pyrrolidinone), 5.18 (2H, s, CH_2), 5.67 (1H, br s, NH), 6.09 (1H, br s, NH), 6.56 (1H, s, H-pyridone), 6.65 (1H, s, H-pyridone), 7.39-7.42 (2H, m, 2 × H-phenyl), 7.78-7.83 (2H, m, 2 × H-phenyl); ^{13}C NMR (125 MHz; $CDCl_3$) δ_C 24.1 (C(CH$_3$)$_2$), 26.5 (NMe), 44.0 (CH_2), 51.9 (C(CH$_3$)$_2$), 112.6, 113.4, 115.4, 127.0, 128.0, 128.2, 133.1, 140.6, 152.6, 161.5 (C-O pyridone), 168.5 (C-O amide), 178.2 (C-O pyrrolidinone); HRMS calcd for $C_{18}H_{20}N_3O_3$ $[M+H]^+$ 326.1499, found 326.1494.

3,3-Dimethyl-6-(4-(methylsulfonyl)benzyl)-1,6-dihydro-2*H*-pyrrolo[2,3-*c*]pyridine-2,5(3*H*)-dione (15i)

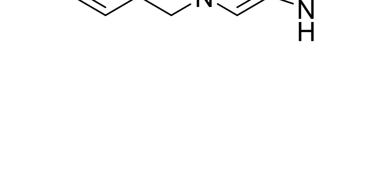

Prepared according to general procedure B using compound **12** (87 mg, 0.298 mmol), 1-(bromomethyl)-4-(methylsulfonyl)benzene (148 mg, 0.596 mmol) and MeCN (2.5 mL). Purification by MPLC on SiO_2 (0-7% MeOH/DCM) gave a white solid (55 mg, 53%). R_f 0.27 (5% MeOH/DCM); m.p. 320 °C (degraded); λ_{max} (EtOH)/nm 339.4, 255.6, 223.2; IR ν_{max}/cm^{-1} 3083, 3008, 2973, 2926, 2731, 1717 (C=O), 1549, 1306 (SO), 1146 (SO); ^{1}H NMR (500 MHz, DMSO-d_6) δ_H 1.26 (6H, s, 2×C*H₃*), 3.20 (1H, s, SO_2CH_3), 5.16 (2H, s, CH_2), 6.58 (1H, s, 1×pyridone-*H*), 7.28 (1H, s, 1×pyridone-*H*), 7.52-7.54 (2H, m, 2×Ar-*H*), 7.89-7.91 (2H, m, 2×Ar-*H*), 10.31 (1H, s, N*H*); ^{13}C NMR (125 MHz, DMSO-d_6) δ_C 24.0 (2×CH_3), 44.0 (SO_2CH_3), 44.1 ($C(CH_3)_2$), 51.2 (NCH_2Ar), 114.8 (CH-pyridone), 115.9 (CH-pyridone), 124.0 (C-Ar), 127.8 (2×C-Ar), 128.9 (2×C-Ar), 140.3 (C-Ar), 144.1 (C-Ar), 154.4 (C-Ar), 160.7 (C=O pyridone), 180.2 (C=O pyrrolidinone); HRMS calcd for $C_{17}H_{19}N_2O_4S$ $[M+H]^+$ 347.1060, found 347.1062.

1,3,3-Trimethyl-6-(4-(methylsulfonyl)benzyl)-1,6-dihydro-2*H*-pyrrolo[2,3-*c*]pyridine-2,5(3*H*)-dione (17i)

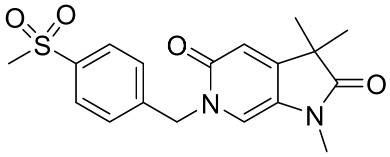

Prepared according to general procedure A using compound **15i** (36 mg, 0.104 mmol), Cs_2CO_3 (102 mg, 0.312 mmol), MeI (16 µL, 0.260 mmol) and DMF (1 mL). Purification by MPLC on SiO_2 (0-5% MeOH/DCM) gave a white solid (32 mg, 86%). R_f 0.35 (5% MeOH/DCM); m.p. 95-97 °C; λ_{max} (EtOH)/nm 338.8, 259.2, 224.6; IR ν_{max}/cm^{-1} 3050, 2969, 2927, 1709 (C=O), 1587 (C=O), 1300 (SO), 1145 (SO); ^{1}H NMR (500 MHz, CD_3OD) δ_H 1.27 (6H, s, 2×CH_3), 2.99 (1H, s, SO_2CH_3), 3.03 (1H, s, N-CH_3) 5.19 (2H, s, CH_2), 6.52 (1H, s, 1×Pyridone-*H*), 7.37 (1H, s, 1×Pyridone-*H*), 7.46-7.48 (2H, m, 2×Ar-*H*), 7.82-7.84 (2H, m, 2×Ar-*H*); ^{13}C NMR (125 MHz, CD_3OD) δ_C 22.6 (2×CH_3), 25.5 (N-CH_3), 42.9 (SO_2CH_3), 44.2 ($C(CH_3)_2$), 52.0 (NCH_2Ar), 113.7 (CH-pyridone), 115.3 (CH-pyridone), 127.5 (2×C-Ar), 127.7 (C-Ar), 128.3 (2×C-Ar), 140.1 (C-Ar), 143.1 (C-Ar), 153.9 (C-Ar), 162.1 (C=O pyridone), 179.0 (C=O pyrrolidinone); HRMS calcd for $C_{18}H_{21}N_2O_4S$ $[M+H]^+$ 361.1217, found 361.1215.

6-(4-Chlorobenzyl)-3,3-dimethyl-1,6-dihydro-2*H*-pyrrolo[2,3-*c*]pyridine-2,5(3*H*)-dione (15j)

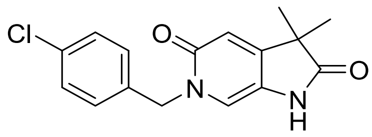

Prepared according to general procedure B using compound **12** (75 mg, 0.25 mmol), 4-chlorobenzyl bromide (105 mg, 0.51 mmol) and MeCN (2 mL). Purification by MPLC on SiO_2 (0-7% MeOH/DCM) gave a white solid (62 mg, 80%). R_f 0.30 (5% MeOH/DCM); m.p. 255-257 °C; λ_{max} (EtOH)/nm 338.8, 256.6, 221.4; IR ν_{max}/cm^{-1} 3071, 3022, 2967, 2930, 2864, 2734, 1711 (C=O), 1559 (C=O); ^{1}H NMR (500 MHz, DMSO-d_6) δ_H 1.24 (6H, s, 2×CH_3), 5.04 (2H, s, CH_2), 6.55 (1H, s, 1×pyridone-*H*), 7.22 (1H, d, J = 0.4 Hz, 1×pyridone-*H*), 7.32-7.34 (2H, m, 2×Ar-*H*), 7.40-7.42 (2H, m, 2×Ar-*H*), 10.27 (1H, s, N*H*); ^{13}C NMR (125 MHz, DMSO-d_6) δ_C 24.0 (2×*C*H$_3$), 44.0 (*C*(CH$_3$)$_2$), 50.7 (N*C*H$_2$Ar), 114.8 (CH-pyridone), 115.7 (CH-pyridone), 123.9 (C-Ar), 129.0 (2×C-Ar), 130.2 (2×C-Ar), 132.6 (C-Ar), 137.3 (C-Ar), 154.2 (C-Ar), 160.6 (C=O pyridone), 180.2 (C=O pyrrolidinone); HRMS calcd for $C_{16}H_{16}ClN_2O_2$ [M(^{35}Cl)+H]$^+$ 303.0895, found 303.0901.

6-(4-Chlorobenzyl)-1,3,3-trimethyl-1,6-dihydro-2*H*-pyrrolo[2,3-*c*]pyridine-2,5(3*H*)-dione (17j)

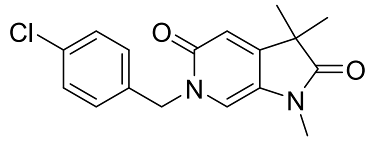

Prepared according to general procedure A using compound **15j** (34 mg, 0.11 mmol), Cs_2CO_3 (110 mg, 0.33 mmol), MeI (17 µL, 0.28 mmol) and DMF (1.2 mL). Purification by MPLC on SiO_2 (0-5% MeOH/DCM) gave a white solid (32 mg, 90%). R_f 0.37 (5% MeOH/DCM); m.p. 181-183 °C; λ_{max} (EtOH)/nm 338.4, 259.4, 221.2; IR ν_{max}/cm^{-1} 3061, 3018, 2973, 2927, 2864, 1713 (C=O), 1597 (C=O); ^{1}H NMR (500 MHz, CD_3OD) δ_H 1.26 (6H, s, 2×CH_3), 3.01 (1H, s, N-CH_3), 5.06 (2H, s, CH_2), 6.50 (1H, s, 1×pyridone-*H*), 7.24 (4H, s, 4×Ar-*H*) 7.31 (1H, s, 1×pyridone-*H*); ^{13}C NMR (125 MHz, CD_3OD) δ_C 22.6 (2×*C*H$_3$), 25.4 (N-*C*H$_3$), 44.1 (*C*(CH$_3$)$_2$), 51.6 (N*C*H$_2$Ar), 113.6 (CH-pyridone), 115.1 (CH-pyridone), 127.6 (C-Ar), 128.4 (2×C-Ar), 129.2 (2×C-Ar), 133.4 (C-Ar), 135.6 (C-Ar), 153.5 (C-Ar), 162.1 (C=O pyridone), 179.0 (C=O pyrrolidinone); HRMS calcd for $C_{17}H_{18}ClN_2O_2$ [M(^{35}Cl)+H]$^+$ 317.1051, found 317.1056.

6-(4-Bromobenzyl)-3,3-dimethyl-1,6-dihydro-2*H*-pyrrolo[2,3-*c*]pyridine-2,5(3*H*)-dione (15k)

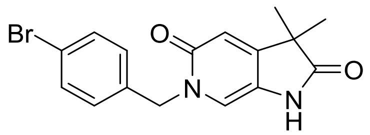

Prepared according to general procedure B using compound **12** (80 mg, 0.27 mmol), 1-bromo-4-(bromomethyl)benzene (135 mg, 0.54 mmol) and MeCN (2 mL). Purification by MPLC on SiO_2 (0-7% MeOH/DCM) gave a white solid (73 mg, 78%). R_f 0.32 (5% MeOH/DCM); m.p. 269-271 °C; λ_{max} (EtOH)/nm 339.4, 259.4, 221.6; IR ν_{max}/cm^{-1} 3025, 2965, 2928, 2860, 2734, 1705 (C=O), 1545 (C=O); ^{1}H NMR (500 MHz, DMSO-d_6) δ_H 1.24 (6H, s, 2×C*H₃*), 5.02 (2H, s, C*H₂*), 6.55 (1H, s, 1×pyridone-*H*), 7.22 (1H, s, 1×pyridone-*H*), 7.26-7.28 (2H, m, 2×Ar-*H*), 7.53-7.55 (2H, m, 2×Ar-*H*), 10.27 (1H, s, N*H*); ^{13}C NMR (125 MHz, DMSO-d_6) δ_C 24.0 (2×*C*H_3), 44.0 (*C*$(CH_3)_2$), 50.7 (N*C*H_2Ar), 114.8 (CH-pyridone), 115.7 (CH-pyridone), 121.1 (C-Ar), 123.9 (C-Ar), 130.5 (2×C-Ar), 131.9 (2×C-Ar), 137.7 (C-Ar), 154.2 (C-Ar), 160.6 (C=O pyridone), 180.2 (C=O pyrrolidinone); HRMS calcd for $C_{16}H_{16}BrN_2O_2$ [M(^{79}Br)+H]$^+$ 347.0390, found 347.0390.

6-(4-Bromobenzyl)-1,3,3-trimethyl-1,6-dihydro-2*H*-pyrrolo[2,3-*c*]pyridine-2,5(3*H*)-dione (17k)

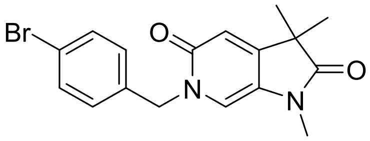

Prepared according to general procedure A using compound **15k** (50 mg, 0.14 mmol), Cs_2CO_3 (137 mg, 0.42 mmol), MeI (22 µL, 0.35 mmol) and DMF (1.4 mL). Purification by MPLC on SiO_2 (0-5% MeOH/DCM) gave a white solid (48 mg, 95%). R_f 0.40 (5% MeOH/DCM); m.p. 198-200 °C; λ_{max} (EtOH)/nm 338.2, 259.4, 222.4; IR ν_{max}/cm^{-1} 3063, 3019, 2977, 2933, 2870, 1715 (C=O), 1598 (C=O); ^{1}H NMR (500 MHz, CD_3OD) δ_H 1.26 (6H, s, 2×C*H*$_3$), 3.01 (1H, s, N-C*H*$_3$), 5.04 (2H, s, C*H₂*), 6.50 (1H, s, 1×Pyridone-*H*), 7.16-7.18 (2H, m, 2×Ar-*H*), 7.31 (1H, s, 1×pyridone-*H*), 7.38-7.40 (2H, m, 2×Ar-*H*); ^{13}C NMR (125 MHz, CD_3OD) δ_C 22.6 (2×*C*H_3), 25.4 (N-*C*H_3), 44.1 (*C*$(CH_3)_2$), 51.7 (N*C*H_2Ar), 113.6 (CH-pyridone), 115.1 (CH-pyridone), 121.3 (C-Ar), 127.6 (C-Ar), 129.5 (2×C-Ar), 131.4 (2×C-Ar), 136.0 (C-Ar), 153.5 (C-Ar),

162.1 (C=O pyridone), 179.0 (C=O pyrrolidinone); HRMS calcd for $C_{17}H_{17}BrN_2O_2$ $[M(^{79}Br)+H]^+$ 361.0546, found 361.0544.

3,3-Dimethyl-6-(4-methylbenzyl)-1,6-dihydro-2*H*-pyrrolo[2,3-*c*]pyridine-2,5(3*H*)-dione (15l)

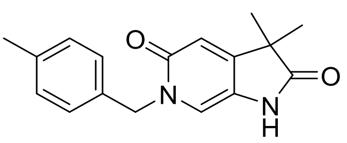

Prepared according to general procedure B using compound **12** (80 mg, 0.27 mmol), 1-(bromomethyl)-4-methylbenzene (75 mg, 0.54 mmol) and MeCN (2.7 mL). Purification by MPLC on SiO_2 (0-7% MeOH/DCM) gave a white solid (66 mg, 85%). R_f 0.30 (5% MeOH/DCM); m.p. 252-254 °C; λ_{max} (EtOH)/nm 339.4, 256.8, 217.6; IR ν_{max}/cm^{-1} 3021, 2968, 2922, 2863, 2736, 1719 (C=O), 1586 (C=O); ^{1}H NMR (500 MHz, DMSO-*d*$_6$) δ_H 1.24 (6H, s, 2×C*H$_3$*), 2.27 (3H, s, C*H*$_3$-Ar), 5.00 (2H, s, C*H$_2$*), 6.53 (1H, s, 1×pyridone-*H*), 7.12-7.15 (2H, m, 2×Ar-*H* + 1×pyridone-*H*), 7.20-7.22 (2H, m, 2×Ar-*H*), 10.18 (1H, s, N*H*); ^{13}C NMR (125 MHz, DMSO-*d*$_6$) δ_C 21.2 (*C*H$_3$-Ar), 24.0 (2×*C*H$_3$), 44.0 (*C*(CH$_3$)$_2$), 50.8 (N*C*H$_2$Ar), 114.7 (CH-pyridone), 115.6 (CH-pyridone), 123.7 (C-Ar), 128.4 (2×C-Ar), 129.6 (2×C-Ar), 135.3 (C-Ar), 137.2 (C-Ar), 153.9 (C-Ar), 160.6 (C=O pyridone), 180.2 (C=O pyrrolidinone); HRMS calcd for $C_{17}H_{19}N_2O_2$ $[M+H]^+$ 283.1441, found 283.1440.

1,3,3-Trimethyl-6-(4-methylbenzyl)-1,6-dihydro-2*H*-pyrrolo[2,3-*c*]pyridine-2,5(3*H*)-dione (17l)

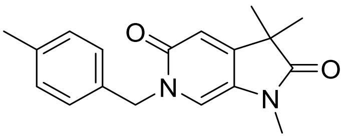

Prepared according to general procedure A using compound **15l** (50 mg, 0.18 mmol), Cs_2CO_3 (173 mg, 0.53 mmol), MeI (22 µL, 0.35 mmol) and DMF (1.7 mL). Purification by MPLC on SiO_2 (0-5% MeOH/DCM) gave a white solid (48 mg, 92%). R_f 0.42 (5% MeOH/DCM); m.p. 180-182 °C; λ_{max} (EtOH)/nm 337.8, 256.0, 218.2; IR ν_{max}/cm^{-1} 3056, 2973, 2928, 2865, 1713 (C=O), 1601 (C=O); ^{1}H NMR (500 MHz, CD_3OD) δ_H 1.25 (6H, s, 2×C*H$_3$*), 2.20 (3H, s, C*H*$_3$-Ar), 3.00 (1H, s, N-C*H*$_3$), 5.04 (2H, s, C*H$_2$*), 6.49 (1H, d, *J* = 0.3 Hz, 1×pyridone-*H*), 7.04-7.06 (2H, m, 2×Ar-*H*), 7.12-7.14 (2H, m, 2×Ar-*H*), 7.24 (1H, d, *J* = 0.3 Hz, 1×pyridone-*H*); ^{13}C NMR (125 MHz, CD_3OD) δ_C 21.1 (*C*H$_3$-Ar), 24.1 (2×*C*H$_3$), 26.8 (N-*C*H$_3$), 45.5 (*C*(CH$_3$)$_2$),

53.3 (N*C*H_2Ar), 115.0 (CH-pyridone), 116.6 (CH-pyridone), 128.9 (C-Ar), 129.0 (2×C-Ar), 130.4 (2×C-Ar), 135.2 (C-Ar), 138.9 (C-Ar), 154.7 (C-Ar), 163.6 (C=O pyridone), 180.5 (C=O pyrrolidinone); HRMS calcd for $C_{18}H_{21}N_2O_2$ $[M+H]^+$ 297.1598, found 297.1596.

3-Dimethyl-6-(4-(trifluoromethyl)benzyl)-1,6-dihydro-2*H*-pyrrolo[2,3-*c*]pyridine-2,5(3*H*)-dione (15m)

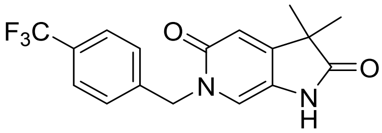

Prepared according to general procedure B using compound **12** (80 mg, 0.27 mmol), 1-(bromomethyl)-4-(trifluoromethyl)benzene (129 mg, 0.54 mmol) and MeCN (2 mL). Purification by MPLC on SiO_2 (0-7% MeOH/DCM) gave a white solid (67 mg, 71%). R_f 0.30 (5% MeOH/DCM); m.p. 275-277 °C; λ_{max} (EtOH)/nm 338.0, 257.0, 217.4; IR ν_{max}/cm^{-1} 3073, 3008, 2973, 2932, 2867, 2735, 1716 (C=O), 1562 (C=O); ^{1}H NMR (500 MHz, DMSO-d_6) δ_H 1.25 (6H, s, 2×C*H_3*), 5.13 (2H, s, C*H_2*), 6.57 (1H, s, 1×pyridone-*H*), 7.26 (1H, s, 1×pyridone-*H*), 7.49 (2H, d, *J* = 8.1 Hz, 2×Ar-*H*), 7.72 (2H, d, *J* = 8.1 Hz, 2×Ar-*H*); ^{13}C NMR (125 MHz, DMSO-d_6) δ_C 24.0 (2×*C*H_3), 44.1 (*C*(CH_3)$_2$), 51.1 (N*C*H_2Ar), 114.8 (CH-pyridone), 115.9 (CH-pyridone), 124.0 (C-Ar), 124.7 (CF_3, q, *J* = 127.9 Hz), 125.9 (2×CF_3C*C*H-Ar, q, *J* = 3.6 Hz), 128.4 (CF_3*C*-Ar, q, *J* = 31.9 Hz), 128.8 (2×CF_3CCH*C*H-Ar), 143.0 (2×C-Ar), 154.4 (C-Ar), 160.7 (C=O pyridone), 180.2 (C=O pyrrolidinone); ^{19}F NMR (470 MHz, DMSO-d_6) δ -60.92 (3F, s, C*F_3*); HRMS calcd for $C_{17}H_{16}F_3N_2O_2$ $[M+H]^+$ 337.1158, found 337.1155.

1,3,3-Trimethyl-6-(4-(trifluoromethyl)benzyl)-1,6-dihydro-2*H*-pyrrolo[2,3-*c*]pyridine-2,5(3*H*)-dione (17m)

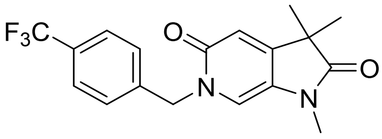

Prepared according to general procedure A using compound **15m** (42 mg, 0.13 mmol), Cs_2CO_3 (122 mg, 0.38 mmol), MeI (20 μL, 0.31 mmol) and DMF (1.2 mL). Purification by MPLC on SiO_2 (0-5% MeOH/DCM) gave a white solid (40 mg, 92%). R_f 0.40 (5% MeOH/DCM); m.p. 172-174 °C; λ_{max} (EtOH)/nm 338.8, 258.8, 218.8; IR ν_{max}/cm^{-1} 3035, 2970, 2928, 2865, 1706 (C=O), 1580 (C=O); ^{1}H NMR (500 MHz, CD_3OD) δ_H 1.27 (6H, s, 2×C*H_3*), 3.02 (1H, s, N-C*H_3*), 5.16 (2H, s, C*H_2*), 6.52 (1H, s, 1×pyridone-*H*), 7.33 (1H, s, 1×pyridone-*H*), 7.41 (2H, d,

J = 8.1 Hz, 2×Ar-H), 7.54 (2H, d, J = 8.1 Hz, 2×Ar-H); ^{13}C NMR (125 MHz, CD_3OD) δ_C 22.6 (2×CH_3), 25.4 (N-CH_3), 44.1 ($C(CH_3)_2$), 51.9 (NCH_2Ar), 113.7 (CH-pyridone), 115.2 (CH-pyridone), 125.2 (2×CF_3CCH-Ar, q, J = 3.6 Hz), 127.6 (C-Ar), 127.9 (2×CF_3CCHCH-Ar), 129.6 (CF_3C-Ar, q, J = 32.2 Hz), 141.2 (C-Ar), 153.7 (C-Ar), 162.2 (C=O pyridone), 179.0 (C=O pyrrolidinone), CF_3 quartet was not observed; ^{19}F NMR (470 MHz, CD_3OD) δ -64.10 (3F, s, CF_3); HRMS calcd for $C_{18}H_{18}F_3N_2O_2$ $[M+H]^+$ 551.1310, found 551.1297.

6-(3,4-Dichlorobenzyl)-3,3-dimethyl-1,6-dihydro-2*H*-pyrrolo[2,3-*c*]pyridine-2,5(3*H*)-dione (15n)

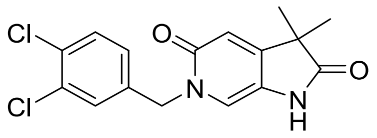

Prepared according to general procedure B using compound **12** (80 mg, 0.27 mmol), 4-(bromomethyl)-1,2-dichlorobenzene (132 mg, 0.54 mmol) and MeCN (2 mL). Purification by MPLC on SiO_2 (0-7% MeOH/DCM) gave a white solid (85 mg, 93%). R_f 0.39 (5% MeOH/DCM); m.p. 248-250 °C; λ_{max} (EtOH)/nm 339.0, 256.8, 220.0; IR ν_{max}/cm^{-1} 2961, 2870, 2789, 2729, 1703 (C=O), 1546 (C=O); ^{1}H NMR (500 MHz, DMSO-d_6) δ_H 1.25 (6H, s, 2×CH_3), 5.04 (2H, s, CH_2), 6.55 (1H, s, 1×pyridone-H), 7.27 (1H, s, 1×pyridone-H), 7.30 (1H, dd, J = 8.2 and 2.0 Hz, 1×Ar-H), 7.60-7.63 (2H, m, 2×Ar-H), 10.27 (1H, s, NH); ^{13}C NMR (125 MHz, DMSO-d_6) δ_C 24.0 (2×CH_3), 44.0 ($C(CH_3)_2$), 50.4 (NCH_2Ar), 114.8 (CH-pyridone), 115.7 (CH-pyridone), 124.1 (C-Ar), 128.8 (C-Ar), 130.5 (C-Ar), 131.3 (C-Ar), 131.4 (C-Ar), 139.4 (2×C-Ar), 154.4 (C-Ar), 160.6 (C=O pyridone), 180.2 (C=O pyrrolidinone); HRMS calcd for $C_{16}H_{15}Cl_2N_2O_2$ $[M(^{35}Cl_2)+H]^+$ 337.0505, found 337.0505.

6-(3,4-Dichlorobenzyl)-1,3,3-trimethyl-1,6-dihydro-2*H*-pyrrolo[2,3-*c*]pyridine-2,5(3*H*)-dione (17n)

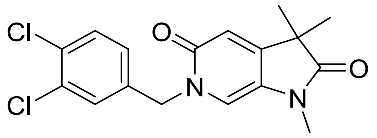

Prepared according to general procedure A using compound **15n** (50 mg, 0.15 mmol), Cs_2CO_3 (145 mg, 0.44 mmol), MeI (23 μL, 0.37 mmol) and DMF (1.5 mL). Purification by MPLC on SiO_2 (0-5% MeOH/DCM) gave a white solid (49 mg, 94%). R_f 0.41 (5% MeOH/DCM); m.p. 151-153 °C; λ_{max} (EtOH)/nm 340.2, 259.6, 220.6; IR ν_{max}/cm^{-1} 3063, 3022, 2974, 2929, 2866,

1712 (C=O), 1595 (C=O); ^{1}H NMR (500 MHz, CD_3OD) δ_H 1.26 (6H, s, 2×C*H*$_3$), 3.02 (1H, s, N-C*H*$_3$), 5.04 (2H, s, C*H*$_2$), 6.50 (1H, s, 1×pyridone-*H*), 7.18 (1H, dd, *J* = 8.3 and 2.0 Hz, Ar-*H*), 7.33 (1H, s, 1×pyridone-*H*), 7.39 (1H, d, *J* = 8.3 Hz, Ar-*H*), 7.44 (1H, d, *J* = 2.0 Hz, Ar-*H*); ^{13}C NMR (125 MHz, CD_3OD) δ_C 24.0 (2×CH_3), 26.9 (N-*C*H_3), 45.5 (*C*(CH_3)$_2$), 52.7 (N*C*H_2Ar), 115.1 (CH-pyridone), 116.5 (CH-pyridone), 128.9 (C-Ar), 129.1 (C-Ar), 131.1 (C-Ar), 131.8 (C-Ar), 132.8 (C-Ar), 133.5 (C-Ar), 138.9 (C-Ar), 155.1 (C-Ar), 163.5 (C=O pyridone), 180.4 (C=O pyrrolidinone); HRMS calcd for $C_{17}H_{17}Cl_2N_2O_2$ [M($^{35}Cl_2$)+H]$^+$ 351.0662, found 351.0661.

2-((3,3-Dimethyl-2,5-dioxo-1,2,3,5-tetrahydro-6*H*-pyrrolo[2,3-*c*]pyridin-6-yl)methyl)benzonitrile (15o)

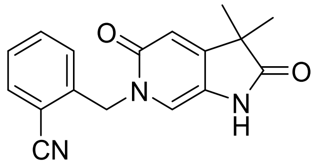

Prepared according to general procedure B using compound **12** (120 mg, 0.411 mmol), 2-(bromomethyl)benzonitrile (162 mg, 0.824 mmol) and MeCN (3.8 mL). Purification by MPLC on SiO_2 (0-7% MeOH/DCM) gave a white solid (75 mg, 63%). R_f 0.32 (5% MeOH/DCM); m.p. 315 °C (degraded); λ_{max} (EtOH)/nm 339.2, 224.0; IR ν_{max}/cm^{-1} 3069, 3019, 2972, 2726, 2224 (CN), 1708 (C=O), 1588 (C=O); ^{1}H NMR (500 MHz, DMSO-*d*$_6$) δ_H 1.27 (6H, s, 2×C*H*$_3$), 5.24 (2H, s, C*H*$_2$), 6.58 (1H, s, 1×pyridone-*H*), 7.12 (1H, d, *J* = 7.8 Hz, Ar*H*), 7.27 (1H, s, 1×pyridone-*H*), 7.48 (1H, t, *J* = 7.6 Hz, Ar*H*), 7.67 (1H, td, *J* = 7.8 and 1.1 Hz, Ar*H*), 7.88 (1H, dd, *J* = 7.7 and 1.1 Hz, Ar*H*), 10.33 (1H, s, N*H*); ^{13}C NMR (125 MHz, DMSO-*d*$_6$) δ_C 24.0 (2×*C*H_3), 44.1 (*C*(CH_3)$_2$), 50.4 (N*C*H_2Ar), 110.9 (C-Ar), 114.8 (CH-pyridone), 116.2 (CH-pyridone), 117.7 (*C*N), 124.1 (C-Ar), 127.8 (C-Ar), 128.5 (C-Ar), 133.5 (C-Ar), 134.1 (C-Ar), 141.5 (C-Ar), 154.7 (C-Ar), 160.7 (C=O pyridone), 180.1 (C=O pyrrolidinone); HRMS calcd for $C_{17}H_{16}N_3O_2$ [M+H]$^+$ 294.1237, found 224.1240.

2-((1,3,3-Trimethyl-2,5-dioxo-1,2,3,5-tetrahydro-6*H*-pyrrolo[2,3-*c*]pyridin-6-yl)methyl)benzonitrile (17o)

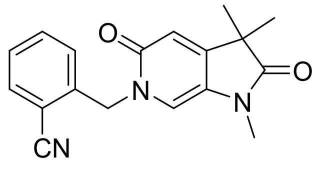

Prepared according to general procedure A using compound **15o** (50 mg, 0.17 mmol), Cs_2CO_3 (166 mg, 0.51 mmol), MeI (30 μL, 0.43 mmol) and DMF (1.5 mL). Purification by MPLC on SiO_2 (0-5% MeOH/DCM) gave a white solid (50 mg, 96%). R_f 0.42 (5% MeOH/DCM); m.p. 195-197 °C; λ_{max} (EtOH)/nm 258.4, 225.6; IR ν_{max}/cm^{-1} 3054, 2973, 2924, 2221 (CN), 1703 (C=O), 1599 (C=O); ^{1}H NMR (500 MHz, CD_3OD) δ_H 1.28 (6H, s, 2×C*H₃*), 3.03 (3H, s, N-C*H₃*), 5.28 (2H, s, C*H₂*), 6.51 (1H, s, 1×pyridone-*H*), 7.17 (1H, d, *J* = 7.9 Hz, Ar-*H*), 7.35-7.38 (2H, m, Ar-*H* + pyridone-*H*), 7.52 (1H, td, *J* = 7.7, 1.2 Hz, Ar-*H*), 7.67 (1H, dd, *J* = 7.7, 1.0 Hz, Ar-*H*); ^{13}C NMR (125 MHz, CD_3OD) δ_C 22.6 (2×CH$_3$), 25.4 (N-CH$_3$), 44.2 (C(CH$_3$)$_2$), 51.2 (NCH$_2$Ar), 111.2 (C-Ar), 113.7 (CH-pyridone), 115.7 (CH-pyridone), 116.9 (CN), 127.5 (C-Ar), 127.7 (C-Ar), 128.1 (C-Ar), 132.9 (C-Ar), 133.1 (C-Ar), 139.9 (C-Ar), 154.0 (C-Ar), 162.2 (C=O pyridone), 179.0 (C=O pyrrolidine); HRMS calcd for $C_{18}H_{18}N_3O_2$ [M+H]$^+$ 308.1394, found 308.1396.

3-((3,3-Dimethyl-2,5-dioxo-1,2,3,5-tetrahydro-6*H*-pyrrolo[2,3-*c*]pyridin-6-yl)methyl)benzonitrile (15p)

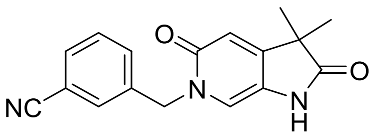

Prepared according to general procedure B using compound **12** (100 mg, 0.342 mmol), 3-(bromomethyl)benzonitrile (134 mg, 0.684 mmol) and MeCN (2.5 mL). Purification by MPLC on SiO_2 (0-7% MeOH/DCM) gave a white solid (65 mg, 65%). R_f 0.32 (5% MeOH/DCM); m.p. 310 °C (degraded); λ_{max} (EtOH)/nm 338.4, 255.8, 223.0; IR ν_{max}/cm^{-1} 3059, 2964, 2925, 2723, 2227 (CN), 1709 (C=O), 1584 (C=O); ^{1}H NMR (500 MHz, DMSO-d_6) δ_H 1.25 (6H, s, 2×C*H₃*), 5.09 (2H, s, C*H₂*), 6.56 (1H, s, 1×pyridone-*H*), 7.29 (1H, s, 1×pyridone-*H*), 7.55-7.58 (1H, m, Ar*H*), 7.62-7.65 (1H, m, Ar*H*), 7.76-7.79 (2H, m, Ar*H*), 10.31 (1H, s, N*H*); ^{13}C NMR (125 MHz, DMSO-d_6) δ_C 24.0 (2×CH$_3$), 44.1 (C(CH$_3$)$_2$), 50.9 (NCH$_2$Ar), 111.8 (C-Ar) 114.8 (CH-pyridone), 115.8 (CH-pyridone), 119.1 (CN), 124.1 (C-Ar), 130.3 (C-Ar), 131.8 (C-Ar), 131.9 (C-Ar), 133.2 (C-Ar), 139.8 (C-Ar), 154.4 (C-Ar), 160.7 (C=O pyridone), 180.2 (C=O pyrrolidinone); HRMS calcd for $C_{17}H_{16}N_3O_2$ [M+H]$^+$ 294.1237, found 294.1242.

3-((1,3,3-trimethyl-2,5-dioxo-1,2,3,5-tetrahydro-6*H*-pyrrolo[2,3-*c*]pyridin-6-yl)methyl)benzonitrile (17p)

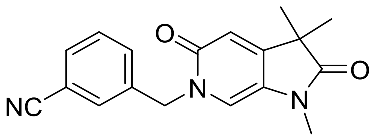

Prepared according to general procedure A using compound **15p** (40 mg, 0.136 mmol), Cs_2CO_3 (133 mg, 0.408 mmol), MeI (21 µL, 0.34 mmol) and DMF (1.5 mL). Purification by MPLC on SiO_2 (0-5% MeOH/DCM) gave a white solid (38 mg, 91%). R_f 0.40 (5% MeOH/DCM); m.p. 170-172 °C; λ_{max} (EtOH)/nm 338.4, 259.4, 225.4; IR ν_{max}/cm^{-1} 3033, 2926, 2864, 2229 (CN), 1713 (C=O), 1579 (C=O); ^{1}H NMR (500 MHz, CD_3OD) δ_H 1.26 (6H, s, 2×C*H₃*), 3.03 (3H, s, N-CH_3), 5.11 (2H, s, CH_2), 6.51 (1H, s, 1×pyridone-*H*), 7.38 (1H, s, Ar-*H*), 7.43 (1H, app t, *J* = 7.8 Hz, Ar-*H*), 7.55-7.58 (2H, m, 2×Ar-*H*), 7.63-7.65 (1H, m, Ar-*H*); ^{13}C NMR (125 MHz, CD_3OD) δ_C 22.6 (2×*C*H_3), 25.5 (N-*C*H_3), 44.2 (*C*(CH_3)$_2$), 51.8 (N*C*H_2Ar), 112.4 (C-Ar), 113.7 (CH-pyridone), 115.2 (CH-pyridone), 118.0 (CN), 127.7 (C-Ar), 129.5 (C-Ar), 131.2 (C-Ar), 131.3 (C-Ar), 132.3 (C-Ar), 138.5 (C-Ar), 153.8 (C-Ar), 162.1 (C=O pyridone), 179.0 (C=O pyrrolidinone); HRMS calcd for $C_{18}H_{18}N_3O_2$ [M+H]$^+$ 308.1394, found 308.1393.

2-((1,3,3-Trimethyl-2,5-dioxo-1,2,3,5-tetrahydro-6*H*-pyrrolo[2,3-*c*]pyridin-6-yl)methyl)benzoic acid (17q)

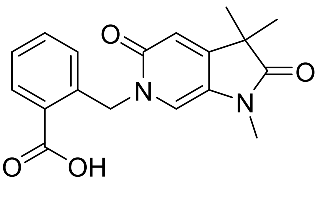

Compound **17p** (32 mg, 0.10 mmol) was dissolved in ethanol (4 mL), NaOH (1 M in water, 2 mL) was added and the mixture was stirred at 80 ˚C for 16 h. Further 1 M NaOH (1 mL) was added and the reaction mixture was stirred at 100 ˚C for 7 h. Ethanol was evaporated *in vacuo* and the residue was acidified to pH 1 using 1 M aqueous HCl. The aqueous layer was extracted with EtOAc (4×20 mL), the organic layers were combined, dried ($MgSO_4$) and the solvent removed *in vacuo* (11 mg, 32%). R_f 0.29 (5% MeOH/DCM); m.p. 268 °C (degraded); λ_{max} (EtOH)/nm 339.0, 258.8; IR ν_{max}/cm^{-1} 3071-2180 (broad spectrum), 2919, 2851, 1713 (C=O), 1545 (C=O); ^{1}H NMR (500 MHz, CD_3OD) δ_H 1.29 (6H, s, 2×CH_3), 2.99 (1H, s, N-CH_3), 5.52 (2H, s, CH_2), 6.54 (1H, s, 1×pyridone-*H*), 6.88 (1H, d, *J* = 7.8 Hz, Ar-*H*), 7.26-7.29 (1H, m, 1×Ar-*H*), 7.31 (1H, s, 1×pyridone-*H*), 7.37 (1H, td, *J* = 7.8 and 1.3 Hz, Ar-*H*), 7.93 (1H, dd, *J*

= 7.8 and 1.0 Hz, Ar-*H*); ^{13}C NMR (125 MHz, CD_3OD) δ_C 22.7 (2×CH_3), 25.4 (N-CH_3), 44.2 (*C*(CH_3)$_2$), 51.1 (NCH_2Ar), 113.6 (CH-pyridone), 115.8 (CH-pyridone), 126.9 (C-Ar), 127.1 (C-Ar), 127.6 (C-Ar), 130.8 (C-Ar), 132.0 (C-Ar), 135.8 (C-Ar), 137.7 (C-Ar), 153.6 (C-Ar), 162.4 (C=O pyridone), 169.4 (*C*OOCH_3), 179.1 (C=O pyrrolidinone); HRMS calcd for $C_{18}H_{19}N_2O_4$ $[M+H]^+$ 327.1339, found 327.1343.

6-(4-Chlorobenzyl)-3,3-diethyl-1*H*-pyrrolo[2,3-c]pyridine-2,5(3*H*,6*H*)-dione (16a)

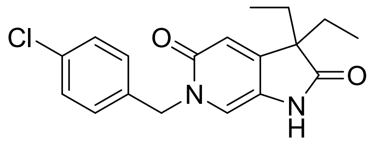

Prepared according to general procedure B using **13** (120 mg, 0.37 mmol), 4-chloromethylbenzyl bromide (153 mg, 0.74 mmol, 2 eq.) and MeCN (3 mL). Purification by MPLC on silica (0-20% MeOH/DCM) gave the product as a white solid (83 mg, 67%); R_f 0.35 (5% MeOH/DCM); ^{1}H NMR (500 MHz; $CDCl_3$) δ_H 0.73 (6H, t, J = 7.4 Hz, 2 × CH_2CH_3), 1.70-1.83 (2H, m, CH_2CH_3), 1.89-1.97 (2H, m, CH_2CH_3), 5.12 (2H, s, NCH_2), 6.76 (1H, s, H-pyridone), 6.85 (1H, s, H-pyridone), 7.21-7.37 (4H, m, 2 × H-phenyl), 7.42 (1H, br s, NH); ^{13}C NMR (125 MHz, $CDCl_3$) δ_C 8.75, 30.5, 51.5, 54.8, 113.9, 116.1, 124.7, 129.2, 129.6, 134.1, 134.9, 150.6, 161.2, 179.8; MS ES+ 331.3 $[M+H]^+$

6-(4-Chlorobenzyl)-3,3-diethyl-1-methyl-1*H*-pyrrolo[2,3-c]pyridine-2,5(3*H*,6*H*)-dione (18a)

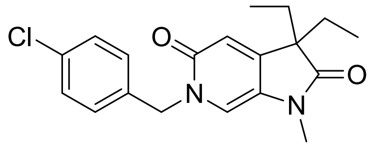

Prepared according to general procedure A using **16a** (75 mg, 0.23 mmol), MeI (28 µL, 0.45 mmol, 2 eq.), cesium carbonate (221 mg, 0.68 mmol, 3 eq.) and DMF (2 mL). Purification by MPLC on silica (50-100% EtOAc/petrol) gave the product as a white solid (33 mg, 43%); R_f 0.5 (5% MeOH/DCM); m.p. 146-148 °C; IR ν_{max}/cm^{-1} 3045, 2966, 2928, 1708 (C=O), 1594 (C=O); ^{1}H NMR (500 MHz; $CDCl_3$) δ_H 0.67 (6H, t, J = 7.4 Hz, 2 × CH_2CH_3), 1.68-1.78 (2H, m, CH_2CH_3), 1.87-1.97 (2H, m, CH_2CH_3), 3.07 (3H, s, N-Me pyrrolidinone), 5.12 (2H, s, NCH_2), 6.55 (1H, s, H-pyridone), 6.61 (1H, s, H-pyridone), 7.24-7.29 (2H, m, 2 × H-phenyl), 7.32-7.36 (2H, m, 2 × H-phenyl); ^{13}C NMR (125 MHz; $CDCl_3$) δ_C 8.8 (2 × CH_2CH_3), 26.2

(NMe), 30.4 (2 × CH_2CH_3), 51.7 (CH_2Ar), 54.4(*C*$(Et)_2$), 112.0 (CH-pyridone), 115.9 (CH-pyridone), 128.6, 129.2, 129.5, 134.1, 134.9, 149.8, 177.0 (C-O pyrrolidinone); HRMS calcd for $C_{19}H_{22}N_2O_2Cl$ $[M+H]^+$ 345.1364, found 345.1358.

3,3-Diethyl-6-(4-(trifluoromethyl)benzyl)-1*H*-pyrrolo[2,3-c]pyridine-2,5(3*H*,6*H*)-dione (16b)

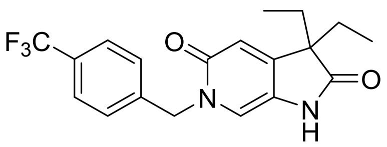

Prepared according to general procedure B using **13** (70 mg, 0.22 mmol), 4-trifluoromethylbenzyl bromide (104 mg, 0.43 mmol, 2 eq) and MeCN (2 mL). Purification by MPLC on silica (0-3% MeOH/DCM) gave the title compound as a white solid (52 mg, 71%); R_f 0.35 (5% MeOH/DCM); ^{1}H NMR (500 MHz; $CDCl_3$) δ_H 0.75 (3H, t, J = 7.4 Hz, 2 x Me), 1.70-1.80 (2H, m, CH_2CH_3), 1.89-1.98 (2H, m, CH_2CH_3), 5.16 (2H, s, $CONCH_2Ar$), 6.51 (1H, s, H-pyridone), 6.80 (1H, s, H-pyridone), 7.42 (2H, d, J = 8.0 Hz, 2 × H-phenyl), 7.59 (1H, br s, NH), 7.62 (2H, d, J = 8.0 Hz, 2 × H-phenyl); ^{13}C NMR (125 MHz; $CDCl_3$) δ_C 8.8 (2 × CH_2CH_3), 30.5 (2 × CH_2CH_3), 51.8 (CH_2Ar), 54.8 (*C*$(Et)_2$), 113.8 (CH-pyridone), 116.2 (CH-pyridone), 124.7, 125.9 (q, J = 3.8 Hz, 2 × CF_3CCH-Ar), 128.3, 140.4, 150.8, 161.1, 179.5 (C=O pyrrolidinone); ^{19}F NMR (125 MHz; $CDCl_3$) δ_F -62.66; MS ES+ 365.2 $[M+H]^+$

3,3-Diethyl-1-methyl-6-(4-(trifluoromethyl)benzyl)-1*H*-pyrrolo[2,3-c]pyridine-2,5(3*H*,6*H*)-dione (18b)

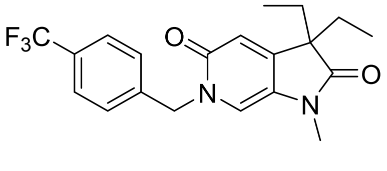

Prepared according to general procedure A using **16b** (45 mg, 0.13 mmol), MeI (25 uL, 0.40 mmol, 2 eq), cesium carbonate (87 mg, 0.27 mmol, 3 eq) and MeCN (2 mL). Purification by MPLC on silica (0-3% MeOH/DCM) gave the title compound as a white solid (36 mg, 77%); R_f 0.55 (5% MeOH/DCM); m.p. 141-143 ºC; IR ν_{max}/cm^{-1} 3043, 2959, 2930, 1707 (C=O), 1597 (C=O); ^{1}H NMR (500 MHz; $CDCl_3$) δ_H 0.68 (3H, t, J = 7.4 Hz, 2 x Me), 1.74 (2H, m, CH_2CH_3), 1.94 (2H, m, CH_2CH_3), 3.08 (3H, s, N-Me pyrrolidinone), 5.20 (2H, s, $CONCH_2Ar$), 6.53 (1H, s, H-pyridone), 6.62 (1H, s, H-pyridone), 7.43 (2H, d, J = 8.5 Hz, 2 × H-phenyl),

7.62 (2H, d, *J* = 8.5 Hz, 2 × H-phenyl); ^{13}C NMR (125 MHz; $CDCl_3$) δ_C 8.7 (2 × CH_2CH_3), 26.2 (NMe), 30.4 (2 × CH_2CH_3), 51.9 (CH_2Ar), 54.4 (*C*($Et)_2$), 112.0 (CH-pyridone), 115.9 (CH-pyridone), 124.0 (q, *J* = 272.5 Hz, CF_3), 125.9 (q, *J* = 3.6 Hz), 128.1, 128.5, 130.2, 130.5 140.5, 149.9, 161.4 (CO pyridone), 177.0 (C-O pyrrolidinone); HRMS calcd for $C_{20}H_{22}N_2O_2F_3$ $[M+H]^+$ 379.1628, found 379.1622.

3-Benzylidene-5-methoxy-1,3-dihydro-2*H*-pyrrolo[2,3-*c*]pyridin-2-one (19a)

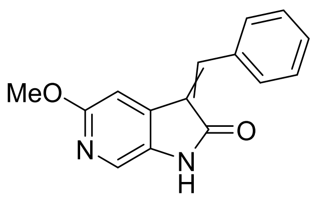

Prepared according to general procedure C using **11** (250 mg, 0.95 mmol), benzaldehyde (0.10 mL, 1.04 mmol), piperidine (0.29 mL, 2.93 mmol) and THF (2 mL). Purification by MPLC on SiO_2 (0-10% MeOH/DCM) gave an orange solid (210 mg, 88%, E:Z = 1:4 by ^1H NMR). R_f 0.36 (5% MeOH/DCM); m.p. 221-224 °C; λ_{max} (EtOH)/nm 323.2, 260.6; IR ν_{max}/cm^{-1} 3161, 2978, 2938, 2345, 1694 (C=O); ^1H NMR (500 MHz, DMSO-d_6) δ_H 3.76 (3H, s, OC*H*$_3$, Z-isomer), 3.83 (3H, s, OC*H*$_3$, E-isomer), 6.84 (1H, s, 1×pyridine-*H*, Z-isomer), 7.26 (1H, s, 1×pyridine-*H*, E-isomer), 7.50-7.59 (3H, m 3×Ar-H, E and Z-isomers), 7.64 (1H, s, 1×pyridine-*H*, E-isomer), 7.71-7.74 (3H, m, 2×Ar-*H* + 1×pyridine-*H*, Z-isomer), 7.90 (1H, s, C*H*Ar, Z-isomer), 8.09 (1H, s, C*H*Ar, E-isomer), 8.43-8.45 (2H, m, 2×Ar-*H*, E-isomer), 10.67 (1H, s, N*H*, E and Z isomers); ^{13}C NMR (125 MHz, DMSO-d_6) δ_C 53.7 (O*C*H$_3$, Z-isomer), 53.7 (O*C*H$_3$, E-isomer), 101.8 (CH-pyridine, E-isomer), 104.0 (CH-pyridine, Z-isomer), 125.5 (CH-pyridine, E-isomer), 125.8 (C-Ar), 126.4 (CH-pyridine, Z-isomer), 126.9 (C-Ar), 128.8 (C-Ar), 129.4 (C-Ar), 130.0 (C-Ar), 131.1 (C-Ar), 132.0 (C-Ar), 132.4 (C-Ar), 132.8 (C-Ar), 133.1 (C-Ar), 133.8 (C-Ar), 134.2 (C-Ar), 137.7 (C-Ar), 142.1 (*C*H-Ar, Z-isomer), 142.3 (*C*HAr, E-isomer), 159.4 (C-Ar), 159.7 (C-Ar), 167.0 (C=O, E-isomer), 168.2 (C=O, Z-isomer); HRMS calcd for $C_{15}H_{13}N_2O_2$ $[M+H]^+$ 551.1310, found 551.1297.

3-Benzyl-5-methoxy-1,3-dimethyl-1,3-dihydro-2*H*-pyrrolo[2,3-*c*]pyridin-2-one (21a)

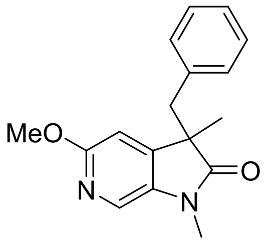

Prepared according to general procedure D using **19a** (122 mg, 0.483 mmol), 10% Pd/C (40 mg), THF (18 mL) and MeOH (9 mL) in step A, and Cs_2CO_3 (315 mg, 0.97 mmol), MeI (55 µL, 0.88 mmol), DMF (7.5 mL) in Step B. Purification by MPLC on SiO_2 (0-5% MeOH/DCM) gave a colourless sticky solid (75 mg, 55%). R_f 0.70 (5% MeOH/DCM); m.p. 73-76 °C; λ_{max} (EtOH)/nm 305.0, 251.0; IR ν_{max}/cm^{-1} 2927, 2655, 1708 (C=O); ^{1}H NMR (500 MHz, $CDCl_3$) δ_H 1.45 (3H, s, C*H*$_3$), 2.97 (1H, d, *J* = 13.2 Hz, C*H*$_2$Ar), 3.00 (3H, s, N-C*H*$_3$), 3.14 (1H, d, *J* = 13.2 Hz, C*H*$_2$Ar), 3.89 (3H, s, OC*H*$_3$), 6.55 (1H, d, *J* = 0.6 Hz, 1×pyridine-*H*), 6.88-6.90 (2H, m, 2×Ar-*H*), 7.09-6.10 (3H, m, 3×Ar-*H*), 7.41 (1H, d, *J* = 0.6 Hz, 1×pyridine-*H*); ^{13}C NMR (125 MHz, $CDCl_3$) δ_C 22.6 (2×*C*H$_3$), 26.2 (N-*C*H$_3$), 44.0 (*C*H$_2$Ar), 50.3 (*C*(CH$_3$)CH$_2$Ar), 53.7 (O*C*H$_3$), 106.8 (CH-pyridine), 123.5 (CH-pyridine), 126.8 (C-Ar), 127.8 (2×C-Ar), 129.8 (2×C-Ar), 134.8 (C-Ar), 135.4 (C-Ar), 146.5 (C-Ar), 160.2 (C=O pyridone), 178.4 (C=O pyrrolidinone); HRMS calcd for $C_{17}H_{18}N_2O_2$ $[M+H]^+$ 253.0972, found 253.0971.

3-Benzyl-6-(4-chlorobenzyl)-1,3-dimethyl-1,6-dihydro-2*H*-pyrrolo[2,3-*c*]pyridine-2,5(3*H*)-dione (22a)

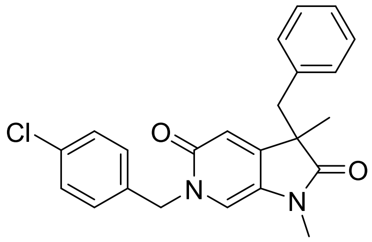

Prepared according to general procedure B using **21a** (60 mg, 0.21 mmol), 1-(bromomethyl)-4-chlorobenzene (87 mg, 0.42 mmol) and MeCN (2 mL). Reaction time: 30 min. Purification by MPLC on SiO_2 (0-6% MeOH/DCM) gave a white solid (53 mg, 64%). R_f 0.45 (5% MeOH/DCM); m.p. 175-178 °C; λ_{max} (EtOH)/nm 340.4, 260.0; IR ν_{max}/cm^{-1} 3049, 2971, 2926, 1718 (C=O), 1594 (C=O); ^{1}H NMR (500 MHz, $CDCl_3$) δ_H 1.48 (3H, s, C*H*$_3$), 2.81 (3H, s, N-C*H*$_3$), 2.90 (1H, d, *J* = 13.1 Hz, C*H*$_2$Ar), 3.22 (1H, d, *J* = 13.1 Hz, C*H*$_2$Ar), 5.01 (1H, d, *J* = 14.9 Hz, 1×4-chlorophenyl-*CH$_2$*), 5.06 (1H, d, *J* = 14.9 Hz, 1×4-chlorophenyl-*CH$_2$*), 6.33 (1H,

s, 1×pyridone-*H*), 6.58 (1H, s, 1×pyridone-*H*), 6.95-6.96 (2H, m, 2×4-chlorophenyl-*H*), 7.09-7.12 (5H, m, 5×Ph-*H*), 7.29-7.31 (2H, m, 2×4-chlorophenyl-*H*); ^{13}C NMR (125 MHz, $CDCl_3$) δ_C 22.6 (2×CH_3), 26.2 (N-CH_3), 44.7 (CH_2Ar), 50.3 ($C(CH_3)CH_2$Ar), 51.6 ((4-chlorophenyl-CH_2N), 112.4 (CH-pyridone), 116.3 (CH-pyridone), 127.1 (C-Ar), 127.6 (C-Ar), 128.0 (2×C-Ar), 129.1 (2×C-Ar), 129.2 (2×C-Ar), 129.9 (2×C-Ar), 134.0 (C-Ar), 135.0 (C-Ar), 135.4 (C-Ar), 150.5 (C-Ar), 161.3 (C=O pyridone), 177.0 (C=O pyrrolidinone); HRMS calcd for $C_{23}H_{22}ClN_2O_2$ [M(^{35}Cl)+H]$^+$ 393.1364, found 393.1359.

5-Methoxy-3-(pyridin-2-ylmethylene)-1,3-dihydro-2*H*-pyrrolo[2,3-*c*]pyridin-2-one (19b)

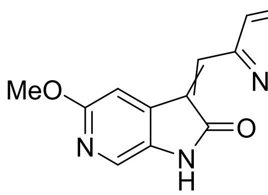

Prepared according to general procedure C using **11** (250 mg, 0.95 mmol), picolinaldehyde (99 μL, 1.04 mmol), piperidine (291 μL, 2.95 mmol) and THF (2 mL). Purification by MPLC on SiO_2 (0-10% MeOH/DCM) gave an orange solid (120 mg, 50%). R_f 0.39 (5% MeOH/DCM); m.p. 217-230 °C; λ_{max} (EtOH)/nm 333.2, 263.6, 210.2; IR ν_{max}/cm^{-1} 3163, 3105, 2992, 2943, 2851, 1717 (C=O); ^{1}H NMR (500 MHz, DMSO-d_6) δ_H 3.83 (3H, s, OCH_3), 7.52-7.55 (1H, m, pyridine-*H*), 7.71 (1H, s, 1×methoxypyridine-*H*), 7.80 (1H, s, C*H*Ar), 7.97-8.02 (2H, m, 2×pyridine-H), 8.47 (1H, s, 1×methoxypyridine-*H*), 8.93-8.95 (2H, m, 2×Ar-*H*), 10.68 (1H, s, N*H*); ^{13}C NMR (125 MHz, DMSO-d_6) δ_C 53.7 (OCH_3), 109.6 (CH-methoxypyridine), 125.5 (CH-methoxypyridine), 125.8 (C-Ar), 128.3 (C-Ar), 130.1 (C-Ar), 133.1 (*C*=CH), 134.2 (C-Ar), 138.0 (C-Ar), 139.4 (C=*C*H), 150.3 (C-Ar), 152.9 (C-Ar), 160.0 (C-Ar), 168.8 (C=O); HRMS calcd for $C_{14}H_{11}N_3O_2$ [M+H]$^+$ 254.0924, found 254.0927.

5-Methoxy-1,3-dimethyl-3-(pyridin-2-ylmethyl)-1,3-dihydro-2*H*-pyrrolo[2,3-*c*]pyridin-2-one (21b)

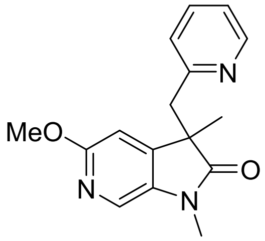

Prepared according to general procedure D using **19b** (95 mg, 0.37 mmol), 10% Pd/C (30 mg), THF (12 mL) and MeOH (6 mL) in step A, and Cs_2CO_3 (257 mg, 0.79 mmol), MeI (47 µL, 0.75 mmol), DMF (4 mL) in Step B. Purification by MPLC on SiO_2 (0-5% MeOH/DCM) gave a beige solid (60 mg, 56%). R_f 0.48 (5% MeOH/DCM); m.p. 105-108 °C; λ_{max} (EtOH)/nm 306.2, 252.2, 205.0; IR ν_{max}/cm^{-1} 3005, 3009, 2976, 2937, 1700 (C=O); ^{1}H NMR (500 MHz, $CDCl_3$) δ_H 1.46 (3H, s, CH_3), 3.14 (3H, s, N-CH_3), 3.22 (1H, d, J = 13.8 Hz, CH_2Ar), 3.41 (1H, d, J = 13.8 Hz, CH_2Ar), 3.85 (3H, s, OCH_3), 6.54 (1H, s, 1×methoxypyridine-H), 6.98-7.03 (2H, m, 2×Ar-H), 7.45-7.49 (2H, m, 2×Ar-H), 8.30-8.32 (2H, m, 2×Ar-H); ^{13}C NMR (125 MHz, $CDCl_3$) δ_C 23.4 (2×CH$_3$), 26.5 (N-CH$_3$), 45.0 (CH$_2$Ar), 48.9 (C(CH$_3$)CH$_2$Ar), 53.5 (OCH$_3$), 106.9 (CH-methoxypyridine), 121.7 (C-Ar), 123.5 (CH-methoxypyridine), 123.8 (C-Ar), 134.9 (C-Ar), 136.1 (C-Ar), 146.4 (C-Ar), 148.7 (C-Ar), 156.3 (C-Ar), 160.3 (C-Ar), 178.8 (C=O); HRMS calcd for $C_{16}H_{18}N_3O_2$ [M+H]$^+$ 284.1394, found 284.1393.

6-(4-Chlorobenzyl)-1,3-dimethyl-3-(pyridin-2-ylmethyl)-1,6-dihydro-2*H*-pyrrolo[2,3-*c*]pyridine-2,5(3*H*)-dione (22b)

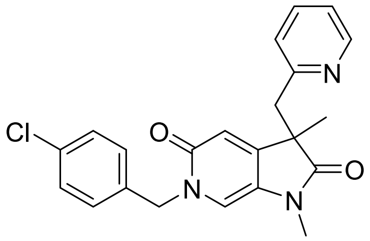

Prepared according to general procedure B using **21b** (46 mg, 0.16 mmol), 1-(bromomethyl)-4-chlorobenzene (67 mg, 0.32 mmol) and MeCN (1 mL). Reaction time: 30 min. Purification by MPLC on SiO_2 (0-6% MeOH/DCM) gave a brown solid (32 mg, 50%). R_f 0.30 (5% MeOH/DCM); m.p. 182-185 °C; λ_{max} (EtOH)/nm 340.6, 260.8, 222.0; IR ν_{max}/cm^{-1} 3187, 3044, 2966, 2927, 1711 (C=O), 1586 (C=O); ^{1}H NMR (500 MHz, CD_3OD) δ_H 1.48 (3H, s, CH_3), 3.02 (3H, s, N-CH_3), 3.26 (1H, d, J = 14.1 Hz, CH_2-pyridine), 3.41 (1H, d, J = 14.1 Hz, CH_2-pyridine), 5.04 (1H, d, J = 14.8 Hz, 1×4-chlorophenyl-CH_2), 5.12 (1H, d, J = 14.8 Hz, 1×4-chlorophenyl-CH_2), 6.51 (1H, s, 1×pyridone-H), 7.09-7.14 (2H, m, 2×pyridine-H), 7.16 (1H, s, 1×pyridone-H), 7.18-7.19 (2H, m, 2×4-chlorophenyl-H), 7.30-7.32 (2H, m, 2×4-chlorophenyl-H), 7.57 (1H, td, J = 11.5 and 1.9 Hz, 1×pyridine-H), 8.22-8.23 (1H, m, 1×pyridine-H); ^{13}C NMR (125 MHz, CD_3OD) δ_C 23.5 (2×CH$_3$), 26.8 (N-CH$_3$), 45.8 (CH$_2$-pyridine), 50.0 (C(CH$_3$)CH$_2$Ar), 52.8 (4-chlorophenyl-CH$_2$N), 116.0 (CH-pyridone), 116.1 (CH-pyridone), 123.2 (C-Ar), 125.3 (C-Ar), 129.7 (C-Ar), 129.8 (2×C-Ar), 130.2 (2×C-Ar),

134.7 (C-Ar), 137.0 (C-Ar), 137.8 (C-Ar), 149.6 (C-Ar), 152.6 (C-Ar), 157.5 (C-Ar), 163.2 (C=O pyridone), 179.5 (C=O pyrrolidinone); HRMS calcd for $C_{22}H_{21}ClN_3O_2$ $[M(^{35}Cl)+H]^+$ 394.1311, found 394.1311.

5-Methoxy-3-((2-methylthiazol-4-yl)methylene)-1,3-dihydro-2H-pyrrolo[2,3-c]pyridin-2-one (19c)

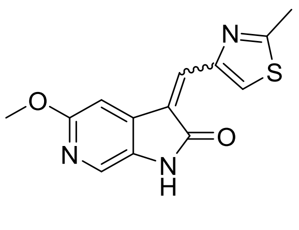

Prepared according to general procedure C using **11** (200 mg, 0.767 mmol), THF (1.5 mL), 2-methylthiazole-5-carboxaldehyde (106 mg, 0.832 mmol) and piperidine (235 µL, 2.378 mmol). Purification by MPLC on SiO_2 (0-10% MeOH/DCM) gave an orange solid (98 mg, 48%). ^{1}H NMR (500 MHz, CD_3OD) δ_H 2.84 (3H, s, thiazole C*H₃*), 3.84 (3H, s, pyridine OCH_3), 7.68 (1H, d, *J* = <1.0 Hz, pyridine C^7-*H*), 7.70 (1H, s, CH), 8.47 (1H, s, thiazole C^4-*H*), 8.49 (1H, s, pyridine C^4-*H*), 10.6 (1H, bs, *NH*). ^{13}C NMR (125 MHz, CD_3OD) δ_C 19.8, 53.7, 109.1, 123.7, 125.6, 131.5, 132.5, 133.3, 133.8, 150.7, 159.9, 167.8, 169.3. LRMS $[M]^+$ 274.2.

5-Methoxy-1,3-dimethyl-3-((2-methylthiazol-4-yl)methyl)-1,3-dihydro-2H-pyrrolo[2,3-c]pyridin-2-one (21c)

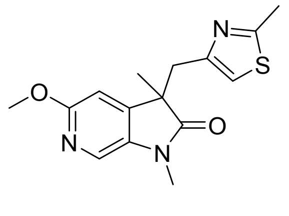

Prepared according to general procedure D using **19c** (98 mg, 0.359 mmol), MeOH (8 mL), THF (2.0 mL); MeI (47 µL, 0.754 mmol), cesium carbonate (269 mg, 0.826 mmol), DMF (2.0 mL). Purification by MPLC on SiO_2 (0-5% MeOH/DCM) gave a dark brown oil (91 mg, 83%).^{1}H NMR (500 MHz, $CDCl_3$) δ_H 1.37 (CCH_3), 2.48 (3H, s, thiazole CH_3), 3.08 (3H, s, NCH_3), 3.17-3.09 (2H, CH_2), 3.80 (3H, s, pyridine OCH_3), 6.45 (1H, d, *J* = <1.0 Hz, pyridine C^7-*H*), 6.53 (1H, s, thiazole C^4-*H*), 7.45 (1H, d, *J* = <1.0 Hz, pyridine C^4-*H*). ^{13}C NMR (125 MHz, $CDCl_3$) δ_C 19.0, 22.8, 26.5, 38.5, 48.9, 53.5, 107.1, 115.1, 123.5, 134.9, 146.4, 151.0, 160.4, 164.8, 178.7. LRMS $[M]^+$ 304.2.

6-(4-Chlorobenzyl)-1,3-dimethyl-3-((2-methylthiazol-4-yl)methyl)-1,6-dihydro-2H-pyrrolo[2,3-c]pyridine-2,5(3H)-dione (22c)

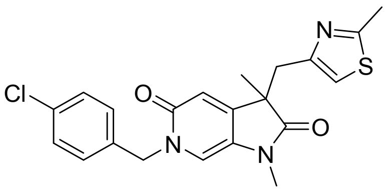

Prepared according to general procedure B using **21c** (34 mg, 0.111 mmol), 4-chlorobenzyl bromide (46 mg, 0.222 mmol) and acetonitrile (0.75 mL). Reaction time: 30 min. Purification by MPLC on SiO_2 (0-6% MeOH/DCM) gave an off white solid (37 mg, 81%). ^{1}H NMR (500 MHz, $CDCl_3$) δ_H 1.39 (3H, s, CCH_3), 2.47 (3H, s, thiazole C*H₃*), 2.92 (3H, s, NCH_3), 3.05 (1H, d, *J* = 14.2 Hz, CCH_2), 3.21 (1H, d, *J* = 14.2 Hz, CCH_2), 5.02-4.95 (2H, m, NCH_2), 6.36 (1H, s, pyridone C^7-*H*), 6.42 (1H, s, thiazole C^4-*H*), 6.61 (1H, s, pyridone C^4-*H*), 7.10 (2H, d, *J* = 8.3 Hz, chlorobenzyl C*H*), 7.24 (2H, d, *J* = 8.4 Hz, chlorobenzyl C*H*). ^{13}C NMR (125 MHz, $CDCl_3$) δ_C 19.1, 22.9, 26.4, 38.8, 48.4, 51.4, 112.0, 115.4, 116.5, 127.5, 129.0 (2×), 129.1 (2×), 133.9, 135.1, 150.2, 150.7, 161.3, 164.9, 177.4. LRMS $[M]^+$ 414.2. HRMS calcd for $C_{21}H_{21}N_3O_4ClS$ $[M+H]^+$ 414.1038, found 414.1026.

3-(2,2-Dimethylpropylidene)-5-methoxy-1*H*-pyrrolo[2,3-c]pyridin-2(3*H*)-one (19d)

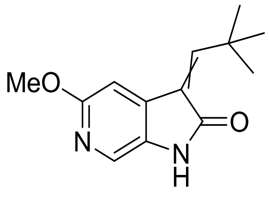

Prepared according to general procedure C using **11** (200 mg, 0.76 mmol), pivaloyl aldehyde (91 µL, 0.84 mmol, 1.1 eq.), piperidine (244 µL, 2.28 mmol, 3 eq.) and THF (2 mL). Purification by MPLC (0-6% MeOH/DCM) gave the title compound as a single stereoisomer (52 mg, 30%). R_f 0.3 (5% MeOH/DCM); ^{1}H NMR (500 MHz; $CDCl_3$) δ_H 1.39 (3H, s, tBu), 3.93 (3H, s, OMe), 7.26 (1H, s, H-Ar), 7.40 (1H, s, HC=C), 7.48 (1H, br s, NH), 7.72 (1H, d, *J* = 0.5 Hz, H-Ar); ^{13}C NMR (125 MHz, $CDCl_3$) δ_C 29.0 ($C(CH_3)_3$), 33.3 ($C(CH_3)_3$), 53.7 (OCH_3), 109.0, 125.5, 125.8, 131.8, 132.3, 159.4, 160.1, 168.9 (C=O); MS ES+ 233.2 $[M+H]^+$

5-Methoxy-3-neopentyl-1*H*-pyrrolo[2,3-c]pyridin-2(3*H*)-one (20d)

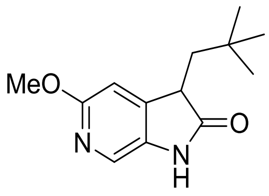

Prepared according to general procedure D step 1 using **19d** (52 mg, 0.22 mmol), MeOH (3 mL) and DCM (1 mL) to give the title compound as a yellow solid (52 mg, 100%); R_f 0.5 (5% MeOH/DCM); ^{1}H NMR (500 MHz; $CDCl_3$) δ_H 1.06 (9H, s, $C(CH_3)_3$), 1.64 (1H, dd, J = 5.2 and 14.4 Hz, $CH_aH_b{}^tBu$), 2.02 (1H, dd, J = 3.9 and 14.4 Hz, $CH_aH_b{}^tBu$), 3.30-3.34 (1H, m, COCH), 3.90 (3H, s, OMe), 6.70 (1H, s, H-pyridine), 7.67 (1H, s, H-pyridine); ^{13}C NMR not obtained; MS ES+ 235.2 $[M+H]^+$

5-Methoxy-1,3-dimethyl-3-neopentyl-1*H*-pyrrolo[2,3-c]pyridin-2(3*H*)-one (21d)

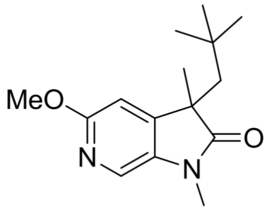

Prepared according to general procedure D step 2 using **20d** (52 mg, 0.22 mmol), Cs_2CO_3 (167 mg, 0.51 mmol, 2.3 eq.), MeI (29 uL, 0.47 mmol, 2.1 eq.) and DMF (4 mL). Purification by MPLC (0-2% MeOH/DCM) gave the title compound as a yellow solid (30 mg, 52%); R_f 0.55 (30% EtOAc/petrol); ^{1}H NMR (500 MHz; $CDCl_3$) δ_H 0.66 (9H, s, $C(CH_3)_3$), 1.29 (3H, s, CCH_3), 1.84 (1H, d, J = 14.5 Hz, $CH_aH_b{}^tBu$), 2.17 (1H, dd, J = 14.5 Hz, $CH_aH_b{}^tBu$), 3.22 (3H, s, NMe), 3.91 (3H, s, OMe), 6.66 (1H, s, H-pyridine), 7.65 (1H, s, H-pyridine); ^{13}C NMR (125 MHz; $CDCl_3$) δ_C 26.5, 28.2, 30.9, 31.9, 47.8, 50.5, 53.6, 107.3, 123.9, 134.8, 147.9, 160.3, 179.3 (C=O); MS ES+ 263.2 $[M+H]^+$

6-(4-Chlorobenzyl)-1,3-dimethyl-3-neopentyl-1*H*-pyrrolo[2,3-c]pyridine-2,5(3*H*,6*H*)-dione (22d)

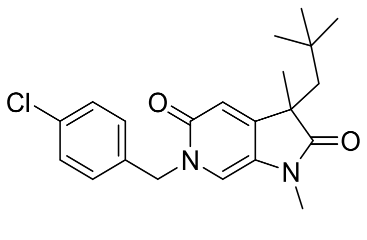

Prepared according to general procedure B using **21d** (30 mg, 0.12 mmol), 4-chlorobenzyl bromide (47 mg, 0.229 mmol, 2 eq.) and MeCN (2 mL). Purification by MPLC on silica (50-80% EtOAc/petrol) gave the title compound as an orange solid (28 mg, 65%); R_f 0.40 (5% MeOH/DCM); m.p. 156 ºC dec.; IR ν_{max}/cm^{-1} 3051, 2951, 1714 (C=O), 1597 (C=O); ^{1}H NMR (500 MHz; $CDCl_3$) δ_H 0.73 (9H, s, $C(CH_3)_3$), 1.30 (3H, s, CCH_3), 1.80 (1H, d, J = 14.3 Hz, $CH_aH_b{}^tBu$), 2.14 (1H, d, J = 14.3 Hz, $CH_aH_b{}^tBu$), 3.07 (3H, s, NMe), 5.03 (1H, d, J = 14.8 Hz, NCH_aH_bAr), 5.20 (1H, d, J = 14.8 Hz, NCH_aH_bAr), 6.56 (1H, s, H-pyridone), 6.63 (1H, s, H-pyridone), 7.21-7.27 (2H, m, H-Ph), 7.30-7.35 (2H, m, H-Ph); ^{13}C NMR (125 MHz; $CDCl_3$) δ_C 26.5, 28.0, 31.0, 32.0, 50.8, 51.5, 112.4, 116.5, 127.3, 129.1, 129.3, 134.0, 135.1, 151.7, 161.2, 177.9 (C=O); HRMS calcd for $C_{21}H_{26}ClN_2O_2$ [M(^{35}Cl)+H]$^+$ 373.1677, found 373.1671.

3-Methyloxetane-3-carbaldehyde (26)

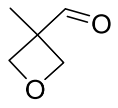

(3-Methyloxetan-3-yl)methanol (**25**, 0.49 mL, 4.89 mmol) was dissolved in DCM (15 mL) and cooled to 0 ˚C. Dess-Martin periodinane (3.11 g, 7.34 mmol) in DCM (30 mL) was added and the solution was stirred at 0 ˚C for 30 min and at r.t. for 3 h. The reaction mixture was poured into a solution of saturated aqueous $Na_2S_2O_3$ (30 mL) and saturated aqueous $NaHCO_3$ (30 mL) and stirred for 30 min. The aqueous layer was extracted with DCM (3×40 mL), the organic layers were combined, dried over $MgSO_4$ and the solvent removed *in vacuo* to give a colourless oil (380 mg, 78%). ^{1}H NMR (500 MHz, $CDCl_3$) δ_H 1.47 (3H, s, CH_3), 4.49 (2H, d, J = 6.3 Hz, CH_2), 4.86 (2H, d, J = 6.3 Hz, CH_2), 9.94 (1H, s, CHO); ^{13}C NMR (125 MHz, $CDCl_3$) δ_C 17.5 (CH_3), 49.3 (Cq), 76.7 (2×CH_2), 200.6 (C=O).

5-Methoxy-3-((3-methyloxetan-3-yl)methylene)-1,3-dihydro-2*H*-pyrrolo[2,3-*c*]pyridin-2-one (19e)

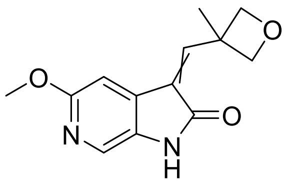

Prepared according to general procedure C using **11** (200 mg, 0.76 mmol), **26** (121 mg, 1.21 mmol), piperidine (150 µL, 1.51 mmol) and THF (0.7 mL). Purification by MPLC on SiO_2 (0-10% MeOH/DCM) gave an pale orange solid (120 mg, 50%). R_f 0.27 (5% MeOH/DCM); m.p. 154-156 °C; λ_{max} (EtOH)/nm 255.8; IR ν_{max}/cm^{-1} 3157, 3069, 3023, 2955, 2855, 1715 (C=O); Ratio of diatereoisomers by ^{1}H NMR = 3:5; ^{1}H NMR (500 MHz, DMSO-d_6) δ_H 1.59 (3H, s, CH_3-oxetane, isomer 1), 1.60 (3H, s, CH_3-oxetane, isomer 2), 3.80 (3H, s, OCH_3, isomer 1), 3.80 (3H, s, OCH_3, isomer 2), 4.48 (1H, d, J = 6.1 Hz, 2×oxetane-H, isomer 1), 4.67-4.69 (4H, m, 2×oxetane-H, isomer 1 and 2), 4.76 (1H, d, J = 5.8 Hz, 1×oxetane-H, isomer 2), 6.38 (1H, s, 1×methoxypyridine-*H*, isomer 2), 7.06 (1H, s, 1×methoxypyridine-*H*, isomer 1), 7.34 (1H, s, C*H*=CCO, isomer 2), 7.60 (1H, s, C*H*=CCO, isomer 2), 7.63 (1H, s, 1×methoxypyridine-*H*, isomer 1), 7.70 (1H, s, 1×methoxypyridine-*H*, isomer 2), 10.53 (1H, s, NH, isomer 1), 10.62 (1H, s, NH, isomer 2); ^{13}C-NMR was not obtained; HRMS calcd for $C_{13}H_{14}N_3O_2$ [M+H]$^+$ 247.1077, found 247.1080.

5-Methoxy-1,3-dimethyl-3-((3-methyloxetan-3-yl)methyl)-1,3-dihydro-2*H*-pyrrolo[2,3-*c*]pyridin-2-one (21e)

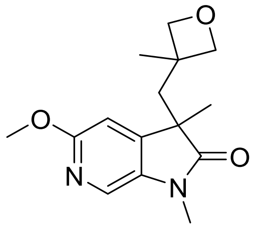

Prepared according to general procedure D using **19e** (58 mg, 0.24 mmol), 10% Pd/C (20 mg), THF (10 mL) and MeOH (5 mL) in step 1, and Cs_2CO_3 (176 mg, 0.54 mmol), MeI (30 µL, 0.48 mmol), DMF (4.7 mL) in step 2. Purification by MPLC on SiO_2 (0-5% MeOH/DCM) gave an off-white solid (30 mg, 42%). R_f 0.40 (5% MeOH/DCM); m.p. 98-101 °C; λ_{max} (EtOH)/nm 305.6, 249.8, 205.2; IR ν_{max}/cm^{-1} 3060, 2931, 2866, 1715 (C=O); ^{1}H NMR (500 MHz, $CDCl_3$) δ_H 0.96 (3H, s, CH_3-oxetane), 1.34 (3H, s, CH_3), 2.32 (1H, d, J = 14.5 Hz, CH_2-oxetane), 2.39

(1H, d, J = 14.5 Hz, CH_2-oxetane), 3.20 (3H, s, N-CH_3), 3.78 (1H, d, J = 5.8 Hz, 1×oxetane-H_2), 3.90 (3H, s, OCH_3), 4.05 (1H, d, J = 5.8 Hz, 1×oxetane-H_2), 4.37 (1H, d, J = 5.8 Hz, 1×oxetane-H_2), 4.46 (1H, d, J = 5.8 Hz, 1×Oxetane-H_2), 6.64 (1H, s, 1×methoxypyridine-*H*), 7.65 (1H, s, 1×methoxypyridine-*H*); ^{13}C NMR (125 MHz, $CDCl_3$) δ_C 22.9 (*C*H_3-oxetane), 26.6 (N-*C*H_3), 27.0 (*C*H_3), 39.2 (*C*-oxetane), 45.5 (pyrrolidinone-*C*H_2-oxetane), 47.3 (*C*(CH_3)CH_2oxetane), 53.7 (O*C*H_3), 83.5 (CH_2-oxetane), 83.8 (CH_2-oxetane), 106.8 (*C*H-methoxypyridine), 124.1 (*C*H-methoxypyridine), 134.6 (C-Ar), 146.6 (C-Ar), 160.4 (C-Ar), 178.7 (C=O); HRMS calcd for $C_{15}H_{21}N_2O_3$ $[M+H]^+$ 277.1547, found 277.1544.

6-(4-Chlorobenzyl)-1,3-dimethyl-3-((3-methyloxetan-3-yl)methyl)-1,6-dihydro-2*H*-pyrrolo[2,3-*c*]pyridine-2,5(3*H*)-dione (22e)

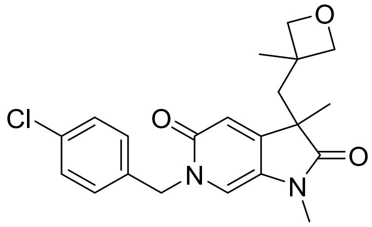

Prepared according to general procedure B using **21e** (18 mg, 0.065 mmol), 1-(bromomethyl)-4-chlorobenzene (27 mg, 0.13 mmol) and MeCN (1 mL). Reaction time: 45 min. Purification by MPLC on SiO_2 (0-6% MeOH/DCM) gave an off-white solid (21 mg, 83%). R_f 0.32 (5% MeOH/DCM); m.p. 167-170 °C; IR ν_{max}/cm^{-1} 3049, 2964, 2923, 2863, 1709 (C=O) 1598 (C=O); ^{1}H NMR (500 MHz, $CDCl_3$) δ_H 1.05 (3H, s, CH_3-oxetane), 1.35 (3H, s, CH_3), 2.22 (1H, d, J = 14.5 Hz, CH_2-oxetane), 2.40 (1H, d, J = 14.5 Hz, CH_2-oxetane), 3.04 (3H, s, N-CH_3), 3.93 (1H, d, J = 5.8 Hz, 1×oxetane-H_2), 4.05 (1H, d, J = 5.8 Hz, 1×oxetane-H_2), 4.44 (1H, d, J = 5.8 Hz, 1×oxetane-H_2), 4.48 (1H, d, J = 5.8 Hz, 1×oxetane-H_2), 4.98 (1H, d, J = 14.6 Hz, 1×4-chlorophenyl-CH_2), 5.21 (1H, d, J = 14.6 Hz, 1×4-chlorophenyl-CH_2), 6.54 (1H, s, 1×pyridone-*H*), 6.64 (1H, s, 1×pyridone-*H*), 7.23-7.24 (2H, m, 2×4-chlorophenyl-H_2), 7.31-7.33 (2H, m, 2×4-chlorophenyl-H_2); ^{13}C NMR (125 MHz, $CDCl_3$) δ_C 23.4 (*C*H_3-oxetane), 26.7 (N-*C*H_3), 27.1 (*C*H_3), 39.4 (*C*-oxetane), 45.7 (pyrrolidinone-*C*H_2-oxetane), 46.9 (*C*(CH_3)CH_2oxetane), 51.7 (N*C*H_2-Ar), 83.6 (CH_2-oxetane), 83.6 (CH_2-oxetane), 113.0 (*C*H-methoxypyridine), 116.3 (*C*H-methoxypyridine), 127.0 (C-Ar), 129.3 (2×C-Ar), 129.5 (2×C-Ar), 134.3 (C-Ar), 135.0 (C-Ar), 150.6 (C-Ar), 161.1 (C-Ar), 177.3 (C=O); HRMS calcd for $C_{21}H_{24}ClN_2O_3$ $[M(^{35}Cl)+H]^+$ 387.1470, found 387.1465.

3-(Cyclopropylmethylene)-5-methoxy-1,3-dihydro-2*H*-pyrrolo[2,3-c]pyridin-2-one (19f)

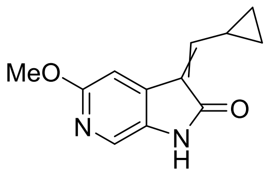

Prepared according to general procedure C using **11** (200 mg, 0.76 mmol), cyclopropylcarboxaldehyde (62 µL, 0.83 mmol, 1.1 eq.), piperidine (225 µL, 2.28 mmol, 3 eq.) and THF (1.5 mL). Purification by MPLC on silica (0-6% MeOH/DCM) gave the title compound as yellow solid comprising a ca. 2.2:1 mixture of stereoisomers (88 mg, 54%); R_f 0.35 (5% MeOH/DCM); ^{1}H NMR (500 MHz; $CDCl_3$) δ_H 0.84-0.89 (2H minor, m, H-cyclopropyl), 0.94-1.00 (2H major, m, H-cyclopropyl), 1.24-1.35 (H, m, H-cyclopropyl), 2.16-2.25 (1H major, m, H-cyclopropyl), 3.34-3.43 (1H minor, m, H-cyclopropyl), 3.89 (3H minor, s, OMe), 3.92 (2H major, s, OMe), 6.33 (1H minor, d, J = 11.2 Hz, H-alkene), 6.59 (1H major, d, J = 11.5 Hz, H-alkene), 6.67 (1H minor, s, H-pyridone), 7.03 (1H major, s, H-pyridone), 6.68 (1H minor, d, J = 0.8 Hz, H-pyridone), 7.73 (1H major, d, J = 0.7 Hz, H-pyridone), 7.85 (1H minor, br s, NH), 7.96 (1H major, br s, NH); ^{13}C NMR (125 MHz; $CDCl_3$) δ_C 11.3, 11.4, 13.1, 14.3, 53.6 (minor), 53.7 (major), 100.5 (minor), 104.8 (major), 124.0 (minor), 124.5 (major), 125.4 (minor), 125.6 (major), 129.9 (minor), 131.1 (major), 134.5 (major), 135.6 (minor), 153.7 (major), 154.2(minor), 160.2 (minor), 160.3 (major), 168.5 (major), 168.9 (minor); MS ES+ 217.1[M+H]$^+$

3-(Cyclopropylmethyl)-5-methoxy-1,3-dihydro-2*H*-pyrrolo[2,3-c]pyridin-2-one (20f)

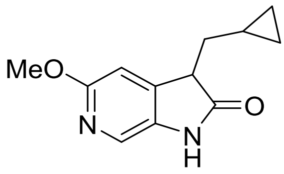

Prepared according to general procedure D, step 1 using **19f** (113 mg, 0.52 mmol) in MeOH (10 mL) and DCM (2 mL), to give a white solid (114 mg, 100%) containing a mixture of product and an inseparable over-reduced by-product (28 mol%). The material was carried forward without purification due to chemical instability on silica. R_f 0.3 (5% MeOH/DCM); MS ES+ 219.2 [M+H]$^+$

3-(Cyclopropylmethyl)-5-methoxy-1,3-dimethyl-1,3-dihydro-2*H*-pyrrolo[2,3-c]pyridin-2-one (21f)

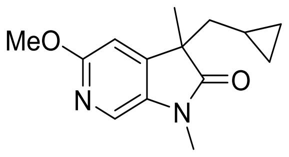

Prepared according to general procedure D, step 2 using **20f** (108 mg, 0.493 mmol), MeI (64 µL, 1.04 mmol, 2.1 eq.), Cs_2CO_3 (370 mg, 1.134 mmol, 2.3 eq) and DMF (8mL). Purification by MPLC on silica (2-25% EtOAc/DCM) gave the target compound as a yellow gum (47 mg, 39%); R_f 0.6 (5% MeOH/DCM); ^{1}H NMR (500 MHz; $CDCl_3$) δ_H -0.10- -0.04 (1H, m, H-cyclopropyl), 0.03-0.10 (1H, m, H-cyclopropyl), 0.18-0.24 (2H, m, H-cyclopropyl), 0.27-0.36 (1H, m, H-cyclopropyl), 1.35 (3H, s, CMe), 1.61 (1H, dd, J = 8.4 and 13.9 Hz, CH_aH_bcPr), 1.90 (1H, dd, J = 5.8 and 13.9 Hz CH_aH_bcPr), 3.23 (3H, s, NMe), 3.92 (3H, s, OMe), 6.67 (1H, d, J = 0.7 Hz, H-pyridone), 7.64 (1H, d, J = 0.7 Hz, H-pyridone); ^{13}C NMR (125 MHz; $CDCl_3$) δ_C 3.8, 3.9, 6.4, 22.9, 26.4, 42.8, 49.2, 53.6, 106.4, 123.6, 135.3, 147.7, 160.5, 179.3; MS ES+ 247.2 $[M+H]^+$

6-(4-Chlorobenzyl)-3-(cyclopropylmethyl)-1,3-dimethyl-1,6-dihydro-2*H*-pyrrolo[2,3-c]pyridine-2,5(3*H*)-dione (22f)

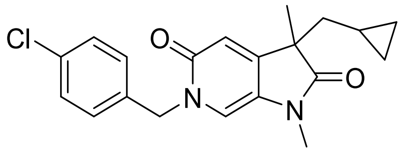

Prepared according to general procedure B using **21f** (42 mg, 0.17 mmol), 4-chlorobenzyl bromide (70 mg, 0.34 mmol, 2 eq.) and MeCN (2 mL). Purification by MPLC on silica (0-3% MeOH/DCM) gave the title compound as a clear glass (30 mg, 49%); R_f 0.25 (5% MeOH/DCM); m.p. 113-116 ºC; IR ν_{max}/cm^{-1} 3041, 2926, 1710 (C=O), 1588 (C=O); ^{1}H NMR (500 MHz; $CDCl_3$) δ_H -0.04-0.02 (1H, m, H-cyclopropyl), 0.04-0.10 (1H, m, H-cyclopropyl), 0.21-0.32 (2H, m, H-cyclopropyl), 0.36-0.45 (1H, m, H-cyclopropyl), 1.35 (3H, s, CMe), 1.64 (1H, dd, J = 8.2 and 13.9 Hz, CH_aH_bcPr), 1.83 (1H, dd, J = 5.8 and 13.9 Hz CH_aH_bcPr), 3.07 (3H, s, NMe), 5.04 (1H, d, J = 14.7 Hz, NCH_aH_bAr), 5.19 (1H, d, J = 14.7 Hz, NCH_aH_bAr), 6.57 (1H, s, H-pyridone), 6.61 (1H, s, H-pyridone), 7.24-7.29 (2H, m, H-Ar), 7.30-7.36 (2H, m, H-Ar); ^{13}C NMR (125 MHz; $CDCl_3$) δ_C 3.8, 4.1, 6.4, 22.9, 26.4, 42.9, 48.8, 51.5, 112.1,

115.9, 127.7, 129.1, 129.4, 134.0, 135.1, 151.5, 161.4, 177.9; HRMS calcd for $C_{20}H_{22}N_2O_2Cl$ $[M+H]^+$ 357.1364, found 357.1361.

1.10 References

1. A. M. Giannetti, in *Methods in Enzymology*, ed. L. C. Kuo, Academic Press, Editon edn., 2011, vol. 493, pp. 169.
2. P. Evans, *Acta Crystallographica Section D*, 2011, **67**, 282.
3. Collaborative Computational Project, *Acta Crystallographica Section D*, 1994, **50**, 760.
4. A. J. McCoy, R. W. Grosse-Kunstleve, P. D. Adams, M. D. Winn, L. C. Storoni and R. J. Read, *Journal of Applied Crystallography*, 2007, **40**, 658.
5. G. N. Murshudov, A. A. Vagin and E. J. Dodson, *Acta Crystallographica Section D*, 1997, **53**, 240.
6. P. Emsley, B. Lohkamp, W. G. Scott and K. Cowtan, *Acta Crystallographica Section D*, 2010, **66**, 486.
7. WO2009/5672, 2009.
8. WO2015/92420, 2015.
